# Effectiveness of stress management and relaxation interventions for management of hypertension and prehypertension: systematic review and network meta-analysis

**DOI:** 10.1136/bmjmed-2024-001098

**Published:** 2025-04-08

**Authors:** Katie E Webster, Monika Halicka, Russell J Bowater, Thomas Parkhouse, Dara Stanescu, Athitya Vel Punniyakotty, Jelena Savović, Alyson Huntley, Sarah Dawson, Christopher E Clark, Rachel Johnson, Julian PT Higgins, Deborah M Caldwell

**Affiliations:** 1 National Institute for Health and Care Research (NIHR) Bristol Evidence Synthesis Group, Population Health Sciences, University of Bristol, Bristol, UK; 2 Population Health Sciences, University of Bristol, Bristol, UK; 3 St Luke’s Campus, University of Exeter, Exeter, UK; 4 National Centre for Infectious Diseases, Singapore; 5 National Institute for Health and Care Research (NIHR) Applied Research Collaboration West, University Hospitals Bristol and Weston NHS Foundation Trust, Bristol, UK; 6 Centre for Academic Primary Care, Population Health Sciences, University of Bristol, Bristol, UK; 7 Health and Community Sciences, University of Exeter, Exeter, UK

**Keywords:** Hypertension

## Abstract

**ABSTRACT:**

**Objective:**

To assess whether relaxation and stress management techniques are useful in reducing blood pressure in individuals with hypertension and prehypertension.

**Design:**

Systematic review and network meta-analysis.

**Data sources:**

Medline, PsycInfo, and CENTRAL (Cochrane Central Register of Controlled Trials) from inception to 23 February 2024, and CINAHL (Cumulative Index to Nursing and Allied Health Literature) from inception to 27 February 2024.

**Eligibility criteria for selecting studies:**

Studies published in English of adults with hypertension (blood pressure ≥140/90 mm Hg) or prehypertension (blood pressure ≥120/80 mm Hg but <140/90 mm Hg). Studies that compared non-pharmacological interventions used to promote relaxation or reduce stress with each other, or with a control group (eg, no intervention, waiting list, or standard care). Where possible, network meta-analysis was used to compare the efficacy of the different interventions. Studies were assessed with the risk of bias 2 tool (RoB2), and those at high risk of bias were excluded from the primary analysis. The certainty of the evidence was assessed with CINeMA (Confidence in Network Meta-Analysis) and GRADE (Grading of Recommendations Assessment, Development, and Evaluation).

**Results:**

182 studies were included (166 for hypertension and 16 for prehypertension). Results from a random effects network meta-analysis showed that, at short term follow-up (≤3 months), most relaxation interventions appeared to have a beneficial effect on systolic and diastolic blood pressure for individuals with hypertension. Between study heterogeneity was moderate (τ=2.62-4.73). Compared with a passive comparator (ie, no intervention, waiting list, or usual care), moderate reductions in systolic blood pressure were found for breathing control (mean difference −6.65 mm Hg, 95% credible interval −10.39 to −2.93), meditation (mean difference −7.71 mm Hg, −14.07 to −1.29), meditative movement (including tai chi and yoga, mean difference −9.58 mm Hg, −12.95 to −6.17), mindfulness (mean difference −9.90 mm Hg, −16.44 to −3.53), music (mean difference −6.61 mm Hg, −11.62 to −1.56), progressive muscle relaxation (mean difference −7.46 mm Hg, −12.15 to −2.96), psychotherapy (mean difference −9.83 mm Hg, −16.24 to −3.43), and multicomponent interventions (mean difference −6.78 mm Hg, −11.59 to −1.99). Reductions were also seen in diastolic blood pressure. Few studies conducted follow-up for more than three months, but effects on blood pressure seemed to lessen over time. Limited data were available for prehypertension; only two studies compared short term follow-up of relaxation therapies with a passive comparator, and the effects on systolic blood pressure were small (mean difference −3.84 mm Hg, 95% credible interval −6.25 to −1.43 for meditative movement; mean difference −0.53 mm Hg, −2.03 to 0.97 for multicomponent intervention). The certainty of the evidence was considered to be very low based on the CINeMA framework, owing to the risk of bias in the primary studies, potential publication bias, and imprecision in the effect estimates.

**Conclusions:**

The results of the study indicated that relaxation and stress management techniques might have beneficial short term effects on blood pressure for people with hypertension, but the effectiveness of these interventions is still uncertain. Future studies should ensure rigorous methods are used to minimise the risk of bias, and a longer duration of follow-up to establish whether these effects persist.

**Systematic review registration:**

PROSPERO CRD42023469128

**WHAT IS ALREADY KNOWN ON THIS TOPIC**

- Relaxation techniques have been suggested to have beneficial effects on blood pressure
- Which methods might be effective, and to what extent blood pressure can be reduced, are unclear

**WHAT THIS STUDY ADDS**

- Most relaxation interventions seemed to reduce blood pressure in people with hypertension in the short term (≤3 months), but the longer term effect was unclear
- For studies that assessed blood pressure with a longer follow-up period, the relaxation techniques had often already been discontinued
- Concerns exist about the potential for bias in many of the primary studies

**HOW THIS STUDY MIGHT AFFECT RESEARCH, PRACTICE, OR POLICY**

- Relaxation methods show promise for people with hypertension
- Future studies should use rigorous methods and a longer follow-up period to establish whether these techniques might be important adjuncts for control of blood pressure

## Introduction

 Hypertension is a major cause of morbidity and mortality worldwide. It is estimated to affect about 30% of the adult population (aged 30-79 years) and is one of the leading attributable causes of deaths in men and women.^1^ Hypertension is known to be associated with an increased risk of cardiovascular, cerebrovascular, and peripheral arterial disease, among other conditions.^2^

Antihypertensive agents are commonly used to treat hypertension and are known to reduce some of the risks associated with the disease.^3^ Adherence to antihypertensive drug treatments is poor, however, with estimates of only 62-75% adherence in high income countries, and even lower estimates in low and middle income countries.^4^ Consequently, interest in alternatives to drug treatments to control blood pressure has increased. Most international guidelines now include recommendations for behavioural and lifestyle changes that can improve control of blood pressure, such as advice on stopping smoking, and recommendations on physical exercise, weight loss, and diet.^5^ High stress levels have long been considered a risk for hypertension and cardiovascular disease,^6^,^8^ and there is interest in whether relaxation techniques can modify this risk. Many different relaxation techniques exist, ranging from simple, self-administered techniques (such as breathing control and mindfulness) to meditative movement practices (including yoga and tai chi), to biofeedback methods. Previous reviews have reported that some of these methods have potentially beneficial effects on blood pressure.^9^,^11^

A prioritisation exercise by the James Lind Alliance identified the use of lifestyle interventions (including relaxation techniques) as one of the top 10 research priorities in hypertension.^12^ In the UK, the National Institute for Health and Care Excellence (NICE) recommended in their 2019 guideline update that research should be conducted to assess whether relaxation interventions are beneficial in hypertension (a 2011 recommendation for relaxation was withdrawn because of insufficient evidence).^13^ Although uncertainty exists about the effectiveness of these techniques, great interest in their potential use for the control of hypertension is evident. Previous reviews have used pairwise meta-analysis methods and focused on individual types of relaxation interventions (eg, yoga or mindfulness), typically compared with a management-as-usual or no intervention comparator.^10 14 15^ Because of the diversity of relaxation interventions available, however, it seems appropriate to assess the comparative effectiveness of these different methods with each other, as well as with frequently used comparators (such as no intervention, usual care, or waiting list controls). This approach can be achieved with network meta-analysis, a statistical technique that enables the simultaneous analysis of studies making different comparisons from a set of eligible interventions. Our aim in this review was to assess the comparative effectiveness of non-pharmacological stress management and relaxation interventions for improving health outcomes for people with a diagnosis of hypertension or prehypertension.

## Methods

### Protocol and reporting methods

The review was registered in PROSPERO (CRD42023469128, https://www.crd.york.ac.uk/PROSPERO/view/CRD42023469128) and conducted according to a prespecified protocol (https://fundingawards.nihr.ac.uk/award/NIHR161214). We adhered to the PRISMA-NMA (Preferred Reporting Items for Systematic Reviews and Meta-Analyses extension statement for network meta-analysis) guideline^16^ to report the review.

### Eligibility criteria

#### Population

We included randomised controlled trials conducted in adults (≥18 years) with hypertension or prehypertension, as defined by the authors, or where an explicit blood pressure threshold was used. The threshold for hypertension was office measured blood pressure ≥140/90 mm Hg (or ambulatory or home blood pressure of ≥135/85 mm Hg). For prehypertension, the threshold was a blood pressure measurement of ≥120/80 mm Hg but <140/90 mm Hg (measured in any setting). We included studies regardless of whether participants were prescribed antihypertensive agents. We excluded studies of people with secondary hypertension, people who were pregnant or had recently given birth, and people with a hypertensive emergency or blood pressure ≥180/120 mm Hg.

#### Interventions

We included any non-pharmacological intervention used to promote relaxation or reduce stress. We prespecified several relevant interventions, but also considered additional interventions retrieved by our searches. We excluded interventions not intended to manage or reduce stress, such as complementary or alternative medicines, nutritional supplements, acupuncture, reflexology, and moxibustion.

#### Comparators

We included studies that compared stress management interventions with each other or with a control group. Eligible controls were passive comparators (no intervention, waiting list, or standard care) or non-specific comparators (sham control). The combined list of stress management interventions and these control comparators formed the decision set for the review (ie, those comparisons of primary interest in the study). We also included studies that compared stress management interventions with alternative interventions, such as drug treatment and exercise. These studies were used to enhance the connectivity of the network and provide further indirect evidence on the comparisons of interest (online supplemental information). The relative efficacy of these additional interventions, however, was not the primary focus of this review.

#### Outcomes

The primary outcomes were systolic and diastolic blood pressure reported at ≤3 months (short term), >3 to 12 months (medium term), and >12 months (long term). Secondary outcomes were mortality, cerebrovascular disease, ischaemic heart disease, heart failure, vascular procedures, and economic outcomes.

### Search strategy

We searched Medline, PsycInfo, and CENTRAL (Cochrane Central Register of Controlled Trials) from inception to 23 February 2024, and CINAHL (Cumulative Index to Nursing and Allied Health Literature) from inception to 27 February 2024. Online supplementary information has full details of the search strategies for each database. We did not apply date restrictions but we limited our search to reports published in English. We excluded preprints, conference abstracts, dissertations and theses, and ongoing trial protocols. To identify published or unpublished research beyond our main searches, we scanned the reference lists of included studies and relevant systematic reviews.

### Screening and inclusion assessment

Titles and abstracts were screened by at least two reviewers (of KEW, MH, and TP). The full text of any study that seemed relevant was retrieved. Selection of included studies was then carried out independently by two reviewers (of KEW, MH, and TP). Any differences were resolved by consensus or by discussion with other authors (DMC and JS).

### Data extraction and management

A Microsoft Excel form was developed for data extraction and piloted on a small number of studies. Data were extracted by one author (KEW or MH) and checked in detail by a second author (KEW or MH). We collected data on inclusion and exclusion criteria, personal characteristics of participants, comorbidities, use of antihypertensive drug treatments, and severity of hypertension or prehypertension (according to blood pressure measurements). We used the PROGRESS-Plus framework to identify characteristics associated with health inequity.^17 18^ We collected details on the nature of the interventions and comparators, and the method used to measure blood pressure.

For continuous outcomes, data were extracted at each time point for number of participants, mean values, and relevant measures of variance (ie, standard deviation (SD), standard error, or confidence intervals). Where reported, we extracted both endpoint data and change-from-baseline data. For synthesis, we prioritised estimates of arm level mean change from baseline and standard errors in accordance with guidance from the NICE guidelines technical support unit.^19^ For studies that did not report change from baseline or its standard error, we applied a prespecified imputation hierarchy.^19 20^ Change from baseline and standard error were derived from reported baseline and follow-up means and SDs, assuming a before-after correlation of 0.5. Online supplementary information has full details on data extraction.

After data extraction, similar interventions were grouped according to their main features. The categories identified were autogenic training, biofeedback, breathing control, hypnosis, massage therapy, meditation, meditative movement, mindfulness, multicomponent interventions, music, progressive muscle relaxation, and psychotherapy. This grouping was informed by the classes of relevant interventions specified in the protocol, but was also driven by the similarities and differences identified between interventions used in different studies (online supplemental table S1).

### Risk of bias assessment

Risk of bias for the outcomes systolic and diastolic blood pressure was assessed at each time point with the risk of bias tool, RoB2.^21^ Most studies were assessed independently by two of the authors (KEW, MH, DS, and AVP). Because the ratings were mostly in agreement, the remaining studies were assessed by one author (KEW or MH), and ratings were checked in detail by a second author (MH or KEW). Any discrepancies were resolved by consensus, or with a third author if required (DMC or JS).

### Evidence synthesis

To assess connectedness, network plots were drawn in R version 4.3.1^22^ with the multinma package^23^ that estimates models in a bayesian framework using Stan.^24^ Studies contributing to disconnected comparisons were excluded from the statistical analysis and summarised narratively. Transitivity was assessed before analysis by comparing potential effect modifiers (such as age, use of antihypertensive drugs, and duration of the intervention) across comparisons with visual and descriptive summaries. If evidence of intransitivity had been identified, we planned to conduct pairwise or narrative synthesis instead of a network meta-analysis.

We conducted separate analyses for individuals with hypertension and prehypertension. Studies that recruited a mixed population (participants with hypertension and prehypertension) were included in the hypertension analyses. Studies considered to be at high risk of bias were excluded from the primary analyses.

### Network meta-analysis model fitting and selection

Random effects models assuming a common between study heterogeneity parameter were fitted for all analyses with multinma, where sufficient data were available and transitivity was considered plausible. Fixed effect models were implemented as a sensitivity analysis and as part of model selection checks. Inconsistency was assessed with both global and local approaches.^25^ We specified uninformative (vague) prior distributions for the intercept (normal (µ=0, σ=100)), treatment effect (normal (µ=0, σ=100)), and a weakly informative between study heterogeneity parameter (half-normal (µ=0, σ=10)). Online supplemental tables S4–S9 have further information on checking assumptions, convergence, and the statistical models fitted. Intervention effects are reported as mean differences in change from baseline in systolic or diastolic blood pressure relative to the reference intervention (passive comparator), with the limits of the 95% credible intervals defined as the 2.5th and 97.5th centiles of the posterior distribution concerned. If network meta-analysis was not possible, we aimed to present pairwise random effects meta-analyses instead, with meta in R.^26^ For comparisons informed by three or fewer studies, however, we did not present the pooled result because of concerns that the heterogeneity estimate might be unreliable.

### Subgroup and sensitivity analyses

We prespecified four subgroup analyses to explore potential heterogeneity in the results.

- Drug treatment of hypertension. Studies were grouped according to the proportion of participants prescribed antihypertensive drugs, categorised as none, some (any proportion from >0% to <100% of the sample), all, or not reported.
- Country level economic resource. Countries in which the included studies were conducted were classified into lower (low income and lower middle income) and higher (upper middle income and high income) based on the country level economic resource according to the World Bank.^27^
- Age. Studies were grouped according to the average age of participants: ≥75 years versus <75 years.
- Severity of hypertension (grade 1 <160/100 mm Hg *v* grade 2 ≥160/100 mm Hg).

We also conducted two prespecified sensitivity analyses to consider the robustness of the results: including studies considered to be at high risk of bias in the analysis; and restricting the analysis to studies in which all participants met the specific blood pressure thresholds (ie, removing studies that recruited individuals with both hypertension and prehypertension from the hypertension analyses).

Three further sensitivity analyses were conducted because of concerns that arose during the preparation of the review: an alternative grouping method for interventions including biofeedback; the use of a different correlation between measurements before and after the intervention; and excluding studies with imputed data. The supplementary methods in the online supplemental information has more information.

### Assessment of certainty of evidence

We assessed the certainty of the evidence for our primary analyses with the Confidence in Network Meta-Analysis (CINeMA)^28^ framework for results of the network meta-analyses and with Grading of Recommendations Assessment, Development, and Evaluation (GRADE)^29 30^ for other results. The domains considered were risk of bias, publication bias, imprecision in effect estimates, indirectness of the evidence, and between study heterogeneity. For outcomes assessed with a network meta-analysis, we also considered inconsistency (incoherence) between the direct and indirect evidence.

The minimally important difference for systolic blood pressure was considered to be 5 mm Hg because a reduction of this magnitude correlates with improved cardiovascular outcomes.^31^ We could not identify a recently published minimally important difference for diastolic blood pressure, and therefore we used a distribution based approach.^32^ This approach indicated that a change in diastolic blood pressure of about 3 mm Hg might be regarded as meaningful.

### Patient and public involvement

While developing the research question and protocol for this review, we held two online meetings with four people with lived experience of hypertension, including individuals with hypertension and carers for those with high blood pressure. These individuals provided suggestions on interventions that could be included in the review, how these interventions might be used, and perspectives on the timing and nature of important outcomes. At the end of the review process, the advisory group also discussed and commented on the findings and provided advice on creating a plain language summary. Findings from this review will be disseminated locally and nationally via engagement with charities supporting people with hypertension and public outreach events, including the Bristol Heart Institute Festival (held in March 2025).

## Results

### Search results

**Figure 1:**
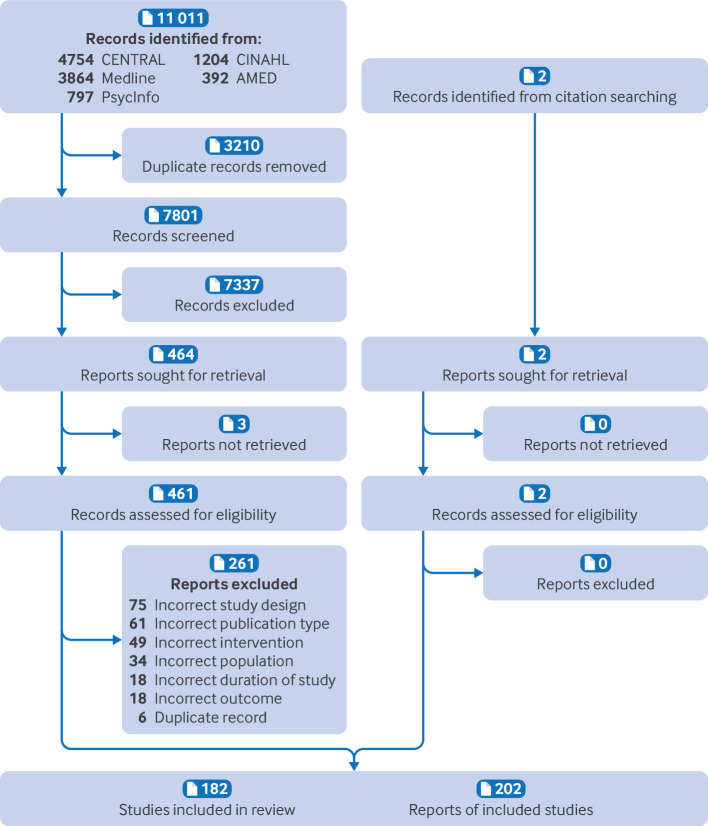
Preferred Reporting Items for Systematic Reviews and Meta-Analyses (PRISMA) flow diagram.^79^ CENTRAL=Cochrane Central Register of Controlled Trials; CINAHL=Cumulative Index to Nursing and Allied Health Literature; AMED=Allied and Complementary Medicine Database

We screened 7801 records and assessed the full text of 461 articles. In the final review, we included 182 studies, published in 202 separate reports (figure 1). One study^33^ separately randomised two groups of participants to different interventions and for the purposes of our analysis, these were considered to be separate studies. Online supplemental table S3 lists the references and characteristics of all of the included studies and online supplemental table S2 has a list of the excluded studies.

The studies predominantly assessed individuals with hypertension (139 studies specifically included people with hypertension, and 27 studies included a mixed population of those with prehypertension and hypertension). A smaller number of studies specifically included people with prehypertension (16 studies). Characteristics of the included studies are presented in online supplemental table S3.

### Risk of bias assessment

All included studies had at least some concerns of risk of bias for the outcomes systolic and diastolic blood pressure (online supplemental tables S20–S28). Of the 182 studies included, 99 were considered to have a high risk of bias and 71 had some concerns for blood pressure outcomes at all time points. Eight studies had a mixed rating, with a different risk of bias at separate follow-up times. Four studies did not provide any numeric data for analysis and were therefore not rated with the RoB 2 tool.^34^,^37^

### Hypertension: primary analyses

A total of 163 studies provided numeric data for the analysis of the primary outcomes. At short term follow-up, systolic blood pressure was reported in 139 studies, of which 137 also reported diastolic blood pressure. Sixty-five studies reported both outcomes at medium term follow-up and seven studies reported both outcomes at long term follow-up. Only 10 studies reported data at both short term and medium term follow-up, and one study reported data at medium term and long term follow-up. Where reported, participants had a mean age of 53.16 (SD 9.29) years and the sex distribution was balanced (52% men). Use of antihypertensive agents varied across the included studies (in 23% of studies, all participants were receiving antihypertensive drugs; in 39%, some participants were receiving antihypertensive drugs; in 21%, participants were not receiving antihypertensive drugs; and in 15% of studies, this information was not reported). In two studies,^38 39^ participants were not receiving drug treatments at baseline, but were randomised to receive drug treatments as part of the trial. Across the studies, the intervention period lasted from five days to 12 months (median two months, mean 2.66 months, SD 1.90 months). The transitivity assumption for treatment comparisons was considered plausible, after assessment of baseline characteristics by comparison, including blood pressure.

We report the results of the primary analyses, considering the effects of relaxation and stress management interventions relative to a passive comparator (no intervention, usual care, or waiting list control), expressed as the mean difference (95% credible intervals). The supplementary materials have more details on the results of each analysis, including the relative effects between all comparisons (online supplemental tables S11–S16), model fit statistics and details of convergence (online supplemental tables S4–S9), results of subgroup and sensitivity analyses (online supplemental table S10), and GRADE assessments for comparisons that were disconnected from the networks (online supplemental table S30).

#### Short term follow-up

**Figure 2:**
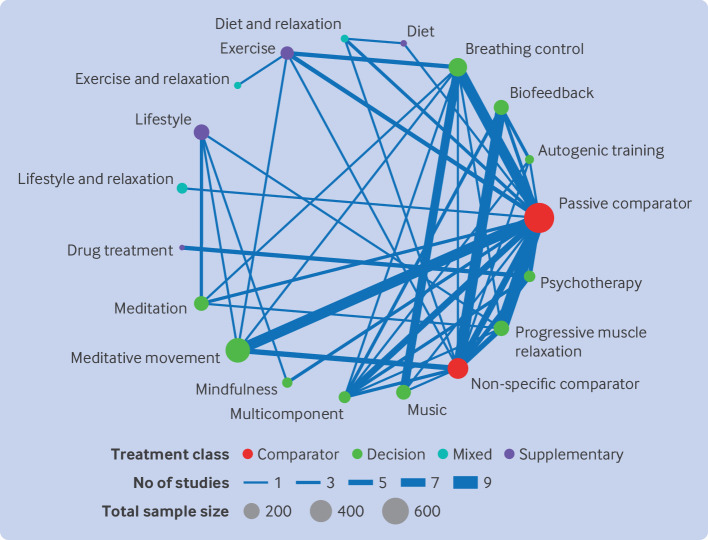
Network plot for hypertension, short term follow-up; 54 studies were included

We included 54 studies of 19 active interventions in the network meta-analysis (83 studies were excluded owing to a high risk of bias, online supplemental tables S20 and S21); two studies were disconnected from the network.^40 41^ Figure 2 shows the network plot. A passive comparator was the most commonly used control arm (n=28 trials). The most commonly included interventions were breathing control (13 trials), meditative movement (11 trials), biofeedback (eight trials), progressive muscle relaxation (seven trials), and music (seven trials). At the time blood pressure was measured, relaxation interventions were continuing in 42 trials (78%) but had already stopped in four trials (7%). In the remaining eight trials (15%), the interventions were ongoing, but the frequency or intensity of the relaxation practice had already been reduced.

Model fit and selection statistics indicated that a random effects model assuming consistency was appropriate (online supplemental tables S4 and S5 in online supplemental materials). The between study SD (τ) indicated moderate heterogeneity for both systolic blood pressure (posterior median 4.73 mm Hg, 95% credible intervals 3.59 to 6.27) and diastolic blood pressure (2.62 mm Hg, 1.85 to 3.60).

**Figure 3:**
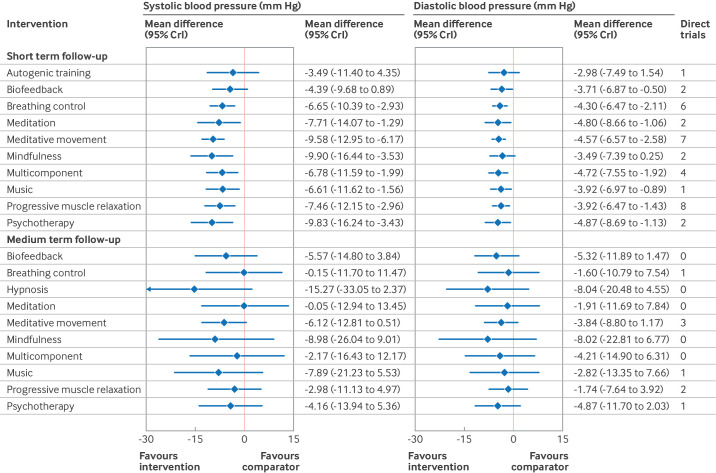
Primary random effects network meta-analysis estimates of relative effects of relaxation interventions versus passive comparator on systolic and diastolic blood pressure at short term (≤3 months) and medium term (>3 months to ≤12 months) follow-up, in individuals with hypertension. Effect estimates were based on a combination of direct and indirect evidence. Direct trials represent the number of head-to-head comparisons. 54 trials were included in the analysis of short term follow-up and 21 trials of medium term follow-up. CrI, credible interval

Figure 3 and online supplemental table S10 in the online supplemental materials present the relative effects of each relaxation intervention versus a passive comparator. These results suggest that breathing control, meditation, meditative movement, mindfulness, multicomponent interventions, music, progressive muscle relaxation, and psychotherapeutic approaches might reduce systolic and diastolic blood pressure relative to a passive comparator. The point estimate for each of these interventions exceeded the minimally important difference, although the credible intervals included the possibility of a smaller effect. Biofeedback might also reduce diastolic blood pressure by more than the minimally important difference. Relative to a passive comparator, the point estimates for autogenic training (systolic and diastolic blood pressure) and biofeedback (systolic blood pressure) did not exceed the minimally important difference and therefore these interventions might not have a meaningful effect on blood pressure. The evidence was assessed as very low certainty, however, because of the risk of bias in the primary studies, potential publication bias, imprecision in the effect estimates, and indirectness (online supplemental figures S1 and S2).

#### Medium term follow-up

**Figure 4:**
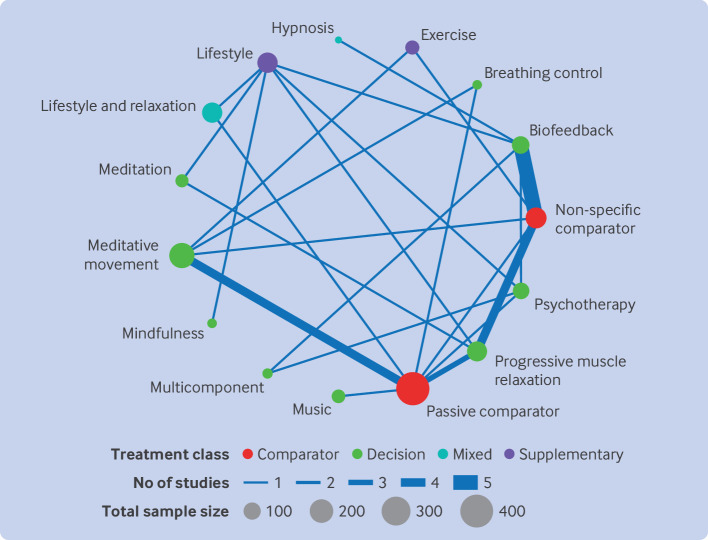
Network plot for hypertension, medium term follow-up; 21 studies were included

We included 21 studies of 15 interventions in the primary analysis at this time point (42 studies were excluded because of a high risk of bias, online supplemental tables S22 and S23); two studies were disconnected from the network.^40 42^ Figure 4 shows the network plot. A passive comparator was the most commonly used control arm (n=8 trials). The most commonly studied interventions were biofeedback (n=7 trials), meditative movement (n=4 trials), and progressive muscle relaxation (n=4 trials). At the time blood pressure was measured, relaxation interventions were continuing in four trials (19%) but had already stopped in 14 trials (67%). The interventions were ongoing, but at a lower frequency or intensity, in the remaining three trials (14%).

Model fit was not materially different between consistency or inconsistency models (online supplemental tables S6 and S7 in online supplemental materialsmaterials). Between study heterogeneity (τ) was smaller for the inconsistency model, however, suggesting potential inconsistency. Further investigation indicated that this inconsistency could be attributed to one study.^43^ A post hoc sensitivity analysis, excluding this study, substantially improved model fit and reduced between study heterogeneity in the consistency model (online supplemental tables S6, S7, and S17 in online supplemental materialsupplementary materials). Re-examination of this study did not identify a valid clinical reason for exclusion, and the study is included in the results reported here.

Figure 3 and online supplemental table S10 in the online supplemental materials present the relative effects of each relaxation intervention compared with a passive comparator. Statistical evidence of effectiveness for any intervention at the medium time point was absent and, for all treatment effects, the certainty of the evidence was very low (online supplemental figures S3 and S4).

#### Long term follow-up

**Figure 5:**
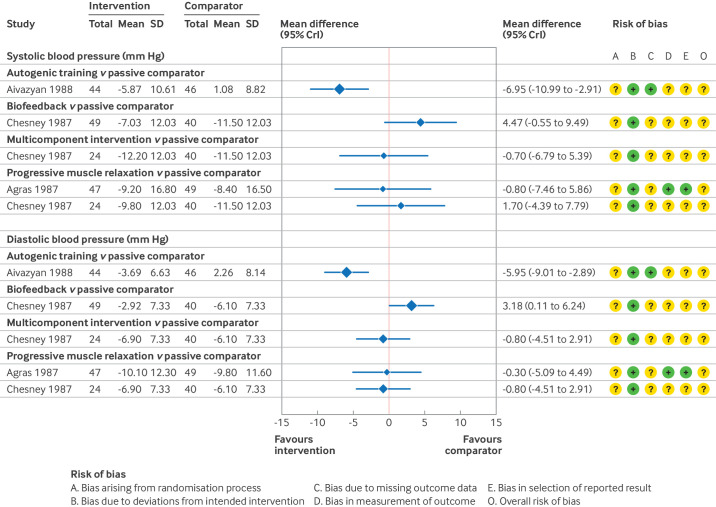
Study level estimates and risk of bias assessments for relaxation interventions in individuals with hypertension at long term follow-up.^80^,^82^ SD=standard deviation; CrI=credible interval

Systolic and diastolic blood pressure outcomes were reported in seven studies, of which three were excluded from the primary analysis because of a high risk of bias (online supplemental table S24); one study was disconnected from the network.^44^ The network consisted of the remaining three studies, comparing six interventions (online supplemental figure S7). Given the challenges of reliably estimating between study heterogeneity from such a sparse network, figure 5 reports study level direct estimates only. Online supplemental tables S8–S10 report the model fit statistics and results of the network meta-analysis. At this time point, relaxation interventions had either stopped (one trial), or the frequency or intensity of practice had been reduced since the start of the trial (three trials).

Relative to a passive comparator, we found that autogenic training might result in a reduction in both systolic and diastolic blood pressure, although the certainty of the evidence was low. The point estimates exceeded the minimally important differences, indicating that this result could be a meaningful reduction. Confidence intervals for other interventions were wide, and the certainty of the evidence was low or very low (online supplemental table S29), and therefore we cannot draw firm conclusions.

### Hypertension: sensitivity analyses

Online supplemental materials has full details and all findings of the sensitivity analyses. As planned in the review protocol, we conducted sensitivity analyses at each time point, including all studies, regardless of the risk of bias (online supplemental table S10 and online supplemental figures S5 and S6). Overall, the effect estimates from these analyses were similar, but more precise compared with the primary analysis. The remaining sensitivity analyses also showed similar results to the main analyses, with no substantial changes in the effect estimates for individual relaxation therapies (online supplemental tables S10 and S17).

### Hypertension: subgroup analyses

Online supplemental tables S4–S10 have full details and all findings of the subgroup analyses. We assessed the effect of different relaxation interventions according to whether participants were receiving antihypertensive drug treatment (online supplemental table S10 and online supplemental figures S8 and S9). Relatively few studies contributed to these analyses, the credible intervals were wide, and so we cannot draw firm conclusions. For short term follow-up, however, most relaxation interventions seemed to reduce systolic blood pressure, regardless of the drug treatment status of participants. The only exception was biofeedback, which seemed to be less effective in individuals receiving antihypertensive agents. We also conducted a subgroup analysis according to the country level economic resource. Where it was possible to fit a network meta-analysis, the results were broadly similar to the primary analyses (online supplemental table S10 and online supplemental figures S10 and S11).

We planned to conduct a subgroup analysis according to the age of participants, but only two studies recruited participants who were, on average, aged ≥75 years.^45 46^ After removing these studies from the analyses, the overall results were essentially unchanged (online supplemental table S10). We also intended to conduct a subgroup analysis according to severity of hypertension (grade 1 *v* grade 2). Only 10 studies reported details on the proportion of participants belonging to each category, however, and therefore this subgroup analysis was not possible.

### Prehypertension: primary analyses

Sixteen studies were identified that included participants with prehypertension.^47^,^62^ In general, the population was younger (mean age 40.48 years, SD 11.69). Most studies included men and women (mean 56% men). Most studies stated that no participants were taking antihypertensive agents (four studies did not provide information on the use of antihypertensive drugs^48 55 59 62^). The duration of the intervention ranged from 15 days to 36 months (median two months, mean 6.54, SD 9.39 months).

**Figure 6:**
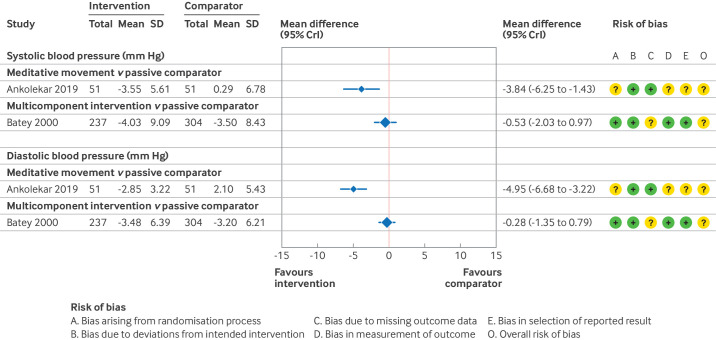
Study level estimates and risk of bias assessments for relaxation interventions in individuals with pre-hypertension at short term follow-up.^48 49^ SD=standard deviation; CrI=credible interval

Given the diversity of the interventions and comparators used across these studies, we could not conduct a network meta-analysis. Instead, forest plots are presented, showing the mean difference in effect between relaxation interventions and a passive comparator, for those studies at lower risk of bias. Figures6  7 show the estimated effects for short term and medium term follow-up, as well as a summary of the risk of bias assessments (full details in online supplemental tables S25–S27). Similar to the hypertension analyses, we report the effects of relaxation and stress management interventions relative to a passive comparator. Online supplemental figures S12–S17 show the results for other comparisons and online supplemental table S31 details the GRADE assessments.

**Figure 7:**
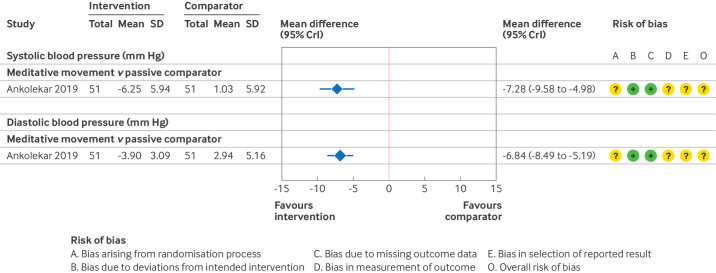
Study level estimates and risk of bias assessments for relaxation interventions in individuals with prehypertension at medium term follow-up.^48^ SD=standard deviation; CrI=credible interval

#### Short term, medium term, and long term follow-up

For short term follow-up, only two studies compared a relaxation intervention with a passive comparator (figure 5).^48 49^ We found that meditative movement might result in a reduction in both systolic and diastolic blood pressure at short term follow-up (low certainty of evidence).^48^ A multicomponent relaxation intervention resulted in unimportant changes in blood pressure (low certainty of evidence).^49^

We found that meditative movement might also result in a reduction in both systolic and diastolic blood pressure at medium term follow-up (low certainty of evidence).^48^ No other studies compared a relaxation intervention with a passive comparator (figure 5). No studies at low risk of bias, or with some concerns over risk of bias, reported long term follow-up.

### Prehypertension: sensitivity and subgroup analyses

Online supplemental materials has information on sensitivity and subgroup analyses.

### Secondary outcomes

Our review protocol specified secondary outcomes of interest, including mortality, cerebrovascular disease, ischaemic heart disease, heart failure, vascular procedures, and economic outcomes. These outcomes were found to be reported infrequently, however, and we could not conduct any meaningful analysis. Online supplemental tables S18 and S19 have further details, with risk of bias assessments in online supplemental table S28 and GRADE assessments in online supplemental table S32.

### Equality, diversity, and inclusion perspectives

We extracted data from the included studies on characteristics that might be associated with unequal health status, with the PROGRESS-Plus framework, but we found that many studies failed to report these features. Online supplemental table S33 has a full summary of the PROGRESS-Plus characteristics and we present a brief summary here.

Mean age of participants in studies that reported age was 52.12 years. Most studies reported the sex of participants, showing a slight preponderance of men (52%). Few studies reported the recruitment setting, but those that did tended to be conducted in urban areas. Most studies (69.7%) did not provide information on participants’ ethnic group. When reported, participants were predominantly white, and few studies recruited participants from other ethnic groups. Of the studies that reported employment, most recruited a high proportion of participants who were working, and in studies that reported social capital, most participants were married or cohabiting. Only 12 studies provided any information on socioeconomic status, and one study reported on religion. Many studies excluded individuals with mental health disorders. A smaller number of studies also excluded those who had difficulty operating or accessing technological devices, people with lower educational qualifications, people with limited mobility, or individuals with sight or hearing difficulties.

## Discussion

### Principal findings

The results of our systematic review and network meta-analysis indicate that relaxation or stress management techniques might result in meaningful reductions in blood pressure at up to three months of follow-up. Uncertainty exists about this effect, however, because of the risk of bias in the primary studies, the potential for publication bias in this area, and imprecision in the effect estimates, meaning that the observed changes in blood pressure might be too small to affect cardiovascular or cerebrovascular outcomes. The effect on blood pressure could also diminish over time but we have less confidence in these results because few studies conducted long term follow-up and we did not explicitly analyse the effects of treatment over time. We found that many relaxation interventions had already stopped, or their frequency had been reduced, before later follow-up assessments were undertaken. This finding might have had a considerable effect on their reported efficacy.

### Limitations of this study

This review had some limitations. Because of resource limitations, we did not carry out searches of the grey literature or translate studies that were not published in English. The nature of these interventions is such that prospective trial registration and publication of the studies, regardless of the findings, might not be widespread and consequently, this potential publication bias could have affected our findings. Inclusion of non-English language studies, in general, however, has been suggested to have limited effect on overall effect estimates.^63^ Descriptions of relaxation interventions were sometimes incomplete or sparse, meaning that grouping studies that used equivalent relaxation techniques was challenging. We could not contact all study authors to establish full details of the interventions used.

Also, we did not consider adverse effects. Others have noted that adverse effects tend to be poorly reported alongside these types of interventions.^64 65^ Although it might seem unlikely that substantial harm will result from stress management and relaxation interventions, reports exist of important adverse effects, including musculoskeletal and respiratory problems.^65^,^67^ The suitability of different relaxation interventions should therefore be assessed on an individual basis, to minimise the potential for harm. Inclusion of many different interventions in this network meta-analysis, however, most of which were shown to have some beneficial effects on blood pressure, suggests that individuals might be able to select the relaxation therapy that suits them best. Over time, clinical assessment of hypertension has moved away from focusing on one risk factor (blood pressure) towards an approach that includes multiple risk factors when assessing the risk of cardiovascular disease (with tools such as the cardiovascular risk score QRISK^68^). Ideally, we would have assessed the effect of relaxation techniques for those at different levels of risk of cardiovascular disease, according to these tools. But most studies did not report information on the risk of cardiovascular disease, perhaps because they were published before the routine use of these tools. Finally, we chose to analyse the outcomes over three separate follow-up periods. An alternative could be to include multiple time points in the same analysis and account for their correlation, with multivariate network meta-analysis. This approach, however, was not feasible because only 11 studies contributed data to more than one follow-up period.

We noted several shortcomings in the evidence base. Few studies included data on cardiovascular events or mortality alongside data on blood pressure. Treating hypertension is necessary because of its recognised effect on morbidity and mortality. Assessing whether relaxation interventions also have an effect on these outcomes is therefore critical. Longer term follow-up is clearly necessary to acquire sufficient data for these rarer outcomes. The duration of relaxation interventions was typically short, even for studies that had longer term follow-up. Few studies conducted follow-up for >1 year and the effect on blood pressure at these later time points was much less certain. Hypertension is a chronic condition, likely to require long term drug treatments or behavioural changes. As such, interventions that are used for a brief period, or provide only short term benefits, are unlikely to be clinically useful. Too few studies exist, however, to assess whether the beneficial effects of relaxation are maintained when the techniques are practised for longer than three months. Future studies must clearly report whether participants were still using relaxation methods at the time of the outcome assessment, with details on adherence to the relaxation schedule. These factors might strongly influence the effectiveness of the different relaxation and stress management techniques.

Data on costs and the cost effectiveness of these therapies were also lacking. Costs of relaxation therapies can vary widely depending on the nature of the intervention. Some interventions might incur little or no associated cost, such as self-guided breathing exercises or listening to music, but interventions requiring professional facilitators on a regular basis might be costly. Delivering these interventions in groups is effective and could mitigate costs, although cost effectiveness has not been shown so far.^69^ Nonetheless, if effective, these interventions could also be associated with reductions in healthcare appointments, antihypertensive prescribing, and associated side effects related to drug treatment use. Relaxation therapies might also have wider benefits for both physical and psychological wellbeing,^70 71^ which could result in further improvement in health outcomes and greater cost effectiveness.

Despite removing studies at high risk of bias from the primary analyses, we had concerns over the potential for bias in the remaining studies. For many, the description of randomisation and allocation to intervention or comparator groups was insufficient, raising concerns about the randomisation process. Measuring blood pressure was sometimes not robust. Outcome assessors were frequently aware of the treatment allocation for participants (ie, lack of blinding), and many studies did not use a formal protocol for measuring blood pressure. Finally, missing data was a concern, with relatively large numbers of drop-outs. Adequate randomisation, blinding of outcome assessors and analysts, and use of a blood pressure measurement protocol should be straightforward to implement in future studies. Missing outcome data could be a persistent problem unless efforts are made to encourage follow-up for all participants.

As with all interventions, assessing whether they have a meaningful effect, and not only a statistically significant effect, on an outcome is important.^72^ For many of our results, the wide credible intervals meant that it was not clear whether the interventions had a major effect (reduced systolic blood pressure by >5 mm Hg) or a trivial effect (reduced blood pressure by ≤5 mm Hg). This doubt was reflected in the CINeMA and GRADE ratings of the certainty of the evidence. Some studies have indicated that smaller changes in systolic blood pressure might also have substantial effects on cardiovascular outcomes.^73 74^ If this finding were true, it would give us more confidence that these interventions have an important effect on blood pressure.

Because of the limited data reported, we could not draw firm conclusions about aspects of equality, diversity, and inclusion in this review, but some features might be useful to consider when planning future studies. Mean age across the included studies was about 52 years, despite the fact that the highest prevalence of hypertension is in those aged >60 years.^75 76^ This finding could reflect an assumption that younger participants might be more able and willing to undertake stress management interventions, or prefer these to drug treatments. Also, individuals from non-white ethnic backgrounds were under-represented in the review, despite these groups being at greater risk of hypertension.^77 78^ Some groups who might struggle to access healthcare also seemed to be under-represented in the studies (eg, those with mental health disorders, individuals in rural settings, and people who were unemployed or retired). Finally, interventions were sometimes not considered suitable for individuals with physical disabilities (mobility, sight, or hearing problems), resulting in the exclusion of these participants from the studies, rather than modifying the interventions to allow for their inclusion.

### Conclusions

The results of our study indicated that many relaxation interventions show promise for reducing blood pressure in the short term but the longer term effects are unclear. Future studies in this area should include adequate follow-up to establish whether the effects on blood pressure persist over time, both while the relaxation interventions are ongoing and after they have been completed. Researchers should also use rigorous study methods and reporting to minimise the risk of bias in the results. Finally, we encourage researchers to assess all relevant outcomes, including cardiovascular events and adverse events, as well as blood pressure itself.

## Supplementary material

10.1136/bmjmed-2024-001098 online supplemental file 1

10.1136/bmjmed-2024-001098 online supplemental file 2

## Data Availability

Data are available upon reasonable request.

## References

[1] Zhou B, Carrillo-Larco RM, Danaei G (2021). Worldwide trends in hypertension prevalence and progress in treatment and control from 1990 to 2019: a pooled analysis of 1201 population-representative studies with 104 million participants. Lancet.

[2] Rapsomaniki E, Timmis A, George J (2014). Blood pressure and incidence of twelve cardiovascular diseases: lifetime risks, healthy life-years lost, and age-specific associations in 1·25 million people. Lancet.

[3] Law MR, Morris JK, Wald NJ (2009). Use of blood pressure lowering drugs in the prevention of cardiovascular disease: meta-analysis of 147 randomised trials in the context of expectations from prospective epidemiological studies. BMJ.

[4] Lee EKP, Poon P, Yip BHK (2022). Global Burden, Regional Differences, Trends, and Health Consequences of Medication Nonadherence for Hypertension During 2010 to 2020: A Meta-Analysis Involving 27 Million Patients. J Am Heart Assoc.

[5] Maniero C, Lopuszko A, Papalois K-B (2023). Non-pharmacological factors for hypertension management: a systematic review of international guidelines. Eur J Prev Cardiol.

[6] Kivimäki M, Steptoe A (2018). Effects of stress on the development and progression of cardiovascular disease. Nat Rev Cardiol.

[7] Liu M-Y, Li N, Li WA (2017). Association between psychosocial stress and hypertension: a systematic review and meta-analysis. Neurol Res.

[8] Sparrenberger F, Cichelero FT, Ascoli AM (2009). Does psychosocial stress cause hypertension? A systematic review of observational studies. J Hum Hypertens.

[9] Dickinson HO, Campbell F, Beyer FR (2008). Relaxation therapies for the management of primary hypertension in adults. Cochrane Database Syst Rev.

[10] Hagins M, States R, Selfe T (2013). Effectiveness of yoga for hypertension: systematic review and meta-analysis. Evid Based Complement Alternat Med.

[11] Zhong D, Li J, Yang H (2020). Tai Chi for Essential Hypertension: a Systematic Review of Randomized Controlled Trials. Curr Hypertens Rep.

[12] Khan N, Bacon SL, Khan S (2017). Hypertension management research priorities from patients, caregivers, and healthcare providers: A report from the Hypertension Canada Priority Setting Partnership Group. J Clin Hypertens.

[13] NICE (2019). Hypertension in adults: diagnosis and management. https://www.nice.org.uk/guidance/NG136.

[14] Geiger C, Cramer H, Dobos G (2023). A systematic review and meta-analysis of mindfulness-based stress reduction for arterial hypertension. J Hum Hypertens.

[15] de Freitas Gonçalves KS, Queiroz Godoy Daniel AC, Tatagiba Lamas JL (2022). Device and nondevice-guided slow breathing to reduce blood pressure in hypertensive patients: A systematic review and meta-analysis. Health Sci Rep.

[16] Hutton B, Salanti G, Caldwell DM (2015). The PRISMA Extension Statement for Reporting of Systematic Reviews Incorporating Network Meta-analyses of Health Care Interventions: Checklist and Explanations. Ann Intern Med.

[17] Evans T, Brown H (2003). Road traffic crashes: operationalizing equity in the context of health sector reform. Inj Control Saf Promot.

[18] O’Neill J, Tabish H, Welch V (2014). Applying an equity lens to interventions: using PROGRESS ensures consideration of socially stratifying factors to illuminate inequities in health. J Clin Epidemiol.

[19] Daly C, Welton NJ, Dias S (2021). NICE guidelines technical support unit: meta-analysis of continuous outcomes. Guideline methodology document 2. https://www.bristol.ac.uk/media-library/sites/social-community-medicine/documents/mpes/gmd-2-continuous-jan2021.pdf.

[20] Higgins J, Li T, Deeks JJ, Higgins J, Thomas J, Chandler J (2023). Cochrane Handbook for Systematic Reviews of Interventions 64 (updated August 2023).

[21] Sterne JAC, Savović J, Page MJ (2019). RoB 2: a revised tool for assessing risk of bias in randomised trials. BMJ.

[22] R Core Team (2023). R: a language and environment for statistical computing. https://www.R-project.org/.

[23] Phillippo DM (2024). Multinma: bayesian network meta-analysis of individual and aggregate data. r package 0.6.1.

[24] Carpenter B, Gelman A, Hoffman MD (2017). Stan: A Probabilistic Programming Language. J Stat Softw.

[25] Dias S, Welton NJ, Sutton AJ (2014). NICE DSU technical support document 2: a generalised linear modelling framework for pairwise and network meta-analysis of randomised controlled trials. https://www.ncbi.nlm.nih.gov/books/NBK310366/pdf/Bookshelf_NBK310366.pdf.

[26] Balduzzi S, Rücker G, Schwarzer G (2019). How to perform a meta-analysis with R: a practical tutorial. Evid Based Ment Health.

[27] The World Bank (2024). World Bank country and lending groups. https://datahelpdesk.worldbank.org/knowledgebase/articles/906519-world-bank-country-and-lending-groups.

[28] Nikolakopoulou A, Higgins JPT, Papakonstantinou T (2020). CINeMA: An approach for assessing confidence in the results of a network meta-analysis. PLoS Med.

[29] Guyatt G, Oxman AD, Akl EA (2011). GRADE guidelines: 1. Introduction—GRADE evidence profiles and summary of findings tables. J Clin Epidemiol.

[30] Guyatt GH, Oxman AD, Vist GE (2008). GRADE: an emerging consensus on rating quality of evidence and strength of recommendations. BMJ.

[31] Rahimi K, Bidel Z, Nazarzadeh M (2021). Pharmacological blood pressure lowering for primary and secondary prevention of cardiovascular disease across different levels of blood pressure: an individual participant-level data meta-analysis. Lancet.

[32] Watt JA, Veroniki AA, Tricco AC (2021). Using a distribution-based approach and systematic review methods to derive minimum clinically important differences. BMC Med Res Methodol.

[33] Friedman H, Taub HA (1977). The use of hypnosis and biofeedback procedures for essential hypertension. Int J Clin Exp Hypn.

[34] Arslan G, Ceyhan Ö, Mollaoğlu M (2021). The influence of foot and back massage on blood pressure and sleep quality in females with essential hypertension: a randomized controlled study. J Hum Hypertens.

[35] Hager JL, Surwit RS (1978). Hypertension self-control with a portable feedback unit or meditation-relaxation. Biofeedback Self Regul.

[36] Manikonda JP, Störk S, Tögel S (2008). Contemplative meditation reduces ambulatory blood pressure and stress-induced hypertension: a randomized pilot trial. J Hum Hypertens.

[37] Park JE, Hong S, Lee M (2014). Randomized, controlled trial of qigong for treatment of prehypertension and mild essential hypertension. Altern Ther Health Med.

[38] Murugesan R, Govindarajulu N, Bera TK (2000). Effect of selected yogic practices on the management of hypertension. Indian J Physiol Pharmacol.

[39] Perez MI, Linden W, Perry T (2009). Failure of psychological interventions to lower blood pressure: a randomized controlled trial. Open Med.

[40] Loucks EB, Schuman-Olivier Z, Saadeh FB (2023). Effect of Adapted Mindfulness Training in Participants With Elevated Office Blood Pressure: The MB-BP Study: A Randomized Clinical Trial. J Am Heart Assoc.

[41] Palomba D, Ghisi M, Scozzari S (2011). Biofeedback-Assisted Cardiovascular Control in Hypertensives Exposed to Emotional Stress: A Pilot Study. *Appl Psychophysiol Biofeedback*.

[42] Ziv A, Vogel O, Keret D (2013). Comprehensive Approach to Lower Blood Pressure (CALM-BP): a randomized controlled trial of a multifactorial lifestyle intervention. J Hum Hypertens.

[43] Achmon J, Granek M, Golomb M (1989). Behavioral treatment of essential hypertension: a comparison between cognitive therapy and biofeedback of heart rate. Psychosom Med.

[44] Shapiro D, Hui KK, Oakley ME (1997). Reduction in drug requirements for hypertension by means of a cognitive-behavioral intervention. Am J Hypertens.

[45] Huijuan Y, Zhexin Y, Jing C (2021). Observation on the clinical effect of tcm tone-breathing exercise therapy on hyperactivity of liver-yang type of hypertension. Acta Medica Mediterranea.

[46] Teng XF, Wong MYM, Zhang YT (2007). The effect of music on hypertensive patients. *Annu Int Conf IEEE Eng Med Biol Soc*.

[47] Adams ZW, Sieverdes JC, Brunner-Jackson B (2018). Meditation smartphone application effects on prehypertensive adults’ blood pressure: Dose-response feasibility trial. Health Psychol.

[48] Ankolekar VH, Reddy G, Sanju, Chidananda SV (2019). Role of yoga intervention on quality of life and prehypertension. Indian Journal of Traditional Knowledge.

[49] Batey DM, Kaufmann PG, Raczynski JM (2000). Stress management intervention for primary prevention of hypertension: detailed results from Phase I of Trials of Hypertension Prevention (TOHP-I). Ann Epidemiol.

[50] Chandler J, Sox L, Diaz V (2020). Impact of 12-Month Smartphone Breathing Meditation Program upon Systolic Blood Pressure among Non-Medicated Stage 1 Hypertensive Adults. *IJERPH*.

[51] Chen S, Sun P, Wang S (2016). Effects of heart rate variability biofeedback on cardiovascular responses and autonomic sympathovagal modulation following stressor tasks in prehypertensives. J Hum Hypertens.

[52] Givi M, Sadeghi M, Garakyaraghi M (2018). Long-term effect of massage therapy on blood pressure in prehypertensive women. *J Edu Health Promot*.

[53] Hughes JW, Fresco DM, Myerscough R (2013). Randomized controlled trial of mindfulness-based stress reduction for prehypertension. Psychosom Med.

[54] Li X, Chang P, Wu M (2024). Effect of Tai Chi vs Aerobic Exercise on Blood Pressure in Patients With Prehypertension. JAMA Netw Open.

[55] Lin G, Xiang Q, Fu X (2012). Heart rate variability biofeedback decreases blood pressure in prehypertensive subjects by improving autonomic function and baroreflex. J Altern Complement Med.

[56] Mir IA, Chowdhury M, Islam RM (2021). Relaxing music reduces blood pressure and heart rate among pre‐hypertensive young adults: A randomized control trial. J Clin Hypertens.

[57] (2023). Effects of a Mindfulness Meditation on Blood Pressure in Prehypertension Patients: A Randomized Controlled Trial. *J Med Assoc Thai*.

[58] Schneider RH, Grim C, Kotchen T (2021). Randomized controlled trial of stress reduction with meditation and health education in black men and women with high normal and normal blood pressure. *Am J Prev Cardiol*.

[59] Singh VP, Khandelwal B (2022). Effectiveness Of Yoga and Lifestyle Modification On Prehypertensive Subjects-A Randomized Controlled Trial. Neuroquantology.

[60] Thiyagarajan R, Pal P, Pal GK (2015). Additional benefit of yoga to standard lifestyle modification on blood pressure in prehypertensive subjects: a randomized controlled study. *Hypertens Res*.

[61] Wang S-Z, Li S, Xu X-Y (2010). Effect of slow abdominal breathing combined with biofeedback on blood pressure and heart rate variability in prehypertension. J Altern Complement Med.

[62] Xu XY, Gao J, Ling D (2007). Biofeedback treatment of prehypertension: analyses of efficacy, heart rate variability and EEG approximate entropy. J Hum Hypertens.

[63] Dobrescu AI, Nussbaumer-Streit B, Klerings I (2021). Restricting evidence syntheses of interventions to English-language publications is a viable methodological shortcut for most medical topics: a systematic review. J Clin Epidemiol.

[64] Van Gordon W, Shonin E, Garcia-Campayo J (2017). Are there adverse effects associated with mindfulness?. *Aust N Z J Psychiatry*.

[65] Wayne PM, Berkowitz DL, Litrownik DE (2014). What do we really know about the safety of tai chi?: A systematic review of adverse event reports in randomized trials. Arch Phys Med Rehabil.

[66] Cramer H, Krucoff C, Dobos G (2013). Adverse events associated with yoga: a systematic review of published case reports and case series. PLoS One.

[67] Landman GWD, van Hateren KJJ, van Dijk PR (2014). Efficacy of Device-Guided Breathing for Hypertension in Blinded, Randomized, Active-Controlled Trials. JAMA Intern Med.

[68] Hippisley-Cox J, Coupland C, Vinogradova Y (2007). Derivation and validation of QRISK, a new cardiovascular disease risk score for the United Kingdom: prospective open cohort study. BMJ.

[69] McDonagh STJ, Reburn C, Smith J (2024). Group-delivered interventions for lowering blood pressure in hypertension: systematic review and meta-analysis. Br J Gen Pract.

[70] Dong Y, Zhang X, Zhao R (2024). The effects of mind-body exercise on anxiety and depression in older adults: a systematic review and network meta-analysis. Front Psychiatry.

[71] Sanogo F, Xu K, Cortessis VK (2023). Mind- and Body-Based Interventions Improve Glycemic Control in Patients with Type 2 Diabetes: A Systematic Review and Meta-Analysis. *J Integr Complement Med*.

[72] Jaeschke R, Singer J, Guyatt GH (1989). Measurement of health status. Ascertaining the minimal clinically important difference. Control Clin Trials.

[73] Staessen JA, Wang J-G, Thijs L (2001). Cardiovascular protection and blood pressure reduction: a meta-analysis. Lancet.

[74] Turnbull F (2003). Effects of different blood-pressure-lowering regimens on major cardiovascular events: results of prospectively-designed overviews of randomised trials. Lancet.

[75] NHS (2023). Health survey for England, 2021 part 2. https://digital.nhs.uk/data-and-information/publications/statistical/health-survey-for-england/2021-part-2/adult-health-hypertension.

[76] Ostchega Y, Fryar CD, Nwankwo T (2020). Hypertension Prevalence Among Adults Aged 18 and Over: United States, 2017-2018. NCHS Data Brief.

[77] Aggarwal R, Chiu N, Wadhera RK (2021). Racial/Ethnic Disparities in Hypertension Prevalence, Awareness, Treatment, and Control in the United States, 2013 to 2018. Hypertension.

[78] NHS Digital (2022). Health survey England additional analyses, ethnicity and health, 2011-2019 experimental statistics. https://digital.nhs.uk/data-and-information/publications/statistical/health-survey-england-additional-analyses/ethnicity-and-health-2011-2019-experimental-statistics/blood-pressure.

[79] Page MJ, McKenzie JE, Bossuyt PM (2021). The PRISMA 2020 statement: an updated guideline for reporting systematic reviews. BMJ.

[80] Aivazyan TA, Zaitsev VP, Yurenev AP (1988). Autogenic training in the treatment and secondary prevention of essential hypertension: five-year follow-up. Health Psychol.

[81] Chesney MA, Black GW, Swan GE (1987). Relaxation training for essential hypertension at the worksite: I. The untreated mild hypertensive. Psychosom Med.

[82] Agras WS, Taylor CB, Kraemer HC (1987). Relaxation training for essential hypertension at the worksite: II. The poorly controlled hypertensive. Psychosom Med.

