# Supplementary material for: Effectiveness of stress management and relaxation interventions for management of hypertension and prehypertension: systematic review and network meta-analysis
Source: BMJ Med. 2025 Apr 8;4(1):e001098. doi: 10.1136/bmjmed-2024-001098 (PMC12164322; doi:10.1136/bmjmed-2024-001098)
Supplement: online supplemental file 1 [file bmjmed-4-1-s001.pdf]

Supplementary materials

Supplementary methods 2

Search methods 2

Search strategies 2

Supplementary interventions 30

Grouping of interventions for synthesis 30

Data extraction and missing data 35

NMA model fitting and selection 36

Supplementary results 37

Excluded studies 37

Included studies 52

Model fit statistics 65

Relative effects of relaxation interventions compared to passive comparator 75

All relative effects from primary analyses 82

Hypertension: Short term follow-up (up to 3 months) 91

Disconnected studies 91

Network meta-analysis 91

Assessment of the certainty of the evidence 91

Hypertension: Medium term follow-up (>3 to 12 months) 94

Disconnected studies 94

Network meta-analysis 94

Assessment of the certainty of the evidence 95

Hypertension: Long term follow-up (>12 months) 98

Disconnected studies 99

Network meta-analysis 101

Hypertension: Sensitivity analyses 101

Including all studies, regardless of risk of bias 101

Excluding studies with mixed hypertensive and pre-hypertensive participants 102

Biofeedback reclassification 102

Changing pre-post correlation 103

Imputed data 103

Hypertension: Subgroup analyses 106

Antihypertensive medication status 106

Country-level economic resource 109

Age 110
Severity of hypertension 111
Pre-hypertension analyses 111
Sensitivity and subgroup analyses 114
Secondary outcomes 115
Economic outcomes 117
Risk of Bias 2 Assessments 119
GRADE Assessments 164
Equality, Diversity and Inclusion (EDI) perspectives 180
References 213

Supplementary methods

Search methods

To identify potentially eligible studies, we searched the following databases using relevant subject headings (controlled vocabularies), text-words and search syntax, appropriate to each resource.

- MEDLINE (Ovid) (1946 to 23 February 2024);
- PsycINFO (Ovid) (1806 to 23 February 2024);
- CINAHL (EBSCOhost) (Cumulative Index to Nursing and Allied Health Literature; 1982 to 27 February 2024);
- Cochrane Central Register of Controlled Trials (CENTRAL; 2024, Issue 2) in the Cochrane Library (searched 23 February 2024).

Records of randomized controlled trials from Embase were identified via CENTRAL (from a scoping exercise we did not anticipate finding many additional records unique to this database).

We also searched AMED (Ovid) (Allied and Complementary Medicine; 1985 to 10 October 2023). This database is produced by the Health Care Information Service at the British Library and as a consequence of a ransomware attack on our national library, this database was only current to October 2023 when we completed our searches in February 2024.

We did not apply any date restrictions to our search but limited it to reports published in English. We excluded pre-prints, conference abstracts, dissertations and theses and ongoing trial protocols.

To identify further published or unpublished research (beyond our main searches), we scanned the reference lists of included studies and relevant systematic reviews.

Search strategies

MEDLINE

Ovid MEDLINE(R) ALL <1946 to February 23, 2024>

1	exp Hypertension/	322615

2 (hypertensi* or prehypertensi*).tw,kf. 529031

3 antihypertensi*.ti,kf. 16821

4 (antihypertensi* adj3 (effect* or outcome?)).ab. 9278

5 ((elevated or high* or increased or borderline) adj3 (blood pressure or bloodpressure or BP)).tw,kf. 65507

6 ((elevated or high* or increased or borderline) adj3 ((arterial or diastolic or systolic) adj3 pressur*)).tw,kf.20188

7 ((reduc* or lower*) adj3 (blood pressure or bloodpressure or BP)).tw,kf. 49227

8 ((reduc* or lower*) adj3 ((arterial or diastolic or systolic) adj3 pressur*)).tw,kf. 14402

9 blood pressure.tw,kf,hw. and Cardiovascular Diseases/pc [Prevention & Control] 7439

10 or/1-9 654632

11 Relaxation/ or Muscle Relaxation/ or Relaxation Therapy/ 20786

12 relaxation.ti,kf. 30755

13 ((relaxation or relaxing) adj3 (activit* or app* or assist* or based or behavi* or breath* or cognitive or control* or counsel* or effect* or exercis* or hobby or hobbies or hypno* or imag* or intervention* or instruction* or lifestyl* or meditat* or mediat* or mental* or mind* or music* or muscle* or muscular or program* or progressi* or psychotherap* or psycholog* or respons* or self or stress or study or teach* or technique* or therap* or train* or treat* or trial or video* or virtual*)).tw,kf. 42455

14 (music* or acoustic stimulat* or song? or singing or choir?).tw,kf. 41530

15 (audio relaxation or sound therap* or healing sounds).tw,kf. 424

16 (physiological stress/ or exp chronic stress/ or exp mental stress/ or exp physically induced stress/) and therapy.fs. 1949

17 (stress* adj1 (manag* or recovery or prevent* or reduc*)).tw,kf. 24372

18 (stress adj3 coping).tw,kf. 8596

19 ((NPI or nonpharma* or non-pharma* or nondrug? or non-drug?) adj (intervention* or trial or therap*)).tw,kf. and (stress or relax*).mp. 1306

20 Breathing/ and therapy.fs. 4288

21 exp Breathing Exercise/ 4284

22 ((slow adj3 breathing) or ((nostril? or paced or pursed* or rhythmic* or yog*) adj3 breathing) or pranayama).tw,kf. 2111

23 (breath work* or breathwork* or holotropic breathing or buteyk* or pranayam*).tw,kf. 623

24 (breathing adj3 (activit* or app* or exercis* or intervention* or instruction* or meditat* or program* or progressi* or study or teach* or technique* or therap* or train* or trial)).tw,kf. 9316

25 (breathing control or guided breathing).tw,kf. 619

26 (forest bathing or shinrin yoku or shinrinyoku).tw,kf. 120

27 ((inspiratory or respiratory) adj3 (activit* or app* or device? or exercis* or intervention* or instruction* or meditat* or program* or progressi* or study or teach* or technique* or therap* or train* or trial)).tw,kf. 39276

28 ((breathing or inspiratory or respiratory) and (non-pharma* or nonpharma* or npi)).tw,kf. 1837

29 Mindfulness/ or Mindfulness meditation/ or Mindfulness-Based Cognitive Therapy/ or Mindfulness-Based Stress Reduction/ 6710

30 (mindfulnes* or mindfullnes* or (mind? adj3 (therap* or train*)) or mind-body or mindbody).tw,kf. 18308

31 (MBSR or MBCT).tw,kf. 1623

32 exp Meditation/ 3912

33 (meditat* or chanting).tw,kf. 8928

34 exp Mind-Body Therapies/ 48032

35 (kinesiotherap* or movement therap* or motion therap* or dance therap* or pilates or plyometrics or plyometric exercis* or static exercis* or stretching exercis* or qigong or qi gong or baduanjin or ba duan jin or chigung or chi gung or chikung or chi kung or tai chi or taichi or tai ji or taiji or taijiquan or yoga* or yogic).tw,kf. 15726

36 (hypnosis or hypnotism or hypnotherap* or hypno-therap* or hypnoanalysis or hypno-analysis or hypno* exercis* or mesmerism or autosuggestion or auto-suggestion or autogenic training).tw,kf. 10713

37 (guided imagery or (imagery and (therap* or psychotherap* or psychol*)) or reverie therap*).tw,kf. 3405

38 (biofeedback* or bio-feedback* or psycho* feedback* or myofeedback* or myo-feedback* or ((bogus or false) adj physiological adj (feedback* or feed-back*))).tw,kf. 8670

39 (neurofeedback* or neuro-feedback* or ((alpha or brainwave* or brain wave* or EEG or electroencephalo* or electro-encephalo* or electromyo* or electromyo*) adj feedback*)).tw,kf. 2573

40 (psychodrama or psycho* drama or drama therapy or role play* or laughter).tw,kf. 27181

41 massage/ 6882

42 (massag* or chihya or chih ya or shiatsu or shiatzu or tuina or tui na).tw,kf. 13613

43 (aromatherap* or aroma* therap* or essential oils).tw,kf. 14891

44 exp complementary therapies/ or (complementary therap* or alternative medicine*).tw,kf. 256843

45 (naturopath* or nature therap*).tw,kf. 1479

46 ((green adj (environment* or health* or infrastructur* or infra-structur* or space?)) or greenspace* or open space* or open air or outdoor? or countryside or rural environment* or natural environment or natural space? or wilderness or woods or (forest adj (environment* or setting*)) or parks or parkland* or park setting* or ((urban or town? or city or cities or innercit* or "access to") adj3 park) or garden* or horticultur*).tw,kf,hw. or forests.ti,kf,sh. 110313

47 ((blue adj (environment* or health* or infrastructure* or infra-structure* or space?)) or bluespace* or waterfront* or water-front* or waterway* or water-way* or waters or seaside* or sea-side* or beach or beaches or streams or canals or lakes or ponds or rivers or riparian or wetlands or wet-lands or marshes or running water? or fountains or ((outdoor* or out-door* or outside or out-side or open* or wild*) adj (pool? or bath* or water*))).tw,kf,hw. 204873

48 ((blue or water or aquatic or green or nature or land* or outdoor* or out-door*) adj (based or activit* or hobby or hobbies or leisure or recreation*)).tw,kf,hw. 19652

49 exp Balneology/12838

50 (balneo* or ammotherap* or ammo-therap* or ((mud or sand or steam or air) adj3 (bath* or therap*)) or sauna* or hotspring* or hot spring*).tw,kf. 8923

51 exp Hydrotherapy/ 21003

52 (hydrotherap* or hydro-therap* or whirlpool bath* or (shiatsu adj1 water) or watsu).tw,kf. 1658

53 ((water* adj3 immersi*) or baths or bathing).tw,kf. 24221

54 Hyperthermia, induced/19810

55 (((thermal* or hypertherm* or hypertherm*) adj (induc* or intervention* or therap*)) or waon).tw,kf. 9777

56 exp Cognitive Behavioral Therapy/ 37773

57 exp Psychotherapy, Group/ 27902

58 (CBT* or CBGT*).tw,kf. 16750

59 (cognitive behavi* or ((cogniti* or behavio*) adj3 (counsel* or intervention or management or psychotherap* or therap* or training or treatment or technique* or restructur* or defusion))).tw,kf. 116835

60 (rational emotive or (problem* adj2 (focus* or sol*)) or psychoeducat* or psycho-educat* or schema* or self-control* or self controlling).tw,kf. 104561

61 (((psychotherap* or therap*) adj3 (commitment or acceptance)) or ((self* or stress*) adj3 (control or analysis or direct* or esteem or help or instruct* or manage*))).tw,kf. 138138

62 (acceptance adj2 commitment adj2 (intervention or training or treatment or technique*)).tw,kf. 154

63 ((attribution* or reattribution*) adj3 (therap* or psychotherap*)).tw,kf. 61

64 ((anxiety adj2 manag*) or confidence building or coping skills or exposure therapy or exposure task? or sensitivity training or self talk).tw,kf. 9514

65 ((controlling or overcoming) adj2 (anxiety or panic or fear)).tw,kf. 720

66 ((thirdwave or third-wave) adj3 (cogniti* or behavi* or counsel* or intervention or psychotherap* or therap*)).tw,kf. 235

67 (compassion* adj3 (counsel* or intervention or psychotherap* or therap* or train*)).tw,kf. 930

68 (functional analy* adj3 (counsel* or intervention or psychotherap* or therap* or train*)).tw,kf. 170

69 ((metacogniti* or meta-cogniti*) adj3 (counsel* or intervention or psychotherap* or therap* or train*)).tw,kf. 706

70 (dialectic* adj3 (counsel* or intervention or psychotherap* or therap* or train*)).tw,kf. 1392

71 ((behavio* adj1 activat*) or BATD).ti,ab,kf. 3167

72 behavio*.mp. and (self adj (evaluat* or monitor*)).ti,ab,kf. 5088

73 ((gain? or reapprais*) adj2 focus*).ti,ab,kf. 226

74 (behavio* adj3 (contracting or modification or modify* or reinforce* or re-inforce*)).tw,kf. 13848

75 (positive affirmation* or ((positive or contingent) adj1 reinforc*) or (reinforc* adj3 (environment* or experience*))).tw,kf. 4083

76 (positive affect and self-affirmation).tw,kf. 20

77 ((activit* adj2 schedul*) or ((pleas* or enjoyable or rewarding) adj (activit* or event?))).tw,kf. 1598

78 (operant conditioning or instrumental learning or positive interaction* or avoidant coping or environmental contingenc* or contigency management).tw,kf. 7401

79 psychotherapy.mp. 94729

80 ((psychosocial* or psycho-social*) adj3 (activit* or app* or control* or counsel* or intervention* or program* or study or therap* or train* or treat* or trial)).tw,kf. 22602

81 Anger Management Therapy/ 69

82 (anger adj3 (activit* or app* or behavi* or control* or counsel* or intervention* or instruction* or lifestyl* or management or managing or meditat* or mediat* or program* or psycho* or study or teach* or technique* or therap* or train* or treat* or trial or video* or virtual*)).tw,kf. 3107

83 self-control/ or emotional regulation/ 6158

84 (emotion* adj3 regulat*).tw,kf. 19539

85 ((positiv* adj3 (emoti* or psych*)) or (reduc* adj3 negative adj3 (emoti* or psych*)) or (reduc* adj3 hostil*) or qi therap*).tw,kf. 23313

86 exp Animal Assisted Therapy/ 845

87 ((pet or pets or (animal adj (assisted or facilitated))) adj3 therap*).tw,kf. 2419

88 ((complex or factorial or multi component* or multicomponent* or multidimension* or multi dimension* or multifactor* or multi factor* or multifacet* or multi facet* or multilevel* or multi level* or multimodal* or multi modal* or multiparamet* or multi paramet* or multiecological or multi* ecological) adj (evidence or intervention* or trial)).tw,kf. 13306

89 or/11-88 1392217

90 10 and 89 18973

91 clinical trials as topic/ 201801

92 controlled clinical trial.pt. 95566

93 randomized controlled trial.pt. 609373

94 random allocation/ 107080

95 (randomi#ed or randomi#ation or randomi#ing).ti,ab,kf. 848337

96 (RCT or "at random" or (random* adj3 (administ* or allocat* or assign* or class* or cluster or crossover or cross-over or control* or determine* or divide* or division or distribut* or expose* or fashion or number* or place* or pragmatic or quasi or recruit* or split or substitut* or treat*))).tw,kf. 758138

97 trial.ti. 303631

98 (control* adj3 (arm or arms or group*)).ab. 703205

99 ((control* or compar* or versus) and (trial or study or group* or arm or arms) and (waitlist* or wait* list* or attention* control* or treatment-as-usual or TAU or care-as-usual or CAU or ((conventional or routine or standard or usual) adj2 (care or therap* or treatment or control?)) or untreated group* or untreated control* or no-therap* or non-therap* or nontherap* or minim* therap* or no-contact or pseudotherap* or sham or receiv* nothing or no-intervention or no-treatment* or non-treatment* or nontreatment* or (without adj2 (treatment or therap* or intervention)))).tw,kf,hw. 307487

100 or/91-99 2206895

101 exp animals/ not humans/ 5198554

102 100 not 101 1902592

103 90 and 102 4193

104 limit 103 to english language 3806

[S1 (6-Sept-2023) n=3745; S2 (10-Oct-2023) n=34; S3 (23-Feb-2024) n=85; Total n=3864]

PsycInfo

Ovid APA PsycInfo <1806 to February Week 4 2024>

1 exp Hypertension/ 8711

2 (hypertensi* or prehypertensi*).tw,id. 20990

3 antihypertensi*.ti,id. 463

4 (antihypertensi* adj3 (effect* or outcome?)).ab. 153

5 ((elevated or high* or increased or borderline) adj3 (blood pressure or bloodpressure or BP)).tw,id. 5031

6 ((elevated or high* or increased or borderline) adj3 ((arterial or diastolic or systolic) adj3 pressur*)).tw,id. 907

7 ((reduc* or lower*) adj3 (blood pressure or bloodpressure or BP)).tw,id. 2312

8 ((reduc* or lower*) adj3 ((arterial or diastolic or systolic) adj3 pressur*)).tw,id. 565

9 blood pressure.tw,id,hw. and exp Cardiovascular Disorders/ 6922

10 or/1-9 28031

11 relaxation/ or relaxation therapy/ or progressive relaxation therapy/ or muscle relaxation/ 7041

12 relaxation.ti,id. 6909

13 ((relaxation or relaxing) adj3 (activit* or app* or assist* or based or behavi* or breath* or cognitive or control* or counsel* or effect* or exercis* or hobby or hobbies or hypno* or imag* or intervention* or instruction* or lifestyl* or meditat* or mediat* or mental* or mind* or music* or muscle* or muscular or program* or progressi* or psychotherap* or psycholog* or respons* or self or stress or study or teach* or technique* or therap* or train* or treat* or trial or video* or virtual*)).tw,id. 12863

14 music therapy/ or singing/ 7298

15 (music* or acoustic stimulat* or song? or singing or choir?).tw,id. 54009

16 (audio relaxation or sound therap* or healing sounds).tw,id. 160

17 (((chronic or physiological or psychological) adj stress) and (therap* or train* or treat*)).mp. 6457

18 exp Stress Management/ 11944

19 (stress* adj1 (manag* or recovery or prevent* or reduc*)).tw,id. 16746

20 (stress adj3 coping).tw,id. 13496

21 ((NPI or nonpharma* or non-pharma* or nondrug? or non-drug?) adj (intervention* or trial or therap*)).tw,id. and (stress or relax*).mp. 392

22 Breathing Techniques/ 85

23 ((slow adj3 breathing) or ((nostril? or paced or pursed* or rhythmic* or yog*) adj3 breathing)).tw,id. 591

24 (breath work* or breathwork* or holotropic breathing or buteyk* or pranayam*).tw,id. 285

25 (breathing adj3 (activit* or app* or exercis* or intervention* or instruction* or meditat* or program* or progressi* or study or teach* or technique* or therap* or train* or trial)).tw,id. 1853

26 (breathing control or guided breathing).tw,id. 77

27 breath*.ti. and (therap* or treat* or train*).mp. 1023

28 (forest bathing or shinrin yoku or shinrinyoku).tw,id. 19

29 ((inspiratory or respiratory) adj3 (activit* or app* or device? or exercis* or intervention* or instruction* or meditat* or program* or progressi* or study or teach* or technique* or therap* or train* or trial)).tw,id. 1964

30 ((breathing or inspiratory or respiratory) and (non-pharma* or nonpharma* or npi)).tw,id. 177

31 mindfulness/ or mindfulness-based interventions/ or mindfulness meditation/ or mindfulness-based cognitive therapy/ or mindfulness-based stress reduction/ 15783

32 (mindfulnes* or mindfullnes* or (mind? adj3 (therap* or train*)) or mind-body or mindbody).tw,id. 26889

33 (MBSR or MBCT).tw,id. 1822

34 Mind Body Therapy/ 400

35 exp Meditation/ 5906

36 (meditat* or chanting).tw,id. 12012

37 (kinesiotherap* or movement therap* or motion therap* or dance therap* or pilates or plyometrics or plyometric exercis* or static exercis* or stretching exercis* or qigong or qi gong or baduanjin or ba duan jin or chigung or chi gung or chikung or chi kung or tai chi or taichi or tai ji or taiji or taijiquan or yoga* or yogic).tw,id,hw. 8042

38 exp Hypnotherapy/ 5245

39 (hypnosis or hypnotism or hypnotherap* or hypno-therap* or hypnoanalysis or hypno-analysis or hypno* exercis* or mesmerism or autosuggestion or auto-suggestion or autogenic training).tw,id,hw. 19413

40 exp psychotherapeutic techniques/ 34485

41 (visuali#ation? or guided imagery or (imagery and (therap* or psychotherap* or psychol*)) or reverie therap*).tw,id. 16047

42 (psychodrama or psycho* drama or drama therapy or role play* or laughter).tw,id. 20578

43 exp Biofeedback/ 6945

44 (biofeedback* or bio-feedback* or psycho* feedback* or myofeedback* or myo-feedback* or ((bogus or false) adj physiological adj (feedback* or feed-back*))).tw,id. 6269

45 (neurofeedback* or neuro-feedback* or ((alpha or brainwave* or brain wave* or EEG or electroencephalo* or electro-encephalo* or electromyo* or electromyo*) adj feedback*)).tw,id. 2309

46 exp massage/ 599

47 (massag* or chihya or chih ya or shiatsu or shiatzu or tuina or tui na).tw,id. 1938

48 (aromatherap* or aroma* therap* or essential oils).tw,id,hw. 465

49 exp Holistic Health/ 2157

50 (complementary therap* or alternative medicine*).tw,id,hw. 7567

51 (naturopath* or nature therap*).tw,id. 233

52 ((green adj (environment* or health* or infrastructur* or infra-structur* or space?)) or greenspace* or open space* or open air or outdoor? or countryside or rural environment* or natural environment or natural space? or wilderness or woods or (forest adj (environment* or setting*)) or parks or parkland* or park setting* or ((urban or town? or city or cities or innercit* or "access to") adj3 park) or garden* or horticultur*).tw,id,hw. or forests.ti,id. 44701

53 ((blue adj (environment* or health* or infrastructure* or infra-structure* or space?)) or bluespace* or waterfront* or water-front* or waterway* or water-way* or waters or seaside* or sea-side* or beach or beaches or streams or canals or lakes or ponds or rivers or riparian or wetlands or wet-lands or marshes or running water? or fountains or ((outdoor* or out-door* or outside or out-side or open* or wild*) adj (pool? or bath* or water*))).tw,id,hw. 13684

54 ((blue or water or aquatic or green or nature or land* or outdoor* or out-door*) adj (based or activit* or hobby or hobbies or leisure or recreation*)).tw,id,hw. 2598

55 (balneo* or ammotherap* or ammo-therap* or ((mud or sand or steam or air) adj3 (bath* or therap*)) or sauna* or hotspring* or hot spring*).tw,id. 340

56 (hydrotherap* or hydro-therap* or whirlpool bath* or (shiatsu adj1 water) or watsu).tw,id,hw. 183

57 ((water* adj3 immersi*) or baths or bathing).tw,id. 1703

58 (((thermal* or hypertherm* or hyper-therm*) adj (induc* or intervention* or therap*)) or waon).tw,id. 207

59 exp Cognitive Behavior Therapy/ 55676

60 exp Group Psychotherapy/ 24777

61 (CBT* or CBGT*).tw,id. 18880

62 (cognitive behavi* or ((cogniti* or behavio*) adj3 (counsel* or intervention or management or psychotherap* or therap* or training or treatment or technique* or restructur* or defusion))).tw,id,hw. 149029

63 (rational emotive or (problem* adj2 (focus* or sol*)) or psychoeducat* or psycho-educat* or schema* or self-control* or self controlling).tw,id,hw. 134253

64 (((psychotherap* or therap*) adj3 (commitment or acceptance)) or ((self* or stress*) adj3 (control or analysis or direct* or esteem or help or instruct* or manage*))).tw,id,hw. 148257

65 (acceptance adj2 commitment adj2 (intervention or training or treatment or technique*)).tw,id. 274

66 ((attribution* or reattribution*) adj3 (therap* or psychotherap*)).tw,id. 210

67 ((anxiety adj2 manag*) or confidence building or coping skills or exposure therapy or exposure task? or sensitivity training or self talk).tw,id,hw. 18202

68 ((controlling or overcoming) adj2 (anxiety or panic or fear)).tw,id. 889

69 ((thirdwave or third-wave) adj3 (cogniti* or behavi* or counsel* or intervention or psychotherap* or therap*)).tw,id. 440

70 (compassion* adj3 (counsel* or intervention or psychotherap* or therap* or train*)).tw,id,hw. 1363

71 (functional analy* adj3 (counsel* or intervention or psychotherap* or therap* or train*)).tw,id. 451

72 ((metacogniti* or meta-cogniti*) adj3 (counsel* or intervention or psychotherap* or therap* or train*)).tw,id,hw. 1286

73 (dialectic* adj3 (counsel* or intervention or psychotherap* or therap* or train*)).tw,id,hw. 3197

74 ((behavio* adj1 activat*) or BATD).tw,id,hw. 3111

75 behavio*.tw,id,hw. and (self adj (evaluat* or monitor*)).tw,id. 6818

76 ((gain? or reapprais*) adj2 focus*).tw,id. 173

77 (behavio* adj3 (contracting or modification or modify* or reinforce* or re-inforce*)).tw,id,hw. 25585

78 (positive affirmation* or ((positive or contingent) adj1 reinforc*) or (reinforc* adj3 (environment* or experience*))).tw,id,hw. 6813

79 (positive affect and self-affirmation).tw,id. 19

80 ((activit* adj2 schedul*) or ((pleas* or enjoyable or rewarding) adj (activit* or event?))).tw,id. 1892

81 (operant conditioning or instrumental learning or positive interaction* or avoidant coping or environmental contingenc* or contigency management).tw,id,hw. 15920

82 psychotherapy.tw,id,hw. 151313

83 ((psychosocial* or psycho-social*) adj3 (activit* or app* or control* or counsel* or intervention* or program* or study or therap* or train* or treat* or trial)).tw,id. 21445

84 Anger Control/ 1193

85 (anger adj3 (activit* or app* or behavi* or control* or counsel* or intervention* or instruction* or lifestyl* or management or managing or meditat* or mediat* or program* or psycho* or study or teach* or technique* or therap* or train* or treat* or trial or video* or virtual*)).tw,id. 7147

86 self-control/ or emotional regulation/ 27187

87 (emotion* adj3 regulat*).tw,id. 28310

88 ((positiv* adj3 (emoti* or psych*)) or (reduc* adj3 negative adj3 (emoti* or psych*)) or (reduc* adj3 hostil*) or qi therap*).tw,id. 36946

89 exp Animal Assisted Therapy/ 1492

90 ((pet or pets or (animal adj (assisted or facilitated))) adj3 therap*).tw,id. 1036

91 ((complex or factorial or multi component* or multicomponent* or multidimension* or multi dimension* or multifactor* or multi factor* or multifacet* or multi facet* or multilevel* or multi level* or multimodal* or multi modal* or multiparamet* or multi paramet* or multiecological or multi* ecological) adj (evidence or intervention* or trial)).tw,id. 4222

92 or/11-91 849750

93 10 and 92 3986

94 clinical trials.sh. 12305

95 treatment effectiveness evaluation.sh. 29212

96 (empirical study and treatment outcome).md. 24147

97 (randomi#ed or randomi#ation or randomi#ing).tw,id. 113374

98 (RCT or "at random" or (random* adj3 (administ* or allocat* or assign* or class* or cluster or crossover or cross-over or control* or determine* or divide* or division or distribut* or expose* or fashion or number* or place* or pragmatic or quasi or recruit* or split or substitut* or treat*))).tw,id. 132861

99 trial.ti. 39664

100 (control* adj3 (arm or arms or group*)).ab. 117556

101 ((control* or compar* or versus) and (trial or study or group* or arm or arms) and (waitlist* or wait* list* or attention* control* or treatment-as-usual or TAU or care-as-usual or CAU or ((conventional or routine or standard or usual) adj2 (care or therap* or treatment or control?)) or untreated group* or untreated control* or no-therap* or non-therap* or nontherap* or minim* therap* or no-contact or pseudotherap* or sham or receiv* nothing or no-intervention or no-treatment* or non-treatment* or nontreatment* or (without adj2 (treatment or therap* or intervention)))).tw,id,hw. 48829

102 or/94-101 313351

103 93 and 102 847

104 ((animal model* or mouse or mice or murine* or rat or rats or rodent* or muridae or murids or rabbit* or leporine* or leporidae or guineapig* or cavies or caviidae or hamster* or cricetidae or gerbil* or gerbillinae or cat or cats or feline* or felidae or dog or dogs or canine* or canidae or pig or pigs or piglet* or minipig* or swine* or porcine* or suidae or horse or horses or donkey or donkies or burros or asses or equine* or equidae or sheep or lamb or lambs or ovine or ovidae or goat or goats or cow or cows or cattle or bovine* or bovidae or primate* or monkey or monkeys or macaque or macaques or marmoset or marmosets) not human*).ti. 180938

105 103 not 104 833

106 limit 105 to english language 797

[S1 (10-Oct-2023) n=783; S2 (27-Feb-2024) n=14 (new records)]

CINAHL

EBSCOHost CINAHL <1982 to 27 February 2024>

Searched: 27-Feb-2024

[S1 (23-Oct-2023) n=1160; S2 (27-Feb-2024) n= 44 (new records)]

Query Results

S96 S17 AND S94 1,188 [Limiters - Exclude MEDLINE Journals]

S95 S17 AND S94 3,131

S94 S18 OR S19 OR S20 OR S21 OR S22 OR S23 OR S24 OR S25 OR S26 OR S27 OR S28 OR S29 OR S30 OR S31 OR S32 OR S33 OR S34 OR S35 OR S36 OR S37 OR S38 OR S39 OR S40 OR S41 OR S42 OR S43 OR S44 OR S45 OR S46 OR S47 OR S48 OR S49 OR S50 OR S51 OR S52 OR S53 OR S54 OR S55 OR S56 OR S57 OR S58 OR S59 OR S60 OR S61 OR S62 OR S63 OR S64 OR S65 OR S66 OR S67 OR S68 OR S69 OR S70 OR S71 OR S72 OR S73 OR S74 OR S75 OR S76 OR S77 OR S78 OR S79 OR S80 OR S81 OR S82 OR S83 OR S84 OR S85 OR S86 OR S87 OR S88 OR S89 OR S90 OR S91 OR S92 OR S93 875,344

S93 TI (((complex or factorial or “multi component*” or multicomponent* or multidimension* or “multi dimension*” or multifactor* or “multi factor*” or multifacet* or “multi facet*” or multilevel* or “multi level*” or multimodal* or “multi modal*” or multiparamet* or “multi paramet*” or multiecological or “multi* ecological”) N1 (evidence or intervention* or trial))) OR AB (((complex or factorial or “multi component*” or multicomponent* or multidimension* or “multi dimension*” or multifactor* or “multi factor*” or multifacet* or “multi facet*” or multilevel* or “multi level*” or multimodal* or “multi modal*” or multiparamet* or “multi paramet*” or multiecological or “multi* ecological”) N1 (evidence or intervention* or trial))) 9,704

S92 TI (((pet or pets or "animal assisted" or "animal facilitated") N3 therap*)) OR AB (((pet or pets or "animal assisted" or "animal facilitated") N3 therap*)) 1,245

S91 (MH "Pet Therapy+") 1,881

S90 TI (((reduc* N3 hostil*) or "qi therap*")) OR AB (((reduc* N3 hostil*) or "qi therap*")) 117

S89 TI ((reduc* N3 negative N3 (emoti* or psych*))) OR AB ((reduc* N3 negative N3 (emoti* or psych*))) 459

S88 TI (((positiv* N3 (emoti* or psych*))) OR AB (((positiv* N3 (emoti* or psych*))) 11,756

S87 TI (emotion* N3 regulat*) OR AB (emotion* N3 regulat*) OR MW (emotion* N3 regulat*) 8,572

S86 TI ((anger N3 (activit* or app* or behavi* or control* or counsel* or intervention* or instruction* or lifestyl* or management or managing or meditat* or mediat* or program* or psycho* or study or teach* or technique* or therap* or train* or treat* or trial or video* or virtual*))) OR AB ((anger N3 (activit* or app* or behavi* or control* or counsel* or intervention* or instruction* or lifestyl* or management or managing or meditat* or mediat* or program* or psycho* or study or teach* or technique* or therap* or train* or treat* or trial or video* or virtual*))) 2,034

S85 TI (((psychosocial* or psycho-social*) N3 (activit* or app* or control* or counsel* or intervention* or program* or study or therap* or train* or treat* or trial))) OR AB (((psychosocial* or psycho-social*) N3 (activit* or app* or control* or counsel* or intervention* or program* or study or therap* or train* or treat* or trial))) OR MW (((psychosocial* or psycho-social*) N3 (activit* or app* or control* or counsel* or intervention* or program* or study or therap* or train* or treat* or trial))) 27,880

S84 TI psychotherapy OR AB psychotherapy OR MW psychotherapy 39,160

S83 TI ("operant conditioning" or "instrumental learning" or "positive interaction*" or "avoidant coping" or "environmental contingenc*" or "contigency management") OR AB ("operant conditioning" or "instrumental learning" or "positive interaction*" or "avoidant coping" or "environmental contingenc*" or "contigency management") 1,596

S82 TI ((activit* N2 schedul*) or ((pleas* or enjoyable or rewarding) W1 (activit* or event or events))) OR AB ((activit* N2 schedul*) or ((pleas* or enjoyable or rewarding) W1 (activit* or event or events))) 915

S81 TI ("positive affect" and self*) OR AB ("positive affect" and self*) 1,240

S80 TI ("positive affirmation*" or "positive reinforc*" or "contingent reinforc*" or (reinforc* N3 (environment* or experience*))) OR AB ("positive affirmation*" or "positive reinforc*" or "contingent reinforc*" or (reinforc* N3 (environment* or experience*))) 1,212

S79 TI ((behavio* N3 (contracting or modification or modify* or reinforce* or re-inforce*))) OR AB ((behavio* N3 (contracting or modification or modify* or reinforce* or re-inforce*))) OR MW ((behavio* N3 (contracting or modification or modify* or reinforce* or re-inforce*))) 6,937

S78 TI (((gain or gains or reapprais*) N2 focus*)) OR AB (((gain or gains or reapprais*) N2 focus*)) 181

S77 TI (behavio* and ("self evaluat*" or "self monitor*")) OR AB (behavio* and ("self evaluat*" or "self monitor*")) OR MW (behavio* and ("self evaluat*" or "self monitor*")) 2,162

S76 TI ((behavio* W1 activat*) or BATD) OR AB ((behavio* W1 activat*) or BATD) 1,015

S75 TI ((dialectic* N3 (counsel* or intervention or psychotherap* or therap* or train*))) OR AB ((dialectic* N3 (counsel* or intervention or psychotherap* or therap* or train*))) OR MW ((dialectic* N3 (counsel* or intervention or psychotherap* or therap* or train*))) 884

S74 TI ((metacogniti* or meta-cogniti*) N3 (counsel* or intervention or psychotherap* or therap* or train*)) OR AB ((metacogniti* or meta-cogniti*) N3 (counsel* or intervention or psychotherap* or therap* or train*)) 453

S73 TI (("functional analy*" AND (counsel* or intervention or psychotherap* or therap* or train*))) OR AB (("functional analy*" AND (counsel* or intervention or psychotherap* or therap* or train*))) 712

S72 TI ((compassion* N3 (counsel* or intervention or psychotherap* or therap* or train*))) OR AB ((compassion* N3 (counsel* or intervention or psychotherap* or therap* or train*))) 876

S71 TI ((thirdwave or third-wave) N3 (cogniti* or behavi* or counsel* or intervention or psychotherap* or therap*)) OR AB ((thirdwave or third-wave) N3 (cogniti* or behavi* or counsel* or

intervention or psychotherap* or therap*)) OR MW ((thirdwave or third-wave) N3 (cogniti* or behavi* or counsel* or intervention or psychotherap* or therap*)) 149

S70 TI ((controlling or overcoming) N2 (anxiety or panic or fear)) OR AB ((controlling or overcoming) N2 (anxiety or panic or fear)) 600

S69 TI ((anxiety N2 manag*) or "confidence building" or "coping skills" or "exposure therapy" or "exposure task*" or "sensitivity training" or "self talk") OR AB ((anxiety N2 manag*) or "confidence building" or "coping skills" or "exposure therapy" or "exposure task*" or "sensitivity training" or "self talk") OR MW ((anxiety N2 manag*) or "confidence building" or "coping skills" or "exposure therapy" or "exposure task*" or "sensitivity training" or "self talk") 5,670

S68 TI ((attribution* or reattribution*) N3 (therap* or psychotherap*)) OR AB ((attribution* or reattribution*) N3 (therap* or psychotherap*)) 42

S67 TI ((acceptance N2 commitment) AND (intervention or training or treatment or technique*)) OR AB ((acceptance N2 commitment) AND (intervention or training or treatment or technique*)) OR MW ((acceptance N2 commitment) AND (intervention or training or treatment or technique*)) 1,049

S66 TI (((self* or stress*) N3 (control or analysis or direct* or esteem or help or instruct* or manage*))) OR AB (((self* or stress*) N3 (control or analysis or direct* or esteem or help or instruct* or manage*))) OR MW (((self* or stress*) N3 (control or analysis or direct* or esteem or help or instruct* or manage*))) 91,849

S65 TI ((psychotherap* or therap*) N3 (commitment or acceptance)) OR AB ((psychotherap* or therap*) N3 (commitment or acceptance)) OR MW ((psychotherap* or therap*) N3 (commitment or acceptance)) 2,074

S64 TI ("rational emotive" or (problem* N2 (focus* or sol*)) or psychoeducat* or psycho-educat* or schema* or self-control* or "self controlling") OR AB ("rational emotive" or (problem* N2 (focus* or sol*)) or psychoeducat* or psycho-educat* or schema* or self-control* or "self controlling") OR MW ("rational emotive" or (problem* N2 (focus* or sol*)) or psychoeducat* or psycho-educat* or schema* or self-control* or "self controlling") 45,341

S63 TI ("cognitive behavi*" or ((cogniti* or behavio*) N3 (counsel* or intervention or management or psychotherap* or therap* or training or treatment or technique* or restructur* or defusion))) OR AB ("cognitive behavi*" or ((cogniti* or behavio*) N3 (counsel* or intervention or management or psychotherap* or therap* or training or treatment or technique* or restructur* or defusion))) OR MW ("cognitive behavi*" or ((cogniti* or behavio*) N3 (counsel* or intervention or management or psychotherap* or therap* or training or treatment or technique* or restructur* or defusion))) 88,331

S62 TI (CBT* or CBGT*) OR AB (CBT* or CBGT*) 7,229

S61 (MH "Psychotherapy, Group+") 25,925

S60 (MH "Cognitive Therapy+") 30,642

S59 TI ((((thermal* or hypertherm* or hyper-therm*) N1 (induc* or intervention* or therap*)) or waon)) OR AB ((((thermal* or hypertherm* or hyper-therm*) N1 (induc* or intervention* or therap*)) or waon)) 1,163

S58 (MH "Hyperthermia, Induced+") 5,048

S57 TI ((water* N3 immersi*) or baths or bathing) OR AB ((water* N3 immersi*) or baths or bathing) OR MW ((water* N3 immersi*) or baths or bathing) 9,188

S56 TI ((hydrotherap* or hydro-therap* or "whirlpool bath*" or (shiatsu W1 water) or watsu) OR AB ((hydrotherap* or hydro-therap* or "whirlpool bath*" or (shiatsu W1 water) or watsu) 594

S55 (MH "Hydrotherapy+") 7,230

S54 TI (balneo* or ammotherap* or ammo-therap* or ((mud or sand or steam or air) N3 (bath* or therap*)) or sauna* or hotspring* or "hot spring*") OR AB (balneo* or ammotherap* or ammo-therap* or ((mud or sand or steam or air) N3 (bath* or therap*)) or sauna* or hotspring* or "hot spring*") OR MW (balneo* or ammotherap* or ammo-therap* or ((mud or sand or steam or air) N3 (bath* or therap*)) or sauna* or hotspring* or "hot spring*") 1,443

S53 (MH "Balneology") OR (MH "Bathing and Baths") 3,800

S52 TI (((blue or water or aquatic or green or nature or land* or outdoor* or out-door*) W1 (based or activit* or hobby or hobbies or leisure or recreation*))) OR AB (((blue or water or aquatic or green or nature or land* or outdoor* or out-door*) W1 (based or activit* or hobby or hobbies or leisure or recreation*))) 3,083

S51 ((blue W1 (environment* or health* or infrastructure* or infra-structure* or space*)) or bluespace* or waterfront* or water-front* or waterway* or water-way* or waters or seaside* or sea-side* or beach or beaches or streams or canals or lakes or ponds or rivers or riparian or wetlands or wet-lands or marshes or “running water*” or fountains or ((outdoor* or out-door* or outside or out-side or open* or wild*) W1 (pool or pools or bath* or water*))) 107,222

S50 ((green W1 (environment* or health* or infrastructur* or infra-structur* or space*)) or greenspace* or "open space*" or "open air" or outdoor* or countryside or "rural environment*" or "natural environment" or "natural space*" or wilderness or woods or (forest W1 (environment* or setting*)) or parks or parkland* or "park setting*" or ((urban or town or towns or city or cities or innercit* or "access to") N3 park) or garden* or horticultur*) 101,118

S49 TI (naturopath* or "nature therap*") OR AB (naturopath* or "nature therap*") 1,040

S48 TI ("complementary therap*" or "alternative medicine*") OR AB ("complementary therap*" or "alternative medicine*") 11,121

S47 (MH "Alternative Therapies+") 269,733

S46 TI (aromatherap* or (aroma* N1 therap*) or "essential oils") OR AB (aromatherap* or (aroma* N1 therap*) or "essential oils") OR MW (aromatherap* or (aroma* N1 therap*) or "essential oils") 8,265

S45 TI (massag* or chihya or "chih ya" or shiatsu or shiatzu or tuina or "tui na") OR AB (massag* or chihya or "chih ya" or shiatsu or shiatzu or tuina or "tui na") OR MW (massag* or chihya or "chih ya" or shiatsu or shiatzu or tuina or "tui na") 22,451

S44 TI (psychodrama or (psycho* N1 drama) or "drama therapy" or "role play*" or laughter) OR AB (psychodrama or (psycho* N1 drama) or "drama therapy" or "role play*" or laughter) OR MW (psychodrama or (psycho* N1 drama) or "drama therapy" or "role play*" or laughter) 8,238

S43 TI (((alpha or brainwave* or brain wave* or EEG or electroencephalo* or electro-encephalo* or electromyo* or electromyo*) N1 feedback*)).) OR AB (((alpha or brainwave* or brain wave* or EEG or electroencephalo* or electro-encephalo* or electromyo* or electromyo*) N1 feedback*))) 88

S42 TI (neurofeedback* or neuro-feedback*) OR AB (neurofeedback* or neuro-feedback*) OR MW (neurofeedback* or neuro-feedback*) 715

S41 TI ((bogus or false) AND physiological AND (feedback* or feed-back*)) OR AB ((bogus or false) AND physiological AND (feedback* or feed-back*)) 10

S40 TI (biofeedback* or bio-feedback* or (psycho* N1 feedback*) or myofeedback* or myo-feedback*) OR AB (biofeedback* or bio-feedback* or (psycho* N1 feedback*) or myofeedback* or myo-feedback*) OR MW (biofeedback* or bio-feedback* or (psycho* N1 feedback*) or myofeedback* or myo-feedback*) 5,305

S39 TI (("guided imagery" or (imagery and (therap* or psychotherap* or psychol*)) or "reverie therap*")) OR AB (("guided imagery" or (imagery and (therap* or psychotherap* or psychol*)) or "reverie therap*")) OR MW (("guided imagery" or (imagery and (therap* or psychotherap* or psychol*)) or "reverie therap*")) 3,945

S38 (MH "Distraction") 2,044

S37 TI ((hypnosis or hypnotism or hypnotherap* or hypno-therap* or hypnoanalysis or hypno-analysis or "hypno* exercis*" or mesmerism or autosuggestion or auto-suggestion or "autogenic training")) OR AB ((hypnosis or hypnotism or hypnotherap* or hypno-therap* or hypnoanalysis or hypno-analysis or "hypno* exercis*" or mesmerism or autosuggestion or auto-suggestion or "autogenic training")) OR MW ((hypnosis or hypnotism or hypnotherap* or hypno-therap* or hypnoanalysis or hypno-analysis or "hypno* exercis*" or mesmerism or autosuggestion or auto-suggestion or "autogenic training")) 4,094

S36 TI ((kinesiotherap* or "movement therap*" or "motion therap*" or "dance therap*" or pilates or plyometric* or "static exercis*" or "stretching exercis*" or qigong or "qi gong" or baduanjin or "ba duan jin" or chigung or "chi gung" or chikung or "chi kung" or "tai chi" or taichi or "tai ji" or taiji or taijiquan or yoga* or yogic)) OR AB ((kinesiotherap* or "movement therap*" or "motion therap*" or "dance therap*" or pilates or plyometric* or "static exercis*" or "stretching exercis*" or qigong or "qi gong" or baduanjin or "ba duan jin" or chigung or "chi gung" or chikung or "chi kung" or "tai chi" or taichi or "tai ji" or taiji or taijiquan or yoga* or yogic)) OR MW ((kinesiotherap* or "movement therap*" or "motion therap*" or "dance therap*" or pilates or plyometric* or "static exercis*" or "stretching exercis*" or qigong or "qi gong" or baduanjin or "ba duan jin" or chigung or "chi gung" or chikung or "chi kung" or "tai chi" or taichi or "tai ji" or taiji or taijiquan or yoga* or yogic)) 19,730

S35 TI ((meditat* or chanting)) OR AB ((meditat* or chanting)) OR MW ((meditat* or chanting)) 7,862

S34 TI ((mindfulnes* or mindfullnes* or MBSR or MBCT or (mind* N3 (therap* or train*)) or mind-body or mindbody)) OR AB ((mindfulnes* or mindfullnes* or MBSR or MBCT or (mind* N3 (therap* or train*)) or mind-body or mindbody)) OR ((mindfulnes* or mindfullnes* or MBSR or MBCT or (mind* N3 (therap* or train*)) or mind-body or mindbody)) 16,911

S33 TI (((breathing or inspiratory or respiratory) and (non-pharma* or nonpharma* or npi))) OR AB (((breathing or inspiratory or respiratory) and (non-pharma* or nonpharma* or npi))) 589

S32 ((inspiratory or respiratory) N3 (activit* or app* or device or devices or exercis* or intervention* or instruction* or meditat* or program* or progressi* or study or teach* or technique* or therap* or train* or trial)) 33,659

S31 TI ("forest bathing" or "shinrin yoku" or shinrinyoku) OR AB ("forest bathing" or "shinrin yoku" or shinrinyoku) OR MW ("forest bathing" or "shinrin yoku" or shinrinyoku) 34

S30 TI breath* AND (therap* or treat* or train*) 4,215

S29 TI ("breathing control" or "guided breathing") OR AB ("breathing control" or "guided breathing") OR MW ("breathing control" or "guided breathing") 150

S28 TI ((breathing N3 (activit* or app* or exercis* or intervention* or instruction* or meditat* or program* or progressi* or study or teach* or technique* or therap* or train* or trial))) OR AB ((breathing N3 (activit* or app* or exercis* or intervention* or instruction* or meditat* or program* or progressi* or study or teach* or technique* or therap* or train* or trial))) OR MW ((breathing N3 (activit* or app* or exercis* or intervention* or instruction* or meditat* or program* or progressi* or study or teach* or technique* or therap* or train* or trial))) 6,242

S27 TI (("breath work*" or breathwork* or "holotropic breathing" or buteyk* or pranayam*)) OR AB (("breath work*" or breathwork* or "holotropic breathing" or buteyk* or pranayam*)) OR MW (("breath work*" or breathwork* or "holotropic breathing" or buteyk* or pranayam*)) 277

S26 TI (((nostril* or paced or pursed* or rhythmic* or yog*) N3 breathing))) OR AB (((nostril* or paced or pursed* or rhythmic* or yog*) N3 breathing))) 441

S25 TI (slow N3 breathing) OR AB (slow N3 breathing) 190

S24 (NPI or nonpharma* or non-pharma* or nondrug* or non-drug*) AND (intervention* or trial or therap*) AND (stress or relax*) 1,385

S23 TI (stress N3 coping) OR AB (stress N3 coping) OR MW (stress N3 coping) 5,797

S22 TI ((stress* N1 (manag* or recovery or prevent* or reduc*))) OR AB ((stress* N1 (manag* or recovery or prevent* or reduc*))) OR MW ((stress* N1 (manag* or recovery or prevent* or reduc*))) 31,602

S21 TI (("chronic stress" or "physiological stress" or "psychological stress") and (therap* or train* or treat*)) OR AB (("chronic stress" or "physiological stress" or "psychological stress") and (therap* or train* or treat*)) 1,533

S20 TI ("acoustic stimulat*" or "sound therap*" or "healing sounds") OR AB ("acoustic stimulat*" or "sound therap*" or "healing sounds") OR MW ("acoustic stimulat*" or "sound therap*" or "healing sounds") 5,319

S19 TI ((music* or song or songs or singing or choir*)) OR AB ((music* or song or songs or singing or choir*)) OR MW ((music* or song or songs or singing or choir*)) 25,159

S18 TI (relaxation or relaxing) OR AB (relaxation or relaxing) OR MW (relaxation or relaxing) 22,022

S17 S7 AND S16 17,864

S16 S8 OR S9 OR S10 OR S11 OR S12 OR S13 OR S14 OR S15 150,453

S15 TI (((reduc* or lower*) N3 ((arterial or diastolic or systolic) N3 pressur*))) OR AB (((reduc* or lower*) N3 ((arterial or diastolic or systolic) N3 pressur*))) 4,133

S14 TI (((reduc* or lower*) N3 ("blood pressure" or bloodpressure or BP))) OR AB (((reduc* or lower*) N3 ("blood pressure" or bloodpressure or BP))) 12,700

S13 TI (((elevated or high* or increased or borderline) N3 ((arterial or diastolic or systolic) N3 pressur*))) OR AB (((elevated or high* or increased or borderline) N3 ((arterial or diastolic or systolic) N3 pressur*))) 5,801

S12 TI (((elevated or high* or increased or borderline) N3 ("blood pressure" or bloodpressure or BP))) OR AB (((elevated or high* or increased or borderline) N3 ("blood pressure" or bloodpressure or BP))) 17,134

S11 TI antihypertensi*. OR AB ((antihypertensi* N3 (effect* or outcome*))) 4,405

S10 TI ((hypertensi* or prehypertensi*)) OR AB ((hypertensi* or prehypertensi*)) 112,986

S9 (MH "Prehypertension") 439

S8 (MH "Essential Hypertension") OR (MH "Hypertension") 66,922

S7 S1 OR S2 OR S3 OR S4 OR S5 OR S6 526,312

S6 (MH "Random Assignment") 83,806

S5 (MH "Randomized Controlled Trials") 142,192

S4 TI (trial) 190,061

S3 TI ((randomly n3 (administ* or allocat* or assign* or class* or cluster or crossover or cross-over or control* or determine* or divide* or division or distribut* or expose* or fashion or number* or place* or pragmatic or quasi or recruit* or split or substitut* or treat*)))) OR AB ((random* n3 (administ* or allocat* or assign* or class* or cluster or crossover or cross-over or control* or determine* or divide* or division or distribut* or expose* or fashion or number* or place* or pragmatic or quasi or recruit* or split or substitut* or treat*)))) 231,227

S2 TI ((random n3 (administ* or allocat* or assign* or class* or cluster or crossover or cross-over or control* or determine* or divide* or division or distribut* or expose* or fashion or number* or place* or pragmatic or quasi or recruit* or split or substitut* or treat*))) OR AB ((random n3 (administ* or allocat* or assign* or class* or cluster or crossover or cross-over or control* or determine* or divide* or division or distribut* or expose* or fashion or number* or place* or pragmatic or quasi or recruit* or split or substitut* or treat*))) 10,678

S1 TI (randomiz* OR randomis* OR “at random” OR RCT OR cRCT or “controlled clinical trial”) OR AB (randomiz* OR randomis* OR “at random” OR RCT OR cRCT or “controlled clinical trial”) 388,640

CENTRAL

Cochrane Central Register of Controlled Trials (CENTRAL) in the Cochrane Library;
Issue 2 of 12, searched 27-Feb-2024

#1 MeSH descriptor: [Hypertension] explode all trees 25226

#2 (hypertensi* or prehypertensi* or pre-hypertensi* or (pre NEXT hypertensi*)):ti,ab,kw 78526

#3 (antihypertensi* or anti-hypertensi* or (anti NEXT hypertensi*)):ti 4184

#4 ((antihypertensi* or anti-hypertensi* or (anti NEXT hypertensi*)) NEAR (effect* or outcome*)):ab,kw 6298

#5 ((elevated or high* or increased or borderline) NEAR/3 ("blood pressure" or bloodpressure or BP)):ti,ab,kw 10499

#6 ((elevated or high* or increased or borderline) NEAR/3 ((arterial or diastolic or systolic) NEAR pressur*)):ti,ab,kw 3797

#7 ((reduc* or lower*) NEAR/3 ("blood pressure" or bloodpressure or BP)):ti,ab,kw 18528

#8 ((reduc* or lower*) NEAR/3 ((arterial or diastolic or systolic) NEAR pressur*)):ti,ab,kw 7234

#9 MeSH descriptor: [Cardiovascular Diseases] this term only and with qualifier(s): [prevention & control - PC] 5369

#10 ("blood pressure" or bloodpressure):ti,ab,kw 114690

#11 #9 AND #10 1768

#12 #1 OR #2 OR #3 OR #4 OR #5 OR #6 OR #7 OR #8 OR #10 157545

#13 (2023* OR 2024*) 157736

#14 **#12 AND #13** 10964

#15 relaxation:ti,kw 6877

#16 ((relaxation or relaxing) NEAR/3 (activit* or app* or assist* or based or behavi* or breath* or cognitive or control* or counsel* or effect* or exercis* or hobby or hobbies or hypno* or imag* or intervention* or instruction* or lifestyl* or meditat* or mediat* or mental* or mind* or music* or muscle* or muscular or program* or progressi* or psychotherap* or psycholog* or respons* or self or stress or study or teach* or technique* or therap* or train* or treat* or trial or video* or virtual*)):ti,ab,kw 12291

#17 (music* or (acoustic NEXT stimulat*) or song or songs or singing or choir or choirs):ti,ab,kw 9443

#18 ("audio relaxation" or (sound NEXT therap*) or "healing sounds"):ti,ab,kw 175

#19 MeSH descriptor: [Stress, Physiological] this term only 1700

#20 MeSH descriptor: [Stress, Psychological] explode all trees and with qualifier(s): [therapy - TH] 1980

#21 (stress* NEAR/2 (manag* or recovery or prevent* or reduc*)):ti,ab,kw 12446

#22 (stress NEAR/3 coping):ti,ab,kw 1237

#23 ((NPI or nonpharma* or non-pharma* or (non NEXT pharma*) or nondrug* or non-drug* or (non NEXT drug*)) NEAR/2 (intervention* or trial or therap*)):ti,ab,kw and (stress or relax*):ti,ab,kw 526

#24 MeSH descriptor: [Breathing Exercises] explode all trees 1331

#25 ((slow NEAR/3 breathing) or ((nostril* or paced or pursed* or rhythmic* or yog*) NEAR/3 breathing)):ti,ab,kw 1140

#26 ((breath NEXT work*) or breathwork* or "holotropic breathing" or buteyk* or pranayam*):ti,ab,kw 855

#27 (breathing NEAR/3 (activit* or app* or exercis* or intervention* or instruction* or meditat* or program* or progressi* or study or teach* or technique* or therap* or train* or trial)):ti,ab,kw 9492

#28 ("breathing control" or "guided breathing"):ti,ab,kw 239

#29 ("forest bathing" or "shinrin yoku" or shinrinyoku):ti,ab,kw 37

#30 (inspiratory NEAR/3 (activit* or app* or device or devices or exercis* or intervention* or instruction* or meditat* or program* or progressi* or study or teach* or technique* or therap* or train* or trial)):ti,ab,kw 2084

#31 (respiratory NEAR/3 (activit* or app* or device or devices or exercis* or intervention* or instruction* or meditat* or program* or progressi* or study or teach* or technique* or therap* or train* or trial)):ti,ab,kw 17974

#32 ((breathing or inspiratory or respiratory) and (NPI or nonpharma* or non-pharma* or (non NEXT pharma*))):ti,ab,kw 728

#33 MeSH descriptor: [Mindfulness] explode all trees 2227

#34 (mindfulnes* or mindfullnes* or (mind NEAR/3 (therap* or train*)) or (minds NEAR/3 (therap* or train*)) or mind-body or mindbody or (mind NEXT body)):ti,ab,kw 10639

#35 (MBSR or MBCT):ti,ab 1809

#36 MeSH descriptor: [Meditation] explode all trees 1071

#37 (meditat* or chanting):ti,ab,kw 4832

#38 MeSH descriptor: [Mind-Body Therapies] explode all trees 9261

#39 (kinesiotherap* or (movement NEXT therap*) or (motion NEXT therap*) or (dance NEXT therap*) or pilates or plyometrics or (plyometric NEXT exercis*) or (static NEXT exercis*) or (stretching NEXT exercis*) or qigong or "qi gong" or baduanjin or "ba duan jin" or chigung or "chi gung" or chikung or "chi kung" or "tai chi" or taichi or "tai ji" or taiji or taijiquan or yoga* or yogic):ti,ab,kw 18489

#40 (hypnosis or hypnotism or hypnotherap* or hypno-therap* or (hypno* NEXT therap*) or hypnoanalysis or hypno-analysis or (hypno* NEXT analysis) or (hypno* NEXT exercis*) or mesmerism or autosuggestion or auto-suggestion or (auto NEXT suggesti*) or "autogenic training"):ti,ab,kw 2485

#41 ("guided imagery" or (imagery and (therap* or psychotherap* or psychol*)) or (reverie NEXT therap*)):ti,ab,kw 2590

#42 (biofeedback* or bio-feedback* or (bio NEXT feedback*) or (psycho* NEXT feedback*) or myofeedback* or myo-feedback* or (myo NEXT feedback*)):ti,ab,kw 4503

#43 ((bogus or false) NEXT physiological NEXT feed*):ti,ab,kw 4

#44 (neurofeedback* or neuro-feedback* or (neuro NEXT feedback*)):ti,ab,kw 1273

#45 ((alpha or brainwave* or (brain NEXT wave*) or EEG or electroencephalo* or electro-encephalo* or (electro NEXT encephalo*) or electromyo* or electro-myo* or (electro NEXT myo*)) NEXT feedback*):ti,ab,kw 161

#46 (psychodrama or drama or (role NEXT play*) or laughter or happiness):ti,ab,kw 3843

#47 MeSH descriptor: [Massage] explode all trees 1654

#48 (massag* or chihya or "chih ya" or shiatsu or shiatzu or tuina or "tui na"):ti,ab,kw
7983

#49 (aromatherap* or (aroma* NEXT therap*) or "essential oils"):ti,ab,kw 1967

#50 MeSH descriptor: [Complementary Therapies] this term only 629

#51 MeSH descriptor: [Spiritual Therapies] explode all trees 2360

#52 ((complementary NEXT therap*) or (alternative NEXT medicine*)):ti,ab,kw 3871

#53 (naturopath* or (nature NEXT therap*)):ti,ab,kw245

#54 ((green NEXT (environment* or health* or infrastructur* or infra-structur* or space or spaces)) or greenspace* or "open space" or "open spaces" or "open air" or outdoor or outdoors or countryside or (rural NEXT environment*) or (natural NEXT environment) or "natural space" or "natural spaces"):ti,ab,kw 2459

#55 (wilderness* or woods or (forest NEXT (environment* or setting*)) or garden* or horticultur*):ti,ab,kw 992

#56 (parks or parkland* or (park NEXT setting*) or ((urban or town or towns or city or cities or innercit* or "access to") NEAR/3 park)):ti,ab,kw 373

#57 (blue NEXT (environment* or health* or infrastructure* or infra-structure* or space or spaces)):ti,ab,kw 9

#58 (bluespace* or waterfront* or water-front* or waterway* or water-way* or waters or seaside* or sea-side* or (sea NEXT side*) or beach or beaches or streams or canals or lakes or ponds or rivers or riparian or wetlands or wet-lands or marshes or (running NEXT water*) or fountains):ti,ab,kw 3455

#59 ((outdoor* or out-door* or outside or out-side or open* or wild*) NEXT (pool or pools or bath* or water*)):ti,ab,kw 33

#60 ((blue or water or aquatic or green or nature or land or outdoor* or out-door*) NEXT (based or activit* or hobby or hobbies or leisure or recreation*)):ti,ab,kw 1246

#61 MeSH descriptor: [Balneology] explode all trees 773

#62 (balneo* or ammotherap* or ammo-therap* or (ammo NEXT therap*) or ((mud or sand or steam or air) NEAR/3 (bath* or therap*)) or sauna* or hotspring* or (hot NEXT spring*)):ti,ab,kw 1066

#63 MeSH descriptor: [Hydrotherapy] explode all trees 1933

#64 (hydrotherap* or hydro-therap* or (hydro NEXT therap*) or whirlpool or (shiatsu NEAR/2 water) or watsu):ti,ab,kw 774

#65 ((water* NEAR/3 immersi*) or baths or bathing):ti,ab,kw2672

#66 MeSH descriptor: [Hyperthermia, Induced] this term only 826

#67 (((thermal* or hypertherm* or hyper-therm* or (hyper NEXT therm*)) NEAR/2 (induc* or intervention* or therap*)) or waon):ti,ab,kw 1404

#68 MeSH descriptor: [Cognitive Behavioral Therapy] explode all trees 13835

#69 MeSH descriptor: [Psychotherapy, Group] explode all trees 4267

#70 (CBT* or CBGT*):ti,ab 11603

#71 ((cognitive NEXT behavi*) or (cogniti* NEAR/3 (counsel* or intervention or management or psychotherap* or therap* or training or treatment or technique* or restructur* or defusion)) or (behavio* NEAR/3 (counsel* or intervention or management or psychotherap* or therap* or training or treatment or technique* or restructur* or defusion))):ti,ab,kw 68426

#72 ("rational emotive" or (problem* NEAR/2 (focus* or sol*)) or psychoeducat* or psycho-educat* or (psycho* NEXT educat*) or schema* or self-control* or (self NEXT control) or "self controlling"):ti,ab,kw 18476

#73 ((psychotherap* or therap*) NEAR/3 (commitment or acceptance)):ti,ab,kw 2152

#74 ((self* or stress*) NEAR/3 (control or analysis or direct* or esteem or help or instruct* or manage*)):ti,ab,kw 37576

#75 (acceptance NEAR/2 commitment NEAR/2 (intervention or training or treatment or technique*)):ti,ab,kw 358

#76 ((attribution* or reattribution*) NEAR/3 (therap* or psychotherap*)):ti,ab,kw 35

#77 ((anxiety NEAR/2 manag*) or "confidence building" or "building confidence" or "coping skills" or "exposure therapy" or (exposure NEXT task*) or "sensitivity training" or "self talk"):ti,ab,kw 4719

#78 ((controlling or overcoming) NEAR/2 (anxiety or panic or fear)):ti,ab,kw 104

#79 ((thirdwave or third-wave or (third NEXT wave)) NEAR (cogniti* or behavi* or counsel* or intervention or psychotherap* or therap*)):ti,ab,kw 105

#80 (compassion* NEAR (counsel* or intervention or psychotherap* or therap* or train*)):ti,ab,kw789

#81 (functional NEAR analy* NEAR (counsel* or intervention or psychotherap* or therap* or train*)):ti,ab,kw513

#82 (metacogniti* or meta-cogniti* or (meta NEXT cogniti*)):ti,ab,kw 1122

#83 (dialectic* NEAR (counsel* or intervention or psychotherap* or therap* or train)):ti,ab,kw 627

#84 ((behavio* NEAR/2 activat*) or BATD):ti,ab,kw 1462

#85 (behavio* and (self NEXT (evaluat* or monitor*))):ti,ab,kw 3134

#86 ((gain or gains or reapprais*) NEAR/2 focus*):ti,ab,kw 48

#87 (behavio* NEAR/3 (contracting or modification or modify* or reinforce* or re-inforce*)):ti,ab,kw 2694

#88 ((positive NEXT affirmation*) or ((positive or contingent) NEAR/2 reinforc*) or (reinforc* NEAR/3 (environment* or experience*))):ti,ab,kw 633

#89 ("positive affect" and self):ti,ab,kw 669

#90 ((activit* NEAR/2 schedul*) or ((pleas* or enjoyable or rewarding) NEXT (activit* or event or events))):ti,ab,kw 665

#91 ("operant conditioning" or "instrumental learning" or (positive NEXT interaction*) or "avoidant coping" or (environmental NEXT contingenc*) or "contigency management"):ti,ab,kw 502

#92 psychotherapy:ti,ab,kw 15969

#93 ((psychosocial* or psycho-social* or (psycho NEXT social*)) NEAR (activit* or app* or control* or counsel* or intervention* or program* or study or therap* or train* or treat* or trial)):ti,ab,kw 11661

#94 MeSH descriptor: [Anger Management Therapy] this term only 29

#95 (anger NEAR (activit* or app* or behavi* or control* or counsel* or intervention* or instruction* or lifestyl* or management or managing or meditat* or mediat* or program* or psycho* or study or teach* or technique* or therap* or train* or treat* or trial or video* or virtual*)):ti,ab,kw 1593

#96 MeSH descriptor: [Self-Control] explode all trees699

#97 (emotion* NEAR/3 regulat*):ti,ab,kw 4029

#98 ((positiv* NEAR/3 (emoti* or psych*)) or (reduc* NEAR negative NEAR (emoti* or psych*)) or (reduc* aNEAR/3 hostil*) or (qi NEXT therap*)):ti,ab,kw 4271

#99 MeSH descriptor: [Animal Assisted Therapy] explode all trees 136

#100 ((pet or pets or "animal assisted" or "animal facilitated") NEAR/3 therap*):ti,ab,kw 418

#101 ((factorial or (multi NEXT component*) or multicomponent* or multidimension* or (multi NEXT dimension*) or multifactor* or (multi NEXT factor*) or multifacet* or (multi NEXT facet*) or multilevel* or (multi NEXT level*) or multimodal* or (multi NEXT modal*) or multiparamet* or (multi NEXT paramet*) or multiecological or (multi* NEXT ecological)) NEXT (evidence or intervention* or trial)):ti,ab,kw 4268

#102 "complex intervention":ti,ab,kw 1027

#103 #15 OR #16 OR #17 OR #18 OR #19 OR #20 OR #21 OR #22 OR #23 OR #24 OR #25 OR #26 OR #27 OR #28 OR #29 OR #30 OR #31 OR #32 61732

#104 #14 AND #103 727

#105 #33 OR #34 OR #35 OR #36 OR #37 OR #38 OR #39 OR #40 OR #41 OR #42 OR #43 OR #44 OR #45 OR #46 OR #47 OR #48 OR #49 OR #50 OR #51 OR #52 56289

#106 #14 AND #105 413

#107 #53 OR #54 OR #55 OR #56 OR #57 OR #58 OR #59 OR #60 OR #61 OR #62 OR #63 OR #64 OR #65 OR #66 OR #67 14995

#108 #14 AND #107 104

#109 #68 OR #69 OR #70 OR #71 OR #72 OR #73 OR #74 OR #75 OR #76 OR #77 OR #78 OR #79 OR #80 OR #81 OR #82 OR #83 OR #84 OR #85 OR #86 OR #87 OR #88 OR #89 OR #90 OR #91 OR #92 OR #93 132476

#110 #14 AND #109 688

#111 #94 OR #95 OR #96 OR #97 OR #98 OR #99 OR #100 OR #101 OR #102 15442

#112 #14 AND #111 111

#113 (#104 OR #106 OR #108 OR #110 OR #112) 1641

CLib:CENTRAL (Trials) n=1624

Date-of-entry into CENTRAL (Trials) 25-Oct-2023 to 27-Feb-2024, n=522

[Duplicates (6); Trial Registry Records (162); Conference Abstracts (48)]

Records retained (28 Feb 2024) n=306;
Previous search (25-Oct-2023) records retained n=4448

AMED

Ovid AMED (Allied and Complementary Medicine Database) <1985 to 10 October 2023>

[AMED was only up-to-date as of Oct 2023, due to a ransomware attack at the British Library]

1 exp Hypertension/ 1265

2 (hypertensi* or prehypertensi*).mp. 2336

3 antihypertensi*.mp. 540

4 blood pressure/ 991

5 ((elevated or high* or increased or borderline) adj3 (blood pressure or bloodpressure or BP)).ti,ab,et. 359

6 ((elevated or high* or increased or borderline) adj3 ((arterial or diastolic or systolic) adj3 pressur*)).ti,ab,et. 72

7 ((reduc* or lower*) adj3 (blood pressure or bloodpressure or BP)).ti,ab,et. 516

8 ((reduc* or lower*) adj3 ((arterial or diastolic or systolic) adj3 pressur*)).ti,ab,et. 173

9 blood pressure.mp. and exp cardiovascular disease/ 1075

10 or/1-9 3686

11 exp Relaxation/ or Muscle Relaxation/ 1356

12 relax*.mp. 3570

13 music/ or music therapy/ or sound therapy/ 1320

14 (music* or acoustic stimulat* or song? or singing or choir?).mp. 1884

15 (audio relaxation or sound therap* or healing sounds).mp. 70

16 (((chronic or physiological or psychological) adj stress) and (therap* or train* or treat*)).mp. 169

17 (stress* adj1 (manag* or recovery or prevent* or reduc*)).mp. 1039

18 (stress adj3 coping).mp. 260

19 (NPI or nonpharma* or non-pharma* or nondrug? or non-drug?).mp. 934

20 breath*.ti. and (therap* or treat* or train*).mp. 394

21 exp breathing exercises/ or breathing therapies/ 546

22 ((slow adj3 breathing) or ((nostril? or paced or pursed* or rhythmic* or yog*) adj3 breathing)).mp. 128

23 (breath work* or breathwork* or holotropic breathing or buteyk* or pranayam*).mp. 108

24 (breathing adj3 (activit* or app* or exercis* or intervention* or instruction* or meditat* or program* or progressi* or study or teach* or technique* or therap* or train* or trial)).mp. 958

25 (breathing control or guided breathing).mp. 16

26 (forest bathing or shinrin yoku or shinrinyoku).mp. 4

27 ((inspiratory or respiratory) adj3 (activit* or app* or device? or exercis* or intervention* or instruction* or meditat* or program* or progressi* or study or teach* or technique* or therap* or train* or trial)).mp. 1151

28 (mindfulnes* or mindfullnes* or (mind? adj3 (therap* or train*)) or mind-body or mindbody).mp. 1663

29 (MBSR or MBCT).mp. 81

30 exp Meditation/ 508

31 (meditat* or chanting).mp. 926

32 exp spiritual therapies/ 1922

33 (kinesiotherap* or movement therap* or motion therap* or dance therap* or pilates or plyometrics or plyometric exercis* or static exercis* or stretching exercis* or qigong or qi gong or baduanjin or ba duan jin or chigung or chi gung or chikung or chi kung or tai chi or taichi or tai ji or taiji or taijiquan or yoga* or yogic).mp. 3846

34 exp psychosomatic therapies/ 7229

35 exp hypnosis/ 3665

36 (hypnosis or hypnotism or hypnotherap* or hypno-therap* or hypnoanalysis or hypno-analysis or hypno* exercis* or mesmerism or autosuggestion or auto-suggestion or autogenic training).mp. 3881

37 visualization/ 189

38 (visuali#ation? or guided imagery or (imagery and (therap* or psychotherap* or psychol*)) or reverie therap*).mp. 1026

39 biofeedback/ 1323

40 (biofeedback* or bio-feedback* or psycho* feedback* or myofeedback* or myo-feedback* or ((bogus or false) adj physiological adj (feedback* or feed-back*))).mp. 1624

41 (neurofeedback* or neuro-feedback* or ((alpha or brainwave* or brain wave* or EEG or electroencephalo* or electro-encephalo* or electromyo* or electromyo*) adj feedback*)).mp. 73

42 (psychodrama or psycho* drama or drama therapy or role play* or laughter).mp. 620

43 massage/ 2961

44 (massag* or chihya or chih ya or shiatsu or shiatzu or tuina or tui na).mp. 4037

45 (aromatherap* or aroma* therap* or essential oils).mp. 1488

46 exp complementary therapies/ or (complementary therap* or alternative medicine*).mp. 69596

47 (naturopath* or nature therap*).mp. 1419

48 ((green adj (environment* or health* or infrastructur* or infra-structur* or space?)) or greenspace* or open space* or open air or outdoor? or countryside or rural environment* or natural environment or natural space? or wilderness or woods or (forest adj (environment* or setting*)) or parks or parkland* or park setting* or ((urban or town? or city or cities or innercit* or "access to") adj3 park) or garden* or horticultur* or forests).mp. 1171

49 ((blue adj (environment* or health* or infrastructure* or infra-structure* or space?)) or bluespace* or waterfront* or water-front* or waterway* or water-way* or waters or seaside* or sea-side* or beach or beaches or streams or canals or lakes or ponds or rivers or riparian or wetlands or wet-lands or marshes or running water? or fountains or ((outdoor* or out-door* or outside or out-side or open* or wild*) adj (pool? or bath* or water*))).mp. 281

50 ((blue or water or aquatic or green or nature or land* or outdoor* or out-door*) adj (based or activit* or hobby or hobbies or leisure or recreation*)).mp. 268

51 balneotherapy/ or exp steam bath/ 79

52 (balneo* or ammotherap* or ammo-therap* or ((mud or sand or steam or air) adj3 (bath* or therap*)) or sauna* or hotspring* or hot spring*).mp. 287

53 exp Hydrotherapy/ 843

54 (hydrotherap* or hydro-therap* or whirlpool bath* or (shiatsu adj1 water) or watsu).mp.846

55 ((water* adj3 immersi*) or baths or bathing).mp. 638

56 Hyperthermia, induced/130

57 (((thermal* or hypertherm* or hypertherm*) adj (induc* or intervention* or therap*)) or waon).mp. 168

58 exp Psychotherapy/ 10609

59 (CBT* or CBGT*).mp. 370

60 (cognitive behavi* or ((cogniti* or behavio*) adj3 (counsel* or intervention or management or psychotherap* or therap* or training or treatment or technique* or restructur* or defusion))).mp. 5624

61 (rational emotive or (problem* adj2 (focus* or sol*)) or psychoeducat* or psycho-educat* or schema* or self-control* or self controlling).mp. 2120

62 (psychotherap* or (therap* adj3 (commitment or acceptance)) or ((self* or stress*) adj3 (control or analysis or direct* or esteem or help or instruct* or manage*))).mp. 7445

63 (acceptance adj2 commitment).mp. 43

64 (attribution* or reattribution*).mp. 307

65 ((anxiety adj2 manag*) or confidence building or coping skills or exposure therapy or exposure task? or sensitivity training or self talk).mp. 390

66 ((controlling or overcoming) adj2 (anxiety or panic or fear)).mp. 17

67 (thirdwave or third-wave).mp. 11

68 (compassion* adj3 (counsel* or intervention or psychotherap* or therap* or train*)).mp. 35

69 functional analy*.mp. 170

70 ((metacogniti* or meta-cogniti*) adj3 (counsel* or intervention or psychotherap* or therap* or train*)).mp. 30

71 (dialectic* adj3 (counsel* or intervention or psychotherap* or therap* or train*)).mp. 24

72 ((behavio* adj1 activat*) or BATD).mp. 47

73 (behavio* and (self adj (evaluat* or monitor*))).mp. 89

74 ((gain? or reapprais*) adj2 focus*).mp. 4

75 (behavio* adj3 (contracting or modification or modify* or reinforce* or re-inforce*)).mp. 258

76 (positive affirmation* or ((positive or contingent) adj1 reinforc*) or (reinforc* adj3 (environment* or experience*))).mp. 86

77 (positive affect and self-affirmation).mp. 2

78 ((activit* adj2 schedul*) or ((pleas* or enjoyable or rewarding) adj (activit* or event?))).mp. 68

79 (operant conditioning or instrumental learning or positive interaction* or avoidant coping or environmental contingenc* or contigency management).mp. 104

80 (psychosocial* or psycho-social*).mp. 4496

81 Anger/ 157

82 (anger adj3 (activit* or app* or behavi* or control* or counsel* or intervention* or instruction* or lifestyl* or management or managing or meditat* or mediat* or program* or psycho* or study or teach* or technique* or therap* or train* or treat* or trial or video* or virtual*)).mp.127

83 (emotion* adj3 regulat*).mp. 155

84 ((positiv* adj3 (emoti* or psych*)) or (reduc* adj3 negative adj3 (emoti* or psych*)) or (reduc* adj3 hostil*) or qi therap*).mp. 604

85 exp Animal Assisted Therapy/ 122

86 ((pet or pets or (animal adj (assisted or facilitated))) adj3 therap*).mp. 167

87 ((complex or factorial or multi component* or multicomponent* or multidimension* or multi dimension* or multifactor* or multi factor* or multifacet* or multi facet* or multilevel* or multi level* or multimodal* or multi modal* or multiparamet* or multi paramet* or multiecological or multi* ecological) adj (evidence or intervention* or trial)).mp. 269

88 or/11-87 97599

89 10 and 88 1490

90 randomized controlled trials/ 3211

91 random allocation/ 335

92 (randomi#ed or randomi#ation or randomi#ing).mp. 18749

93 (RCT or "at random" or (random* adj3 (administ* or allocat* or assign* or class* or cluster or crossover or cross-over or control* or determine* or divide* or division or distribut* or expose* or fashion or number* or place* or pragmatic or quasi or recruit* or split or substitut* or treat*))).mp. 21557

94 trial.ti. 7655

95 (control* adj3 (arm or arms or group*)).ab. 13702

96 ((control* or compar* or versus) and (trial or study or group* or arm or arms) and (waitlist* or wait* list* or attention* control* or treatment-as-usual or TAU or care-as-usual or CAU or ((conventional or routine or standard or usual) adj2 (care or therap* or treatment or control?)) or untreated group* or untreated control* or no-therap* or non-therap* or nontherap* or minim* therap* or no-contact or pseudotherap* or sham or receiv* nothing or no-intervention or no-treatment* or non-treatment* or nontreatment* or (without adj2 (treatment or therap* or intervention)))).mp. 32975

97 or/90-96 47772

98 97 and 89 474

99 ((animal model* or mouse or mice or murine* or rat or rats or rodent* or muridae or murids or rabbit* or leporine* or leporidae or guineapig* or cavies or caviidae or hamster* or cricetidae or gerbil* or gerbillinae or cat or cats or feline* or felidae or dog or dogs or canine* or canidae or pig or pigs or piglet* or minipig* or swine* or porcine* or suidae or horse or horses or donkey or donkies or burros or asses or equine* or equidae or sheep or lamb or lambs or ovine or ovidae or goat or

goats or cow or cows or cattle or bovine* or bovidae or primate* or monkey or monkeys or macaque or macaques or marmoset or marmosets) not human*).ti. 11104

100 98 not 99 400

101 limit 100 to english 392

Supplementary interventions

In addition to the primary interventions of interest and comparators, several additional comparators were eligible. These formed a 'supplementary set' of interventions to complement the decision set, and comprised:

- Self-monitoring of blood pressure;
- Antihypertensive medication (classes of such interventions include ACE inhibitors, angiotensin-II receptor blockers, beta blockers, calcium channel blockers, centrally acting alpha agonists and diuretics; any pharmacological treatment not classified as an antihypertensive will be excluded);
- Physical activity and exercise strategies of moderate to vigorous intensity;
- Specific dietary interventions, such as the Dietary Approaches to Stop Hypertension (DASH) diet or a Mediterranean diet;
- General lifestyle advice, including recommendations for weight loss, smoking cessation and/or dietary modification e.g. salt reduction.

These interventions were used to enhance the connectivity of the network and provide further indirect evidence on the comparisons of interest. However, the relative efficacy of interventions in the supplementary set was not the primary focus of this review.

Grouping of interventions for synthesis

In order to conduct a meaningful analysis, interventions were grouped.

Our review protocol included a number of pre-specified interventions and comparators to be included. The stress management and relaxation interventions (decision set) were:

- Accessing outdoor green or blue space e.g. 'forest bathing' or horticultural therapy
- Animal-assisted therapies
- Autogenic training
- Biofeedback
- Breathing control and guided breathing
- Massage therapy
- Meditation and mindfulness-based strategies e.g. mindfulness-based stress reduction
- Meditative movement-based strategies e.g. Qigong, Tai chi, Yoga
- Music therapy
- Progressive muscle relaxation

- Psychotherapeutic and counselling strategies, e.g. cognitive behavioural therapy (CBT).

These categories were used to inform the grouping of interventions for the final review. We attempted to group together interventions that used similar techniques.

None of the identified eligible trials investigated accessing outdoor green or blue spaces or animal-assisted therapies. As the descriptions of meditation and mindfulness-based therapies were quite distinct in the included studies, this category was separated into two groups for analysis. In addition, although hypnosis was not explicitly listed in the protocol, the descriptions of this intervention in the included studies were distinct from autogenic training, therefore, it was considered a separate category.

Of the studies that used biofeedback, some described the specific relaxation techniques that were used to encourage relaxation during a biofeedback session. However, other studies did not describe the specific techniques used, or used a combination of relaxation techniques. Consequently, for the primary analysis we grouped together all studies that reported using biofeedback interventions. However, we conducted a sensitivity analysis to assess the impact of categorising these interventions according to the relaxation method used.

Finally, a number of studies used combinations of multiple relaxation methods that did not align with any of the individual categories above. These were grouped into a 'multicomponent intervention' category.

The controls of interest (comparator set) pre-specified in the review protocol included:

- No intervention
- Sham control
- Waiting list
- Standard care.

Classification of specific comparators described in the primary studies was guided by decision frameworks regarding the choice of comparators in clinical trials (1, 2). The above controls were grouped into two broader categories – 'passive' comparators (including no intervention, waiting list and standard care) and 'nonspecific' comparators (sham control), for reasons outlined below.

When categorising comparator arms, it was often difficult to distinguish between studies that offered 'no intervention', 'standard care' or 'wait-list control'. Many studies employing a wait-list control group also offered standard care before participants received the active intervention. Furthermore, it was not clear that studies where participants received 'no intervention' actually offered no care to participants. Clinical guidelines and quality standards for the management of hypertension have been in use for decades (3), and most of the included studies recruited participants from primary or secondary care settings. We therefore considered it unlikely that standard care would be prohibited or withheld from control arms described as 'no intervention' or 'waitlist'. Finally, standard care can differ between settings and will evolve over time. It is often insufficiently defined or reported in the primary studies, and (depending on the trial) it may involve no treatment at all or multiple treatments, such as continued medication and lifestyle advice (2, 4). In the context of the current review, we therefore considered it appropriate to combine no intervention, waiting list, and standard care as passive comparators.

Sham control conditions included any form of intervention that was not expected to have an effect on blood pressure or simply controlled for the contact time or participant engagement level. This included interventions such as sham biofeedback, intermittent office blood pressure checks, or attention controls (for example, where participants attended educational classes about topics other than hypertension).

A supplementary set of interventions was pre-specified in the review protocol to complement the network of evidence. The following interventions were therefore included if they were compared to any of the decision interventions:

- Self-monitoring of blood pressure
- Antihypertensive medication
- Physical activity and exercise of moderate to vigorous intensity
- Dietary interventions (including salt reduction, Mediterranean or DASH diet)
- Lifestyle advice (such as advice on smoking cessation, moderation of alcohol consumption, weight reduction and generic advice regarding the interventions above).

This set comprised interventions known to reduce blood pressure, including those recommended in hypertension management guidelines. These were grouped into the above pre-specified categories. Where a study arm included interventions combining two or more of the listed categories, it was classified as a 'multicomponent supplementary intervention' (e.g. home blood pressure monitoring plus exercise). Where a study arm combined interventions from the decision and supplementary sets, it was considered a mixed intervention and classified as a specific supplementary set intervention plus relaxation (regardless of the type of stress management intervention; e.g. diet plus relaxation).

Definitions of specific intervention and comparator categories that guided the grouping approach in this review are presented in Table S1.

Table S1: Definitions of intervention and comparator categories

**Intervention**	**Definition**
Decision set	
Autogenic training	Self-relaxation training assisted by auto-suggestion including repetitive phrases and mental imagery that emphasize physical sensations of muscle relaxation, warmth, and heaviness in individual body parts.
Biofeedback	Any intervention that utilised a form of electronic monitoring of physiological functions (such as heart rate, blood pressure, body temperature, muscle tension, or electrical skin resistance/sweating) to provide feedback to the participant on their relaxation state. Participants were typically instructed to try to control their physiological responses by modulating the continuous feedback. These attempts may have been facilitated by a variety of other relaxation techniques, e.g., breathing control, meditation, progressive muscle relaxation.
Breathing control	Interventions that predominantly involved the use of specific breathing techniques. This included simple breathing exercises, such as abdominal

	breathing or slow breathing (aiming to achieve a specific respiration rate), that may be unassisted or device-guided. Specific breathing exercises from other relaxation techniques were also included, such as yogic breathing (pranayama), when used without other features of yoga.
Hypnosis	Interventions involving either self-hypnosis, or hypnosis induced by a researcher, where participants' relaxed state was encouraged.
Massage therapy	Any intervention involving systematic manual manipulation of soft tissue (such as rubbing or kneading), including foot massage, back massage, Swedish massage.
Meditation	Interventions where participants maintain mental focus on a particular feeling, image, or mantra, and become highly attentive to specific sensory information while avoiding unrelated distracting thoughts. Specific techniques included mantra meditation (repetition of a specific word or a phrase), transcendental meditation (aiming to achieve a state of restful alertness while supressing ordinary thinking processes), and zen meditation (breath counting).
Meditative movement	Mind-body interventions consisting of three integrated components: postures/movements, controlled breathing, and meditation. These specifically included yoga, tai chi, and qigong, grouped together as conceptually similar practices of different origin (Indian and Chinese). The slow and controlled postures/movements used would be consistent with low intensity physical activity (i.e. too light to directly improve cardiorespiratory fitness).
Mindfulness	Interventions based on awareness of one's internal states and surroundings, intentionally directing one's attention to the present moment and noticing individual experiences (thoughts, emotions, physical sensations) with openness and acceptance. Mindfulness would aim to help participants avoid automatic (and often destructive) responses to these experiences. Practices utilising this concept included mindfulness meditation and mindfulness-based stress reduction.
Multicomponent	Interventions combining two or more of the listed decision set intervention categories (e.g. meditation and progressive muscle relaxation).
Music therapy	Interventions involving structured sessions of listening to specific music for a predefined period of time while not engaging in other activities, or more active engagement with live music, music recreation, improvisation, and composition in a form of music therapy.
Progressive muscle relaxation	Training in consciously tensing and releasing specific muscle groups sequentially, to achieve muscle relaxation throughout the body. Some of these interventions also included practice in applying this relaxation technique in imagined and real-life situations.
Psychotherapeutic approaches	Comprehensive interventions focused on developing and training stress coping skills and increasing resilience to stress. Participants often learned how to identify and modify maladaptive thoughts, feelings, and behaviours. Therapies may have been delivered in a standardised or individualised manner

	considering individual psychological risk factors. This included approaches such as cognitive therapy (including cognitive restructuring), behavioural therapy, cognitive-behavioural therapy, anger management / assertiveness training and problem solving.
Comparator set	
Nonspecific comparators	These comparators were intended to control for certain components of an active intervention, while not using techniques regarded as therapeutic. Examples are to control for the contact time with a therapist or research staff, the level of participant engagement, or nonspecific components of interventions of interest - such as support or educational materials, or expectancies of improvement. Example comparators would be office blood pressure monitoring (attending regular office visits only to have blood pressure measured, distinct from outcome assessments or follow-up visits attended by all participants), rest (being instructed to rest and/or relax for similar durations of time as an active intervention group without mention of any specific relaxation techniques), attention control (engaging in a specific activity, education, or discussion with a therapist or research staff unrelated to stress, relaxation, or hypertension), nonspecific therapy (discussing stress or stressful events without advising participants on how to deal with them, or discussing the importance of relaxation without mention of any specific techniques), sham intervention (usually in the context of sham biofeedback, where participants may be connected to a device but receive fake, distorted, or no feedback), light exercise (low intensity physical activity not expected to produce cardiovascular benefits, such as stretching, warm-up routines, walking) or placebo medication (as a way of controlling for expectations of improvement).
Passive comparators	Included control arms described as no intervention (not receiving any new treatment and only attending the same assessment or follow-up visits as other intervention groups), waitlist (not receiving any new treatment during the initial intervention period but being offered the same treatment as one of the other intervention groups at a later stage), and standard care (also described as routine or usual care, specifically for individuals with high blood pressure; including regular blood pressure checks, lifestyle advice or education regarding diet, salt reduction, exercise, smoking and alcohol reduction, and/or continued pharmacological treatment).
Supplementary set	
Diet	Dietary programmes beyond simple advice to follow a particular diet, including educational classes or consultations with a dietician/nutritionist, provision of specific eating/meal plans, cooking classes, and/or monitoring compliance. Specific interventions followed DASH, Mediterranean, and low-sodium diets.

Exercise	Programmes of at least moderate or vigorous intensity aerobic exercise or resistance exercise known to be beneficial for cardiovascular health, either supervised or with monitored compliance.
Home blood pressure monitoring	Regular self-monitoring of blood pressure at home, following training or instructions provided by research staff and using a provided device, often including keeping of written records.
Lifestyle intervention	Interventions aimed at lifestyle modification where comprehensive information was provided to participants during designated session(s) and may cover advice regarding diet, salt reduction, exercise, weight loss, smoking cessation, and/or adherence to medication. Unlike the remaining supplementary interventions, adherence or implementation of the provided advice did not have to be monitored.
Medication	Interventions introducing new antihypertensive medication as part of the study, in previously unmedicated participants or after undergoing a medication washout period (in contrast to continuation of existing medication prescribed by participant's physician, that was regarded as 'standard care')
Multicomponent supplementary intervention	Combination of two or more listed supplementary set interventions (e.g., diet and exercise).

Data extraction and missing data

All outcome data were extracted and prepared for analysis following guidance in Chapter Six of the Cochrane Handbook for Systematic Reviews of Interventions (5). For continuous outcomes, we also followed the NICE Guidelines Technical Support Unit guidance on Meta-Analysis of Continuous Outcomes (6). Following the TSU guidance for order of data extraction and analysis preference, arm-level outcome data were prioritised over contrast-level summaries. Where possible, we also preferred raw summaries over model-based, or adjusted, estimates, because adjusted estimates were not commonly reported and factors adjusted for were not consistent across the eligible studies For the systolic and diastolic blood pressure outcomes, the TSU guidance was operationalised as follows:

1. *Within-arm mean change-from-baseline (CfB), and standard deviation (SD):*

For analysis, the standard error (SE) of the within-arm mean change-from-baseline SE(CfB) was derived from reported within-study statistics (e.g. SD, p-values, confidence intervals).

2. *Within-arm baseline N, mean and SD(mean), and follow-up N, mean and SD(mean):*

Where the within-arm mean CfB or SD(CfB) were not reliably reported, they were calculated from arm-level means and SD at baseline and follow-up, using an empirical estimate of the within-arm pre-post correlation. This correlation was estimated as 0.5 from other studies in our dataset. Sensitivity of the results to an alternative correlation value of 0.7 was also explored using the empirically derived estimates from Balk *et al* (7).

3. *Mean difference (MD) in change from baseline and standard error (SE) [contrast level, CfB]:*

If arm-based data were unavailable, then contrast-level MD CfB and SE (MD CfB) were utilised. If necessary, SE (MD CfB) was derived from within-study statistics (e.g. p-values, confidence intervals).

Where either SD(CfB) or SE(CfB) were missing and could not be derived from within-study statistics, they were imputed from other studies within the dataset. Baseline and follow-up SDs of mean BP values were estimated using complete baseline data, separately for systolic and diastolic BP, and separately for participants with hypertension and pre-hypertension. For hypertension, the resulting average SDs were 12.026 for systolic, and 7.33 for diastolic BP. For pre-hypertension, the estimated average SDs were 7.837 for systolic, and 5.846 for diastolic BP. These SDs were applied to baseline and follow-up estimates used to calculate any missing SDs or SEs for change from baseline estimates, and those outcome data were considered to be imputed. A sensitivity analysis was conducted, excluding studies with imputed SDs or SEs. Where either a within-arm mean change-from-baseline or contrast-level mean difference in change from baseline could not be derived from reported data, we referred to the TSU guidance for alternatives. Where necessary, we followed guidance in the Cochrane handbook to account for clustering in the data (8).

If multiple blood pressure measurement methods were reported within a single study, we selected a single measure to be used in the analysis. For the purposes of analysis, outcomes were grouped according to the follow-up time: short-term (up to 3 months), medium term (>3-12 months) and long-term (over 12 months). If a study reported multiple time points within these time frames, we selected the longest duration of follow-up for analysis (e.g., if a study reported at 4, 6 and 10 weeks then the 10-week data were used in the short-term analysis). We prioritised 24-hour ambulatory blood pressure measures, as these are widely accepted as a reference standard test for blood pressure monitoring and have been shown to have good correlation with health outcomes (9) (if full 24-hour data are unavailable, night-time or day-time only measures were selected). If ambulatory monitoring was not available for a particular timepoint, then we considered home blood pressure monitoring to be the next preferred measure, as these values are most similar to ambulatory measures (10, 11). In the absence of other data, we used office blood pressure measures in the analysis. Although this was our least preferred measure, it should be noted that it was the most frequently available outcome measure from the studies included.

NMA model fitting and selection

Network meta-analyses were conducted using the multiNMA package, available in R. MultiNMA estimates models in a Bayesian framework, using Stan (12). Random-effects models assuming a common between study heterogeneity parameter were fitted for all analyses, where sufficient data were available. We specified uninformative ('vague') prior distributions for intervention effect and the between study heterogeneity parameters. Fixed effect models were implemented as a sensitivity analysis and as part of model selection checks. Model convergence was assessed based on four chains using R-hat values. We also inspected MCMC trace plots and effective sample size values to ensure there were no serious convergence issues.

We specified uninformative priors for the intercept, treatment effect (both normal prior distribution with location = 0 and scale = 100), and heterogeneity (half-normal distribution with location = 0 and scale = 10), appropriate to the continuous nature of the outcomes and the range of observed study-level effects. Model fitting procedures commenced with the default sampling parameters. However, to obtain the final model estimates, the number of post-warmup iterations was increased to 10,000. Model convergence was assessed based on four chains using R-hat values, which should not exceed 1.01. Where important runtime warnings or convergence problems were noted, we decreased the step size by adjusting the target average proposal acceptance probability. Where a small number of

divergent transitions remained during model fitting, we inspected MCMC trace plots and effective sample size values to ensure there were no serious convergence issues.

As part of model selection, random-effects NMA models assuming homogeneous between-study variance were compared to fixed-effect models. Model fit was assessed based on the posterior mean residual deviance. For the purpose of model selection, the trade-off between the goodness-of-fit and model complexity was considered, with a difference in the deviance information criterion (DIC) of ≥5 points considered meaningful, with lower values preferred (13). For global assessment of the inconsistency assumption, random-effects NMA models assuming consistency between direct and indirect evidence were also compared to those allowing for inconsistency (unrelated mean effects models, i.e. models which assume intervention effects for separate comparisons are unrelated and separate intervention effects can be estimated). Where global assessment suggested potential inconsistency (e.g. based on a better fit or substantial reduction in between-study heterogeneity [tau] in the inconsistency model) we further examined the potential sources of inconsistency via deviance contributions plots and plotting residual deviance from the consistency model against that from the inconsistency model. Further investigation of inconsistency employed a local node-splitting approach to identify the problematic comparisons (14). If inconsistency could not be explained by data extraction or imputation errors, or specific study-level effect modifiers, we planned to only report pairwise meta-analyses. Additional details on model parameters, model fit statistics and selection process are reported in Table S4, Table S5, Table S6, Table S7, Table S8, Table S9.

Supplementary results

Excluded studies

Table S2: Excluded studies list

Reference	Reason for exclusion
Aalasyam N, Goothy SSK, Mukkadan JK. Effectiveness of structured “om” chanting and listening program on psychological parameters in pre-hypertensive women. National journal of physiology, pharmacy and pharmacology 2021;11(10):1095-8. https://dx.doi.org/10.5455/njppp.2021.11.04117202110052021	Incorrect outcome
Aberg H, Tibblin G. Addition of non-pharmacological methods of treatment in patients on antihypertensive drugs: results of previous medication, laboratory tests and life quality. Journal of Internal Medicine 1989;226(1):39-46.	Incorrect intervention
Aburizik A, Dindo L, Kaboli P, Charlton M, Dawn K, Turvey C. A pilot randomized controlled trial of a depression and disease management program delivered by phone. Journal of Affective Disorders 2013;151(2):769-74. https://dx.doi.org/10.1016/j.jad.2013.06.028	Incorrect population
Alabdulgader AA. Coherence: a novel nonpharmacological modality for lowering blood pressure in hypertensive patients. Global Advances in Health & Medicine 2012;1(2):56-64. https://dx.doi.org/10.7453/gahmj.2012.1.2.011	Incorrect study design
Allen DH, Puddey IB, Morton AR, Beilin LJ. A controlled study of the effects of aerobic exercise on antihypertensive drug requirements of essential hypertensive patients in the general practice setting. Clinical & Experimental Pharmacology & Physiology 1991;18(5):279-82.	Incorrect intervention
Allen JC, Jr., Hala, B, Shirore RM, Jafar TH. Statistical analysis plan for management of hypertension and multiple risk factors to enhance cardiovascular health in Singapore: the SingHypertension pragmatic cluster randomized controlled trial. Trials [Electronic Resource] 2021;22(1):66. https://dx.doi.org/10.1186/s13063-020-05016-4	Incorrect intervention
Almeida IDS, Andrade LS, Sousa AMM, Junior GC, Catai AM, Mota YL, et al. Is the Combination of Aerobic Exercise with Mat Pilates Better than Mat Pilates Training Alone on Autonomic Modulation Related to Functional Outcomes in Hypertensive Women? Secondary Analysis of a Randomized Controlled Trial. International Journal of Environmental Research & Public Health [Electronic Resource] 2022;19(17):25. https://dx.doi.org/10.3390/ijerph191710577	Incorrect intervention

Alsaqer K, Bebis H. Self-care of hypertension of older adults during COVID-19 lockdown period: a randomized controlled trial. Clinical Hypertension 2022;28(1):21. https://dx.doi.org/10.1186/s40885-022-00204-7	Incorrect intervention
Alsubaie M, Dickens C, Dunn BD, Gibson A, Ukoumunne OC, Evans A, et al. Feasibility and acceptability of mindfulness-based cognitive therapy compared with mindfulness-based stress reduction and treatment as usual in people with depression and cardiovascular disorders: A three-arm randomised controlled trial. Mindfulness 2020;11(1):30-50. https://dx.doi.org/10.1007/s12671-018-0999-8	Incorrect population
Alturki M, Liberman K, Delaere A, De Dobbeleer L, Knoop V, Mets T, et al. Effect of Antihypertensive and Statin Medication Use on Muscle Performance in Community-Dwelling Older Adults Performing Strength Training. Drugs & Aging 2021;38(3):253-63. https://dx.doi.org/10.1007/s40266-020-00831-5	Incorrect intervention
Amigo I, Buceta JM, Becona E, Bueno AM. Cognitive behavioural treatment for essential hypertension: A controlled study. Stress Medicine 1991;7(2):103-8. https://dx.doi.org/10.1002/smi.2460070209	Incorrect study design
An E. Mindfulness and lifestyle education for blood pressure reduction in hypertension. Dissertation Abstracts International: Section B: The Sciences and Engineering 2019;80(11):1-131.	Incorrect publication type
An E, Irwin MR, Doering LV, Brecht ML, Watson KE, Corwin E, et al. Mindfulness effects on lifestyle behavior and blood pressure: A randomized controlled trial. Health Science Reports 2021;4(2):e296. https://dx.doi.org/10.1002/hsr2.296	Incorrect study design
Anderson DE, Reeves AN, Mehling WE, Chesney MA. Capnometric feedback training decreases 24-h blood pressure in hypertensive postmenopausal women. BMC Cardiovascular Disorders 2021;21(1):447. https://dx.doi.org/10.1186/s12872-021-02240-x	Incorrect study design
Andrade I, Melo KCB, Andrade KTP, Almeida LG, Moreira SR. Pilates training reduces blood pressure in older women with type 2 diabetes: A randomized controlled trial. Journal of Bodywork & Movement Therapies 2022;30:168-75. https://dx.doi.org/10.1016/j.jbmt.2022.02.022	Incorrect intervention
Anjana K, Archana R, Mukkadan JK, Jidhu G. Changes in inflammatory biomarkers in hypertensive subjects due to om chanting-a randomized controlled trial. NeuroQuantology 2022;20(8):3321-6. 10.14704/nq.2022.20.8.NQ44361	Incorrect outcome
Anonymous. MRC trial of treatment of mild hypertension. British Medical Journal Clinical Research Ed 1985;291(6491):346-7.	Incorrect publication type
Anonymous. Reducing stress can drive down blood pressure. Health News 2001;7(6):7.	Incorrect publication type
Anonymous. For the patient. Spiritual program proven to reduce high blood pressure. Ethnicity & Disease 2007;17(4):765.	Incorrect publication type
Anonymous. Resperate for hypertension. Medical Letter on Drugs & Therapeutics 2007;49(1264):55-6.	Incorrect publication type
Araya R, Menezes PR, Claro HG, Br, t LR, Daley KL, et al. Effect of a Digital Intervention on Depressive Symptoms in Patients With Comorbid Hypertension or Diabetes in Brazil and Peru: Two Randomized Clinical Trials. JAMA 2021;325(18):1852-62. https://dx.doi.org/10.1001/jama.2021.4348	Incorrect intervention
Arora R, Dhokpatil S, Verma C, Rajadhyaksha G. Effect of Buteyko Breathing Technique on Haemodynamic Parameters and Functional Capacity in Subjects with Primary Hypertension. Indian Journal of Physiotherapy & Occupational Therapy 2022;16(3):73-80. 10.37506/ijpot.v16i3.18400	Incorrect study design
Astuti NF, Rekawati E, Wati DNK. Decreased blood pressure among community dwelling older adults following progressive muscle relaxation and music therapy (RESIK). BMC Nursing 2019;18:36. https://dx.doi.org/10.1186/s12912-019-0357-8	Incorrect study design
Augusto TRL, Peroni J, de Vargas W, Santos PC, Dantas W, Padavini RL, et al. Carotid-body modulation through meditation in stage-I hypertensive subjects: Study protocol of a randomized and controlled study. Medicine 2023;102(1):e32295. https://dx.doi.org/10.1097/MD.0000000000032295	Incorrect publication type
Aweto HA, Owoeye OB, Akinbo SR, Onabajo AA. Effects of dance movement therapy on selected cardiovascular parameters and estimated maximum oxygen consumption in hypertensive patients. Nigerian Quarterly Journal of Hospital Medicine 2012;22(2):125-9.	Incorrect intervention
Bali LR. Long-term effect of relaxation on blood pressure and anxiety levels of essential hypertensive males: a controlled study. Psychosomatic medicine 1979;41(8):637-46.	Incorrect study design

Basler H-D, Brinkmeier U, Buser K, Haehn K-D, Molders-Kober R. Psychological group treatment of obese essential hypertensives by lay therapists in rural general practice settings. Journal of Psychosomatic Research 1985;29(4):383-91. https://dx.doi.org/10.1016/0022-3999%2885%2990024-8	Incorrect study design
Basler HD, Brinkmeier U, Buser K, Haehn KD, Molders-Kober R. Psychological group treatment of essential hypertension in general practice. British Journal of Clinical Psychology 1982;21:295-302.	Incorrect study design
Batista JP, Tavares JB, Goncalves LF, de Souza TCF, Mariano IM, Amaral AL, et al. Mat Pilates training reduces blood pressure in both well-controlled hypertensive and normotensive postmenopausal women: a controlled clinical trial study. Clinical & Experimental Hypertension (New York) 2022;44(6):548-56. https://dx.doi.org/10.1080/10641963.2022.2079670	Incorrect study design
Bertera EM, Bertera RL. The cost-effectiveness of telephone vs clinic counseling for hypertensive patients: a pilot study. American Journal of Public Health 1981;71(6):626-9.	Incorrect study design
Billion L. The Effect of EMG Biofeedback, Relaxation, and Sham EEG Alpha Training on Blood Pressure of Essential Hypertensives. PhD thesis. Charlottesville: University of Virginia; 1980	Incorrect publication type
Bittman B, Poornima I, Smith MA, Heidel RE. Gospel Music: A Catalyst for Retention, Engagement, and Positive Health Outcomes for African Americans in a Cardiovascular Prevention and Treatment Program. Advances in Mind-Body Medicine 2020;34(1):8-16.	Incorrect population
Blanchard EB, McCaffrey RJ, Musso A, Gerardi MA, McCoy GC. A controlled comparison of thermal biofeedback and relaxation training in the treatment of essential hypertension: III. Psychological changes accompanying treatment. Biofeedback and self-regulation 1987;12(3):227-40.	Incorrect outcome
Blom K, How M, Dai M, Baker B, Irvine J, Abbey S, et al. Hypertension Analysis of stress Reduction using Mindfulness meditatiON and Yoga (The HARMONY Study): study protocol of a randomised control trial. BMJ Open 2012;2(2):e000848. https://dx.doi.org/10.1136/bmjopen-2012-000848	Incorrect publication type
Blumenthal JA, Emery CF, Madden DJ, George LK, Coleman RE, Riddle MW, et al. Cardiovascular and behavioral effects of aerobic exercise training in healthy older men and women. Journal of Gerontology 1989;44(5):M147-57.	Incorrect population
Blumenthal JA, Siegel WC, Appelbaum M. Failure of exercise to reduce blood pressure in patients with mild hypertension. Results of a randomized controlled trial. JAMA 1991;266(15):2098-104.	Incorrect intervention
Bosley FM. A Study of the Effectiveness of a Stress Management Program on Hypertension. PhD thesis. St. Louis: Washington University; 1982.	Incorrect publication type
Boulware LE, Ephraim PL, Hill-Briggs F, Roter DL, Bone LR, Wolff JL, et al. Hypertension Self-management in Socially Disadvantaged African Americans: the Achieving Blood Pressure Control Together (ACT) Randomized Comparative Effectiveness Trial. Journal of General Internal Medicine 2020;35(1):142-52. https://dx.doi.org/10.1007/s11606-019-05396-7	Incorrect intervention
Boutin-Foster C, Offidani E, Kanna B, Ogedegbe G, Ravenell J, Scott E, et al. Results from the Trial Using Motivational Interviewing, Positive Affect, and Self-Affirmation in African Americans with Hypertension (TRIUMPH). Ethnicity & Disease 2016;26(1):51-60. https://dx.doi.org/10.18865/ed.26.1.51	Incorrect intervention
Buby C, Elfner LF, May Jr JG. Relaxation pretraining, pulse wave velocity and thermal biofeedback in the treatment of essential hypertension. International journal of psychophysiology 1990;9(3):225-30. 10.1016/0167-8760%2890%2990054-H	Incorrect study design
Buist CMDC. Reducing essential hypertension in the elderly using biofeedback-assisted self-regulation training. Dissertation Abstracts International: Section B: The Sciences and Engineering 2002;63(1):516.	Incorrect publication type
Bush MF. Combined relaxation and cognitive restructuring skills in the control of borderline essential hypertension. PhD thesis. Urbana-Champaign: University of Illinois; 1988.	Incorrect publication type
Bynum JL. Christian Meditation and Biofeedback Training as Psychotherapeutic Agents in the Treatment of Essential Hypertension. Unpublished PhD thesis. Fort Worth: Southwestern Baptist Theological Seminary; 1980	Incorrect publication type
Cade WT, Reeds DN, Mondy KE, Overton ET, Grassino J, Tucker S, et al. Yoga lifestyle intervention reduces blood pressure in HIV-infected adults with cardiovascular disease risk factors. HIV Medicine 2010;11(6):379-88. https://dx.doi.org/10.1111/j.1468-1293.2009.00801.x	Incorrect population
Calderon R, Jr. Effects of nonpharmacological approaches on cholesterol levels in mild hypertensive African Americans: A pilot study of the transcendental meditation program and a health education program. Dissertation Abstracts International: Section B: The Sciences and Engineering 2000;61(3):1619.	Incorrect publication type

Can Cicek S, Demir S, Yilmaz D, Acikgoz A, Yildiz S, Yis OM. The Effect of Aromatherapy on Blood Pressure and Stress Responses by Inhalation and Foot Massage in Patients With Essential Hypertension: Randomized Clinical Trial. Holistic Nursing Practice 2022;36(4):209-22. https://dx.doi.org/10.1097/HNP.0000000000000526	Incorrect duration of study
Carson MA, Hathaway A, Tuohey JP, McKay BM. The effect of a relaxation technique on coronary risk factors. Behavioral Medicine 1988;14(2):71-7.	Incorrect population
Chaerudin M, Ulfiana E, Wahyuni ED. The Effects of Deep Breathing on Blood Pressure Reduction in Elderly Hypertensive Patients at a Retirement Home in Surabaya. Medico-legal update 2020;20(4):343-8. 10.37506/mlu.v20i4.1833	Incorrect study design
Chan AW, Sit JW, Chair SY, Leung DY, Lee DT, Wong EM, et al. Evaluation of the Effectiveness of Tai Chi versus Brisk Walking in Reducing Cardiovascular Risk Factors: Protocol for a Ranomized Controlled Trial. International Journal of Environmental Research & Public Health [Electronic Resource] 2016;13(7):05. https://dx.doi.org/10.3390/ijerph13070682	Incorrect publication type
Charlson ME, Boutin-Foster C, Mancuso CA, Peterson JC, Ogedegbe G, Briggs WM, et al. Randomized controlled trials of positive affect and self-affirmation to facilitate healthy behaviors in patients with cardiopulmonary diseases: rationale, trial design, and methods. Contemporary Clinical Trials 2007;28(6):748-62.	Incorrect population
Chin GR, Greeson JM, Hughes JW, Fresco DM. Does Dispositional Mindfulness Predict Cardiovascular Reactivity to Emotional Stress in Prehypertension? Latent Growth Curve Analyses from the Serenity Study. Mindfulness 2021;12(11):2624-34. https://dx.doi.org/10.1007/s12671-021-01745-y	Incorrect study design
Christoph P, Luborsky L, Kron R, Fishman H. Blood pressure, heart rate and respiratory responses to a single session of relaxation: a partial replication. Journal of Psychosomatic Research 1978;22(6):493-501.	Incorrect duration of study
Chu PN. Identifying high-value lifestyle interventions for cardiovascular disease prevention. Dissertation Abstracts International: Section B: The Sciences and Engineering 2018;78(12):1-124	Incorrect population
Cohen BE, Chang AA, Grady D, Kanaya AM. Restorative yoga in adults with metabolic syndrome: a randomized, controlled pilot trial. Metabolic Syndrome & Related Disorders 2008;6(3):223-9. https://dx.doi.org/10.1089/met.2008.0016	Incorrect population
Cohen DL, Bowler A, Fisher SA, Norris A, Newberg A, Rao H, et al. Lifestyle Modification in Blood Pressure Study II (LIMBS): study protocol of a randomized controlled trial assessing the efficacy of a 24 week structured yoga program versus lifestyle modification on blood pressure reduction. Contemporary Clinical Trials 2013;36(1):32-40. https://dx.doi.org/10.1016/j.cct.2013.05.010	Incorrect publication type
Cooper MI. Effect of relaxation on blood pressure and serum cholesterol. Activitas Nervosa Superior 1982;(Suppl. 3, Pt. 2):428-36.	Incorrect study design
Cota ESLA, Gouvea TM, Fern, es FC, Carrillo M, Veloso VM, et al. Yoga practice can reduce metabolic syndrome and cardiovascular risk in climacteric women. Journal of Behavioral Medicine 2023;9:09. https://dx.doi.org/10.1007/s10865-023-00420-y	Incorrect population
Crowther JH. Stress management training and relaxation imagery in the treatment of essential hypertension. Journal of Behavioral Medicine 1983;6(2):169-87.	Incorrect study design
da Silva Almeida I, de Souza Andrade L, de Sousa AMM, Junior GC, Turri-Silva N, Cunha Nascimento DD, et al. The Effect of Mat Pilates Training Combined With Aerobic Exercise Versus Mat Pilates Training Alone on Blood Pressure in Women With Hypertension: A Randomized Controlled Trial. Physical Therapy 2022;102(2):01. https://dx.doi.org/10.1093/ptj/pzab258	Incorrect intervention
Dagistan Akgoz A, Gozum S. Effectiveness of a nurse-led physical activity intervention to decrease cardiovascular disease risk in middle-aged adults: A pilot randomized controlled study. Journal of Vascular Nursing 2020;38(3):140-8. https://dx.doi.org/10.1016/j.jvn.2020.05.002	Incorrect intervention
Datey KK, Deshmukh SN, Dalvi CP, Vinekar SL. "Shavasan": A yogic exercise in the management of hypertension. Angiology 1969;20(6):325-33.	Incorrect study design
Datta SK, Oddone EZ, Olsen MK, Orr M, McCant F, Gentry P, et al. Economic analysis of a tailored behavioral intervention to improve blood pressure control for primary care patients. American Heart Journal 2010;160(2):257-63. https://dx.doi.org/10.1016/j.ahj.2010.05.024	Incorrect intervention
Davis SK, Quarells R, Gibbons GH. A comprehensive cardiovascular disease lifestyle treatment controlled trial among high-risk African Americans. Open Journal of Preventive Medicine 2013;3(9):526-33.	Incorrect intervention

Davison GC, Williams ME, Nezami E, Bice TL, DeQuattro VL. Relaxation, reduction in angry articulated thoughts, and improvements in borderline hypertension and heart rate. Journal of Behavioral Medicine 1991;14(5):453-68.	Incorrect study design
Day AL, Gillan L, Francis L, Kelloway EK, Natarajan M. Massage therapy in the workplace: reducing employee strain and blood pressure. Giornale Italiano di Medicina del Lavoro Ed Ergonomia 2009;31(3):B25-30.	Incorrect population
de Matos Chicayban L, Novaes Malagris LE. Breathing and relaxation training for patients with hypertension and stress. Estudos de Psicologia 2014;31(1):115-26.	Incorrect study design
Deabler HL, Fidel E, Dillenkoffer RL, Elder ST. The use of relaxation and hypnosis in lowering high blood pressure. American Journal of Clinical Hypnosis 1973;16(2):75-83. https://dx.doi.org/10.1080/00029157.1973.10403656	Incorrect study design
Devraj JP, Santosh Kumar B, Raja Sriswan M, Jagdish B, Priya BS, Neelu SB, et al. Effect of Yoganidra on Blood Pressure, Hs-CRP, and Lipid Profile of Hypertensive Subjects: A Pilot Study. Evidence-Based Complementary & Alternative Medicine: eCAM 2021;2021:2858235. https://dx.doi.org/10.1155/2021/2858235	Incorrect study design
Dhameja K, Singh S, Mustafa MD, Singh KP, Banerjee BD, Agarwal M, et al. Therapeutic effect of yoga in patients with hypertension with reference to GST gene polymorphism. Journal of Alternative & Complementary Medicine 2013;19(3):243-9. https://dx.doi.org/10.1089/acm.2011.0908	Incorrect study design
Dhungana RR, Khanal MK, Joshi S, Kalauni OP, Shakya A, Bhrutel V, et al. Impact of a structured yoga program on blood pressure reduction among hypertensive patients: study protocol for a pragmatic randomized multicenter trial in primary health care settings in Nepal. BMC Complementary & Alternative Medicine 2018;18(1):207. https://dx.doi.org/10.1186/s12906-018-2275-9	Incorrect publication type
Drozdz T, Bilo G, Debicka-Dabrowska D, Klocek M, Malfatto G, Kielbasa G, et al. Blood pressure changes in patients with chronic heart failure undergoing slow breathing training. Blood Pressure 2016;25(1):4-10. https://dx.doi.org/10.3109/08037051.2016.1099800	Incorrect population
Duraimani S, Schneider RH, all OS, Nidich SI, Xu S, Ketete M, et al. Effects of Lifestyle Modification on Telomerase Gene Expression in Hypertensive Patients: A Pilot Trial of Stress Reduction and Health Education Programs in African Americans. PLoS ONE [Electronic Resource] 2015;10(11):e0142689. https://dx.doi.org/10.1371/journal.pone.0142689	Incorrect study design
Dye CJ, Williams JE, Evatt JH. Activating Patients for Sustained Chronic Disease Self-Management: Thinking Beyond Clinical Outcomes. Journal of Primary Care & Community Health 2016;7(2):107-12. https://dx.doi.org/10.1177/2150131915626562	Incorrect intervention
Eaker ED, Benfari RC, Reed RB. Coronary risk factor intervention: Characteristics associated with change. Journal of Clinical Psychology 1982;38(4):703-17. https://dx.doi.org/10.1002/1097-4679%28198210%2938:4%3C703::AID-JCLP2270380404%3E3.0.CO;2-C	Incorrect intervention
Edelman D, Oddone EZ, Liebowitz RS, Yancy WS, Olsen MK, Jeffreys AS, et al. A multidimensional integrative medicine intervention to improve cardiovascular risk. Journal of general internal medicine 2006;21(7):728-34. 10.1111/j.1525-1497.2006.00495.x	Incorrect population
Mandel SE, Davis BA, Secic M. Effects of music therapy and music-assisted relaxation and imagery on health-related outcomes in diabetes education: a feasibility study. Diabetes Educator 2013;39(4):568-81. https://dx.doi.org/10.1177/0145721713492216	Incorrect study design
Ephraim PL, Hill-Briggs F, Roter DL, Bone LR, Wolff JL, Lewis-Boyer L, et al. Improving urban African Americans' blood pressure control through multi-level interventions in the Achieving Blood Pressure Control Together (ACT) study: a randomized clinical trial. Contemporary Clinical Trials 2014;38(2):370-82. https://dx.doi.org/10.1016/j.cct.2014.06.009	Incorrect intervention
Erbeck JR, Elfner LF, Driggs DF. Reduction of blood pressure by indirect biofeedback. Biofeedback & Self Regulation 1983;8(1):63-72.	Incorrect duration of study
Esonis SS. The Relative Efficacy of the Relaxation Response, the Self-Control Triad and Food Sensitivity Intervention in the Treatment of Hypertension. Dissertation Abstracts International 1986;47(6B):2613.	Incorrect publication type
Fadlilah S, Erwanto R, Sucipto A, Anita DC, Aminah S. Soak feet with warm water and progressive muscle relaxation therapy on blood pressure in hypertension elderly. Pakistan journal of medical and health sciences 2020;14(3):1444-8.	Incorrect study design
Farinatti P, Monteiro WD, Oliveira RB. Long Term Home-Based Exercise is Effective to Reduce Blood Pressure in Low Income Brazilian Hypertensive Patients: A Controlled Trial. High Blood Pressure & Cardiovascular Prevention 2016;23(4):395-404. https://dx.doi.org/10.1007/s40292-016-0169-9	Incorrect study design

Frankfurt P. A home-based biofeedback intervention in a hypertensive African American sample: A pilot study. Dissertation Abstracts International: Section B: The Sciences and Engineering 2018;78(8):1443.	Incorrect publication type
Frost RO, Holmes DS. Effects of instructions and biofeedback for increasing and decreasing systolic blood pressure. Journal of Psychosomatic Research 1980;24(1):21-7.	Incorrect duration of study
Garcia-Vera MP, Sanz J. How many self-measured blood pressure readings are needed to estimate hypertensive patients' "true" blood pressure? Journal of Behavioral Medicine 1999;22(1):93-113.	Incorrect publication type
Garcia-Vera MP, Sanz J, Labrador FJ. Blood pressure variability and stress management training for essential hypertension. Behavioral Medicine 2004;30(2):53-62.	Incorrect outcome
Gaskin DJ, Zare H, Ibe CA, Yang M, Jones W, Gaston M, et al. The impact of the Prime Time Sister Circles® (PTSC) on blood pressure of low-income mid-life African American women in the United States. Journal of Public Health Policy 2023;44(4):616-33. https://dx.doi.org/10.1057/s41271-023-00450-5	Incorrect intervention
Gerage AM, Ritti-Dias RM, do Nascimento MA, Pina FL, Goncalves CG, Sardinha LB, et al. Chronic resistance training does not affect post-exercise blood pressure in normotensive older women: a randomized controlled trial. Age 2015;37(3):63. https://dx.doi.org/10.1007/s11357-015-9801-1	Incorrect population
Gholami M, Hafezi F, Asgari P, Naderi F. Comparison of the Effectiveness of Mindfulness and Spiritual/Religious Coping Skills on Health Hardiness and Somatic Complaints of Elderly with Hypertension. Health, Spirituality & Medical Ethics Journal 2017;4(3):19-26.	Incorrect outcome
Ginsberg GM, Viskoper JR, Fuchs Z, Drexler I, Lubin F, Berlin S, et al. Partial cost-benefit analysis of two different modes of nonpharmacological control of hypertension in the community. Journal of Human Hypertension 1993;7(6):593-7.	Incorrect intervention
Given CW, Given BA, Coyle BW. The effects of patient characteristics and beliefs on responses to behavioral interventions for control of chronic diseases. Patient education and counseling 1984;6(3):131-40. 10.1016/0738-3991(84)90070-3	Incorrect intervention
Givi M. Durability of effect of massage therapy on blood pressure. International Journal of Preventive Medicine 2013;4(5):511-6.	Incorrect duration of study
Glasgow MS, Engel BT, D'Lugoff BC. A controlled study of a standardized behavioral stepped treatment for hypertension. Psychosomatic Medicine 1989;51(1):10-26.	Incorrect study design
Glasgow MS, Gaarder KR, Engel BT. Behavioral treatment of high blood pressure II. Acute and sustained effects of relaxation and systolic blood pressure biofeedback. Psychosomatic Medicine 1982;44(2):155-70.	Incorrect study design
Goebel M, Viol GW, Lorenz GJ, Clemente J. Relaxation and biofeedback in essential hypertension: A preliminary report of a six-year project. American Journal of Clinical Biofeedback 1980;3(1):20-9.	Incorrect study design
Goebel M, Viol GW, Orebaugh C. An incremental model to isolate specific effects of behavioral treatments in essential hypertension. Biofeedback & Self Regulation 1993;18(4):255-80.	Incorrect study design
Goldstein IB, Shapiro D, Thananopavaran C. Home relaxation techniques for essential hypertension. Psychosomatic Medicine 1984;46(5):398-414.	Incorrect study design
Gordon MA. The effects of measurement frequency on baseline blood pressures in unmedicated hypertensives. Dissertation Abstracts International: Section B: The Sciences and Engineering 1995;56(1):0523.	Incorrect publication type
Greenberg LM. A pilot randomized controlled trial comparing the efficacy of problem-solving therapy to enhanced treatment as usual for reducing high blood pressure. Dissertation Abstracts International: Section B: The Sciences and Engineering 2016;76(11):No-Specified.	Incorrect publication type
Haaga DAF, Davison GC, Williams ME, Dolezal SL, Haleblian J, Rosenbaum J, et al. Mode-specific impact of relaxation training for hypertensive men with Type A behavior pattern. Behavior Therapy 1994;25(2):209-23. https://dx.doi.org/10.1016/S0005-7894%2805%2980284-9	Incorrect outcome
Haber D. Yoga as a preventive health care program for white and black elders: an exploratory study. International Journal of Aging & Human Development 1983;17(3):169-76.	Incorrect population
Hafer DG. Self-directed relaxation as a treatment for essential hypertension. PhD thesis. Denton: North Texas State University; 1984.	Incorrect publication type

Hahn YB, Ro YJ, Song HH, Kim NC, Kim HS, Yoo YS. The effect of thermal biofeedback and progressive muscle relaxation training in reducing blood pressure of patients with essential hypertension. Image - the Journal of Nursing Scholarship 1993;25(3):204-7.	Incorrect study design
Han Y, Tian H, DeJi L. Effect of CSMS Scale Combined with Narrative Psychological Nursing on Rehabilitation of Hypertensive Patients with Coronary Heart Disease. Alternative Therapies in Health & Medicine 2023;29(6):182-6.	Incorrect intervention
Hausswirth C, Nesi X, Dubois A, Duforez F, Rougier Y, Slattery K. Four Weeks of a Neuro-Meditation Program Improves Sleep Quality and Reduces Hypertension in Nursing Staff During the COVID-19 Pandemic: A Parallel Randomized Controlled Trial. Frontiers in Psychology 2022;13:854474. https://dx.doi.org/10.3389/fpsyg.2022.854474	Incorrect study design
Henderson R, Hart M, Lal S, Hunyor S. The effect of home practice of direct blood pressure biofeedback outcome in hypertensives. Australian and New Zealand journal of medicine 1998;28:82.	Duplicate record
Hernandez R, Cohn M, Hernandez A, Daviglus M, et al. A Web-Based Positive Psychological Intervention to Improve Blood Pressure Control in Spanish-Speaking Hispanic/Latino Adults With Uncontrolled Hypertension: Protocol and Design for the Alegrate! Randomized Controlled Trial. JMIR Research Protocols 2020;9(8):e17721. https://dx.doi.org/10.2196/17721	Incorrect publication type
Hernandez R, Daviglus ML, Martinez L, Durazo-Arvizu RA, Huffman JC, et al. "iAlegrate!"-A culturally adapted positive psychological intervention for Hispanics/Latinos with hypertension: Rationale, design, and methods. Contemporary Clinical Trials Communications 2019;14:100348. https://dx.doi.org/10.1016/j.conctc.2019.100348	Incorrect publication type
Hildebrandt MJ. Examining the efficacy of acceptance and commitment therapy for reducing cardiovascular risk in patients diagnosed with hypertension. Dissertation Abstracts International: Section B: The Sciences and Engineering 2015;75(10):No-Specified.	Incorrect publication type
Hunter SD, Kavouras SA, Rahimi M. Exploring heated exercise as a means of preventing the deleterious effects of high-sodium intake in Black women. American Journal of Physiology - Heart & Circulatory Physiology 2023;324(6):H833-H9. https://dx.doi.org/10.1152/ajpheart.00699.2022	Incorrect population
Ibe CA, Haywood DR, Creighton C, Cao Y, Gabriel A, Zare H, et al. Study protocol of a randomized controlled trial evaluating the Prime Time Sister Circles (PTSC) program's impact on hypertension among midlife African American women. BMC Public Health 2021;21(1):610. https://dx.doi.org/10.1186/s12889-021-10459-8	Incorrect publication type
Jacob RG, Shapiro AP, Reeves RA, Johnsen AM, McDonald RH, Coburn PC. Relaxation therapy for hypertension. Comparison of effects with concomitant placebo, diuretic, and beta-blocker. Archives of Internal Medicine 1986;146(12):2335-40.	Incorrect study design
Jafari F, Shahriari M. Effects of Lifestyle Education on Depression, Anxiety, Stress, and Perceived Family Support Among Hypertensive Patients. Medical-Surgical Nursing Journal 2021;10(3):1-9. https://dx.doi.org/10.5812/msnj.122691	Incorrect outcome
Jagadeesan T, Choudhary AK, Loganathan S, Rajendran K, Allu AR, Kuppusamy M. Yoga practice (Sheetali Pranayama) on cognition in patients with hypertension: A randomized controlled study. Integrative Medicine Research 2021;10(3):100716. https://dx.doi.org/10.1016/j.imr.2021.100716	Incorrect publication type
Jefferson LL. Exploring effects of therapeutic massage and patient teaching in the practice of diaphragmatic breathing on blood pressure, stress, and anxiety in hypertensive African-American women: an intervention study. Journal of National Black Nurses Association 2010;21(1):17-24.	Incorrect publication type
Jenaabadi H. Efficacy of anger management training on anger decrease of and blood pressure reactivity among patients with hypertension in Zahedan. Acta medica mediterranea 2018;34:607-12. https://dx.doi.org/10.19193/0393-6384_2018_2s_95	Incorrect study design
Johannesson M, Aberg H, Agreus L, Borgquist L, Jonsson B. Cost-benefit analysis of non-pharmacological treatment of hypertension. Journal of Internal Medicine 1991;230(4):307-12.	Incorrect study design
Jones CU, Sangthong B, Pachirat O, Jones DA. Slow breathing training reduces resting blood pressure and the pressure responses to exercise. Physiological research 2015;64(5):673-82. 10.33549/physiolres.932950	Incorrect outcome
Kaholokula JK, Look M, Mabellos T, Ahn HJ, Choi SY, Sinclair KA, et al. A Cultural Dance Program Improves Hypertension Control and Cardiovascular Disease Risk in Native Hawaiians: A Randomized Controlled Trial. Annals of Behavioral Medicine 2021;55(10):1006-18. https://dx.doi.org/10.1093/abm/kaaa127	Incorrect intervention
Khan MRK. Can a diabetes self-management program improve diabetes distress? Analysis from a randomized clinical trial. Dissertation Abstracts International: Section B: The Sciences and Engineering 2022;83(5):No-Specified.	Incorrect publication type

Ko J, Deprez D, Shaw K, Alcorn J, Hadjistavropoulos T, Tomczak C, et al. Stretching is Superior to Brisk Walking for Reducing Blood Pressure in People With High-Normal Blood Pressure or Stage I Hypertension. Journal of physical activity & health 2021;18(1):21-8. https://dx.doi.org/10.5812/msnj.12269110.1123/jpah.2020-0365	Incorrect intervention
Kohn JN, Lobo JD, Troyer EA, Ang G, Wilson KL, Walker AL, et al. Tai Chi versus health education as a frailty intervention for community-dwelling older adults with hypertension. Aging-Clinical & Experimental Research 2023;35(10):2051-60. https://dx.doi.org/10.1007/s40520-023-02504-w	Duplicate record
Kolbe-Alexander TL, Lambert EV, Charlton KE. Effectiveness of a community based low intensity exercise program for older adults. Journal of Nutrition, Health & Aging 2006;10(1):21-9.	Incorrect study design
Kondwani KA. Nonpharmacologic treatment of hypertensive heart disease in African-Americans: A trial of the transcendental meditation program and a health education program. Dissertation Abstracts International: Section B: The Sciences and Engineering 1998;59(6):3114.	Incorrect publication type
Kordvarkane Z, Oshvandi K, Mohammadi Y, Azizi A. Effect of education based on the Common-Sense Model of Self-Regulation on blood pressure and self-management of hypertensive patients: A clinical trial study. International Journal of Nursing Sciences 2023;10(3):294-301. https://dx.doi.org/10.1016/j.ijnss.2023.06.009	Incorrect intervention
Kostis JB, Rosen RC, Brondolo E, Taska L, Smith DE, Wilson AC. Superiority of nonpharmacologic therapy compared to propranolol and placebo in men with mild hypertension: a randomized, prospective trial. American Heart Journal 1992;123(2):466-74.	Incorrect intervention
Krishna BH, Pulaganti M, Sekhar AC, Jampala S. Exploring the effects of yoga therapy on cardiovascular risk profile, cardiac workload, and oxygen demand in individuals with prehypertension: a pilot study. International journal of academic medicine and pharmacy 2023;5(5):1595-600. 10.47009/jamp.2023.5.5.313	Incorrect study design
Kunikullaya KU, Goturu J, Muradi V, Hukkeri PA, Kunnavil R, Doreswamy V, et al. Music versus lifestyle on the autonomic nervous system of prehypertensives and hypertensives - a randomized control trial. Complementary Therapies in Medicine 2015;23(5):733-40.	Duplicate record
Kusuma AS, Nandeesh NS, Shetty S, Shetty P. Immediate effect of trataka on blood pressure indices in individuals with primary hypertension - A randomized controlled trial. Arterial hypertension (poland) 2021;25(2):82-7. https://dx.doi.org/10.5812/msnj.12269110.5603/AH.a2021.0013	Incorrect duration of study
Larkin KT, Zayfert C. Anger management training with mild essential hypertensive patients. Journal of Behavioral Medicine 1996;19(5):415-33.	Incorrect study design
Lau C, Yu R, Woo J. Effects of a 12-Week Hatha Yoga Intervention on Metabolic Risk and Quality of Life in Hong Kong Chinese Adults with and without Metabolic Syndrome. PloS one 2015;10(6):e0130731. 10.1371/journal.pone.0130731	Incorrect study design
Lee MS, Lee MS, Choi ES, Chung HT. Effects of Qigong on blood pressure, blood pressure determinants and ventilatory function in middle-aged patients with essential hypertension. American Journal of Chinese Medicine 2003;31(3):489-97.	Incorrect study design
Lee MS, Lee MS, Kim HJ, Choi ES. Effects of qigong on blood pressure, high-density lipoprotein cholesterol and other lipid levels in essential hypertension patients. International Journal of Neuroscience 2004;114(7):777-86.	Incorrect study design
Lee MS, Lim HJ, Lee MS. Impact of qigong exercise on self-efficacy and other cognitive perceptual variables in patients with essential hypertension. Journal of Alternative & Complementary Medicine 2004;10(4):675-80.	Incorrect study design
Lee SH, Hwang SM, Kang DH, Yang HJ. Brain education-based meditation for patients with hypertension and/or type 2 diabetes: A pilot randomized controlled trial. Medicine 2019;98(19):e15574. https://dx.doi.org/10.1097/MD.0000000000015574	Incorrect population
Lee SH, Kim BJ, Park IH, Hwang EH, Park EJ, Jang I, et al. Effects of taichi on grade 1 hypertension: A study protocol for a randomized controlled trial. Trials [Electronic Resource] 2020;21(1):177. https://dx.doi.org/10.1186/s13063-019-4028-6	Incorrect publication type
Lehnert H, Kaluza K, Vetter H, Losse H, Dorst K. Long-term effects of a complex behavioral treatment of essential hypertension. Psychosomatic Medicine 1987;49(4):422-30.	Incorrect study design
Leung LY, Chan AW, Sit JW, Liu T, Taylor-Piliae RE. Tai Chi in Chinese adults with metabolic syndrome: A pilot randomized controlled trial. Complementary Therapies in Medicine 2019;46:54-61. https://dx.doi.org/10.1016/j.ctim.2019.07.008	Incorrect population

Levenson JC, Rollman BL, Ritterb, LM, Strollo PJ, Smith KJ, et al. Hypertension with unsatisfactory sleep health (HUSH): study protocol for a randomized controlled trial. Trials [Electronic Resource] 2017;18(1):256. https://dx.doi.org/10.1186/s13063-017-2001-9	Incorrect publication type
Li X, Gao Y, Wu M, Wei D, Xiong X, Yang Y, et al. Effect of Tai Chi versus aerobic exercise on blood pressure in prehypertension patients (TCOBPP): a study protocol for a 12-month single-blind randomized controlled trial. Trials [Electronic Resource] 2022;23(1):1001. https://dx.doi.org/10.1186/s13063-022-06840-6	Incorrect publication type
Li Y, Zhong D, Dong C, Shi L, Zheng Y, Liu Y, et al. The effectiveness and safety of Tai Chi for patients with essential hypertension: study protocol for an open-label single-center randomized controlled trial. BMC Complementary Medicine and Therapies 2021;21(1):23. https://dx.doi.org/10.1186/s12906-020-03192-z	Incorrect publication type
Liu N, Fu D, Chen Y, Nie K, Wang J, Deng T, et al. Study on the effect of liuzijue exercise on blood pressure and oxidative stress in patients with essential hypertension. Acta medica mediterranea 2022;38(5):3429-35. https://dx.doi.org/10.5812/msnj.12269110.19193/0393-6384_2022_5_507	Incorrect study design
Lo HM, Yeh CY, Chang SC, Sung HC, Smith GD. A Tai Chi exercise programme improved exercise behaviour and reduced blood pressure in outpatients with hypertension. International Journal of Nursing Practice 2012;18(6):545-51. https://dx.doi.org/10.1111/ijn.12006	Incorrect study design
Lorber M, Divjak S. Music Therapy as an Intervention to Reduce Blood Pressure and Anxiety Levels in Older Adults With Hypertension: A Randomized Controlled Trial. Research in Gerontological Nursing 2022;15(2):85-92. https://dx.doi.org/10.3928/19404921-20220218-03	Incorrect population
Loucks EB, Kronish IM, Saadeh FB, Scarpaci MM, Proulx JA, Gutman R, et al. Effects of Adapted Mindfulness Training on Interoception and Adherence to the Dietary Approaches to Stop Hypertension (DASH) Diet: The MB-BP Randomized Clinical Trial. MedRxiv : the Preprint Server for Health Sciences 2023;15:15. https://dx.doi.org/10.1101/2023.05.10.23289818	Incorrect outcome
Loucks EB, Kronish IM, Saadeh FB, Scarpaci MM, Proulx JA, Gutman R, et al. Adapted Mindfulness Training for Interoception and Adherence to the DASH Diet: A Phase 2 Randomized Clinical Trial. JAMA Network Open 2023;6(11):e2339243. https://dx.doi.org/10.1001/jamanetworkopen.2023.39243	Incorrect outcome
Loucks EB, Schuman-Olivier Z, Saadeh FB, Scarpaci MM, Nardi WR, Proulx JA, et al. Effect of Adapted Mindfulness Training in Participants With Elevated Office Blood Pressure: The MB-BP Study: A Randomized Clinical Trial. Journal of the American Heart Association 2023;12(11):e028712. https://dx.doi.org/10.1161/JAHA.122.028712	Duplicate record
Mao GX, Cao YB, Lan XG, He ZH, Chen ZM, Wang YZ, et al. Therapeutic effect of forest bathing on human hypertension in the elderly. Journal of Cardiology 2012;60(6):495-502. https://dx.doi.org/10.1016/j.jjcc.2012.08.003	Incorrect duration of study
Marmot M, Patel C, Terry D. Risk factor reduction by biofeedback in the factory. Journal of Psychosomatic Research 1979;23(6):433.	Incorrect publication type
Martins-Meneses DT, Antunes HK, de Oliveira NR, Medeiros A. Mat Pilates training reduced clinical and ambulatory blood pressure in hypertensive women using antihypertensive medications. International Journal of Cardiology 2015;179:262-8. https://dx.doi.org/10.1016/j.ijcard.2014.11.064	Incorrect study design
Marwaha K. Mind-body medicine in cardiovascular health: Mechanisms and clinical outcomes of transcendental meditation in primary and secondary prevention of cardiovascular disease. Dissertation Abstracts International: Section B: The Sciences and Engineering 2020;81(3):No-Specified.	Incorrect publication type
McGrady A, et al. Effect of direct feedback of systolic blood pressure in essential hypertension. American Journal of Clinical Biofeedback 1978;1(2):58-60.	Incorrect study design
McGrath ER, Espie CA, Murphy AW, Newell J, Power A, Madden S, et al. Sleep to lower elevated blood pressure: study protocol for a randomized controlled trial. Trials [Electronic Resource] 2014;15:393. https://dx.doi.org/10.1186/1745-6215-15-393	Incorrect publication type
McGrath ER, Espie CA, Power A, Murphy AW, Newell J, Kelly C, et al. Sleep to Lower Elevated Blood Pressure: A Randomized Controlled Trial (SLEPT). American Journal of Hypertension 2017;30(3):319-27. https://dx.doi.org/10.1093/ajh/hpw132	Incorrect intervention
Meles E, Giannattasio C, Failla M, Gentile G, Capra A, Mancia G. Nonpharmacologic treatment of hypertension by respiratory exercise in the home setting. American Journal of Hypertension 2004;17(4):370-4.	Incorrect study design
Mensorio MS, Cebolla-Marti A, Rodilla E, Palomar G, Lison JF, Botella C, et al. Analysis of the efficacy of an internet-based self-administered intervention ("Living Better") to promote healthy habits in a population with obesity and	Incorrect intervention

hypertension: An exploratory randomized controlled trial. International Journal of Medical Informatics 2019;124:13-23. https://dx.doi.org/10.1016/j.ijmedinf.2018.12.007	
Metgud S, Mahadevan D, D'Souza V, Dourado E. Effect of Music Therapy versus Aromatherapy with Aerobic Exercise on Stage 2 Hypertension: A Randomized Controlled Trial. Indian Journal of Physical Therapy & Research 2023;5(1):46-50. https://dx.doi.org/10.5812/msnj.12269110.4103/ijptr.ijptr_35_22	Incorrect duration of study
Miller RN. Study on the effectiveness of remote mental healing. Medical Hypotheses 1982;8(5):481-90.	Incorrect intervention
Mirzaei A, Alipour A, Safarinia M. Comparison of the Effectiveness of Mindfulness-based Stress Reduction Training and Positive Psychology Intervention in Hostility and Physiological Indicators among Cardiovascular Patients. Razavi international journal of medicine 2022;10(4). https://dx.doi.org/10.5812/msnj.12269110.30483/RIJM.2022.254263.1101	Incorrect population
Mizuno J, Monteiro HL. An assessment of a sequence of yoga exercises to patients with arterial hypertension. Journal of bodywork and movement therapies 2013;17(1):35-41. https://dx.doi.org/10.5812/msnj.12269110.1016/j.jbmt.2012.10.007	Incorrect study design
Moeini M, Givi M, Ghasempour Z, Sadeghi M. The effect of massage therapy on blood pressure of women with pre-hypertension. Iranian Journal of Nursing and Midwifery Research 2011;16(1):61-70.	Incorrect duration of study
Mohammadi R, Javanmard GH, Alipour A, Zare H. Effects of mindful breath awareness and muscle relaxation and transcranial electrical stimulation techniques on improving blood pressure status in patients with type 2 diabetes. Explore: The Journal of Science & Healing 2022;18(2):200-4. https://dx.doi.org/10.1016/j.explore.2021.05.002	Incorrect population
Naumann J, Bureau N, Schmidt S, Sadaghiani C, Huber R. A single center three-arm parallel-group, randomized controlled study to evaluate antihypertensive effects of frequent immersion in thermoneutral water. International Journal of Cardiology 2015;188:73-5. https://dx.doi.org/10.1016/j.ijcard.2015.04.022	Incorrect publication type
Naumann J, Sadaghiani C, Bureau N, Schmidt S, Huber R. Outcomes from a three-arm randomized controlled trial of frequent immersion in thermoneutral water on cardiovascular risk factors. BMC Complementary & Alternative Medicine 2016;16:250. https://dx.doi.org/10.1186/s12906-016-1241-7	Incorrect intervention
Nazzaro P, Mudoni A, Manzari M, Merlo M, Pieri R, Panettieri I, et al. Efficacy of biofeedback treatment compared with drug therapy in hypertensive patients. Functional Neurology 1991;6(1):49-57.	Incorrect study design
Nidich SI, Rainforth MV, Haaga DA, Hagelin J, Salerno JW, Travis F, et al. A randomized controlled trial on effects of the Transcendental Meditation program on blood pressure, psychological distress, and coping in young adults. American Journal of Hypertension 2009;22(12):1326-31. https://dx.doi.org/10.1038/ajh.2009.184	Incorrect population
Nolan RP, Floras JS, Ahmed L, Harvey PJ, Hiscock N, Hendrickx H, et al. Behavioural modification of the cholinergic anti-inflammatory response to C-reactive protein in patients with hypertension. Journal of Internal Medicine 2012;272(2):161-9. https://dx.doi.org/10.1111/j.1365-2796.2012.02523.x	Incorrect outcome
Nualnim N, Parkhurst K, Dhindsa M, Tarumi T, Vavrek J, Tanaka H. Effects of swimming training on blood pressure and vascular function in adults >50 years of age. American Journal of Cardiology 2012;109(7):1005-10. https://dx.doi.org/10.1016/j.amjcard.2011.11.029	Incorrect study design
Okawa J. A Systems Approach to Mild Essential Hypertension: educational Lifestyle Adjustments Versus Biobehavioral Techniques. PhD thesis. Salt Lake City: University of Utah; 1986.	Incorrect publication type
Olah M, Koncz A, Feher J, Kalmanczhey J, Olah C, Nagy G, et al. The effect of balneotherapy on antioxidant, inflammatory, and metabolic indices in patients with cardiovascular risk factors (hypertension and obesity)--a randomised, controlled, follow-up study. Contemporary Clinical Trials 2011;32(6):793-801. https://dx.doi.org/10.1016/j.cct.2011.06.003	Incorrect intervention
Olney CM. The effect of therapeutic back massage in hypertensive persons: a preliminary study. Biological Research for Nursing 2005;7(2):98-105.	Incorrect duration of study
Olney CM. Back massage: long term effects and dosage determination for persons with pre-hypertension and hypertension. PhD thesis. Tampa: University of South Florida; 2007.	Incorrect publication type
Olney CM. Determining long term effects and dosage of back massage for persons with elevated blood pressure: a clinical trial. Southern Online Journal of Nursing Research 2008;8(2):66-76.	Incorrect publication type
Oluwatelure FA. The effect of rational emotive therapy in blood pressure reduction and attitude change among hypertensives. IFE Psychologia: An International Journal 1997;5(2):89-97.	Incorrect study design

Pal PK, Saini N, Mishra VN, Awasthi HH. Evaluation of the effect of yogic practices on raktagata vata (Essential hypertension). Asian journal of pharmaceutical and clinical research 2018;11(9):425-30. https://dx.doi.org/10.22159/ajpcr.2018.v11i9.27734	Incorrect study design
Palta P, Page G, Piferi RL, Gill JM, Hayat MJ, Connolly AB, et al. Evaluation of a mindfulness-based intervention program to decrease blood pressure in low-income African-American older adults. Journal of Urban Health 2012;89(2):308-16.	Incorrect population
Pandey NC, Shahid M, Sachdeva A. Evaluation of the Efficacy of Lifestyle Interventions in Preventing Recurrent Cardiovascular Events. Journal of cardiovascular disease research 2023;14(12):1015-23. https://dx.doi.org/10.48047/jcdr.2023.14.12.120	Incorrect population
Paran E, Amir M, Yaniv N. Evaluating the response of mild hypertensives to biofeedback-assisted relaxation using a mental stress test. Journal of Behavior Therapy & Experimental Psychiatry 1996;27(2):157-67.	Incorrect study design
Park JE, Liu Y, Park T, Hong S, Kim JE, Kim TH, et al. A trial for the use of qigong in the treatment of pre and mild essential hypertension: a study protocol for a randomized controlled trial. Trials [Electronic Resource] 2011;12:244. https://dx.doi.org/10.1186/1745-6215-12-244	Incorrect publication type
Patel C. Yoga and biofeedback in the management of hypertension. Journal of Psychosomatic Research 1975;19(5):355-60.	Incorrect study design
Patel C, Marmot MG, Terry DJ. Controlled trial of biofeedback-aided behavioural methods in reducing mild hypertension. British Medical Journal Clinical Research Ed 1981;282(6281):2005-8.	Incorrect population
Patel C, Marmot MG, Terry DJ, Carruthers M, Hunt B, Patel M. Trial of relaxation in reducing coronary risk: four year follow up. British Medical Journal Clinical Research Ed 1985;290(6475):1103-6.	Incorrect population
Patil SG, Aithala MR, Das KK. Effect of yoga on arterial stiffness in elderly subjects with increased pulse pressure: A randomized controlled study. Complementary Therapies in Medicine 2015;23(4):562-9. https://dx.doi.org/10.1016/j.ctim.2015.06.002	Incorrect population
Patil SG, Patil SS, Aithala MR, Das KK. Comparison of yoga and walking-exercise on cardiac time intervals as a measure of cardiac function in elderly with increased pulse pressure. Indian Heart Journal 2017;69(4):485-90. https://dx.doi.org/10.1016/j.ihj.2017.02.006	Incorrect population
Pender NJ. Physiologic responses of clients with essential hypertension to progressive muscle relaxation training. Research in Nursing & Health 1984;7(3):197-203.	Incorrect study design
Pender NJ. Effects of progressive muscle relaxation training on anxiety and health locus of control among hypertensive adults. Research in Nursing & Health 1985;8(1):67-72.	Incorrect study design
Perez Briones NG, Ruiz Paloalto ML, Casique Casique L, Ramirez-Giron N, Azucena Rodriguez Puente L, Ruiz Lara A, et al. Effect of Reiki Therapy on Blood Pressure and Alcohol Consumption in Young Adults: A Clinical Trial. Alternative Therapies in Health & Medicine 2022;5:05.	Incorrect intervention
Perry RT. The Effects of Systematic Relaxation Training and Circadian Rhythm on Systolic and Diastolic Blood Pressure in Borderline/Essential Hypertensive Subjects. PhD thesis. Commerce: East Texas State University; 1984.	Incorrect publication type
Pierce TW, Madden DJ, Siegel WC, Blumenthal JA. Effects of aerobic exercise on cognitive and psychosocial functioning in patients with mild hypertension. Health Psychology 1993;12(4):286-91. https://dx.doi.org/10.1037/0278-6133.12.4.286	Incorrect intervention
Pierce TW, Madden DJ, Siegel WC, Blumenthal JA. "Effects of aerobic exercise on cognitive and psychosocial functioning in patients with mild hypertension": Correction. Health Psychology 1993;12(5):389. https://dx.doi.org/10.1037/0278-6133.12.5.389	Incorrect publication type
Pikul A, Linchong P, Sirirat P, Rojanee C. Enhancing Autonomy and Self-Management Behaviors Through a Patient-Centered Communication Program for Older Adults with Hypertension: A Randomized Controlled Trial. Pacific Rim International Journal of Nursing Research 2021;25(4):525-38.	Incorrect outcome
Posser SR, Callegaro CC, Beltrami-Moreira M, Moreira LB. Effect of inspiratory muscle training with load compared with sham training on blood pressure in individuals with hypertension: study protocol of a double-blind randomized clinical trial. Trials [Electronic Resource] 2016;17:382. https://dx.doi.org/10.1186/s13063-016-1514-y	Incorrect publication type

Prajapati S, ArpitVerma, BalajiGhugare, Toshi N. Study to evaluate the role of yoga therapy in decreasing blood pressure among hypertensive subjects. Journal of cardiovascular disease research 2022;13(5):382-91. https://dx.doi.org/10.31838/jcdr.2022.13.05.37	Incorrect study design
Printz AM. Stress reduction in the treatment of essential hypertension: A clinical trial utilizing assertion and relaxation coping skills. Dissertation Abstracts International 1979;40(3):1379.	Incorrect publication type
Pugliese R, Zanella MT, Blay SL, Plavinik F, Andrade MA, Galvao R. Efficacy of lifestyle change psychological intervention in coronary risk reduction. Arquivos Brasileiros de Cardiologia 2007;89(4):225-30.	Incorrect intervention
Rajagopalan A, Krishna A, Mukkadan JK. Effect of Om chanting and Yoga Nidra on depression anxiety stress, sleep quality and autonomic functions of hypertensive subjects - a randomized controlled trial. Journal of Basic & Clinical Physiology & Pharmacology 2023;34(1):69-75. https://dx.doi.org/10.1515/jbcpp-2022-0122	Incorrect outcome
Ramakrishna P, Singh R, Sharma SN, Srivastava PK. A study of the use of relaxation technique in the treatment of essential hypertension. Indian Journal of Psychological Medicine 1988;11(2):85-92.	Incorrect study design
Reineke A. The effects of heart rate variability biofeedback in reducing blood pressure for the treatment of essential hypertension. Dissertation Abstracts International: Section B: The Sciences and Engineering 2008;68(7):4880.	Incorrect publication type
Richter-Heinrich E, Homuth V, Gohlke HR, Heinrich B, Schmidt KH, Wiedemann R, et al. Effectiveness of behavioral treatment methods compared to pharmacological therapy and self recordings of blood pressure in essential hypertensives (preliminary report). Activitas Nervosa Superior 1982;(Suppl. 3, Pt. 2):422-7.	Incorrect study design
Rodriguez MA, Wang B, Hyoung S, Friedberg J, Wylie-Rosett J, Fang Y, et al. Sustained Benefit of Alternate Behavioral Interventions to Improve Hypertension Control: A Randomized Clinical Trial. Hypertension 2021;77(6):1867-76. https://dx.doi.org/10.1161/HYPERTENSIONAHA.120.15192	Incorrect intervention
Rossi N, Caldari R, Costa FV, Ambrosioni E. Autogenic training in mild essential hypertension: a placebo-controlled study. Stress medicine 1989;5(1):63-8. https://dx.doi.org/10.1002/smi.2460050111	Incorrect study design
Sajjad Y, Qurat Ul A, Riaz T, Naveed QUA, Ashraf I, Asif M. Immediate effects of Diaphragmatic breathing versus pursed Lip breathing on blood pressure, pulse rate and oxygen saturation of patients with hypertension. Medical forum monthly 2021;32(9):48-52.	Incorrect duration of study
Sallis JF, Trevorrow TR, Johnson CC, Hovell MF, Kaplan RM. Worksite stress management: A comparison of programs. Psychology & Health 1987;1(3):237-55. https://dx.doi.org/10.1080/08870448708400328	Incorrect population
Santos JMD, Sousa Filho LF, Carvalho VO, Wichi RB, Oliveira ED. Hemodynamic and creatine kinase changes after a 12-week equipment-based Pilates training program in hypertensive women. Journal of Bodywork & Movement Therapies 2020;24(4):496-502. https://dx.doi.org/10.1016/j.jbmt.2020.06.020	Incorrect study design
Sarah S, Wolfgang MB, Claudia P. Effect of telerehabilitation on long-term adherence to yoga as an antihypertensive lifestyle intervention: Results of a randomized controlled trial. Complementary Therapies in Clinical Practice 2019;35:148-53. https://dx.doi.org/10.1016/j.ctcp.2019.02.001	Incorrect intervention
Sathe SS, Rajandekar T, Hadake S, Shegaonkar V. Immediate effect of buteyko breathing in hypertensive patients: an experimental prospective study. Indian journal of forensic medicine and toxicology 2020;14(4):7136-41. https://dx.doi.org/10.37506/ijfmt.v14i4.12771	Incorrect duration of study
Sathe SS, Rajandekar T, Thodge K, Bhawane A, Thatere U. Immediate effect of buteyko breathing and bhramari pranayama on blood pressure, heart rate and oxygen saturation in hypertensive patients: a comparative study. Indian journal of forensic medicine and toxicology 2020;14(4):7106-11. https://dx.doi.org/10.37506/ijfmt.v14i4.12764	Incorrect duration of study
Satterfield S, Cutler JA, Langford HG, Applegate WB, Borhani NO, Brittain E, et al. Trials of hypertension prevention. Phase I design. Annals of Epidemiology 1991;1(5):455-71.	Incorrect publication type
Schneider RH, Staggers F, Alexander CN, Sheppard W, Rainforth M, Kondwani K, et al. A randomized controlled trial of stress reduction for hypertension in older African Americans. Hypertension 1995;26(5):820-7.	Duplicate record
Schonfeld GM. Effectiveness of biofeedback assisted relaxation treatment of essential hypertension in the elderly. PhD thesis. Toronto: University of Toronto; 1992.	Incorrect publication type
Serrano-Guzman M, Valenza-Pena CM, Serrano-Guzman C, Aguilar-Ferrandiz E, Valenza-Demet G, Villaverde-Gutierrez C. Effects of a dance therapy programme on quality of life, sleep and blood pressure in middle-aged women: a randomised controlled trial. Medicina clinica 2016;147(8):334-9. 10.1016/j.medcli.2016.06.030	Incorrect intervention

Shoemaker JE, Tasto DL. The effects of muscle relaxation on blood pressure of essential hypertensives. Behaviour Research & Therapy 1975;13(1):29-43.	Incorrect duration of study
Siadat ZD, Hasandokht T, Farajzadegan Z, Paknahad Z. Effects of multicomponent lifestyle modification on blood pressure control in health centers: Design of the study. Journal of Research in Medical Sciences 2013;18(4):308-13.	Incorrect publication type
Sieverdes JC, Treiber FA, Kline CE, Mueller M, Brunner-Jackson B, Sox L, et al. Ethnicity Differences in Sleep Changes Among Prehypertensive Adults Using a Smartphone Meditation App: Dose-Response Trial. JMIR Formative Research 2020;4(10):e20501. https://dx.doi.org/10.2196/20501	Incorrect outcome
Siu PM, Yu AP, Benzie IF, Woo J. Effects of 1-year yoga on cardiovascular risk factors in middle-aged and older adults with metabolic syndrome: a randomized trial. Diabetology & metabolic syndrome 2015;7:40. https://dx.doi.org/10.1186/s13098-015-0034-3	Incorrect population
Southam MA. Generalized Effects of Relaxation Training in Essential Hypertension. PhD thesis. Stanford: Stanford University; 1981.	Incorrect publication type
Srinivasan B, Rajkumar D. Effects of slow breathing on blood pressure and end tidal carbon dioxide in hypertension: randomised controlled trial. Journal of clinical and diagnostic research 2019;13(9):YC01-YC3. https://dx.doi.org/10.7860/JCDR/2019/42327.13121	Incorrect duration of study
Stasi MF, Amati D, Costa C, Resta D, Senepa G, Scarafioiti C, et al. Pet-therapy: a trial for institutionalized frail elderly patients. Archives of Gerontology & Geriatrics 2004:(Suppl. 9):407-12.	Incorrect study design
Steptoe A. New approaches to the management of essential hypertension with psychological techniques. Journal of Psychosomatic Research 1978;22(4):339-54.	Incorrect publication type
Steptoe A, Patel C, Marmot M, Hunt B. Frequency of relaxation practice, blood pressure reduction and the general effects of relaxation following a controlled trial of behaviour modification for reducing coronary risk. Stress Medicine 1987;3(2):101-7. https://dx.doi.org/10.1002/smi.2460030206	Incorrect population
Still CH, Margevicius SP, Wright JT, Jr., Ruksakulpiwat S, Moore SM. A Pilot Study Evaluating the Effects of a Technology-Based and Positive Psychological Training Intervention on Blood Pressure in African Americans With Hypertension. Journal of Primary Care & Community Health 2021;12:21501327211056186. https://dx.doi.org/10.1177/21501327211056186	Incorrect intervention
Stone RA, DeLeo J. Psychotherapeutic control of hypertension. New England journal of medicine 1976;294(2):80-4. 10.1056/NEJM197601082940204	Incorrect study design
Stuhr JK. Randomized controlled anger intervention for hypertensives. Dissertation Abstracts International: Section B: The Sciences and Engineering 2004;64(8):4066.	Incorrect publication type
Subha M, Murugesan S. Effect of yogic practices on Low Density Lipoprotien (LDL) and High Density Lipoprotien (HDL) among hypertensive middle aged women. European journal of molecular and clinical medicine 2020;7(9):1090-5.	Incorrect outcome
Sung J, Woo JM, Kim W, Lim SK, Chung EJ. The effect of cognitive behavior therapy-based "forest therapy" program on blood pressure, salivary cortisol level, and quality of life in elderly hypertensive patients. Clinical & Experimental Hypertension (New York) 2012;34(1):1-7. https://dx.doi.org/10.3109/10641963.2011.618195	Incorrect study design
Supa'at I, Zakaria Z, Maskon O, Aminuddin A, Nordin NA. Effects of Swedish massage therapy on blood pressure, heart rate, and inflammatory markers in hypertensive women. Evidence-Based Complementary & Alternative Medicine: eCAM 2013;2013:171852. https://dx.doi.org/10.1155/2013/171852	Incorrect duration of study
Surwit RS, Shapiro D, Good MI. Comparison of cardiovascular biofeedback, neuromuscular biofeedback, and meditation in the treatment of borderline essential hypertension. Journal of Consulting & Clinical Psychology 1978;46(2):252-63.	Incorrect study design
Tang HY, Harms V, Speck SM, Vezeau T, Jesurum JT. Effects of audio relaxation programs for blood pressure reduction in older adults. European Journal of Cardiovascular Nursing 2009;8(5):329-36. https://dx.doi.org/10.1016/j.ejcnurse.2009.06.001	Incorrect population
Tang J, Harms V, Speck SM. Audio relaxation intervention for blood pressure reduction in older adults. Communicating Nursing Research 2008;41:216-.	Incorrect study design
Tedder M, Shi L, Si M, Franco R, Chen L. eMindfulness Therapy-A Study on Efficacy of Blood Pressure and Stress Control Using Mindful Meditation and Eating Apps among People with High Blood Pressure. Medicines 2015;2(4):298-309. https://dx.doi.org/10.3390/medicines2040298	Incorrect publication type

Tekn E, Ünver F, Yaylalı YT. Investigation of the effects of 8-week Nordic and traditional walking training on blood pressure in prehypertensive postmenopausal women. Spor Hekimligi Dergisi/Turkish Journal of Sports Medicine 2023;58(3):112-7. https://dx.doi.org/10.47447/tjsm.0717	Incorrect intervention
Tibbits D, Ellis G, Piramelli C, Luskin F, Lukman R. Hypertension reduction through forgiveness training. The Journal of Pastoral Care & Counseling: JPCC 2006;60(1):27-34.	Incorrect study design
Tolves T, Pippi CM, Moreira MB, Righi GA, Righi NC, Signori LU, et al. Pilates vs aerobic training effects in hypertensives: randomized trial. Revista brasileira de medicina do esporte 2024;30. 10.1590/1517-8692202430012021_0327i	Incorrect intervention
Toomey M. The effects of the transcendental meditation program on carotid atherosclerosis and cardiovascular disease risk factors in Native Hawaiians. Dissertation Abstracts International: Section B: The Sciences and Engineering 2007;68(6):4169.	Incorrect publication type
Ublosakka-Jones C, Tongdee P, Pachirat O, Jones DA. Slow loaded breathing training improves blood pressure, lung capacity and arm exercise endurance for older people with treated and stable isolated systolic hypertension. Experimental Gerontology 2018;108:48-53. https://dx.doi.org/10.1016/j.exger.2018.03.023	Incorrect intervention
Ubolsakka-Jones C, Tongdee P, Jones DA. The effects of slow loaded breathing training on exercise blood pressure in isolated systolic hypertension. Physiotherapy Research International 2019;24(4):e1785. https://dx.doi.org/10.1002/pri.1785	Incorrect intervention
Uherik A, Sebej F, Biro V. Psychophysiological aspects in prevention and treatment of hypertension. Studia Psychologica 1987;29(3):173-83.	Incorrect study design
Vasantha Priya J, Kanniammal C, Mahendra J, Valli G. Impact of yoga on blood pressure and quality of life in patients with hypertension. International journal of pharmaceutical and clinical research 2017;9(5):413-6.	Incorrect study design
Vyas AP. Effects of mindfulness and stress management on neurocardiovascular and psychological outcomes. Dissertation Abstracts International: Section B: The Sciences and Engineering 2023;84(7):1-173.	Incorrect publication type
Wang M-Y, Chang N-C, Hsieh M-H, Su C-T, Liu J-C, Shyu Y-K, et al. Effect of Feedback Signal on Blood Pressure Self-regulation Capability in Individuals With Prehypertension or Stage I Hypertension. Journal of cardiovascular nursing 2016;31(2):166-72. 10.1097/JCN.0000000000000239	Duplicate record
Wang J, Wu F, Wang B, Yao M, Yan Y. Clinical intervention of baduanjin on anxiety and depression of hypertensive elderly. Acta medica mediterranea 2023;39(1):241-6. 10.19193/0393-6384_2023_1_37	Incorrect outcome
Webb MS, Smyth KA, Yarandi H. A progressive relaxation intervention at the worksite for African-American women. Journal of National Black Nurses Association 2000;11(2):1-6.	Incorrect study design
Wenneberg SR, Schneider RH, Walton KG, Maclean CR, Levitsky DK, Salerno JW, et al. A controlled study of the effects of the Transcendental Meditation program on cardiovascular reactivity and ambulatory blood pressure. International Journal of Neuroscience 1997;89(1):15-28.	Incorrect population
Wiggins NE. A culturally sensitive approach to treating hypertensive African-Americans. Dissertation Abstracts International: Section B: The Sciences and Engineering 1996;57(4):2935.	Incorrect publication type
Wittrock DA, Blanchard EB, McCoy GC. Three studies on the relation of process to outcome in the treatment of essential hypertension with relaxation and thermal biofeedback. Behaviour Research & Therapy 1988;26(1):53-66.	Incorrect outcome
Wittrock DA, Blanchard EB, McCoy GC, McCaffrey RJ, Khramelashvili VV. The relationship of expectancies to outcome in stress management treatment of essential hypertension: results from the Joint USSR-USA Behavioral Hypertension Project. Biofeedback & Self Regulation 1995;20(1):51-63.	Incorrect outcome
Wolff M, Memon AA, Chalmers JP, Sundquist K, Midlov P. Yoga's effect on inflammatory biomarkers and metabolic risk factors in a high risk population - a controlled trial in primary care. BMC Cardiovascular Disorders 2015;15:91. https://dx.doi.org/10.1186/s12872-015-0086-1	Incorrect study design
Wolff M, Sundquist K, Larsson Lonn S, Midlov P. Impact of yoga on blood pressure and quality of life in patients with hypertension - a controlled trial in primary care, matched for systolic blood pressure. BMC Cardiovascular Disorders 2013;13:111. https://dx.doi.org/10.1186/1471-2261-13-111	Incorrect study design
Wong A, Figueroa A. Eight weeks of stretching training reduces aortic wave reflection magnitude and blood pressure in obese postmenopausal women. Journal of Human Hypertension 2014;28(4):246-50. https://dx.doi.org/10.1038/jhh.2013.98	Incorrect intervention

Wong A, Figueroa A, Fischer SM, Bagheri R, Park SY. The Effects of Mat Pilates Training on Vascular Function and Body Fatness in Obese Young Women With Elevated Blood Pressure. American Journal of Hypertension 2020;33(6):563-9. https://dx.doi.org/10.1093/ajh/hpaa026	Incorrect intervention
Wu Q, Ye B, Lv X, Mao G, Wang S, Chen Z, et al. Adjunctive therapeutic effects of cinnamomum camphora forest environment on elderly patients with hypertension. International journal of gerontology 2020;14(4):327-31. https://dx.doi.org/10.6890/IJGE.202011_14(4).0014	Incorrect duration of study
Xiao X, Deng X, Zhang G, Liu M, Fu D, Yang P, et al. Monitoring of the regulatory ability and regulatory state of the autonomic nervous system and its application to the management of hypertensive patients: a study protocol for randomised controlled trials. BMJ Open 2023;13(6):e063434. https://dx.doi.org/10.1136/bmjopen-2022-063434	Incorrect publication type
Xu J, Yang F, Si L, Qian D. Do integrated health care interventions improve well-being among older adults with hypertension? Evidence from rural China. Social Indicators Research 2022;160(2):825-43. https://dx.doi.org/10.1007/s11205-020-02482-w	Incorrect intervention
Xu SK, Chen Y, Liu CY, Spekowius G, van Ee R, de Jong M, et al. A randomized cross-over study on the blood pressure lowering effect of the combined passive head-up and -down movement with Device-Guided slow breathing. Blood Pressure 2019;28(5):291-9. https://dx.doi.org/10.1080/08037051.2019.1613884	Incorrect duration of study
Yang X, Zhang Y, Liu J, Liu Y, Lang Y, Wang Y, Jiang X. The efficacy of internet-based cognitive behavior therapy on blood pressure for comorbid hypertension and insomnia. Medical journal of Chinese people's liberation army 2017;42(4):331-5.	Incorrect publication type
Yoon NH, Yoo S, Kim H, Han Y. Routine Screening and Consultation Facilitate Improvement of Metabolic Syndrome. Journal of Korean Medical Science 2015;30(8):1092-100. https://dx.doi.org/10.3346/jkms.2015.30.8.1092	Incorrect intervention
Yung PM, Keltner AA. A controlled comparison on the effect of muscle and cognitive relaxation procedures on blood pressure: implications for the behavioural treatment of borderline hypertensives. Behaviour Research & Therapy 1996;34(10):821-6.	Incorrect study design
Zare H, Ibe CA, Yang M, Porter G, Gaston M, Jones N, et al. Evaluating the Impact of the Prime Time Sister Circles® Intervention on Reducing Depressive Symptoms Among African American Women with Uncontrolled Hypertension. Journal of general internal medicine 2023;38(13):2879-87. 10.1007/s11606-023-08288-z	Incorrect intervention
Zhang H, Jiang X, Da H, Dai R, Zhao N, Pan W, et al. Effect of comprehensive psychosomatic promotion in hypertension patients with anxiety and depression based on community: A randomized parallel controlled trial. Medicine 2020;99(33):e21451. https://dx.doi.org/10.1097/MD.0000000000021451	Incorrect publication type
Zhang Y, Mei S, Yang R, Chen L, Gao H, Li L. Effects of lifestyle intervention using patient-centered cognitive behavioral therapy among patients with cardio-metabolic syndrome: a randomized, controlled trial. BMC cardiovascular disorders 2016;16(1):227. https://dx.doi.org/10.1186/s12872-016-0398-9	Incorrect intervention
Zhao R, Yang S, Li D, Liu L, Xing Y, Wu M. Effects of Baduanjin Exercise on Antihypertensive Medication Reduction in Older Patients with Hypertension: A Study Protocol for a Randomized Controlled Trial. Evidence-Based Complementary & Alternative Medicine: eCAM 2021;2021:8663022. https://dx.doi.org/10.1155/2021/8663022	Incorrect publication type
Zheng G, Chen B, Fang Q, Lin Q, Tao J, Chen L. Baduanjin exercise intervention for community adults at risk of ischamic stroke: A randomized controlled trial. Scientific Reports 2019;9(1):1240. https://dx.doi.org/10.1038/s41598-018-37544-0	Incorrect population

Included studies

Table S3: Characteristics of included studies

Study	Country	Number randomised at baseline	Population	Percentage using antihypertensives	Age, mean (SD)	Age, other	Percentage of males	Interventions	BP measurement method	Mean baseline BP (systolic/diastolic, mmHg)	Timepoints of outcome assessment (months from baseline)			Notes
											≤3 months	>3 to 12 months	>12 months	
Achmon 1989 (15)	Israel	97	HTN	<50%	40.66 (8.58)	Range 25-60	73.1%	1. Lifestyle<br>2. Psychotherapy<br>3. Biofeedback	Office attended BP	155/98		4.25		
Adams 2018 (16)	United States	64	Pre-HTN	0%	35.1 (12.5)	Range >/=21 years	54.7%	1. Breathing control<br>2. Breathing control<br>3. Breathing control	Office attended BP	128/NR	3			Dose-response study.
Adsett 1989 (17)	Canada	47	HTN	0%	46.57 (8.23)	Range 30-65	100%	1. Lifestyle<br>2. Multicomponent supplementary<br>3. PMR<br>4. Medication and relaxation	Home BP	145/96	3	5		All men.
Agras 1987 (18)	United States	137	HTN	100%	52.82 (NR)		82%	1. Passive comparator<br>2. PMR	Office attended BP	146/98		12	30	
Ahmadpanah 2016 (19)	Iran	45	HTN	100%	46.49 (2.33)		0%	1. Nonspecific comparator<br>2. Mindfulness<br>3. Multicomponent	Office attended BP	161/108	2	4		All women with depression/anxiety symptoms. High baseline diastolic BP.
Aivazyan 1988a (20)	USSR	117	HTN	0%	35.76 (8.84)	Range 20-45	100%	1. Passive comparator<br>2. Nonspecific comparator<br>3. Autogenic training<br>4. Biofeedback<br>5. Multicomponent	Office BP	NR	1.5	12		All men.
Aivazyan 1988b (21)	USSR	90	HTN	NR	39.9 (1.46)	Range 20-50	78%	1. Passive comparator<br>2. Autogenic training	Office attended BP	165/100			60	
Altena 2009 (22)	Netherlands	30	HTN	100%	59.5 (11.17)		50%	1. Music<br>2. Breathing control	Home BP	131/79	2.25			
Amigo 1997 (23)	Spain	45	HTN	<50%	43 (12.59)	Range 18-60	53%	1. Nonspecific comparator<br>2. Exercise<br>3. PMR	Office attended BP	143/88	2	6		BP measurements "obtained during a session just after the treatment were considered as post-treatment scores".

Anderson 2010 (24)	United States	42	Pre-HTN and HTN	0%	53.15 (2.78)		53%	1. Meditation<br>2. Breathing control	24 hour ambulatory BP	138/83	1			
Anjana 2022 (25)	India	80	HTN	100%	46.64 (8.99)	Range 25-60	45%	1. Passive comparator<br>2. Meditation	Office attended BP	135/85	2.14			
Ankolekar 2019 (26)	India	102	Pre-HTN	NR	NR (NR)		NR	1. Passive comparator<br>2. Meditative movement	Office attended BP	134/86	3	6		
Arslan 2021 (27)	Turkey	90	HTN	100%	NR (NR)	6.7% aged 30-39;<br>5.6% aged 40-49;<br>36.7% aged 50-59;<br>32.2% aged 60-69;<br>14.4% aged 70-79;<br>4.4% aged 80-89	0%	1. Passive comparator<br>2. Massage<br>3. Massage	NA	NR	NA	NA	NA	No outcome data at appropriate time point (measured at 4 weeks but not reported and not provided by authors).
Babak 2022 (28)	Iran	80	HTN	NR	49 (1.94)	Range 30-59	0%	1. Passive comparator<br>2. Mindfulness	Office attended BP	141/85		3.25		All women, possibly with depression/anxiety symptoms.
Balasubramanian 2012 (29)	India	40	HTN	100%	52.57 (4.96)	Range 35-60	65%	1. Passive comparator<br>2. Breathing control	Office attended BP	140/84	1			
Batey 2000 (30)<br><br>(Related articles: Whelton 1992 (31), The Trials of Hypertension Prevention Collaborative Research Group 1992 (32), Whelton 1997 (33))	United States	562	Pre-HTN	0%	43.17 (6.73)	Range 30-54	71%	1. Passive comparator<br>2. Multicomponent	Office attended BP	125/84	3	12		Data extracted are a subset of that from a larger trial (including interventions not of relevance to this review).
Bekiroglu 2013 (34)	Turkey	60	HTN	>50%	NR (NR)	Range 60-89;<br>13.3% aged 60-69,<br>50% aged 70-79,<br>36.7% aged 80-89	57%	1. Nonspecific comparator<br>2. Music	Home BP	125/NR	1			All elderly, care home residents. Outcomes assessed immediately after intervention.
Bennett 1991 (35)	United Kingdom	47	HTN	0%	46 (NR)		100%	1. Passive comparator<br>2. Psychotherapy<br>3. Multicomponent	Office attended BP	152/93	2	8	0	8 months follow-up for arms 2 and 3 only. All male, with 'type A behaviour'.
Blanchard 1979 (36)	United States	33	HTN	<50%	39.5 (NR)	Range 23-56	48%	1. Nonspecific comparator<br>2. Biofeedback<br>3. Biofeedback	Office attended BP	146/95	3			
Blanchard 1984 (37)<br><br>(Related article: Blanchard 1986 (38))	United States	42	HTN	100%	48.6 (9.2)		55%	1. Biofeedback<br>2. PMR	Home BP	131/81	2			
Blanchard 1988 (39)	United States and USSR	59	HTN	0%	38.26 (9.87)	Range 21-61	100%	1. Nonspecific comparator<br>2. Autogenic training<br>3. Biofeedback	Office attended BP	145/97	2.5	8.5	0	8.5 months follow-up for arms 2 and 3 only. All men.
Blanchard 1993 (40)	United States	41	HTN	100%	51.55 (NR)		61%	1. Home BP monitoring<br>2. Biofeedback	Home BP	124/78	2			

								3. Biofeedback						
Blanchard 1996 (41)	United States	46	HTN	0%	50.5 (6.99)	Range 32-62	67%	1. Home BP monitoring<br>2. Biofeedback	Office attended BP	141/92	2			
Blom 2014 (42)	Canada	101	HTN	0%	55.99 (11.49)	Range 20-75	37%	1. Passive comparator<br>2. Mindfulness	24 hour ambulatory BP	134/82	3			
Bosley 1989 (43)	United States	41	HTN	100%	57 (NR)	Range 42-68	100%	1. Passive comparator<br>2. Nonspecific comparator<br>3. Psychotherapy	Office attended BP	136/88	2			All men.
Brauer 1979 (44)	United States	35	HTN	100%	57.23 (NR)		86%	1. Nonspecific comparator<br>2. PMR<br>3. PMR	Office attended BP	149/93	2.5	6		
Canino 1994 (45)	Venezuela	21	HTN	0%	35 (2)	Range 25-46	67%	1. Passive comparator<br>2. Nonspecific comparator<br>3. Multicomponent	Office BP	148/97	2			
Chan 2018 (46)	China	246	HTN	>50%	64.4 (9.75)	Range 30-91	45.5%	1. Nonspecific comparator<br>2. Exercise<br>3. Meditative movement	Office BP	141/81	3	9		
Chandler 2020 (47)	United States	84	Pre-HTN	0%	45.05 (13.43)	Range 18-90	49%	1. Lifestyle<br>2. Breathing control	Office attended BP	133/76	3	12		
Charlesworth 1984 (48)	United States	54	HTN	>50%	51 (9.74)	Range 23-65	80%	1. Home BP monitoring<br>2. Multicomponent	Home BP	132/84	2.5			
Chen 2016a (49)	China	60	HTN	NR	66.3 (5.8)		NR	1. Passive comparator<br>2. Meditative movement	Not reported	155/103		6		
Chen 2016b (50)	China	32	Pre-HTN	0%	21.5 (0.18)	Range 19-23	75%	1. Nonspecific comparator<br>2. Breathing control<br>3. Biofeedback	Office BP	129/81	3			
Chesney 1987 (51)	United States	158	HTN	0%	47.38 (NR)	42% aged 50-69 years	89%	1. Passive comparator<br>2. PMR<br>3. Multicomponent<br>4. Biofeedback<br>5. Biofeedback<br>6. Lifestyle and relaxation	Office attended BP	138/95			13.5	
Cheung 2005 (52)	China	91	HTN	0%	54.4 (9.05)	Range 18-75	42%	1. Nonspecific comparator<br>2. Meditative movement	24 hour ambulatory BP	129/84	3			
Clemow 2018 (53)	United States	92	HTN	>50%	48.5 (8.7)	Range 18-70	23%	1. Passive comparator<br>2. Psychotherapy	Office attended BP	148/91		4.5		
Cohen 1983 (54)	United States	30	HTN	>50%	44.47 (NR)	Range 26-72	43%	1. Home BP monitoring<br>2. Meditation<br>3. Biofeedback	Office attended BP	141/96	2.5	4		
Cohen 2011 (55)	United States	78	Pre-HTN and HTN	0%	48.24 (11.96)	Range 22-69	50%	1. Lifestyle<br>2. Meditative movement	24 hour ambulatory BP	134/82	3			
Cohen 2016 (56)	United States	137	Pre-HTN and HTN	0%	47.45 (12.96)	Range 18-80	49%	1. Lifestyle<br>2. Meditative movement<br>3. Lifestyle and relaxation	24 hour ambulatory BP	133/81	3	6		
Cottier 1984 (57)	United States	30	HTN	0%	34.69 (7.71)	Range 18-50	73%	1. Nonspecific comparator<br>2. PMR	Home BP	134/88		3.75		

Cramer 2018 (58)<br><br>(Related article: Guaman 2022 (59))	Germany	75	HTN	100%	58.7 (9.5)	Minimum 18 years	28%	1. Passive comparator<br>2. Breathing control<br>3. Meditative movement	24 hour ambulatory BP	133/82	3	7		Intervention described as yoga without postures - classified as a yogic breathing intervention for analysis.
de Barros 2017 (60)	Brazil	41	HTN	>50%	50.41 (9.94)		41%	1. Music<br>2. Breathing control	24 hour ambulatory BP	127/88	2			
de Fatima Rosas Marchiori 2015 (61)	Brazil	65	Pre-HTN and HTN	>50%	67.09 (5.11)	Minimum 60 years	36%	1. Passive comparator<br>2. Meditation	Office BP	146/85	3			
Dhungana 2021 (62)	Nepal	121	Pre-HTN and HTN	>50%	47.7 (10.7)	Range 18-70	52%	1. Lifestyle<br>2. Lifestyle and relaxation	Office attended BP	139/90		3.4		
Drazen 1982 (63)	United States	25	HTN	0%	40.3 (NR)	Range 22-62	73%	1. Lifestyle<br>2. PMR<br>3. Psychotherapy	Office attended BP	152/94	2.5	4.5		
Dusek 2008 (64)	United States	122	HTN	100%	66.8 (7.23)	Minimum 55 years	45%	1. Lifestyle<br>2. Meditation	Office attended BP	146/77	2			
Elavally 2020 (65)	India	346	HTN	100%	NR (NR)	Range 35-75; 14% aged 36-45, 10% aged 46-55, 29% aged 56-65, 47% aged 66-75	56%	1. Passive comparator<br>2. Biofeedback	Office attended BP	141/88	3	4		
Elliot 2004 (66)	United States	149	HTN	>50%	59 (10)	Range 40-75	50%	1. Home BP monitoring<br>2. Home BP monitoring and relaxation	Home BP	144/85	2			
Fetter 2020 (67)	Brazil	50	HTN	>50%	59.09 (3.81)	Range 45-68	0%	1. Meditative movement<br>2. Breathing control	24 hour ambulatory BP	139/87	3			All women, specifically post-menopausal.
Frankel 1978 (68)	United States	22	HTN	<50%	45.82 (10.24)		55%	1. Nonspecific comparator<br>2. Nonspecific comparator<br>3. Biofeedback	Office attended BP	148/95		4		
Friedman 1977a (69)<br><br>(Related article Friedman 1978 (70))	United States	23	HTN	>50%	47.58 (NR)	Range 23-60	83%	1. Hypnosis<br>2. Biofeedback	Office attended BP	141/93	1.75	6.75		Extracted as two subsets of study from a single article (Friedman 1977), as different populations were separately randomised to the interventions. Specifically included participants susceptible to hypnosis.
Friedman 1977b (69)<br><br>(Related article Friedman 1978 (70))	United States	25	HTN	>50%	47.73 (NR)	Range 29-59	80%	1. Nonspecific comparator<br>2. Biofeedback	Office attended BP	143/95	1.75	6.75		Extracted as two subsets of study from a single article (Friedman 1977), as different populations were separately randomised to the interventions.
Garcia Vera 1997 (71)	Spain	43	HTN	>50%	45.36 (8.83)		100%	1. Passive comparator<br>2. Multicomponent	Home BP	130/84	2	6		All men.
Gay 2007 (72)	France	31	HTN	>50%	47.2 (11.76)	Range 22-60	40%	1. Passive comparator<br>2. Hypnosis	Office attended BP	156/88	2	8		

Givi 2018 (73)	Iran	50	Pre-HTN	0%	NR (NR)	Range 18-60	0%	1. Nonspecific comparator<br>2. Massage therapy	Office attended BP	129/82	1.38			All women.
Grossman 2001 (74)	Israel	33	HTN	>50%	51.09 (9.19)	Range 25-75	70%	1. Music<br>2. Breathing control	Home BP	154/92	2			
Hafner 1982 (75)	United Kingdom	21	HTN	>50%	48.9 (NR)	Range 25-68	57%	1. Passive comparator<br>2. Meditation<br>3. Biofeedback	Office BP	155/102	2	5		Outcome assessment occurred immediately after intervention at short time point.
Hager 1978 (76)	United States	30	HTN	NR	NR (NR)		50%	1. Biofeedback<br>2. Meditation	NA	NR	NA	NA	NA	
Haghighat 2021 (77)	Iran	60	HTN	NR	NR (NR)		NR	1. Passive comparator<br>2. Psychotherapy	Not reported	162/80	1.5			Specifically recruited participants with alexithymia.
Hagins 2014 (78)	United States	84	Pre-HTN and HTN	NR	54.54 (11.08)	Range 21-70	14%	1. Nonspecific comparator<br>2. Meditative movement	24 hour ambulatory BP	135/81	3			
Hasandokht 2015 (79)	Iran	161	HTN	>50%	54.65 (4.87)		0%	1. Passive comparator<br>2. Lifestyle and relaxation	Office attended BP	158/100	1	7		Cluster RCT. All women.
Hatch 1985 (80)	United States	52	HTN	100%	51.1 (NR)	Range 21-70	40%	1. Passive comparator<br>2. Nonspecific comparator<br>3. PMR<br>4. Biofeedback	Home BP	136/87		12	15	
Henderson 1998 (81)	Australia	30	HTN	0%	54 (NR)	Range 38-67	60%	1. Nonspecific comparator<br>2. Biofeedback	Office attended BP	153/97		3.25		
Hernandez Reif 2000 (82)	United States	35	HTN	100%	51.6 (8.8)		30%	1. PMR<br>2. Massage therapy	Office attended BP	138/89	1.25			
Hoelscher 1986 (83)	United States	50	HTN	>50%	51.1 (13)		52%	1. Passive comparator<br>2. PMR<br>3. PMR<br>4. PMR	Office attended BP	149/96	2.5			
Hoelscher 1987 (84)	United States	48	HTN	>50%	51.9 (11.4)		NR	1. Passive comparator<br>2. PMR<br>3. PMR	Office attended BP	145/94	2			
Howorka 2013 (85)	Austria	32	HTN	100%	49.3 (11.7)	Range 18–78	53%	1. Passive comparator<br>2. Breathing control	24 hour ambulatory BP	126/73	2			Specifically recruited participants with diabetes.
Hughes 2013 (86)	United States	56	Pre-HTN	0%	50.3 (6.5)	Range 30-60	43%	1. PMR<br>2. Mindfulness	Office attended BP	130/78	2			
Huijuan 2021 (87)	China	60	HTN	NR	77.2 (4.55)	Range 50-90	40%	1. Passive comparator<br>2. Meditative movement	24 hour ambulatory BP	159/89	1			
Im-Oun 2018 (88)	Thailand	120	HTN	>50%	51.65 (8.77)	Range 40-80	39%	1. Passive comparator<br>2. Music	Home BP and office attended BP	134/80	1.07	4.29		BP measured at home for short time point, and office measurement at medium time point.
Irvine 1986 (89)	United Kingdom	38	HTN	>50%	47.75 (8.5)	Range 34-65	53%	1. Nonspecific comparator<br>2. Biofeedback	Home BP	136/86	2.5	4.5		
Irvine 1991 (90)	Canada	110	HTN	0%	46.25 (8.28)	Range 25-64	82%	1. Nonspecific comparator<br>2. Biofeedback	Office attended BP	137/94	3	6		
Ismail 2023 (91)	Egypt	60	HTN	NR	69.75 (4.16)	Minimum 65 years	37%	1. Passive comparator<br>2. Breathing control	Not reported	157/89	1			

Jacob 1985 (92)	United States	57	Pre-HTN and HTN	0%	54 (NR)	Range 37-65	54%	1. Passive comparator<br>2. Diet and relaxation	Office attended BP	144/86	2	12		
Jacob 1992 (93)	United States	20	HTN	100%	48.82 (10.1)	Range 38-68	68%	1. Nonspecific comparator<br>2. Biofeedback	24 hour ambulatory BP	121/83	2.5			Baseline systolic blood pressure lower than most studies.
Johnston 1993 (94)	United Kingdom	96	HTN	0%	46.6 (8.41)	Range 23-59	48%	1. Nonspecific comparator<br>2. Multicomponent	Daytime ambulatory BP	133/93		6		
Jones 2010 (95)	Thailand	27	HTN	100%	51.5 (4.67)	Range 35-65	35%	1. Passive comparator<br>2. Breathing control	Home BP	142/86	2			
Jorgensen 1981 (96)	United States	18	HTN	NR	54.35 (NR)		100%	1. Passive comparator<br>2. PMR	Office attended BP	138/86	1.5			All men.
Kalmatayeva 2014 (97)	Kazakhstan	75	HTN	NR	48.03 (3.94)		49%	1. Medication<br>2. Medication and relaxation	Not reported	158/98	2.5	3.5		Medication changes throughout the intervention and follow-up period were part of the intervention protocol (more than anticipated with usual care).
Katsarou 2014 (98)	Greece	45	HTN	>50%	63.43 (11.34)	Minimum 18 years	39%	1. Passive comparator<br>2. Diet and relaxation	Office attended BP	141/82	2			
Kohn 2023a (99)<br><br>(Related article: Kohn 2023b (100), additional data supplied by the author)	United States	182	Pre-HTN and HTN	NR	72.6 (7.9)	Range 60-93	28%	1. Lifestyle<br>2. Meditative movement	Office attended BP	135/69	3			Cluster RCT. Baseline diastolic BP lower than most studies.
Kow 2018 (101)	Malaysia	87	HTN	>50%	61.06 (9.65)		53%	1. Music<br>2. Breathing control	Office attended BP	143/86	2			
Kretzer 2013 (102)	United States	72	Pre-HTN and HTN	>50%	NR (NR)	Minimum 21 years	0%	1. Home BP monitoring<br>2. Psychotherapy	Home BP	129/78	2.25			All women.
Kumar 2017 (103)	India	40	HTN	>50%	NR (NR)	Range 35-60; "Majority of subjects in the experimental group (38.89%) and in the control group (30%) belonged to the age group of 45-49 years"	40%	1. Passive comparator<br>2. Mindfulness	Office attended BP	144/95	2			Specifically recruited participants with diabetes.
Kunikullaya 2015 (104)<br><br>(Related article: Kunikullaya 2016 (105))	India	100	Pre-HTN and HTN	>50%	46.69 (8.46)	Range 30-60	67%	1. Lifestyle<br>2. Lifestyle and relaxation	24 hour ambulatory BP	132/85	3			
LaGrone 1988 (106)	United States	39	HTN	>50%	50.8 (NR)	Range 33-66	17%	1. Passive comparator<br>2. Lifestyle<br>3. Lifestyle and relaxation	Office attended BP	138/89	2			

Landman 2013 (107)	Netherlands	48	HTN	100%	64.45 (8.09)	Minimum 18 years	63%	1. Nonspecific comparator<br>2. Breathing control	Home BP	NR	2			Specifically recruited participants with diabetes.
Latha 1991 (108)	India	22	HTN	100%	NR (NR)	Range 45-70	NR	1. Nonspecific comparator<br>2. Biofeedback	Office attended BP	154/103		6		
Lee 1988 (109)	United States	75	HTN	0%	44 (6)	Range 32-51	NR	1. Lifestyle<br>2. Lifestyle and relaxation	Not reported	135/92	2			
Lee 2003 (110)	Korea	58	HTN	NR	56.25 (6.53)		NR	1. Passive comparator<br>2. Meditative movement	Office attended BP	147/94	2.5			
Li 2024 (111)	China	342	Pre-HTN	0%	49.3 (11.9)	Range 18-65	48.5%	1. Exercise<br>2. Meditative movement	24 hour ambulatory BP	129/82		12		
Lin 2012 (112)	China	43	Pre-HTN	NR	22.3 (NR)	Described as young adults (sophomores)	84%	1. Nonspecific comparator<br>2. Breathing control<br>3. Biofeedback	Office attended BP	131/79	3			
Lin 2022 (113)	China	99	Pre-HTN and HTN	NR	64 (4.21)	Range 58-70	66%	1. Passive comparator<br>2. Meditative movement	Office attended BP	143/85	3			
Linden 2001 (114)	Canada	60	HTN	>50%	54.82 (NR)	Range 28-75	72%	1. Passive comparator<br>2. Psychotherapy	24 hour ambulatory BP	153/97	3			
Logtenberg 2007 (115)	Netherlands	30	HTN	100%	61.85 (6.73)	Minimum 18 years	44%	1. Music<br>2. Breathing control	Home BP	NR	2			Specifically recruited participants with diabetes.
Lokesh 2017 (116)	India	20	HTN	0%	NR (NR)	Range 25-50	NR	1. Exercise<br>2. Exercise and relaxation	Not reported	135/93	1			
Loucks 2023 (117)<br><br>(Related article: Polcari 2022 (118))	United States	201	Pre-HTN and HTN	>50%	59.75 (12.9)	Minimum 18 years, range 22-84	61.3%	1. Home BP monitoring<br>2. Home BP monitoring and relaxation	Office unattended BP	139/82	3	6		
Ma 2018 (119)	China	158	HTN	100%	69 (9.37)	Minimum 60 years	69%	1. Passive comparator<br>2. Meditative movement	Office attended BP	150/90		6		
Manikonda 2008 (120)	Germany	52	Pre-HTN and HTN	0%	NR (NR)	Range 29-71; Intervention group: median 53, range 42-71, Control group: median 52, range 29-69	65%	1. Lifestyle<br>2. Lifestyle and relaxation	NA	NR	NA	NA	NA	
McCaffrey 2005 (121)	Thailand	61	HTN	0%	56.45 (NR)		35%	1. Passive comparator<br>2. Meditative movement	Not reported	160/98	2			
McCraty 2003 (122)	United States	38	HTN	>50%	45.97 (6.55)	Range 35-59	72%	1. Passive comparator<br>2. Psychotherapy	Office attended BP	129/83		3.5		
McGrady 1981 (123)	United States	43	HTN	>50%	49.53 (NR)		32%	1. Nonspecific comparator<br>2. Biofeedback	Office attended BP	143/91	2			Follow-up outcome assessment took place during the last treatment session.
McGrady 1994 (124)	United States	138	HTN	>50%	48.31 (NR)		39%	1. Passive comparator<br>2. Biofeedback	Office attended BP	132/86	2			
Mir 2021 (125)	Malaysia	30	Pre-HTN	0%	21.03 (1.66)	Range 18-25	100%	1. Diet<br>2. Diet and relaxation	Office attended BP	128/74	1			All men, much younger than in most studies.
Misra 2019 (126)	United States	133	HTN	>50%	60.8 (11.5)	Minimum 18 years	52%	1. Passive comparator<br>2. Breathing control	Office attended BP	152/87	2.5			

								3. Breathing control						
Modesti 2010 (127)	Italy	86	HTN	>50%	59.08 (12.21)	Range 40-75	60%	1. Nonspecific comparator<br>2. Music<br>3. Breathing control	Office attended BP	131/78		6		
Modesti 2015 (128)	Italy	45	HTN	0%	52.25 (9.22)	Range 30-75	30%	1. Music<br>2. Breathing control	24 hour ambulatory BP	129/83	2			
Mohammed Elsheikh 2023 (129)	Egypt	60	HTN	100%	65.85 (4.03)	Range 60-75	43%	1. Breathing control<br>2. Breathing control	Not reported	147/94	3			
Mohebbi 2014 (130)<br><br>(Related article: Moghadasi 2021 (131))	Iran	90	HTN	100%	57.99 (8.37)	Range 30-70	42%	1. Nonspecific comparator<br>2. Massage therapy	Office attended BP	141/92	1.5			
Momeni 2016 (132)	Iran	60	HTN	100%	47 (7)	Range 35-60	58%	1. Passive comparator<br>2. Mindfulness	Office BP	129/78	2.25			
Mourya 2009 (133)	India	60	HTN	>50%	NR (NR)	Range 20-60	52%	1. Passive comparator<br>2. Breathing control<br>3. Breathing control	Office attended BP	147/91		3.5		
Murugesan 2000 (134)	India	33	HTN	other (see notes)	NR (NR)	Range 35-65	NR	1. Passive comparator<br>2. Medication<br>3. Meditative movement	Office attended BP	157/108	2.75			High baseline diastolic BP. Presumed to be unmedicated at baseline. One intervention group received medication during the study.
Nakao 1997 (135)	Japan	31	HTN	<50%	56 (8)	Range 35-65	33%	1. Home BP monitoring<br>2. Home BP monitoring and relaxation	Home BP	137/86	1			
Nakao 2000 (136)	Japan	41	HTN	<50%	56.09 (9.15)	Range 30-65	30%	1. Home BP monitoring<br>2. Home BP monitoring and relaxation	Home BP	146/89	1			
Nejati 2015 (137)	Iran	30	HTN	NR	43.4 (5.01)	Range 38-48	53%	1. Passive comparator<br>2. Mindfulness	Not reported	155/90	2	4		
Nolan 2010 (138)	Canada	65	HTN	>50%	55.42 (6.82)	Range 35-64	43%	1. Autogenic training<br>2. Biofeedback	24 hour ambulatory BP	130/80	2.25			
Olsson 2010 (139)	Sweden	19	HTN	>50%	56.55 (8.84)	Range 25-75	39%	1. Passive comparator<br>2. Biofeedback	Office attended BP	152/94	2			
Palomba 2011 (140)	Italy	24	Pre-HTN and HTN	0%	36.18 (9.25)	Range 22-55	86%	1. Home BP monitoring<br>2. Home BP monitoring and relaxation	Home BP	131/82	1			
Pandey 2023 (141)	Canada	60	HTN	0%	63.05 (11.76)	Minimum 18 years	70%	1. Exercise<br>2. Exercise and relaxation	Office attended BP	128/77	3			Low baseline BP.
Pandic 2008 (142)	Sweden	54	HTN	>50%	68.79 (8.68)	Range 35-85	26%	1. Music<br>2. Breathing control	Office attended BP	149/82	3	4		
Park 2014 (143)	Korea	40	Pre-HTN and HTN	0%	NR (NR)	Range 19-65; Intervention group: median =	65%	1. Passive comparator<br>2. Meditative movement	NA	NR	NA	NA	NA	

						52 [IQR 43-61];<br>Control group:<br>median = 54 [IQR<br>45-62]								
Park 2017 (144)	Korea	52	Pre-HTN<br>and HTN	<50%	53.69 (7.74)	Range 19-65	67%	1. Passive comparator<br>2. Meditative movement	Office<br>attended BP	132/85	3	4		
Patel 1975 (145)	United Kingdom	36	HTN	>50%	59.05 (NR)	Range 34-75	38%	1. Nonspecific comparator<br>2. Biofeedback	Office<br>attended BP	168/100	2			High baseline systolic BP.
Patel 1988 (146)	United Kingdom	134	HTN	>50%	NR (NR)	Range 35-64; 20%<br>aged 35-44, 32%<br>aged 45-54 range,<br>48% aged >55<br>years	50%	1. Medication<br>2. Passive comparator<br>3. Nonspecific comparator<br>4. Passive comparator<br>5. Medication and<br>relaxation<br>6. Biofeedback<br>7. Biofeedback<br>8. Biofeedback	Office<br>attended BP	140/87	3	12		
Pathan 2023 (147)	Saudi Arabia	64	HTN	NR	47.8 (6.78)	Range 30-60	72%	1. Passive comparator<br>2. Breathing control<br>3. PMR<br>4. Multicomponent	Office<br>attended BP	151/91	1.13			
Patil 2014 (148)	India	60	HTN	0%	68.93 (5.47)	Range 60-80	100%	1. Nonspecific comparator<br>2. Meditative movement	Office<br>attended BP	146/75	3			All men.
Perez 2009 (149)	Canada	65	HTN	other<br>(see<br>notes)	57.23 (8.94)	Minimum 18 years	48%	1. Medication<br>2. Psychotherapy<br>3. Psychotherapy	24 hour<br>ambulatory<br>BP	148/90	3			Required to undergo<br>washout period from<br>medication at baseline.<br>One intervention group<br>received medication as<br>part of the study.
Plaugher 2002<br>(150)	United States	20	HTN	0%	37.61 (8.29)	Maximum 50 years	43%	1. Nonspecific comparator<br>2. Massage therapy	Office<br>attended BP	151/95	3	4		
Ponte Marquez<br>2019 (151)<br><br>(Related article:<br>Concepcion 2022<br>(152))	Spain	42	Pre-HTN<br>and HTN	>50%	56.5 (7.77)	Range 18 - 70	42.9%	1. Lifestyle<br>2. Mindfulness	Office<br>attended BP	134/87	2	5		
Pukdeesamai<br>2023 (153)	Thailand	70	Pre-HTN	0%	50.16 (5.82)	Range 35-60	86%	1. Passive comparator<br>2. Mindfulness	Office<br>attended BP	135/83	3	5		
Punita 2016 (154)<br><br>(Related article:<br>Pushpanathan<br>2015 (155))	India	80	HTN	100%	43.38 (7.56)	Range 35-55	80%	1. Passive comparator<br>2. Meditative movement	Office BP	126/82	3			
Ranjbar 2007<br>(156)	Iran	220	HTN	100%	55 (NR)		49%	1. Passive comparator<br>2. PMR	Office<br>attended BP	192/104	2			High baseline systolic BP.
Roche 2014 (157)	Spain	50	HTN	100%	57.8 (8.04)	Range 40-71	45%	1. Passive comparator<br>2. Meditative movement	Office<br>attended BP	141/82	3			
Roche 2017 (158)	Spain	100	HTN	100%	57.69 (9.42)	Range 40-70	36%	1. Lifestyle<br>2. Meditation<br>3. Breathing control	Office<br>attended BP	NR	2			

								4. Meditative movement						
Rudy 1995 (159)	United States	46	HTN	0%	NR (NR)	Range 28-60	65%	1. Nonspecific comparator<br>2. Psychotherapy	Not reported	142/94	2			
Saensak 2013 (160)	Thailand	432	HTN	0%	55.95 (5.35)	Range 45-65	0%	1. Lifestyle<br>2. PMR	Office attended BP	148/89	3	4		All women, specifically postmenopausal.
Sangthong 2016 (161)	Thailand	30	HTN	100%	NR (NR)	Range 60-79; median 68 (intervention group), 65 (control group)	21%	1. Home BP monitoring<br>2. Home BP monitoring and relaxation	Home BP	141/82	2	4		
Saptharishi 2009 (162)<br><br>(Related article: Subramanian 2011 (163))	India	120	Pre-HTN and HTN	NR	22.5 (1.3)	Range 20-25	67%	1. Passive comparator<br>2. Exercise<br>3. Diet<br>4. Meditative movement	Office attended BP	126/85	2			Much younger age than in most studies.
Schein 2001 (164)	Israel	65	HTN	>50%	57.14 (8.67)	Range 25-75	47%	1. Music<br>2. Breathing control	Office attended BP	156/95	1	8		
Schein 2009 (165)	Israel	71	HTN	>50%	62.5 (8.46)	Range 40-79	62%	1. Passive comparator<br>2. Breathing control	Office attended BP	149/81	2			Specifically recruited participants with diabetes.
Schneider 1995 (166)<br><br>(Related articles: Alexander 1996 (167), Barnes 1997 (168))	United States	127	HTN	>50%	66.8 (7.67)	Range 55-85	43%	1. Lifestyle<br>2. Meditation<br>3. PMR	Office attended BP	147/92	3			
Schneider 2005 (169)	United States	234	HTN	>50%	48.5 (10.1)		47.3%	1. Lifestyle<br>2. PMR<br>3. Meditation	Office attended BP	143/95	3	12		
Schneider 2019 (170)<br><br>(Related articles: Castillo Richmond 2000 (171), Schneider 2001 (172))	United States	171	Pre-HTN and HTN	>50%	52.77 (10.26)	Range 20-75	35%	1. Lifestyle<br>2. Meditation	Office attended BP	147/85		6		
Schneider 2021 (173)	United States	304	Pre-HTN	0%	42.65 (9.59)	Range 21-75	56%	1. Lifestyle<br>2. Meditation	Office attended BP	126/81			20.37	
Seer 1980 (174)	New Zealand	41	HTN	0%	43.24 (9.84)	Range 20-62	56%	1. Passive comparator<br>2. Meditation<br>3. Meditation	Office attended BP	150/102	3	NA*		*6.25 months follow-up for arms 2 and 3 only, both arms are part of the same intervention for analysis.
Selvam 2020 (175)	India	30	HTN	NR	NR (NR)	Range 40-50	100%	1. Passive comparator<br>2. Meditative movement	Not reported	143/93	3			All men.
Shapiro 1997 (176)	United States	39	HTN	100%	51.19 (8.47)		56%	1. Nonspecific comparator<br>2. Psychotherapy	Daytime ambulatory BP	132/79	2		14	Specific medication changes during the follow-up period, more than

														would be anticipated with usual care.
Shetty 2017 (177)	India	60	HTN	100%	NR (NR)	Range 25-65	NR	1. Nonspecific comparator<br>2. Breathing control	Office attended BP	151/NR	1.07			
Shetty 2022 (178)	India	65	HTN	100%	49.48 (7.93)	Minimum 18 years	52%	1. Passive comparator<br>2. Meditative movement	Office attended BP	143/92	3			
Shou 2019 (179)	China	208	HTN	0%	51.52 (7.58)	Range 18-60	52%	1. Lifestyle<br>2. Meditative movement	Office attended BP	141/83	3			
Singh 2022 (180)	India	238	Pre-HTN	NR	49.79 (10.3)	Minimum 18 years	49.6%	1. Lifestyle<br>2. Lifestyle and relaxation	Not reported	128/83	3	6		
Southam 1982 (181)<br><br>(Related articles: Agras 1983 (182), Agras 1984 (183))	United States	42	HTN	>50%	50.7 (NR)		67%	1. Passive comparator<br>2. PMR	Daytime ambulatory BP and office attended BP	141/93	2		15	Office BP at medium time point, ambulatory BP at short and long time points.
Sujatha 2014 (184)	India	238	HTN	100%	NR (NR)	Range 30-60	46%	1. Passive comparator<br>2. Meditative movement	Office attended BP	153/95	3			
Sun 2015 (185)	China	300	HTN	NR	NR (NR)	Range 45-80. Intervention group: 64% aged 45-64: 36% aged ≥65 Control group: 70% aged 45-64, 30% aged ≥65	18%	1. Nonspecific comparator<br>2. Meditative movement	Not reported	131/82		12		
Supriya 2018 (186)	China	97	Pre-HTN and HTN	NR	57.57 (9.1)	Range 30-80	35%	1. Passive comparator<br>2. Meditative movement	Office attended BP	141/85		12		
Taylor 1977 (187)	United States	40	HTN	100%	48.14 (NR)		74%	1. Passive comparator 2. Nonspecific comparator<br>3. PMR	Office attended BP	146/95	2	7		
Teng 2007 (188)	China	30	HTN	100%	81.4 (8)	Range 63-93	27%	1. Nonspecific comparator<br>2. Music	Home BP	137/60	1			Low baseline diastolic BP.
Thanalakshmi 2020 (189)	India	100	HTN	100%	38.5 (11.48)	Range 18-60	71%	1. Passive comparator<br>2. Breathing control	Office BP	145/88	3			
Thiyagarajan 2015 (190)	India	192	Pre-HTN	0%	43.29 (9.21)	Range 20-60	62%	1. Lifestyle<br>2. Lifestyle and relaxation	Office BP	127/85	3			
Tosi 1992 (191)	United States	44	HTN	0%	47 (NR)		79%	1. Nonspecific comparator<br>2. Psychotherapy<br>3. Hypnosis<br>4. Multicomponent	Office attended BP	143/95	2	4		
Tsai 2003 (192)	Taiwan	88	Pre-HTN and HTN	NR	51.04 (13.29)	Range 35-65	50%	1. Passive comparator<br>2. Meditative movement	Office attended BP	145/87	3			
Tsai 2007 (193)	Taiwan	42	HTN	0%	43.1 (10.9)	Range 20-55	63%	1. Nonspecific comparator<br>2. Biofeedback	Office BP	145/NR	3			
van Montfrans 1990 (194)	Netherlands	42	HTN	0%	41.46 (NR)	Range 24-60	51%	1. Nonspecific comparator<br>2. Multicomponent	Daytime ambulatory BP (with cannulation of the brachial artery)	160/97		12		

Venturelli 2015 (195)	Italy	40	HTN	>50%	67.5 (SE 5.16)	Described as "elderly"	50%	1. Passive comparator<br>2. Exercise<br>3. Exercise<br>4. Breathing control	Office attended BP	149/88	3			
Wadden 1984 (196)<br><br>(Related article: Wadden 1983 (197))	United States	48	HTN	>50%	46.36 (10.41)	Range 21-65	53%	1. Lifestyle and relaxation<br>2. Lifestyle and relaxation<br>3. Psychotherapy	Office attended BP	141/92	2.5	6.5		
Walsh 1977 (198)	United States	24	HTN	>50%	NR (NR)	Range 24-69	63%	1. Biofeedback<br>2. PMR	Not reported	147/94	1.25			
Wang 2010 (199)	China	26	Pre-HTN	0%	52.55 (3.81)	Range 45-60	0%	1. Breathing control<br>2. Biofeedback	Office attended BP	134/82	2	4		All women, specifically postmenopausal.
Wang 2016 (200)	Taiwan	67	Pre-HTN and HTN	0%	45.27 (11.26)	Range 18-64	83%	1. Nonspecific comparator<br>2. Biofeedback	Office BP	142/91	2.25	4		
Webb 2006 (201)	United States	35	HTN	>50%	44.3 (7.9)		0%	1. Nonspecific comparator<br>2. Multicomponent<br>3. Psychotherapy	Office BP	129/82	2.5			All women.
Wen 2021 (202)	China	66	Pre-HTN and HTN	NR	57.49 (9.16)	Range 40-75	60%	1. Meditative movement<br>2. Meditative movement	Office attended BP	136/81	1.5			
Wolff 2016 (203)	Sweden	191	Pre-HTN and HTN	>50%	64.75 (8.42)	Range 34-79	48%	1. Passive comparator<br>2. Meditative movement	Office attended BP	149/88	3			
Wright 2021 (204)	United States	38	HTN	>50%	72.27 (5.2)		18%	1. Passive comparator<br>2. Nonspecific comparator<br>3. Diet and relaxation	Office attended BP	138/77	3			Specifically recruited participants with mild cognitive impairment.
Wu 2023 (205)	China	52	HTN	>50%	63 (7.54)		32%	1. Exercise<br>2. Meditative movement	Office attended BP	151/88	3			
Xiao 2016 (206)	China	48	HTN	NR	65.6 (7.8)		NR	1. Passive comparator<br>2. Meditative movement	Not reported	156/101		6		
Xu 2007 (207)	China	58	Pre-HTN	NR	NR (NR)	Range 19-23	NR	1. Nonspecific comparator<br>2. Biofeedback	Office attended BP	127/76	1	4		Outcome assessment occurred immediately after the intervention at short time point.
Yan 2022 (208)	China	186	HTN	NR	59.87 (6.88)	Range 45-75	35%	1. Passive comparator<br>2. Nonspecific comparator<br>3. Meditative movement	Office attended BP	142/86	3			
Yau 2022 (209)	China	72	Pre-HTN and HTN	>50%	66.9 (9.7)	Minimum 50 years	23.6%	1. Passive comparator<br>2. Diet<br>3. Diet and relaxation	Office attended BP	140/85	1			
Yen 1996 (210)	Taiwan	392	HTN	<50%	53.67 (14.63)	Described as adults, no further information	65%	1. Passive comparator<br>2. Lifestyle<br>3. Nonspecific comparator<br>4. Multicomponent	Home BP	145/88	3			Cluster RCT.
Young 1999 (211)	United States	62	Pre-HTN and HTN	0%	66.7 (5.2)	Range 60-80	21%	1. Exercise<br>2. Meditative movement	Office attended BP	140/76	3			
Yuenyongchaiwat 2024 (212)	Thailand	100	Pre-HTN and HTN	NR	61.49 (5.17)	Range 40-70	11%	1. Nonspecific comparator<br>2. Breathing control	Not reported	139/82	1			
Yung 2001 (213)	China	9	HTN	NR	43 (NR)	Range 31-55	44%	1. PMR<br>2. Autogenic training	Office attended BP	163/97	2			

Zanini 2009 (214)	Brazil	46	HTN	100%	67.1 (9.28)	Minimum 50 years. 69.6% aged ≥ 60 years	42%	1. Passive comparator<br>2. Music	Office BP	148/88	3			
Ziv 2013 (215)	Israel	113	HTN	100%	57 (9)	Range 22-75	49%	1. Multicomponent supplementary<br>2. Multicomponent supplementary and relaxation	24 hour ambulatory BP	131/80		4		
Zurawski 1987 (216)	United States	29	HTN	>50%	46.86 (10.55)	Range 18-60	32%	1. Biofeedback<br>2. Multicomponent	Office BP	137/86	2	6		

HTN, hypertension; NR, not reported; Pre-HTN, pre-hypertension

Model fit statistics

Table S4: NMA model fit statistics for systolic blood pressure at up to 3 months' follow up for individuals with hypertension

Models	Adjusted parameters*	Number of treatments	Number of studies	Number of data points	Residual deviance	pD	DIC	Median tau (95% CrI)	Comments
Primary analysis									
FE	NA	19	54	128	282.2	68.6	350.8	NA	NA
RE consistency - default	NA	19	54	128	124.9	103.6	228.4	4.72 (3.63 to 6.22)	NA
**RE consistency – more iterations**	iter = 11000; warmup = 1000	19	54	128	125.2	104	229.2	4.73 (3.59 to 6.27)	NA
RE inconsistency – high number of iterations	iter = 15000; warmup = 5000	19	54	128	123.9	112.7	236.5	5.17 (3.80 to 7.02)	NA
Sensitivity analysis I: Including high risk of bias studies									
RE consistency	iter = 11000; warmup = 1000	25	139	327	334.8	270.5	605.3	5.51 (4.68 to 6.47)	Ahmadpanah 2016 (19): Multicomponent, Nonspecific comparator (high residual deviance contribution)
Sensitivity analysis II: Hypertension population excluding mixed hypertension and pre-hypertension studies									
RE consistency	iter = 11000; warmup = 1000	18	46	111	110.3	86.8	197.1	4.21 (2.93 to 5.89)	NA
Sensitivity analysis III: Alternative classification of biofeedback interventions									
RE consistency	iter = 11000; warmup = 1000	19	54	128	125.3	103.7	229	4.68 (3.52 to 6.22)	NA
Sensitivity analysis IV: Alternative baseline – follow-up correlation for estimation of missing SDs for change from baseline									
RE consistency	iter = 11000; warmup = 1000	19	54	128	127	108.3	235.5	4.96 (3.86 to 6.43)	NA
Sensitivity analysis V: Excluding imputed outcome data									
RE consistency	iter = 11000; warmup = 1000	19	50	114	112.5	95.5	208	5.08 (3.77 to 6.84)	NA
Subgroup analysis I. Antihypertensive medication status									
RE consistency – none on medication	adapt_delta = 0.99; iter = 11000; warmup = 1000	10	7	19	18.5	17.7	36.2	3.91 (0.29 to 12.64)	6 divergent transitions, 15 transitions exceeding maximum treedepth
RE consistency – some on medication	iter = 11000; warmup = 1000	17	21	53	54.2	46.7	101	6.4 (2.97 to 11.19)	NA
RE consistency – all on medication	adapt_delta = 0.99; iter = 11000; warmup = 1000	9	14	31	30	23.7	53.7	1.54 (0.07 to 5.47)	8 divergent transitions

Models	Adjusted parameters*	Number of treatments	Number of studies	Number of data points	Residual deviance	pD	DIC	Median tau (95% CrI)	Comments
RE consistency – not reported	iter = 11000; warmup = 1000	7	9	18	18.2	17.4	35.7	8.14 (4.23 to 16.26)	NA
Subgroup analysis II. Country level economic resource									
RE consistency – lower income	adapt_delta = 0.99; iter = 11000; warmup = 1000	6	7	14	14	13.8	27.8	6.58 (2.13 to 17.44)	53 transitions exceeding maximum treedepth
RE consistency – higher income	adapt_delta = 0.95; iter = 11000; warmup = 1000	18	47	114	111.6	91.6	203.2	4.78 (3.52 to 6.43)	NA
Subgroup analysis III: Age									
RE consistency – age <75 years	iter = 11000; warmup = 1000	19	52	124	121.3	100.7	222	4.73 (3.56 to 6.27)	NA

*The following parameters were used to fit initial NMA models, referred to as default: intercept prior = 100 and treatment effect prior = 100 (normal distributions), tau prior = 10 (half-normal distribution; NA for FE model), chains = 4, thinning = 1, total number of iterations = 2000, number of warmup iterations = 1000, adapt delta (step size) = 0.95 (RE) or 0.80 (FE); some parameters were adjusted to ensure good model convergence and minimise the number of divergent transitions, and only those adjustments are listed in this column.

Preferred model used to obtain the primary treatment effect estimates is highlighted in bold font. Note that for sensitivity and subgroup analyses only the final RE consistency models are reported.

NMA, network meta-analysis; FE, fixed effects; RE, random effects; iter, total number of iterations per chain; pD, number of effective parameters; DIC, deviance information criterion; tau, between-study heterogeneity; 95% CrI, 95% credible interval.

Table S5: NMA model fit statistics for diastolic blood pressure at up to 3 months' follow up for individuals with hypertension

Models	Adjusted parameters*	Number of treatments	Number of studies	Number of data points	Residual deviance	pD	DIC	Median tau (95% CrI)	Comments
Primary analysis									
FE	NA	19	54	128	282.2	68.6	350.8	NA	NA
RE consistency - default	NA	19	54	128	128.7	100.6	229.3	2.62 (1.84 to 3.58)	Borderline Rhat = 1.01 for tau
**RE consistency – more iterations**	iter = 11000; warmup = 1000	19	54	128	128.6	100.6	229.2	2.62 (1.85 to 3.6)	NA
RE inconsistency – high number of iterations	iter = 15000; warmup = 5000	19	54	128	125	109.2	234.1	2.63 (1.8 to 3.74)	NA
Sensitivity analysis I: Including high risk of bias studies									
RE consistency	iter = 11000; warmup = 1000	25	137	322	334.1	266.7	600.8	3.56 (3 to 4.21)	Ahmanpanah 2016 (19): Nonspecific comparator, Mindfulness; Murugesan 2000 (134): Meditative movement (high residual deviance contributions)
Sensitivity analysis II: Hypertension population excluding mixed hypertension and pre-hypertension studies									
RE consistency	iter = 11000; warmup = 1000	18	46	111	113.9	81.3	195.2	2.04 (1.18 to 3.12)	Patel 1975 (145): Biofeedback (high residual deviance contribution)
Sensitivity analysis III: Alternative classification of biofeedback interventions									
RE consistency	iter = 11000; warmup = 1000	19	54	128	127.4	100	227.4	2.55 (1.79 to 3.5)	NA
Sensitivity analysis IV: Alternative baseline – follow-up correlation for estimation of missing SDs for change from baseline									
RE consistency	iter = 11000; warmup = 1000	19	54	128	130.8	105.6	236.3	2.81 (2.07 to 3.75)	4 transitions exceeding maximum treedepth
Sensitivity analysis V: Excluding imputed outcome data									
RE consistency	iter = 11000; warmup = 1000	19	50	114	115.4	93.6	209	2.9 (2.04 to 4.03)	NA
Subgroup analysis I. Antihypertensive medication status									
RE consistency – none on medication	adapt_delta = 0.99; iter = 11000; warmup = 1000	10	7	19	18.3	17.6	35.8	2.27 (0.12 to 9.7)	53 divergent transitions, 6 transitions exceeding maximum treedepth
RE consistency – some on medication	iter = 11000; warmup = 1000	17	21	53	53.9	47.5	101.4	4.34 (2.3 to 7.49)	NA

Models	Adjusted parameters*	Number of treatments	Number of studies	Number of data points	Residual deviance	pD	DIC	Median tau (95% CrI)	Comments
RE consistency – all on medication	adapt_delta = 0.99; iter = 11000; warmup = 1000	9	14	31	28.9	25	53.9	1.84 (0.23 to 4.49)	NA
RE consistency – not reported	adapt_delta = 0.99; iter = 11000; warmup = 1000	7	9	18	18.8	17.3	36.2	3.76 (1.12 to 9.62)	NA
Subgroup analysis II. Country level economic resource									
RE consistency – lower income	adapt_delta = 0.99; iter = 11000; warmup = 1000	6	7	14	13.1	13	26.1	1.44 (0.06 to 9.78)	187 divergent transitions. 179 transitions exceeding maximum treedepth
RE consistency – higher income	adapt_delta = 0.95; iter = 11000; warmup = 1000	18	47	114	113.4	89.3	202.6	2.75 (1.93 to 3.83)	NA
Subgroup analysis III: Age									
RE consistency – age <75 years	iter = 11000; warmup = 1000	19	52	124	124.4	97.9	222.4	2.68 (1.89 to 3.69)	NA

*The following parameters were used to fit initial NMA models, referred to as default: intercept prior = 100 and treatment effect prior = 100 (normal distributions), tau prior = 10 (half-normal distribution; NA for FE model), chains = 4, thinning = 1, total number of iterations = 2000, number of warmup iterations = 1000, adapt delta (step size) = 0.95 (RE) or 0.80 (FE); some parameters were adjusted to ensure good model convergence and minimise the number of divergent transitions, and only those adjustments are listed in this column.

Preferred model used to obtain the primary treatment effect estimates is highlighted in bold font. Note that for sensitivity and subgroup analyses only the final RE consistency models are reported.

NMA, network meta-analysis; FE, fixed effects; RE, random effects; iter, total number of iterations per chain; pD, number of effective parameters; DIC, deviance information criterion; tau, between-study heterogeneity; 95% CrI, 95% credible interval.

Table S6: NMA model fit statistics for systolic blood pressure at >3 to 12 months' follow up for individuals with hypertension

Models	Adjusted parameters*	Number of treatments	Number of studies	Number of data points	Residual deviance	pD	DIC	Median tau (95% CrI)	Comments
Primary analysis									
FE	NA	15	21	49	70.9	35	105.9	NA	NA
RE consistency - default	NA	15	21	49	50.1	43	93.1	5.14 (2.1 to 9.74)	NA
**RE consistency – more iterations**	iter = 11000; warmup = 1000	15	21	49	49.8	42.9	92.7	5.18 (2.23 to 9.67)	1 divergent transition, 3 transitions exceeding maximum treedepth
RE inconsistency – high number of iterations	adapt_delta = 0.99; iter = 15000; warmup = 5000	15	21	49	48.1	43.4	91.5	2.04 (0.09 to 8.82)	34 divergent transitions, 281 transitions exceeding maximum treedepth;<br>substantial difference in tau;<br>Achmon 1989 (15): Lifestyle fits better under inconsistency model
RE nodesplit - default	NA	NA	NA	NA	NA	NA	NA	NA	19 divergent transitions, 4 transitions exceeding maximum treedepth;<br>direct and indirect evidence inconsistent for comparisons: nonspecific comparator versus biofeedback (median omega = 24.75, 95% CrI 12.57 to 35.95), biofeedback versus lifestyle (median omega = 20.82, 95% CrI 4.64 to 36.28)
Sensitivity analysis I: Including high risk of bias studies									
RE consistency	iter = 11000; warmup = 1000	24	65	161	180.2	141.2	321.4	6.8 (5.33 to 8.63)	Ahmadpanah 2016 (19): Nonspecific comparator; Patel 1988 (146): Passive comparator1&2 (high residual deviance contributions)
Sensitivity analysis II: Hypertension population excluding mixed hypertension and pre-hypertension studies									
RE consistency	adapt_delta = 0.99; iter = 11000; warmup = 1000	14	17	49	42.1	35.5	77.1	4.93 (0.89 to 10.63)	1714 transitions exceeding maximum treedepth
Sensitivity analysis III: Alternative classification of biofeedback interventions									
RE consistency	adapt_delta = 0.99;<br>iter = 11000; warmup = 1000	14	20	47	49.1	39.5	88.6	3.79 (0.58 to 8.25)	279 transitions exceeding maximum treedepth
Sensitivity analysis IV: Alternative baseline – follow-up correlation for estimation of missing SDs for change from baseline									
RE consistency	iter = 11000; warmup = 1000	15	21	49	49.6	43.9	93.6	5.71 (3.15 to 9.99)	4 transitions exceeding maximum treedepth
Sensitivity analysis V: Excluding imputed outcome data									

Models	Adjusted parameters*	Number of treatments	Number of studies	Number of data points	Residual deviance	pD	DIC	Median tau (95% CrI)	Comments
RE consistency	iter = 11000; warmup = 1000	14	18	42	43.1	38.1	81.2	6 (2.72 to 11.59)	32 transitions exceeding maximum treedepth
Post-hoc sensitivity analysis: excluding Achmon *et al* 1989(15)									
RE consistency	adapt_delta = 0.99; iter = 11000; warmup = 1000	15	20	46	44	35.9	79.9	1.35 (0.06 to 5.02)	9650 transitions exceeding maximum treedepth
Subgroup analysis I. Antihypertensive medication status									
RE consistency – some on medication	adapt_delta = 0.99; iter = 11000; warmup = 1000	14	12	28	27.2	26.2	53.4	3.91 (0.19 to 13.5)	6 divergent transitions, 17862 transitions exceeding maximum treedepth
RE consistency – all on medication	adapt_delta = 0.99; iter = 11000; warmup = 1000	5	5	13	14.8	9.9	24.7	2.29 (0.09 to 10.85)	4 divergent transitions; Brauer 1979 (44): PMR(2) (high residual deviance contribution)
Subgroup analysis II. Country level economic resource									
RE consistency – higher income	adapt_delta = 0.95; iter = 11000; warmup=1000	14	19	45	45.8	39.1	85	5.04 (1.76 to 9.92)	NA

*The following parameters were used to fit initial NMA models, referred to as default: intercept prior = 100 and treatment effect prior = 100 (normal distributions), tau prior = 10 (half-normal distribution; NA for FE model), chains = 4, thinning = 1, total number of iterations = 2000, number of warmup iterations = 1000, adapt delta (step size) = 0.95 (RE) or 0.80 (FE); some parameters were adjusted to ensure good model convergence and minimise the number of divergent transitions, and only those adjustments are listed in this column.

Preferred model used to obtain the primary treatment effect estimates is highlighted in bold font. Note that for sensitivity and subgroup analyses only the final RE consistency models are reported.

NMA, network meta-analysis; FE, fixed effects; RE, random effects; iter, total number of iterations per chain; pD, number of effective parameter; DIC, deviance information criterion; tau, between-study heterogeneity; 95% CrI, 95% credible interval.

Table S7: NMA model fit statistics for diastolic blood pressure at >3 to 12 months' follow up for individuals with hypertension

Models	Adjusted parameters*	Number of treatments	Number of studies	Number of data points	Residual deviance	pD	DIC	Median tau (95% CrI)	Comments
Primary analysis									
FE	NA	15	21	49	95.7	35.1	130.8	NA	NA
RE consistency - default	NA	15	21	49	49.2	44.3	93.4	4.04 (2.36 to 7.02)	3961 transitions exceeding maximum treedepth
**RE consistency – more iterations**	iter = 11000; warmup = 1000	15	21	49	49	44	93	4.06 (2.36 to 7.03)	39477 transitions exceeding maximum treedepth
RE inconsistency – high number of iterations	adapt_delta = 0.99; iter = 15000; warmup = 5000	15	21	49	48.8	44.1	92.9	1.67 (0.08 to 5.75)	67 divergent transitions, 33219 transitions exceeding maximum treedepth;<br>substantial reduction in tau
RE nodesplit - default	NA	NA	NA	NA	NA	NA	NA	NA	10 divergent transitions, 2000 – 4000 transitions exceeding maximum treedepth;<br>direct and indirect evidence inconsistent for comparisons: nonspecific comparator versus biofeedback (median omega = 13.01, 95% CrI 2.83 to 22.85), biofeedback versus lifestyle (median omega = 17.00, 95% CrI 9.68 to 24.05)
Sensitivity analysis I: Including high risk of bias studies									
RE consistency	iter = 11000; warmup = 1000	24	65	160	163	142.9	305.9	4.85 (3.9 to 6.05)	Ahmadpanah 2016 (19): Nonspecific comparator (borderline)
Sensitivity analysis II: Hypertension population excluding mixed hypertension and pre-hypertension studies									
RE consistency	adapt_delta = 0.95;<br>iter = 11000; warmup = 1000	14	17	41	42	36.3	78.3	3.6 (1.33 to 7.28)	65 divergent transitions, 32148 transitions exceeding maximum treedepth
Sensitivity analysis III: Alternative classification of biofeedback interventions									
RE consistency	iter = 11000; warmup = 1000	14	20	47	47.1	41.4	88.5	3.53 (2.91 to 6.36)	2 divergent transitions, 30773 transitions exceeding maximum treedepth
Sensitivity analysis IV: Alternative baseline – follow-up correlation for estimation of missing SDs for change from baseline									
RE consistency	iter = 11000; warmup = 1000	15	21	49	50.5	44.9	95.4	4.2 (2.63 to 7.12)	39247 transitions exceeding maximum treedepth; Frankel 1978 (68) Nonspecific comparator, 2 (high residual deviance contribution)
Sensitivity analysis V: Excluding imputed outcome data									

Models	Adjusted parameters*	Number of treatments	Number of studies	Number of data points	Residual deviance	pD	DIC	Median tau (95% CrI)	Comments
RE consistency	iter = 11000; warmup = 1000	14	18	42	42.8	38.7	81.5	4.63 (2.64 to 8.54)	39910 transitions exceeding maximum treedepth
Post-hoc sensitivity analysis: excluding Achmon *et al* 1989(15)									
RE consistency	adapt_delta = 0.99; iter = 11000; warmup = 1000	15	20	46	44.3	36.8	81.1	1.19 (0.07 to 3.53)	10 divergent transitions, 29245 transitions exceeding maximum treedepth
Subgroup analysis I. Antihypertensive medication status									
RE consistency – some on medication	iter = 11000; warmup = 1000	14	12	28	27.9	27.5	55.5	5.92 (2.5 to 14.23)	17 divergent transitions, 39982 transitions exceeding maximum treedepth
RE consistency – all on medication	adapt_delta = 0.99; iter = 11000; warmup = 1000	5	5	13	12.1	10.4	22.5	1.95 (0.09 to 9.33)	19 divergent transitions, 7 transitions exceeding maximum treedepth
Subgroup analysis II. Country level economic resource									
RE consistency – higher income	adapt_delta = 0.95; iter = 11000; warmup = 1000	14	19	45	45.8	39.6	85.4	3.43 (1.51 to 6.57)	NA

*The following parameters were used to fit initial NMA models, referred to as default: intercept prior = 100 and treatment effect prior = 100 (normal distributions), tau prior = 10 (half-normal distribution; NA for FE model), chains = 4, thinning = 1, total number of iterations = 2000, number of warmup iterations = 1000, adapt delta (step size) = 0.95 (RE) or 0.80 (FE); some parameters were adjusted to ensure good model convergence and minimise the number of divergent transitions, and only those adjustments are listed in this column.

Preferred model used to obtain the primary treatment effect estimates is highlighted in bold font. Note that for sensitivity and subgroup analyses only the final RE consistency models are reported.

NMA, network meta-analysis; FE, fixed effects; RE, random effects; iter, total number of iterations per chain; pD, number of effective parameter; DIC, deviance information criterion; tau, between-study heterogeneity; 95% CrI, 95% credible interval.

Table S8: NMA model fit statistics for systolic blood pressure at over 12 months' follow up for individuals with hypertension

Models	Adjusted parameters*	Number of treatments	Number of studies	Number of data points	Residual deviance	pD	DIC	Median tau (95% CrI)	Comments
Primary analysis									
FE	NA	6	3	10	9.2	8.1	17.3	NA	NA
RE consistency - default	NA	6	3	10	9.3	8.5	17.8	3.85 (0.17 to 16.59)	23 divergent transitions;<br>borderline Rhat = 1.01 for tau
**RE consistency – more iterations and smaller step size**	adapt_delta = 0.99; iter = 11000; warmup = 1000	6	3	10	9.4	8.6	17.9	3.84 (0.17 to 16.55)	3 divergent transitions, 8 transitions exceeding maximum treedepth
RE inconsistency – high number of iterations	adapt_delta = 0.99; iter = 15000; warmup = 5000	6	3	10	9.7	9	18.7	6.58 (0.30 to 21.89)	10 divergent transitions, 2 transitions exceeding maximum treedepth
Sensitivity analysis I: Including high risk of bias studies									
RE consistency	adapt_delta = 0.99; iter = 11000; warmup = 1000	9	7	20	20	16.8	3685	3.98 (0.21 to 13.27)	3 divergent transitions,1 transition exceeding maximum treedepth
Sensitivity analysis III: Alternative classification of biofeedback interventions									
RE consistency	adapt_delta = 0.99; iter = 11000; warmup = 1000	5	3	10	10.3	8.2	18.5	3.51 (0.18 to 14.38)	9 divergent transitions
Sensitivity analysis IV: Alternative baseline – follow-up correlation for estimation of missing SDs for change from baseline									
RE consistency	adapt_delta = 0.99; iter = 11000; warmup = 1000	6	3	10	10	8.6	18.6	3.73 (0.15 to 17.03)	35 divergent transitions, 1 transition exceeding maximum treedepth

*The following parameters were used to fit initial NMA models, referred to as default: intercept prior = 100 and treatment effect prior = 100 (normal distributions), tau prior = 10 (half-normal distribution; NA for FE model), chains = 4, thinning = 1, total number of iterations = 2000, number of warmup iterations = 1000, adapt delta (step size) = 0.95 (RE) or 0.80 (FE); some parameters were adjusted to ensure good model convergence and minimise the number of divergent transitions, and only those adjustments are listed in this column.

Preferred model used to obtain the primary treatment effect estimates is highlighted in bold font. Note that for sensitivity analyses only the final RE consistency models are reported. Due to small number of studies, subgroup analyses were not possible at this timepoint; instead, relevant study features are described narratively.

NMA, network meta-analysis; FE, fixed effects; RE, random effects; iter, total number of iterations per chain; pD, number of effective parameter; DIC, deviance information criterion; tau, between-study heterogeneity; 95% CrI, 95% credible interval.

Table S9: NMA model fit statistics for diastolic blood pressure at over 12 months' follow up for individuals with hypertension

Models	Adjusted parameters*	Number of treatments	Number of studies	Number of data points	Residual deviance	pD	DIC	Median tau (95% CrI)	Comments
Primary analysis									
FE	NA	6	3	10	9.7	8.2	17.9	NA	NA
RE consistency - default	NA	6	3	10	9.9	8.4	18.3	2.79 (0.16 to 13.85)	20 divergent transitions
**RE consistency – more iterations and smaller step size**	adapt_delta = 0.99; iter = 11000; warmup = 1000	6	3	10	10	8.6	18.6	3.08 (0.13 to 16.49)	3 divergent transitions, 880 transitions exceeding maximum treedepth
RE inconsistency – high number of iterations	adapt_delta = 0.99; iter = 15000; warmup = 5000	6	3	10	10.4	9	19.3	6.66 (0.35 to 21.9)	7 divergent transitions, 65 transitions exceeding maximum treedepth
Sensitivity analysis I: Including high risk of bias studies									
RE consistency	adapt_delta = 0.99; iter = 11000; warmup = 1000	9	7	20	20	17.6	37.7	4.31 (0.6 to 11.86)	3 divergent transitions
Sensitivity analysis III: Alternative classification of biofeedback interventions									
RE consistency	adapt_delta = 0.99; iter = 11000; warmup = 1000	5	3	10	11	8.9	19.9	4.28 (0.37 to 15)	12 transitions exceeding maximum treedepth
Sensitivity analysis IV: Alternative baseline – follow-up correlation for estimation of missing SDs for change from baseline									
RE consistency	adapt_delta = 0.99; iter = 11000; warmup = 1000	6	3	10	11	8.6	19.6	2.98 (0.12 to 15.88)	29 divergent transitions, 1115 transitions exceeding maximum treedepth

*The following parameters were used to fit initial NMA models, referred to as default: intercept prior = 100 and treatment effect prior = 100 (normal distributions), tau prior = 10 (half-normal distribution; NA for FE model), chains = 4, thinning = 1, total number of iterations = 2000, number of warmup iterations = 1000, adapt delta (step size) = 0.95 (RE) or 0.80 (FE); some parameters were adjusted to ensure good model convergence and minimise the number of divergent transitions, and only those adjustments are listed in this column.

Preferred model used to obtain the primary treatment effect estimates is highlighted in bold font. Note that for sensitivity analyses only the final RE consistency models are reported. Due to small number of studies, subgroup analyses were not possible at this timepoint; instead, relevant study features are described narratively.

NMA, network meta-analysis; FE, fixed effects; RE, random effects; iter, total number of iterations per chain; pD, number of effective parameter; DIC, deviance information criterion; tau, between-study heterogeneity; 95% CrI, 95% credible interval.

Relative effects of relaxation interventions compared to passive comparator

Table S10: Relative effects of relaxation interventions compared to a passive comparator from a primary network meta-analysis and sensitivity and subgroup analyses on systolic and diastolic blood pressure at all time points, in people with hypertension

	**Systolic blood pressure**			**Diastolic blood pressure**		
	**Short follow-up (≤3 months)**	**Medium follow-up (>3 to ≤12 months)**	**Long follow-up (>12 months)**	**Short follow-up (≤3 months)**	**Medium follow-up (>3 to ≤12 months)**	**Long follow-up (>12 months)**
	**MD in mmHg (95% CrI)**	**MD in mmHg (95% CrI)**	**MD in mmHg (95% CrI)**	**MD in mmHg (95% CrI)**	**MD in mmHg (95% CrI)**	**MD in mmHg (95% CrI)**
**Primary analysis**						
Passive comparator	Reference	Reference	Reference	Reference	Reference	Reference
Autogenic training	-3.49 (-11.40 to 4.35)		-6.90 (-21.45 to 8.32)	-2.98 (-7.49 to 1.54)		-5.93 (-19.88 to 7.88)
Biofeedback	-4.39 (-9.68 to 0.89)	-5.57 (-14.80 to 3.84)	3.85 (-9.99 to 17.40)	**-3.71 (-6.87 to -0.50)**	-5.32 (-11.89 to 1.47)	3.18 (-9.95 to 15.59)
Breathing control	**-6.65 (-10.39 to -2.93)**	-0.15 (-11.70 to 11.47)		**-4.30 (-6.47 to -2.11)**	-1.60 (-10.79 to 7.54)	
Hypnosis		-15.27 (-33.05 to 2.37)			-8.04 (-20.48 to 4.55)	
Meditation	**-7.71 (-14.07 to -1.29)**	-0.05 (-12.94 to 13.45)		**-4.80 (-8.66 to -1.06)**	-1.91 (-11.69 to 7.84)	
Meditative movement	**-9.58 (-12.95 to -6.17)**	-6.12 (-12.81 to 0.51)		**-4.57 (-6.57 to -2.58)**	-3.84 (-8.80 to 1.17)	
Mindfulness	**-9.90 (-16.44 to -3.53)**	-8.98 (-26.04 to 9.01)		-3.49 (-7.39 to 0.25)	-8.02 (-22.81 to 6.77)	
Multicomponent	**-6.78 (-11.59 to -1.99)**	-2.17 (-16.43 to 12.17)	-1.21 (-15.71 to 13.20)	**-4.72 (-7.55 to -1.92)**	-4.21 (-14.90 to 6.31)	-0.72 (-13.84 to 12.22)
Music	**-6.61 (-11.62 to -1.56)**	-7.89 (-21.23 to 5.53)		**-3.92 (-6.97 to -0.89)**	-2.82 (-13.35 to 7.66)	
PMR	**-7.46 (-12.15 to -2.96)**	-2.98 (-11.13 to 4.97)	0.49 (-10.49 to 11.37)	**-3.92 (-6.47 to -1.43)**	-1.74 (-7.64 to 3.92)	-0.62 (-10.55 to 9.37)
Psychotherapy	**-9.83 (-16.24 to -3.43)**	-4.16 (-13.94 to 5.36)		**-4.87 (-8.69 to -1.13)**	-4.87 (-11.70 to 2.03)	
**Sensitivity analysis I: Including high risk of bias studies**						
Passive comparator	Reference	Reference	Reference	Reference	Reference	Reference
Autogenic training	**-6.79 (-13.39 to -0.17)**	-6.22 (-16.82 to 4.47)	-6.89 (-19.66 to 6.13)	-4.11 (-8.32 to 0.11)	-4.42 (-11.67 to 2.76)	-5.95 (-17.87 to 5.92)
Biofeedback	**-7.29 (-10.50 to -4.07)**	**-6.24 (-10.88 to -1.54)**	4.11 (-5.48 to 14.66)	**-4.53 (-6.63 to -2.46)**	**-5.02 (-8.25 to -1.78)**	1.83 (-7.40 to 10.70)
Breathing control	**-9.21 (-12.24 to -6.17)**	-4.14 (-11.23 to 3.04)		**-4.92 (-6.94 to -2.89)**	-3.99 (-9.09 to 1.07)	
Hypnosis	**-8.95 (-16.69 to -1.35)**	**-13.25 (-22.26 to -4.24)**	-6.76 (-21.25 to 7.90)	**-6.15 (-10.93 to -1.37)**	**-7.23 (-13.48 to -1.03)**	-9.66 (-22.09 to 2.77)

	Systolic blood pressure			Diastolic blood pressure		
	Short follow-up (≤3 months)	Medium follow-up (>3 to ≤12 months)	Long follow-up (>12 months)	Short follow-up (≤3 months)	Medium follow-up (>3 to ≤12 months)	Long follow-up (>12 months)
	MD in mmHg (95% CrI)	MD in mmHg (95% CrI)	MD in mmHg (95% CrI)	MD in mmHg (95% CrI)	MD in mmHg (95% CrI)	MD in mmHg (95% CrI)
Massage therapy	-3.39 (-12.14 to 5.32)	8.29 (-9.24 to 25.94)		-3.80 (-9.22 to 1.60)	5.41 (-6.83 to 17.85)	
Meditation	**-8.63 (-12.80 to -4.44)**	-0.48 (-8.67 to 7.84)		**-5.02 (-7.65 to -2.39)**	-2.35 (-8.02 to 3.39)	
Meditative movement	**-9.95 (-12.43 to -7.44)**	**-7.65 (-12.46 to -2.88)**		**-5.99 (-7.62 to -4.39)**	**-5.23 (-8.66 to -1.76)**	
Mindfulness	**-11.31 (-16.37 to -6.35)**	**-18.50 (-26.32 to -10.85)**		**-5.91 (-9.24 to -2.67)**	**-12.16 (-17.70 to -6.59)**	
Multicomponent	**-10.68 (-14.58 to -6.82)**	**-8.88 (-15.11 to -2.65)**	-1.51 (-13.38 to 10.99)	**-6.66 (-9.21 to -4.14)**	**-6.66 (-10.93 to -2.40)**	-2.10 (-13.08 to 8.65)
Music	**-9.63 (-14.03 to -5.28)**	-4.99 (-13.46 to 3.54)		**-4.47 (-7.27 to -1.68)**	-1.89 (-7.95 to 4.26)	
PMR	**-7.02 (-10.36 to -3.71)**	-3.16 (-8.45 to 2.22)	-0.79 (-7.99 to 7.02)	**-3.76 (-5.85 to -1.64)**	-3.62 (-7.35 to 0.12)	-3.47 (-10.18 to 3.00)
Psychotherapy	**-9.83 (-13.84 to -5.85)**	-5.25 (-11.42 to 0.94)	-8.73 (-34.55 to 17.77)	**-5.15 (-7.73 to -2.60)**	-2.53 (-6.84 to 1.72)	-3.53 (-22.42 to 15.16)
**Sensitivity analysis II: Hypertension population excluding mixed hypertension and pre-hypertension studies**						
Passive comparator	Reference	Reference		Reference	Reference	
Autogenic training	-3.39 (-10.64 to 3.85)			-3.14 (-7.08 to 0.85)		
Biofeedback	-4.26 (-9.28 to 0.76)	-6.41 (-16.41 to 4.15)		**-3.92 (-6.81 to -1.03)**	-4.94 (-11.78 to 2.16)	
Breathing control	**-6.45 (-10.07 to -2.86)**	-0.44 (-12.34 to 11.49)		**-4.13 (-6.05 to -2.20)**	-1.97 (-10.91 to 6.90)	
Hypnosis		-16.13 (-33.99 to 2.46)			-7.71 (-19.74 to 4.66)	
Meditation	**-8.60 (-16.73 to -0.47)**	2.09 (-11.75 to 16.61)		**-6.71 (-11.44 to -2.20)**	0.13 (-9.61 to 9.71)	
Meditative movement	**-10.37 (-14.55 to -6.23)**	-6.67 (-14.76 to 1.44)		**-4.74 (-7.04 to -2.46)**	-4.58 (-10.19 to 1.13)	
Mindfulness	**-8.31 (-14.96 to -1.71)**	-2.37 (-17.06 to 12.51)		-1.64 (-5.24 to 1.93)	-3.40 (-13.73 to 6.85)	
Multicomponent	**-6.73 (-11.25 to -2.22)**	-7.86 (-21.07 to 5.39)		**-4.71 (-7.23 to -2.29)**	-2.88 (-12.90 to 6.97)	
Music	**-6.41 (-11.25 to -1.61)**	-1.68 (-10.07 to 6.59)		**-3.85 (-6.48 to -1.19)**	-0.54 (-6.50 to 4.90)	
PMR	**-7.29 (-11.90 to -2.91)**	-4.02 (-14.31 to 6.16)		**-4.09 (-6.46 to -1.88)**	-3.97 (-10.87 to 2.90)	

	Systolic blood pressure			Diastolic blood pressure		
	Short follow-up (≤3 months)	Medium follow-up (>3 to ≤12 months)	Long follow-up (>12 months)	Short follow-up (≤3 months)	Medium follow-up (>3 to ≤12 months)	Long follow-up (>12 months)
	**MD in mmHg (95% CrI)**	**MD in mmHg (95% CrI)**	**MD in mmHg (95% CrI)**	**MD in mmHg (95% CrI)**	**MD in mmHg (95% CrI)**	**MD in mmHg (95% CrI)**
Psychotherapy	**-9.73 (-15.74 to -3.65)**	2.09 (-11.75 to 16.61)		**-4.95 (-8.38 to -1.56)**	0.13 (-9.61 to 9.71)	
**Sensitivity analysis III: Alternative classification of biofeedback interventions**						
Passive comparator	Reference	Reference	Reference	Reference	Reference	Reference
Autogenic training	-4.56 (-11.83 to 2.69)		-6.98 (-19.99 to 5.69)	-3.15 (-7.27 to 0.99)		-5.87 (-19.58 to 8.10)
Biofeedback	-4.27 (-11.52 to 3.07)	**-9.01 (-17.28 to -0.27)**		-2.94 (-7.32 to 1.53)	**-7.54 (-13.87 to -0.86)**	
Breathing control	**-6.66 (-10.31 to -3.09)**	0.51 (-7.55 to 8.30)		**-4.31 (-6.41 to -2.22)**	-1.72 (-8.20 to 4.89)	
Meditation	**-9.19 (-15.02 to -3.45)**	-0.84 (-11.28 to 10.52)		**-6.45 (-9.87 to -3.11)**	-2.11 (-10.77 to 6.68)	
Meditative movement	**-9.69 (-13.07 to -6.38)**	**-5.80 (-11.44 to -0.31)**		**-4.64 (-6.64 to -2.70)**	-3.77 (-8.23 to 0.75)	
Mindfulness	**-10.03 (-16.49 to -3.70)**	-10.53 (-25.07 to 5.53)		-3.63 (-7.45 to 0.09)	-8.55 (-22.37 to 5.51)	
Multicomponent	**-6.73 (-11.51 to -1.93)**	-2.82 (-13.49 to 7.86)	0.13 (-10.47 to 10.71)	**-4.61 (-7.41 to -1.89)**	-2.72 (-10.43 to 4.81)	1.64 (-9.00 to 12.45)
Music	**-6.66 (-11.68 to -1.69)**	-7.88 (-18.84 to 3.19)		**-3.94 (-6.83 to -1.00)**	-2.87 (-12.32 to 6.67)	
PMR	**-5.97 (-10.04 to -1.99)**	-2.98 (-9.51 to 3.63)	1.73 (-7.97 to 10.89)	**-3.48 (-5.75 to -1.27)**	-1.64 (-6.82 to 3.42)	0.13 (-9.91 to 9.88)
Psychotherapy	**-10.00 (-16.34 to -3.67)**	-5.28 (-13.28 to 2.83)		**-4.89 (-8.59 to -1.26)**	-5.06 (-11.11 to 1.03)	
**Sensitivity analysis IV: Alternative baseline – follow-up correlation for estimation of missing SDs for change from baseline**						
Passive comparator	Reference	Reference	Reference	Reference	Reference	Reference
Autogenic training	-3.65 (-11.28 to 4.09)		-6.86 (-21.73 to 8.35)	-2.94 (-7.42 to 1.50)		-5.90 (-19.11 to 7.24)
Biofeedback	-4.46 (-9.56 to 0.65)	-5.69 (-15.05 to 3.80)	3.93 (-10.27 to 17.29)	**-3.61 (-6.68 to -0.48)**	-5.35 (-11.77 to 1.24)	3.21 (-8.96 to 15.11)
Breathing control	**-6.71 (-10.56 to -2.92)**	-0.17 (-12.10 to 11.80)		**-4.36 (-6.56 to -2.14)**	-1.58 (-10.43 to 7.45)	
Hypnosis		-15.44 (-32.46 to 1.98)			-8.15 (-20.18 to 4.27)	
Meditation	**-7.87 (-14.07 to -1.75)**	0.53 (-13.29 to 14.55)		**-5.17 (-8.94 to -1.49)**	-1.78 (-12.07 to 8.28)	

	Systolic blood pressure			Diastolic blood pressure		
	Short follow-up (≤3 months)	Medium follow-up (>3 to ≤12 months)	Long follow-up (>12 months)	Short follow-up (≤3 months)	Medium follow-up (>3 to ≤12 months)	Long follow-up (>12 months)
	MD in mmHg (95% CrI)	MD in mmHg (95% CrI)	MD in mmHg (95% CrI)	MD in mmHg (95% CrI)	MD in mmHg (95% CrI)	MD in mmHg (95% CrI)
Meditative movement	**-9.72 (-13.09 to -6.31)**	-6.09 (-12.94 to 0.84)		**-4.75 (-6.79 to -2.75)**	-3.82 (-8.67 to 1.19)	
Mindfulness	**-10.07 (-16.63 to -3.55)**	-7.90 (-25.73 to 10.19)		-3.53 (-7.46 to 0.27)	-7.69 (-22.39 to 6.98)	
Multicomponent	**-6.81 (-11.44 to -2.14)**	-2.26 (-16.26 to 11.56)	-1.21 (-15.51 to 12.70)	**-4.81 (-7.62 to -2.04)**	-4.31 (-14.41 to 5.90)	-0.70 (-13.21 to 11.87)
Music	**-6.68 (-11.91 to -1.45)**	-7.80 (-21.81 to 6.22)		**-3.98 (-7.11 to -0.84)**	-2.89 (-13.61 to 7.90)	
PMR	**-8.19 (-12.77 to -3.80)**	-2.95 (-11.26 to 5.15)	0.65 (-10.37 to 11.31)	**-4.20 (-6.72 to -1.69)**	-1.83 (-7.71 to 3.87)	-0.59 (-10.00 to 8.98)
Psychotherapy	**-9.77 (-16.05 to -3.65)**	-4.16 (-14.19 to 5.84)		**-4.90 (-8.60 to -1.28)**	-4.90 (-11.94 to 2.17)	
**Sensitivity analysis V: Excluding imputed outcome data**						
Passive comparator	Reference	Reference		Reference	Reference	
Autogenic training	-2.01 (-15.15 to 10.90)			-3.18 (-10.88 to 4.69)		
Biofeedback	-3.48 (-10.74 to 3.61)	-6.77 (-18.37 to 4.97)		-3.54 (-8.07 to 1.05)	-4.69 (-12.88 to 3.99)	
Breathing control	**-6.49 (-10.52 to -2.51)**	-0.18 (-13.60 to 13.50)		**-4.26 (-6.65 to -1.89)**	-1.61 (-11.92 to 8.88)	
Meditation	**-7.43 (-14.15 to -0.84)**	0.52 (-15.01 to 16.50)		**-4.84 (-8.91 to -0.82)**	-0.98 (-12.58 to 10.75)	
Meditative movement	**-9.45 (-13.15 to -5.78)**	-6.34 (-14.08 to 1.49)		**-4.59 (-6.80 to -2.40)**	-3.74 (-9.48 to 2.12)	
Mindfulness	**-9.92 (-16.84 to -3.15)**	-8.56 (-27.81 to 11.96)		-3.55 (-7.74 to 0.53)	-7.33 (-23.50 to 9.03)	
Multicomponent	**-6.51 (-12.25 to -0.77)**	-3.01 (-19.31 to 13.28)		**-4.64 (-8.13 to -1.19)**	-3.78 (-15.73 to 8.45)	
Music	**-6.46 (-11.96 to -1.05)**	-7.88 (-22.89 to 7.30)		**-3.89 (-7.16 to -0.57)**	-2.87 (-14.63 to 8.95)	
PMR	**-6.75 (-12.01 to -1.59)**	-2.22 (-12.78 to 8.27)		**-3.85 (-6.72 to -1.00)**	-0.76 (-8.47 to 6.84)	
Psychotherapy	**-8.12 (-16.21 to -0.01)**	-4.80 (-16.12 to 6.35)		-4.55 (-9.33 to 0.19)	-4.49 (-12.61 to 3.83)	
**Subgroup analysis I. Antihypertensive medication status**						
None on medication						
Passive comparator	Reference	Reference	Reference	Reference	Reference	Reference

	Systolic blood pressure			Diastolic blood pressure		
	Short follow-up (≤3 months)	Medium follow-up (>3 to ≤12 months)	Long follow-up (>12 months)	Short follow-up (≤3 months)	Medium follow-up (>3 to ≤12 months)	Long follow-up (>12 months)
	**MD in mmHg (95% CrI)**	**MD in mmHg (95% CrI)**	**MD in mmHg (95% CrI)**	**MD in mmHg (95% CrI)**	**MD in mmHg (95% CrI)**	**MD in mmHg (95% CrI)**
Autogenic training	-6.06 (-18.43 to 6.39)			-4.25 (-12.52 to 4.02)		
Biofeedback	-7.78 (-19.45 to 3.40)			-6.28 (-14.32 to 1.24)		
Breathing control	-7.74 (-26.26 to 10.78)			-11.16 (-23.77 to 1.61)		
Meditation	-6.72 (-20.38 to 6.90)			-9.07 (-18.34 to 0.22)		
Meditative movement	**-18.29 (-34.29 to -1.53)**			-4.40 (-15.57 to 7.30)		
Multicomponent	-8.63 (-18.64 to 1.34)			**-6.91 (-13.76 to -0.35)**		
Music	-0.53 (-22.46 to 21.05)			-7.71 (-22.92 to 7.76)		
Psychotherapy	-10.53 (-23.85 to 2.76)			-6.76 (-15.69 to 2.31)		
Some on medication						
Passive comparator	Reference	Reference		Reference	Reference	
Autogenic training	-0.42 (-18.56 to 17.59)			-1.84 (-13.87 to 10.56)		
Biofeedback	-1.88 (-12.97 to 8.88)	**-20.18 (-35.57 to -2.69)**		-2.16 (-9.58 to 5.40)	-11.90 (-28.25 to 6.60)	
Breathing control	-5.35 (-19.64 to 10.13)			-3.14 (-12.16 to 6.38)		
Hypnosis		**-30.04 (-51.64 to -6.30)**			-14.89 (-37.82 to 9.37)	
Meditation	-11.46 (-28.53 to 5.05)	-2.18 (-19.75 to 17.96)		-5.98 (-16.53 to 4.84)	-0.34 (-20.45 to 21.02)	
Meditative movement	-5.76 (-17.29 to 4.51)	**-30.97 (-54.14 to -6.47)**		-3.50 (-11.18 to 3.41)	-16.53 (-40.75 to 9.74)	
Mindfulness	-7.69 (-20.28 to 4.19)	-12.99 (-31.86 to 8.34)		-3.35 (-11.70 to 4.80)	-8.77 (-31.05 to 14.60)	
Multicomponent	-2.24 (-13.53 to 8.90)	-17.19 (-41.14 to 8.18)		-2.74 (-10.63 to 4.89)	-12.87 (-38.42 to 13.89)	
Music	-7.40 (-19.62 to 5.89)	-7.98 (-21.67 to 5.60)		-3.51 (-11.54 to 4.98)	-3.12 (-19.11 to 12.48)	
PMR	**-10.62 (-20.96 to -0.73)**	-3.22 (-20.82 to 17.03)		-4.62 (-10.54 to 1.31)	1.98 (-18.99 to 24.43)	
Psychotherapy	-2.69 (-21.34 to 15.87)	-9.93 (-21.46 to 2.27)		-0.45 (-12.42 to 11.08)	-6.55 (-19.23 to 7.02)	
All on medication						

	Systolic blood pressure			Diastolic blood pressure		
	**Short follow-up (≤3 months)**	**Medium follow-up (>3 to ≤12 months)**	**Long follow-up (>12 months)**	**Short follow-up (≤3 months)**	**Medium follow-up (>3 to ≤12 months)**	**Long follow-up (>12 months)**
	**MD in mmHg (95% CrI)**	**MD in mmHg (95% CrI)**	**MD in mmHg (95% CrI)**	**MD in mmHg (95% CrI)**	**MD in mmHg (95% CrI)**	**MD in mmHg (95% CrI)**
Passive comparator	Reference	Reference		Reference	Reference	
Biofeedback	5.07 (-8.16 to 18.21)			6.12 (-3.18 to 15.51)		
Breathing control	**-3.76 (-7.12 to -0.70)**	0.67 (-8.92 to 10.33)		-2.66 (-5.50 to 0.27)	-1.60 (-9.60 to 6.76)	
Meditative movement	-4.46 (-9.29 to 0.42)	-4.39 (-11.40 to 2.80)		-2.91 (-6.63 to 1.33)	-3.82 (-9.44 to 2.25)	
Mindfulness	**-13.91 (-20.72 to -7.17)**			-2.57 (-8.30 to 3.20)		
Music	-5.00 (-10.81 to 0.13)			-3.41 (-8.33 to 1.57)		
PMR	-7.76 (-16.25 to 0.72)	-3.00 (-10.97 to 4.26)		-2.77 (-8.72 to 3.20)	-2.06 (-8.48 to 3.22)	
Psychotherapy	**-11.06 (-18.31 to -3.81)**			-5.00 (-10.25 to 0.20)		
Not reported						
Passive comparator	Reference			Reference		
Breathing control	-8.57 (-22.03 to 4.60)			-4.99 (-12.11 to 1.60)		
Meditative movement	**-13.32 (-22.85 to -3.82)**			**-6.75 (-11.80 to -1.58)**		
Multicomponent	-9.27 (-26.41 to 7.30)			-4.54 (-13.73 to 4.01)		
PMR	-7.34 (-21.92 to 6.22)			-3.71 (-11.80 to 3.06)		
**Subgroup analysis II. Country level economic resource**						
Lower income						
Passive comparator	Reference			Reference		
Breathing control	-5.19 (-18.34 to 7.43)			-4.97 (-10.38 to 0.30)		
Meditative movement	-8.57 (-26.09 to 8.66)			-4.18 (-11.42 to 3.20)		
Mindfulness	-8.48 (-21.15 to 3.78)			-1.58 (-7.01 to 3.53)		
Higher income						

	Systolic blood pressure			Diastolic blood pressure		
	**Short follow-up (≤3 months)**	**Medium follow-up (>3 to ≤12 months)**	**Long follow-up (>12 months)**	**Short follow-up (≤3 months)**	**Medium follow-up (>3 to ≤12 months)**	**Long follow-up (>12 months)**
	**MD in mmHg (95% CrI)**	**MD in mmHg (95% CrI)**	**MD in mmHg (95% CrI)**	**MD in mmHg (95% CrI)**	**MD in mmHg (95% CrI)**	**MD in mmHg (95% CrI)**
Passive comparator	Reference	Reference		Reference	Reference	
Autogenic training	-4.13 (-11.97 to 3.69)			-2.90 (-7.59 to 1.80)		
Biofeedback	-5.09 (-10.48 to 0.28)	-4.19 (-13.83 to 5.40)		**-3.60 (-6.93 to -0.27)**	-3.98 (-10.31 to 2.32)	
Breathing control	**-7.38 (-11.80 to -3.01)**	0.00 (-11.76 to 11.75)		**-4.22 (-6.90 to -1.57)**	-1.48 (-9.78 to 6.84)	
Hypnosis		-13.99 (-32.21 to 3.99)			-6.73 (-18.21 to 4.72)	
Meditation	**-8.65 (-15.33 to -1.97)**	2.61 (-11.09 to 16.78)		**-5.70 (-9.76 to -1.74)**	0.48 (-8.76 to 9.45)	
Meditative movement	**-9.33 (-13.15 to -5.60)**	-5.82 (-12.56 to 0.87)		**-5.00 (-7.29 to -2.70)**	-3.58 (-8.02 to 1.07)	
Mindfulness	**-15.72 (-29.88 to -1.62)**	-5.16 (-23.59 to 14.25)		**-10.89 (-19.63 to -2.21)**	-4.69 (-19.21 to 9.62)	
Multicomponent	**-7.23 (-12.11 to -2.36)**	-0.89 (-15.39 to 13.52)		**-4.72 (-7.72 to -1.79)**	-2.79 (-12.76 to 7.04)	
Music	**-7.23 (-12.67 to -1.82)**	-7.87 (-21.12 to 5.57)		**-3.85 (-7.14 to -0.58)**	-2.93 (-12.50 to 6.55)	
PMR	**-8.00 (-12.80 to -3.34)**	-1.49 (-9.98 to 6.63)		**-4.16 (-6.84 to -1.57)**	-0.34 (-6.00 to 4.96)	
Psychotherapy	**-10.38 (-16.81 to -3.83)**	-2.94 (-13.06 to 6.96)		**-4.81 (-8.68 to -0.95)**	-3.51 (-10.11 to 2.96)	
**Subgroup analysis III: Age**						
Age <75 years						
Passive comparator	Reference			Reference		
Autogenic training	-3.78 (-11.56 to 3.95)			-3.06 (-7.67 to 1.58)		
Biofeedback	-4.70 (-10.05 to 0.64)			**-3.81 (-7.07 to -0.49)**		
Breathing control	**-6.61 (-10.39 to -2.82)**			**-4.26 (-6.52 to -2.03)**		
Meditation	**-7.79 (-14.13 to -1.55)**			**-4.85 (-8.67 to -1.05)**		
Meditative movement	**-10.31 (-13.90 to -6.73)**			**-4.84 (-7.01 to -2.71)**		
Mindfulness	**-9.95 (-16.49 to -3.54)**			-3.54 (-7.46 to 0.30)		
Multicomponent	**-6.95 (-11.73 to -2.10)**			**-4.79 (-7.67 to -1.91)**		

	Systolic blood pressure			Diastolic blood pressure		
	Short follow-up (≤3 months)	Medium follow-up (>3 to ≤12 months)	Long follow-up (>12 months)	Short follow-up (≤3 months)	Medium follow-up (>3 to ≤12 months)	Long follow-up (>12 months)
	MD in mmHg (95% CrI)	MD in mmHg (95% CrI)	MD in mmHg (95% CrI)	MD in mmHg (95% CrI)	MD in mmHg (95% CrI)	MD in mmHg (95% CrI)
Music	**-6.30 (-11.66 to -0.89)**			**-3.76 (-7.03 to -0.54)**		
PMR	**-7.57 (-12.26 to -3.04)**			**-3.98 (-6.56 to -1.46)**		
Psychotherapy	**-10.06 (-16.40 to -3.60)**			**-4.97 (-8.83 to -1.14)**		

Note. Relative treatment effects are presented for relaxation (decision set) interventions relative to a passive comparator. Relative effects where 95% credible intervals do not cross 0 (point of no effect) are highlighted in bold font. MD, mean difference; 95% CrI, 95% credible interval; PMR, progressive muscle relaxation.

All relative effects from primary analyses

Table S11: All relative effects from a primary network meta-analysis on systolic blood pressure at short term follow-up (≤3 months) in people with hypertension, expressed as mean differences (95% credible intervals).

Treatment	Passive comparator	Autogenic training	Biofeedback	Breathing control	Diet	Diet + relaxation	Exercise	Exercise + relaxation	Lifestyle
**Autogenic training**	-3.49 (-11.40, 4.35)								
**Biofeedback**	-4.39 (-9.68, 0.89)	-0.89 (-7.98, 6.32)							
**Breathing control**	**-6.65 (-10.39, -2.93)**	-3.16 (-11.57, 5.36)	-2.26 (-8.37, 3.87)						
**Diet**	-2.21 (-12.89, 8.44)	1.28 (-11.90, 14.27)	2.17 (-9.75, 13.98)	4.43 (-6.90, 15.72)					
**Diet + relaxation**	-3.06 (-11.59, 5.45)	0.43 (-10.78, 11.75)	1.32 (-8.39, 11.03)	3.58 (-5.67, 12.81)	-0.85 (-11.95, 10.30)				
**Exercise**	-0.69 (-9.89, 8.16)	2.80 (-8.91, 14.22)	3.70 (-6.38, 13.42)	5.96 (-3.69, 15.23)	1.52 (-12.66, 15.39)	2.38 (-10.15, 14.51)			
**Exercise + relaxation**	-7.65 (-21.36, 5.59)	-4.16 (-19.62, 11.06)	-3.26 (-17.63, 10.68)	-1.00 (-14.97, 12.55)	-5.44 (-23.01, 11.51)	-4.58 (-20.75, 11.00)	-6.96 (-16.87, 2.97)		
**Lifestyle**	-6.46 (-12.95, 0.09)	-2.97 (-13.02, 7.11)	-2.07 (-10.29, 6.13)	0.19 (-6.88, 7.26)	-4.25 (-16.66, 8.22)	-3.40 (-14.03, 7.20)	-5.77 (-16.44, 5.22)	1.19 (-13.25, 16.00)	
**Lifestyle + relaxation**	-5.77 (-15.31, 3.84)	-2.28 (-14.62, 10.10)	-1.38 (-12.32, 9.58)	0.88 (-9.32, 11.20)	-3.55 (-17.76, 10.78)	-2.70 (-15.48, 10.11)	-5.08 (-17.92, 8.35)	1.88 (-14.29, 18.64)	0.69 (-10.87, 12.32)
**Medication**	**-20.16 (-32.22, -8.11)**	**-16.67 (-30.64, -2.65)**	**-15.78 (-28.37, -3.08)**	**-13.51 (-26.00, -1.02)**	**-17.95 (-34.08, -1.81)**	**-17.10 (-31.76, -2.30)**	**-19.47 (-34.00, -4.50)**	-12.51 (-30.06, 5.54)	-13.70 (-27.23, 0.12)
**Meditation**	**-7.71 (-14.07, -1.29)**	-4.21 (-14.09, 5.75)	-3.32 (-11.34, 4.83)	-1.06 (-7.75, 5.61)	-5.49 (-17.98, 7.01)	-4.64 (-15.20, 5.90)	-7.02 (-17.85, 3.95)	-0.06 (-14.76, 14.88)	-1.24 (-7.84, 5.40)
**Meditative movement**	**-9.58 (-12.95, -6.17)**	-6.09 (-14.38, 2.18)	-5.19 (-11.01, 0.64)	-2.93 (-7.60, 1.76)	-7.36 (-18.58, 3.81)	-6.51 (-15.59, 2.54)	-8.89 (-17.71, 0.24)	-1.93 (-15.24, 11.68)	-3.12 (-9.89, 3.65)

**Mindfulness**	**-9.90 (-16.44, -3.53)**	-6.41 (-16.61, 3.74)	-5.52 (-13.89, 2.67)	-3.26 (-10.69, 4.02)	-7.69 (-20.19, 4.82)	-6.84 (-17.50, 3.83)	-9.21 (-19.98, 1.89)	-2.25 (-16.81, 12.71)	-3.44 (-11.20, 4.33)
**Multicomponent**	**-6.78 (-11.59, -1.99)**	-3.29 (-11.58, 5.06)	-2.40 (-8.43, 3.67)	-0.14 (-5.71, 5.48)	-4.57 (-16.24, 7.16)	-3.72 (-13.29, 5.90)	-6.09 (-15.95, 4.05)	0.87 (-12.97, 15.07)	-0.32 (-8.21, 7.55)
**Music**	**-6.61 (-11.62, -1.56)**	-3.12 (-12.11, 5.93)	-2.22 (-9.25, 4.76)	0.04 (-4.26, 4.32)	-4.39 (-16.26, 7.45)	-3.54 (-13.30, 6.35)	-5.92 (-15.74, 4.28)	1.04 (-12.91, 15.38)	-0.15 (-8.05, 7.79)
**Nonspecific comparator**	-1.96 (-5.85, 1.84)	1.53 (-6.13, 9.28)	2.43 (-2.41, 7.18)	4.69 (-0.13, 9.49)	0.26 (-10.88, 11.43)	1.11 (-7.85, 10.11)	-1.27 (-10.13, 7.89)	5.69 (-7.46, 19.35)	4.50 (-2.78, 11.68)
**PMR**	**-7.46 (-12.15, -2.96)**	-3.97 (-12.89, 4.78)	-3.07 (-9.70, 3.43)	-0.81 (-6.29, 4.51)	-5.25 (-16.98, 6.24)	-4.40 (-13.97, 5.09)	-6.77 (-16.43, 3.19)	0.19 (-13.68, 14.32)	-1.00 (-8.11, 5.94)
**Psychotherapy**	**-9.83 (-16.24, -3.43)**	-6.34 (-15.92, 3.22)	-5.45 (-12.96, 2.04)	-3.19 (-10.36, 4.00)	-7.62 (-20.00, 4.79)	-6.77 (-17.27, 3.75)	-9.14 (-19.58, 1.63)	-2.18 (-16.64, 12.51)	-3.37 (-12.28, 5.60)

Table S11 continued

**Treatment**	**Lifestyle + relaxation**	**Medication**	**Meditation**	**Meditative movement**	**Mindfulness**	**Multicomponent**	**Music**	**Nonspecific comparator**	**PMR**
**Medication**	-14.39 (-29.75, 1.00)								
**Meditation**	-1.94 (-13.54, 9.55)	12.46 (-1.12, 26.02)							
**Meditative movement**	-3.81 (-14.06, 6.40)	10.58 (-1.86, 22.86)	-1.87 (-8.79, 5.07)						
**Mindfulness**	-4.14 (-15.85, 7.41)	10.26 (-3.36, 23.80)	-2.20 (-10.77, 6.29)	-0.32 (-7.59, 6.79)					
**Multicomponent**	-1.02 (-11.60, 9.50)	**13.38 (1.02, 25.84)**	0.92 (-6.85, 8.67)	2.80 (-2.88, 8.35)	3.12 (-4.82, 11.28)				
**Music**	-0.84 (-11.70, 10.00)	**13.55 (0.62, 26.54)**	1.10 (-6.50, 8.75)	2.97 (-2.88, 8.79)	3.30 (-4.80, 11.48)	0.17 (-6.51, 6.77)			
**Nonspecific comparator**	3.81 (-6.60, 14.06)	**18.20 (6.15, 30.17)**	5.75 (-1.45, 12.85)	**7.62 (3.36, 11.83)**	**7.95 (0.47, 15.32)**	4.82 (-0.45, 10.04)	4.65 (-1.21, 10.45)		
**PMR**	-1.69 (-12.55, 8.71)	**12.70 (0.01, 25.29)**	0.24 (-6.76, 7.11)	2.12 (-3.36, 7.47)	2.44 (-5.33, 10.10)	-0.68 (-6.84, 5.31)	-0.85 (-7.37, 5.58)	**-5.50 (-10.92, -0.20)**	
**Psychotherapy**	-4.07 (-15.66, 7.40)	**10.33 (0.08, 20.49)**	-2.13 (-10.96, 6.80)	-0.25 (-7.22, 6.67)	0.07 (-8.91, 9.21)	-3.05 (-10.11, 3.99)	-3.22 (-11.08, 4.67)	**-7.87 (-14.16, -1.59)**	-2.37 (-9.93, 5.24)

Note: Negative estimates indicate that the treatment specified in a row is more effective (greater reduction in blood pressure) relative to the treatment specified in a column. Relative effects where 95% credible intervals do not cross 0 (point of no effect) are highlighted in bold font. PMR, progressive muscle relaxation.

Table S12: All relative effects from a primary network meta-analysis on diastolic blood pressure at short term follow-up (≤3 months) in people with hypertension, expressed as mean differences (95% credible intervals).

**Treatment**	**Passive comparator**	**Autogenic training**	**Biofeedback**	**Breathing control**	**Diet**	**Diet + relaxation**	**Exercise**	**Exercise + relaxation**	**Lifestyle**
**Autogenic training**	-2.98 (-7.49, 1.54)								
**Biofeedback**	**-3.71 (-6.87, -0.50)**	-0.73 (-4.79, 3.38)							
**Breathing control**	**-4.30 (-6.47, -2.11)**	-1.32 (-6.21, 3.55)	-0.58 (-4.27, 3.09)						
**Diet**	-0.68 (-6.86, 5.58)	2.30 (-5.38, 9.93)	3.04 (-3.89, 9.92)	3.62 (-2.94, 10.21)					
**Diet + relaxation**	-1.66 (-6.36, 3.07)	1.32 (-5.18, 7.73)	2.06 (-3.52, 7.57)	2.64 (-2.46, 7.76)	-0.98 (-7.21, 5.33)				
**Exercise**	-0.23 (-5.49, 4.82)	2.75 (-4.01, 9.22)	3.48 (-2.40, 9.13)	4.07 (-1.36, 9.30)	0.45 (-7.72, 8.36)	1.43 (-5.60, 8.28)			
**Exercise + relaxation**	-5.23 (-12.91, 2.33)	-2.25 (-11.08, 6.31)	-1.52 (-9.63, 6.41)	-0.93 (-8.78, 6.77)	-4.55 (-14.52, 5.15)	-3.57 (-12.54, 5.17)	-5.00 (-10.60, 0.70)		
**Lifestyle**	-3.44 (-7.23, 0.31)	-0.46 (-6.30, 5.27)	0.27 (-4.61, 5.04)	0.86 (-3.28, 5.01)	-2.76 (-10.06, 4.59)	-1.78 (-7.83, 4.25)	-3.21 (-9.34, 3.06)	1.79 (-6.43, 10.18)	
**Lifestyle + relaxation**	-4.10 (-9.48, 1.31)	-1.12 (-8.16, 5.95)	-0.38 (-6.67, 5.86)	0.20 (-5.64, 6.01)	-3.42 (-11.72, 4.88)	-2.44 (-9.60, 4.74)	-3.87 (-11.19, 3.68)	1.13 (-8.11, 10.54)	-0.66 (-7.20, 5.89)
**Medication**	**-10.77 (-17.77, -3.72)**	-7.79 (-15.93, 0.34)	-7.05 (-14.50, 0.36)	-6.47 (-13.71, 0.81)	**-10.09 (-19.55, -0.80)**	**-9.11 (-17.48, -0.74)**	**-10.53 (-18.94, -1.90)**	-5.54 (-15.66, 4.74)	-7.33 (-15.25, 0.61)
**Meditation**	**-4.80 (-8.66, -1.06)**	-1.82 (-7.66, 3.94)	-1.09 (-5.95, 3.71)	-0.50 (-4.54, 3.39)	-4.12 (-11.40, 3.12)	-3.14 (-9.25, 2.82)	-4.57 (-10.75, 1.69)	0.43 (-7.95, 8.84)	-1.36 (-5.18, 2.42)
**Meditative movement**	**-4.57 (-6.57, -2.58)**	-1.59 (-6.41, 3.15)	-0.86 (-4.41, 2.58)	-0.27 (-3.08, 2.50)	-3.89 (-10.45, 2.56)	-2.91 (-8.00, 2.09)	-4.34 (-9.32, 0.81)	0.66 (-6.91, 8.34)	-1.13 (-5.07, 2.76)
**Mindfulness**	-3.49 (-7.39, 0.25)	-0.51 (-6.47, 5.32)	0.22 (-4.80, 5.09)	0.81 (-3.57, 5.10)	-2.81 (-10.14, 4.37)	-1.83 (-7.92, 4.12)	-3.26 (-9.48, 3.09)	1.74 (-6.61, 10.19)	-0.05 (-4.71, 4.54)
**Multicomponent**	**-4.72 (-7.55, -1.92)**	-1.74 (-6.59, 3.04)	-1.01 (-4.73, 2.68)	-0.43 (-3.71, 2.87)	-4.05 (-10.85, 2.79)	-3.07 (-8.44, 2.31)	-4.49 (-10.09, 1.28)	0.50 (-7.41, 8.56)	-1.28 (-5.91, 3.29)
**Music**	**-3.92 (-6.97, -0.89)**	-0.94 (-6.27, 4.33)	-0.21 (-4.44, 4.02)	0.38 (-2.26, 3.02)	-3.24 (-10.10, 3.63)	-2.26 (-7.77, 3.26)	-3.69 (-9.39, 2.21)	1.31 (-6.75, 9.44)	-0.48 (-5.16, 4.23)
**Nonspecific comparator**	**-2.29 (-4.59, -0.04)**	0.69 (-3.75, 5.05)	1.42 (-1.43, 4.25)	2.01 (-0.87, 4.85)	-1.61 (-8.20, 4.90)	-0.63 (-5.64, 4.34)	-2.06 (-7.03, 3.09)	2.94 (-4.59, 10.59)	1.15 (-3.08, 5.35)
**PMR**	**-3.92 (-6.47, -1.43)**	-0.95 (-6.04, 4.07)	-0.21 (-4.14, 3.58)	0.37 (-2.71, 3.40)	-3.25 (-10.01, 3.41)	-2.27 (-7.62, 2.99)	-3.69 (-9.25, 1.90)	1.30 (-6.57, 9.20)	-0.48 (-4.61, 3.55)
**Psychotherapy**	**-4.87 (-8.69, -1.13)**	-1.90 (-7.42, 3.60)	-1.16 (-5.65, 3.25)	-0.58 (-4.77, 3.62)	-4.20 (-11.47, 3.01)	-3.22 (-9.12, 2.70)	-4.64 (-10.65, 1.56)	0.35 (-7.87, 8.72)	-1.44 (-6.64, 3.81)

Table S12 continued

**Treatment**	**Lifestyle + relaxation**	**Medication**	**Meditation**	**Meditative movement**	**Mindfulness**	**Multicomponent**	**Music**	**Nonspecific comparator**	**PMR**

**Medication**	-6.67 (-15.54, 2.13)								
**Meditation**	-0.70 (-7.43, 5.89)	5.96 (-2.03, 13.85)							
**Meditative movement**	-0.47 (-6.21, 5.27)	6.19 (-1.01, 13.32)	0.23 (-3.84, 4.36)						
**Mindfulness**	0.61 (-6.05, 7.09)	7.28 (-0.74, 15.20)	1.31 (-3.68, 6.35)	1.08 (-3.17, 5.29)					
**Multicomponent**	-0.63 (-6.77, 5.37)	6.04 (-1.17, 13.29)	0.08 (-4.46, 4.69)	-0.15 (-3.46, 3.19)	-1.23 (-5.87, 3.53)				
**Music**	0.18 (-5.98, 6.43)	6.85 (-0.79, 14.45)	0.88 (-3.62, 5.56)	0.65 (-2.81, 4.18)	-0.43 (-5.24, 4.52)	0.80 (-3.13, 4.77)			
**Nonspecific comparator**	1.81 (-4.07, 7.63)	**8.48 (1.47, 15.38)**	2.51 (-1.74, 6.76)	2.28 (-0.23, 4.78)	1.20 (-3.14, 5.59)	2.43 (-0.75, 5.58)	1.63 (-1.90, 5.12)		
**PMR**	0.17 (-5.86, 6.00)	6.84 (-0.55, 14.20)	0.88 (-3.18, 4.90)	0.65 (-2.42, 3.68)	-0.43 (-4.86, 4.01)	0.80 (-2.68, 4.25)	-0.00 (-3.81, 3.73)	-1.63 (-4.72, 1.45)	
**Psychotherapy**	-0.78 (-7.45, 5.79)	5.89 (-0.08, 11.85)	-0.07 (-5.25, 5.20)	-0.30 (-4.42, 3.77)	-1.39 (-6.63, 4.00)	-0.15 (-4.29, 4.00)	-0.96 (-5.68, 3.75)	-2.59 (-6.24, 1.09)	-0.95 (-5.32, 3.45)

Note: Negative estimates indicate that the treatment specified in a row is more effective (greater reduction in blood pressure) relative to the treatment specified in a column. Relative effects where 95% credible intervals do not cross 0 (point of no effect) are highlighted in bold font. PMR, progressive muscle relaxation.

Table S13: All relative effects from a primary network meta-analysis on systolic blood pressure at medium term follow-up (>3 to ≤12 months) in people with hypertension, expressed as mean differences (95% credible intervals).

Treatment	Nonspecific comparator	Biofeedback	Breathing control	Exercise	Hypnosis	Lifestyle	Lifestyle + relaxation	Meditation	Meditative movement
**Biofeedback**	-5.52 (-12.20, 1.16)								
**Breathing control**	-0.10 (-13.83, 13.42)	5.42 (-9.18, 19.75)							
**Exercise**	7.52 (-4.64, 19.44)	13.04 (-0.52, 26.41)	7.62 (-8.65, 23.82)						
**Hypnosis**	-15.23 (-31.92, 1.40)	-9.70 (-24.96, 5.52)	-15.12 (-36.06, 5.92)	**-22.74 (-42.87, -2.46)**					
**Lifestyle**	2.98 (-6.88, 13.70)	8.51 (-1.72, 19.55)	3.08 (-11.34, 18.72)	-4.53 (-18.90, 10.98)	**18.21 (0.07, 36.97)**				
**Lifestyle + relaxation**	-9.96 (-21.03, 1.78)	-4.44 (-16.15, 7.80)	-9.86 (-24.29, 5.48)	**-17.48 (-32.39, -1.75)**	5.27 (-13.89, 24.79)	**-12.94 (-22.87, -3.65)**			
**Meditation**	-0.01 (-13.43, 13.79)	5.52 (-8.46, 19.78)	0.10 (-16.93, 17.78)	-7.52 (-24.52, 10.08)	15.22 (-5.29, 36.28)	-2.99 (-15.36, 8.70)	9.95 (-4.62, 24.37)		
**Meditative movement**	-6.07 (-15.02, 2.61)	-0.55 (-10.77, 9.52)	-5.97 (-17.64, 5.61)	**-13.59 (-25.46, -1.64)**	9.15 (-8.99, 27.43)	-9.06 (-20.94, 1.51)	3.88 (-7.98, 14.86)	-6.07 (-20.69, 7.97)	
**Mindfulness**	-8.93 (-26.40, 9.39)	-3.41 (-21.05, 15.19)	-8.83 (-29.27, 12.69)	-16.44 (-36.88, 5.41)	6.30 (-17.09, 30.34)	-11.91 (-26.50, 2.79)	1.03 (-16.14, 18.82)	-8.92 (-27.31, 10.17)	-2.85 (-20.72, 16.39)
**Multicomponent**	-2.12 (-16.10, 11.70)	3.40 (-9.82, 16.59)	-2.02 (-20.24, 16.27)	-9.64 (-27.41, 8.18)	13.11 (-7.02, 33.20)	-5.10 (-20.91, 9.87)	7.84 (-8.65, 23.76)	-2.12 (-20.50, 15.64)	3.95 (-11.18, 19.13)
**Music**	-7.84 (-23.40, 7.75)	-2.32 (-18.47, 13.87)	-7.74 (-25.53, 9.88)	-15.36 (-33.54, 3.22)	7.39 (-14.62, 29.53)	-10.82 (-27.76, 5.09)	2.12 (-14.64, 18.06)	-7.84 (-26.69, 10.40)	-1.77 (-16.67, 13.28)
**Passive comparator**	0.05 (-8.23, 8.04)	5.57 (-3.84, 14.80)	0.15 (-11.47, 11.70)	-7.47 (-20.16, 5.26)	15.27 (-2.37, 33.05)	-2.93 (-13.07, 6.13)	10.01 (0.00, 19.15)	0.05 (-13.45, 12.94)	6.12 (-0.51, 12.81)
**PMR**	-2.93 (-11.83, 5.68)	2.59 (-7.43, 12.58)	-2.83 (-16.78, 11.13)	-10.45 (-24.31, 3.33)	12.29 (-5.95, 30.64)	-5.92 (-16.01, 2.99)	7.02 (-4.43, 17.59)	-2.93 (-15.12, 8.55)	3.14 (-6.73, 12.95)
**Psychotherapy**	-4.12 (-14.44, 5.91)	1.41 (-8.50, 11.06)	-4.01 (-19.12, 10.70)	-11.63 (-26.55, 3.23)	11.11 (-6.95, 29.04)	-7.10 (-18.94, 3.55)	5.84 (-6.89, 17.65)	-4.11 (-19.34, 10.34)	1.96 (-9.21, 12.97)

Table S13 continued

Treatment	Mindfulness	Multicomponent	Music	Passive comparator	PMR
**Multicomponent**	6.81 (-15.11, 27.70)				
**Music**	1.09 (-21.34, 22.37)	-5.72 (-25.25, 13.98)			
**Passive comparator**	8.98 (-9.01, 26.04)	2.17 (-12.17, 16.43)	7.89 (-5.53, 21.23)		
**PMR**	5.99 (-12.12, 22.74)	-0.81 (-16.04, 14.39)	4.91 (-10.70, 20.32)	-2.98 (-11.13, 4.97)	

**Psychotherapy**	4.81 (-14.09, 22.90)	-2.00 (-14.55, 10.34)	3.72 (-12.75, 20.12)	-4.16 (-13.94, 5.36)	-1.18 (-12.34, 9.87)

Note: Negative estimates indicate that the treatment specified in a row is more effective (greater reduction in blood pressure) relative to the treatment specified in a column. Relative effects where 95% credible intervals do not cross 0 (point of no effect) are highlighted in bold font. PMR, progressive muscle relaxation.

Table S14: All relative effects from a primary network meta-analysis on diastolic blood pressure at medium term follow-up (>3 to ≤12 months) in people with hypertension, expressed as mean differences (95% credible intervals).

Treatment	Nonspecific comparator	Biofeedback	Breathing control	Exercise	Hypnosis	Lifestyle	Lifestyle + relaxation	Meditation	Meditative movement
**Biofeedback**	-4.40 (-9.04, 0.36)								
**Breathing control**	-0.68 (-11.37, 9.66)	3.72 (-7.33, 14.50)							
**Exercise**	0.29 (-8.57, 9.08)	4.68 (-5.11, 14.43)	0.97 (-11.47, 13.43)						
**Hypnosis**	-7.12 (-18.80, 4.48)	-2.72 (-13.40, 7.95)	-6.44 (-21.72, 8.91)	-7.40 (-21.83, 7.29)					
**Lifestyle**	3.02 (-4.04, 10.17)	7.41 (0.30, 14.57)	3.70 (-7.44, 15.05)	2.73 (-7.97, 13.52)	10.13 (-2.72, 22.86)				
**Lifestyle + relaxation**	-5.19 (-13.51, 3.19)	-0.79 (-9.42, 7.93)	-4.51 (-15.78, 7.11)	-5.48 (-16.77, 6.00)	1.93 (-11.84, 15.70)	**-8.20 (-15.35, -0.99)**			
**Meditation**	-0.98 (-11.01, 8.87)	3.41 (-7.00, 13.55)	-0.30 (-13.50, 12.98)	-1.27 (-14.02, 11.38)	6.13 (-8.80, 20.64)	-4.00 (-13.05, 4.80)	4.21 (-6.76, 14.85)		
**Meditative movement**	-2.92 (-9.42, 3.43)	1.48 (-5.89, 8.73)	-2.24 (-11.56, 7.05)	-3.21 (-12.09, 5.57)	4.19 (-8.83, 17.13)	-5.94 (-13.91, 2.04)	2.27 (-6.24, 10.62)	-1.94 (-12.43, 8.71)	
**Mindfulness**	-7.10 (-22.19, 7.79)	-2.70 (-17.75, 12.25)	-6.42 (-23.61, 10.80)	-7.38 (-24.29, 9.66)	0.02 (-18.36, 18.32)	-10.11 (-23.36, 3.04)	-1.91 (-16.91, 13.14)	-6.11 (-21.96, 9.68)	-4.18 (-19.52, 11.27)
**Multicomponent**	-3.28 (-13.90, 7.02)	1.12 (-8.99, 10.94)	-2.60 (-16.40, 11.11)	-3.57 (-16.86, 9.55)	3.83 (-10.80, 18.46)	-6.30 (-17.43, 4.41)	1.91 (-10.30, 13.76)	-2.30 (-15.66, 10.98)	-0.36 (-11.54, 10.69)
**Music**	-1.90 (-13.95, 10.02)	2.50 (-9.91, 14.77)	-1.22 (-14.87, 12.63)	-2.18 (-16.28, 11.81)	5.22 (-11.28, 21.49)	-4.91 (-17.52, 7.53)	3.29 (-9.51, 15.96)	-0.91 (-15.22, 13.63)	1.03 (-10.69, 12.52)
**Passive comparator**	0.92 (-5.07, 6.69)	5.32 (-1.47, 11.89)	1.60 (-7.54, 10.79)	0.64 (-8.74, 9.93)	8.04 (-4.55, 20.48)	-2.09 (-8.94, 4.62)	6.11 (-1.19, 13.02)	1.91 (-7.84, 11.69)	3.84 (-1.17, 8.80)
**PMR**	-0.82 (-7.02, 5.06)	3.58 (-3.55, 10.34)	-0.14 (-10.89, 10.49)	-1.11 (-11.27, 8.85)	6.29 (-6.51, 18.78)	-3.84 (-10.75, 2.81)	4.37 (-4.16, 12.26)	0.16 (-8.68, 8.85)	2.10 (-5.10, 9.02)
**Psychotherapy**	-3.95 (-11.37, 3.44)	0.45 (-6.78, 7.50)	-3.27 (-14.44, 8.08)	-4.24 (-15.15, 6.53)	3.17 (-9.61, 15.91)	-6.97 (-14.58, 0.48)	1.24 (-7.85, 10.03)	-2.97 (-13.59, 7.77)	-1.03 (-9.01, 6.87)

Table S14 continued

Treatment	Mindfulness	Multicomponent	Music	Passive comparator	PMR
**Multicomponent**	3.82 (-13.48, 20.74)				
**Music**	5.20 (-12.98, 23.28)	1.39 (-13.57, 16.40)			
**Passive comparator**	8.02 (-6.77, 22.81)	4.21 (-6.31, 14.90)	2.82 (-7.66, 13.35)		
**PMR**	6.28 (-8.74, 21.04)	2.46 (-8.61, 13.44)	1.08 (-11.07, 13.00)	-1.74 (-7.64, 3.92)	

**Psychotherapy**	3.15 (-12.04, 18.30)	-0.67 (-9.53, 8.38)	-2.05 (-14.51, 10.50)	-4.87 (-11.70, 2.03)	-3.13 (-10.84, 4.81)

Note: Negative estimates indicate that the treatment specified in a row is more effective (greater reduction in blood pressure) relative to the treatment specified in a column. Relative effects where 95% credible intervals do not cross 0 (point of no effect) are highlighted in bold font. PMR, progressive muscle relaxation.

Table S15: All relative effects from a primary network meta-analysis on systolic blood pressure at long term follow-up (>12 months) in people with hypertension, expressed as mean differences (95% credible intervals).

**Treatment**	**Biofeedback**	**Autogenic training**	**Lifestyle + relaxation**	**Multicomponent**	**Passive comparator**
**Autogenic training**	-10.75 (-30.08, 9.65)				
**Lifestyle + relaxation**	-0.41 (-15.61, 15.22)	10.34 (-10.61, 30.64)			
**Multicomponent**	-5.06 (-20.09, 9.98)	5.69 (-15.50, 26.26)	-4.65 (-20.55, 11.16)		
**Passive comparator**	-3.85 (-17.40, 9.99)	6.90 (-8.32, 21.45)	-3.44 (-17.86, 11.13)	1.21 (-13.20, 15.71)	
**PMR**	-3.36 (-17.30, 10.82)	7.39 (-11.44, 25.69)	-2.95 (-17.72, 11.67)	1.70 (-12.84, 16.30)	0.49 (-10.49, 11.37)

Note: Negative estimates indicate that the treatment specified in a row is more effective (greater reduction in blood pressure) relative to the treatment specified in a column. Relative effects where 95% credible intervals do not cross 0 (point of no effect) are highlighted in bold font. PMR, progressive muscle relaxation.

Table S16: All relative effects from a primary network meta-analysis on diastolic blood pressure at long term follow-up (>12 months) in people with hypertension, expressed as mean differences (95% credible intervals).

**Treatment**	**Biofeedback**	**Autogenic training**	**Lifestyle + relaxation**	**Multicomponent**	**Passive comparator**
**Autogenic training**	-9.11 (-27.23, 9.71)				
**Lifestyle + relaxation**	-3.80 (-17.27, 10.12)	5.31 (-13.38, 23.87)			
**Multicomponent**	-3.90 (-17.51, 9.88)	5.21 (-13.80, 23.87)	-0.10 (-14.46, 13.69)		
**Passive comparator**	-3.18 (-15.59, 9.95)	5.93 (-7.88, 19.88)	0.62 (-12.03, 13.19)	0.72 (-12.22, 13.84)	

**PMR**	-3.80 (-16.09, 8.99)	5.32 (-11.46, 21.88)	0.01 (-13.04, 12.93)	0.11 (-13.05, 13.32)	-0.62 (-10.55, 9.37)

Note: Negative estimates indicate that the treatment specified in a row is more effective (greater reduction in blood pressure) relative to the treatment specified in a column. Relative effects where 95% credible intervals do not cross 0 (point of no effect) are highlighted in bold font. PMR, progressive muscle relaxation.

Hypertension: Short term follow-up (up to 3 months)

Disconnected studies

Two studies contributing to the same comparison (home BP monitoring and relaxation versus home BP monitoring alone (117, 140)) were disconnected from the main networks of systolic and diastolic BP evidence, and were therefore excluded from the NMA. Both of these studies indicated there may be a small benefit of relaxation plus home BP monitoring when compared to home BP monitoring alone for systolic BP and diastolic BP. However, the wide credible intervals mean that we are uncertain if this benefit represents an important impact on blood pressure, and the certainty of the evidence was rated as very low (systolic BP: Loucks *et al* 2023 (117) mean difference [MD] = -2.8mmHg, 95% CI -7.1 to 1.5; Palomba *et al* 2011 (140) MD = -5.43mmHg, 95% CI -11.76 to 0.9; diastolic BP: Loucks *et al* 2023 (117) MD = -1.1mmHg, 95% CI -3.7 to 1.4; Palomba *et al* 2011 (140) MD = -2.36mmHg, 95% CI -6.62 to 1.9).

Network meta-analysis

The most frequent direct comparisons in the network were between a passive comparator and PMR (n = 8), meditative movement (n = 7), breathing control (n = 6), multicomponent intervention (n = 4), or a nonspecific comparator (n = 4); between biofeedback and a nonspecific comparator (n = 6); and between breathing control and music (n = 5). Three trials or fewer contributed direct evidence to each of the remaining comparisons.

Inspection of model fit indicated good convergence (R-hat = 1 for all comparisons) for both outcomes. Model comparisons favoured random-effects models assuming consistency between direct and indirect evidence over fixed-effect models, and over inconsistency models, as indicated by lower DIC values (see Table S4, Table S5). Median between-study heterogeneity was estimated as 4.73mmHg (95% CrI 3.59 to 6.27) for systolic BP, and 2.62mmHg (95% CrI 1.85 to 3.6) for diastolic BP models.

Assessment of the certainty of the evidence

We used CINeMA to assess the certainty of the evidence. The assessments for each comparison are shown below.

Figure S1: CINeMA assessments: Hypertension, short-term follow-up, systolic BP

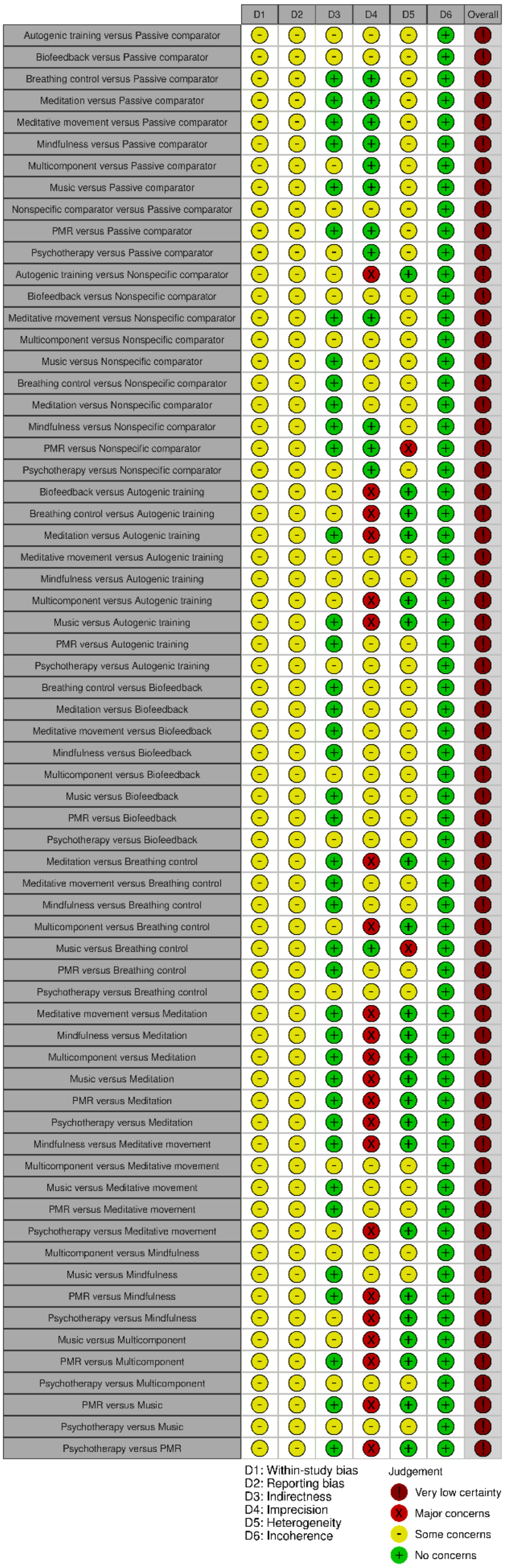

Figure S2: CINeMA assessments: Hypertension, short-term follow-up, diastolic BP

	D1	D2	D3	D4	D5	D6	Overall
Autogenic training versus Passive comparator	-	-	-	-	-	+	!
Biofeedback versus Passive comparator	-	-	-	+	-	+	!
Breathing control versus Passive comparator	-	-	+	+	-	+	!
Meditation versus Passive comparator	-	-	+	+	-	+	!
Meditative movement versus Passive comparator	-	-	+	+	-	+	!
Mindfulness versus Passive comparator	-	-	+	-	-	+	!
Multicomponent versus Passive comparator	-	-	-	+	-	+	!
Music versus Passive comparator	-	-	+	+	-	+	!
Nonspecific comparator versus Passive comparator	-	-	-	+	X	+	!
PMR versus Passive comparator	-	-	+	+	-	+	!
Psychotherapy versus Passive comparator	-	-	-	+	-	+	!
Autogenic training versus Nonspecific comparator	-	-	-	X	+	+	!
Biofeedback versus Nonspecific comparator	-	-	+	-	-	+	!
Meditative movement versus Nonspecific comparator	-	-	+	-	-	+	!
Multicomponent versus Nonspecific comparator	-	-	-	-	-	+	!
Music versus Nonspecific comparator	-	-	+	-	-	+	!
Breathing control versus Nonspecific comparator	-	-	+	-	-	+	!
Meditation versus Nonspecific comparator	-	-	+	-	-	+	!
Mindfulness versus Nonspecific comparator	-	-	-	X	+	+	!
PMR versus Nonspecific comparator	-	-	+	-	-	+	!
Psychotherapy versus Nonspecific comparator	-	-	-	-	-	+	!
Biofeedback versus Autogenic training	-	-	-	X	+	+	!
Breathing control versus Autogenic training	-	-	-	X	+	+	!
Meditation versus Autogenic training	-	-	+	X	+	+	!
Meditative movement versus Autogenic training	-	-	-	X	+	+	!
Mindfulness versus Autogenic training	-	-	-	X	+	+	!
Multicomponent versus Autogenic training	-	-	-	X	+	+	!
Music versus Autogenic training	-	-	-	X	+	+	!
PMR versus Autogenic training	-	-	+	X	+	+	!
Psychotherapy versus Autogenic training	-	-	-	X	+	+	!
Breathing control versus Biofeedback	-	-	+	X	+	+	!
Meditation versus Biofeedback	-	-	+	X	+	+	!
Meditative movement versus Biofeedback	-	-	+	-	-	+	!
Mindfulness versus Biofeedback	-	-	+	X	+	+	!
Multicomponent versus Biofeedback	-	-	-	-	-	+	!
Music versus Biofeedback	-	-	+	X	+	+	!
PMR versus Biofeedback	-	-	+	X	+	+	!
Psychotherapy versus Biofeedback	-	-	-	X	+	+	!
Meditation versus Breathing control	-	-	+	X	+	+	!
Meditative movement versus Breathing control	-	-	+	-	-	+	!
Mindfulness versus Breathing control	-	-	+	X	+	+	!
Multicomponent versus Breathing control	-	-	-	-	-	+	!
Music versus Breathing control	-	-	+	-	-	+	!
PMR versus Breathing control	-	-	+	-	-	+	!
Psychotherapy versus Breathing control	-	-	-	X	+	+	!
Meditative movement versus Meditation	-	-	+	X	+	+	!
Mindfulness versus Meditation	-	-	+	X	+	+	!
Multicomponent versus Meditation	-	-	+	X	+	+	!
Music versus Meditation	-	-	+	X	+	+	!
PMR versus Meditation	-	-	+	X	+	+	!
Psychotherapy versus Meditation	-	-	+	X	+	+	!
Mindfulness versus Meditative movement	-	-	+	X	+	+	!
Multicomponent versus Meditative movement	-	-	-	X	+	+	!
Music versus Meditative movement	-	-	+	-	-	+	!
PMR versus Meditative movement	-	-	+	-	-	+	!
Psychotherapy versus Meditative movement	-	-	-	X	+	+	!
Multicomponent versus Mindfulness	-	-	-	X	+	+	!
Music versus Mindfulness	-	-	+	X	+	+	!
PMR versus Mindfulness	-	-	+	X	+	+	!
Psychotherapy versus Mindfulness	-	-	-	X	+	+	!
Music versus Multicomponent	-	-	-	X	+	+	!
PMR versus Multicomponent	-	-	-	-	-	+	!
Psychotherapy versus Multicomponent	-	-	-	X	+	+	!
PMR versus Music	-	-	+	X	+	+	!
Psychotherapy versus Music	-	-	-	X	+	+	!
Psychotherapy versus PMR	-	-	-	X	+	+	!

D1: Within-study bias
D2: Reporting bias
D3: Indirectness
D4: Imprecision
D5: Heterogeneity
D6: Incoherence

Judgement
- ! Very low certainty
- X Major concerns
- \- Some concerns
- \+ No concerns

Hypertension: Medium term follow-up (>3 to 12 months)

Disconnected studies

Two studies were disconnected from the main networks of both systolic and diastolic BP evidence, and were excluded from the NMA (117, 215). Loucks *et al* 2023 (117) compared home BP monitoring combined with relaxation versus home BP monitoring alone, reporting a small difference in systolic BP, with a point estimate that did not reach the MID of 5mmHg (MD = -4.5mmHg, 95% CI -9 to -0.1; low-certainty evidence) in favour of the combined intervention, and a trivial difference in diastolic BP (MD = 0.3mmHg, 95% CI -2.5 to 3.1; very low-certainty evidence). Ziv *et al* 2013 (215) compared relaxation plus a multicomponent supplementary intervention with the supplementary intervention alone, and reported trivial differences in systolic BP (MD = -0.3mmHg, 95% CI -4.45 to 3.85; low-certainty evidence) and diastolic BP (MD = -1.2mmHg, 95% CI -3.96 to 1.56; very low-certainty evidence).

Network meta-analysis

The most frequent direct comparison in the network was between a nonspecific comparator and biofeedback (n = 5). Three trials or fewer contributed direct evidence to each of the remaining comparisons.

Random-effects NMA models were fitted for systolic and diastolic BP at >3 to 12 months' follow-up. R-hat values for all estimates indicated good convergence for both outcomes. Model comparisons favoured random-effects models assuming consistency between direct and indirect evidence over fixed-effect models, and there were no meaningful differences in model fit with inconsistency models (Table S6, Table S7). However, there was a reduction in median between-study heterogeneity in inconsistency models relative to consistency models (tau = 5.18mmHg, 95% CrI 2.23 to 9.67 for systolic BP, tau = 4.06mmHg, 95% CrI 2.36 to 7.03 for diastolic BP) suggesting that inconsistency was present. Upon further investigation the lifestyle intervention arm in Achmon *et al* 1989 (15) was found to fit better under an inconsistency model for systolic BP. This study was characterised by high variance of effect estimates for systolic BP, and exceptionally large changes in systolic and diastolic blood pressure from baseline in both active intervention arms (psychotherapy and biofeedback). We did not identify any data extraction or conversion errors, there were no differences in participant characteristics between intervention arms, and no apparent differences in study-level characteristics compared to other trials included in the analysis. We did note that the follow-up assessment in Achmon *et al* 1989 (15) was conducted at the end of the intervention period. This contrasts with the majority of studies contributing to these networks, where participants had already discontinued the intervention at the time of outcome assessment. Furthermore, a node-splitting approach indicated discrepancy between the direct and indirect evidence in comparisons between lifestyle interventions versus biofeedback (direct evidence suggesting a beneficial effect of biofeedback, indirect evidence suggesting no effect), and biofeedback versus a nonspecific comparator (direct evidence suggesting no effect of biofeedback, and more uncertain indirect evidence suggesting a beneficial effect; note that the overall NMA estimate was aligned with the direct evidence). Our approach to pooling different active comparator arms into the nonspecific comparator node may account for some extent of this inconsistency, because most direct evidence for this comparison comes from trials comparing biofeedback to sham biofeedback, while indirect evidence may include other comparators such as office BP monitoring, attention control, and nonspecific therapy.

As the inconsistency in the above NMA models appeared to be driven by Achmon *et al* 1989 (15), we examined the impact of excluding this study in a post-hoc sensitivity analysis (see Table S17). This approach substantially reduced the estimates of between-study heterogeneity and resulted in a better fit of consistency rather than inconsistency models (DIC difference >5 points) for both systolic and diastolic BP outcomes. Following this sensitivity analysis, the point estimates for biofeedback changed substantially, such that it no longer appeared to cause a reduction in systolic or diastolic BP. The effect of meditative movement also became smaller, whilst that of mindfulness and music became slightly larger. Multicomponent interventions appeared to have less of an impact on systolic BP, but a slightly larger impact on diastolic BP. Conversely, psychotherapy had a slightly larger impact on systolic BP, but a smaller effect on diastolic BP.

Assessment of the certainty of the evidence

We used CINeMA to assess the certainty of the evidence. The assessments for each comparison are shown below.

Figure S3: CINeMA assessments: Hypertension, medium-term follow-up, systolic BP

	D1	D2	D3	D4	D5	D6	Overall
Breathing control versus Passive comparator	-	-	-	X	+	+	!
Meditative movement versus Passive comparator	-	-	+	-	-	+	!
Music versus Passive comparator	-	-	+	X	+	X	!
Nonspecific comparator versus Passive comparator	-	-	+	X	+	+	!
Biofeedback versus Passive comparator	-	-	+	-	-	X	!
Hypnosis versus Passive comparator	-	-	+	-	-	X	!
Meditation versus Passive comparator	-	-	+	X	+	X	!
Mindfulness versus Passive comparator	-	-	+	X	+	X	!
Multicomponent versus Passive comparator	-	-	+	X	+	X	!
PMR versus Passive comparator	-	-	+	X	-	+	!
Psychotherapy versus Passive comparator	-	-	+	X	+	+	!
Biofeedback versus Nonspecific comparator	-	-	+	-	-	-	!
Meditative movement versus Nonspecific comparator	-	-	+	-	-	-	!
Breathing control versus Nonspecific comparator	-	-	+	X	+	X	!
Hypnosis versus Nonspecific comparator	-	-	+	-	-	X	!
Meditation versus Nonspecific comparator	-	-	+	X	+	X	!
Mindfulness versus Nonspecific comparator	-	-	+	X	+	X	!
Multicomponent versus Nonspecific comparator	-	-	+	X	+	X	!
Music versus Nonspecific comparator	-	-	+	X	+	X	!
PMR versus Nonspecific comparator	-	-	+	X	+	+	!
Psychotherapy versus Nonspecific comparator	-	-	+	X	-	X	!
Breathing control versus Biofeedback	-	-	+	X	+	X	!
Hypnosis versus Biofeedback	-	-	-	X	+	X	!
Meditation versus Biofeedback	-	-	+	X	+	X	!
Meditative movement versus Biofeedback	-	-	+	X	+	X	!
Mindfulness versus Biofeedback	-	-	+	X	+	X	!
Multicomponent versus Biofeedback	-	-	+	X	+	+	!
Music versus Biofeedback	-	-	+	X	+	X	!
PMR versus Biofeedback	-	-	+	X	+	X	!
Psychotherapy versus Biofeedback	-	-	+	X	+	+	!
Hypnosis versus Breathing control	-	-	+	X	+	X	!
Meditation versus Breathing control	-	-	+	X	+	X	!
Meditative movement versus Breathing control	-	-	-	X	+	+	!
Mindfulness versus Breathing control	-	-	+	X	+	X	!
Multicomponent versus Breathing control	-	-	+	X	+	X	!
Music versus Breathing control	-	-	+	X	+	X	!
PMR versus Breathing control	-	-	+	X	+	X	!
Psychotherapy versus Breathing control	-	-	+	X	+	X	!
Meditation versus Hypnosis	-	-	+	X	+	X	!
Meditative movement versus Hypnosis	-	-	+	X	+	X	!
Mindfulness versus Hypnosis	-	-	+	X	+	X	!
Multicomponent versus Hypnosis	-	-	-	X	+	X	!
Music versus Hypnosis	-	-	+	X	+	X	!
PMR versus Hypnosis	-	-	+	X	+	X	!
Psychotherapy versus Hypnosis	-	-	+	X	+	X	!
Meditative movement versus Meditation	-	-	+	X	+	X	!
Mindfulness versus Meditation	-	-	+	X	+	X	!
Multicomponent versus Meditation	-	-	+	X	+	X	!
Music versus Meditation	-	-	+	X	+	X	!
PMR versus Meditation	-	-	+	X	+	+	!
Psychotherapy versus Meditation	-	-	+	X	+	X	!
Mindfulness versus Meditative movement	-	-	+	X	+	X	!
Multicomponent versus Meditative movement	-	-	+	X	+	X	!
Music versus Meditative movement	-	-	+	X	+	X	!
PMR versus Meditative movement	-	-	+	X	+	X	!
Psychotherapy versus Meditative movement	-	-	+	X	+	X	!
Multicomponent versus Mindfulness	-	-	+	X	+	X	!
Music versus Mindfulness	-	-	+	X	+	X	!
PMR versus Mindfulness	-	-	+	X	+	X	!
Psychotherapy versus Mindfulness	-	-	+	X	+	X	!
Music versus Multicomponent	-	-	+	X	+	X	!
PMR versus Multicomponent	-	-	+	X	+	X	!
Psychotherapy versus Multicomponent	-	-	-	X	+	+	!
PMR versus Music	-	-	+	X	+	X	!
Psychotherapy versus Music	-	-	+	X	+	X	!
Psychotherapy versus PMR	-	-	+	X	+	X	!

D1: Within-study bias
D2: Reporting bias
D3: Indirectness
D4: Imprecision
D5: Heterogeneity
D6: Incoherence

Judgement
! Very low certainty
X Major concerns
- Some concerns
+ No concerns

Figure S4: CINeMA assessments: Hypertension, medium-term follow-up, diastolic BP

	D1	D2	D3	D4	D5	D6	Overall
Breathing control versus Passive comparator	-	-	-	X	+	+	!
Meditative movement versus Passive comparator	-	-	+	-	-	+	!
Music versus Passive comparator	-	-	+	X	+	X	!
Nonspecific comparator versus Passive comparator	-	-	+	X	+	+	!
Biofeedback versus Passive comparator	-	-	+	-	-	X	!
Hypnosis versus Passive comparator	-	-	+	X	+	X	!
Meditation versus Passive comparator	-	-	+	X	+	X	!
Mindfulness versus Passive comparator	-	-	+	X	+	X	!
Multicomponent versus Passive comparator	-	-	+	X	+	X	!
PMR versus Passive comparator	-	-	+	X	+	+	!
Psychotherapy versus Passive comparator	-	-	+	-	-	+	!
Biofeedback versus Nonspecific comparator	-	-	+	-	-	-	!
Meditative movement versus Nonspecific comparator	-	-	+	X	+	+	!
Breathing control versus Nonspecific comparator	-	-	+	X	+	X	!
Hypnosis versus Nonspecific comparator	-	-	+	X	+	X	!
Meditation versus Nonspecific comparator	-	-	+	X	+	X	!
Mindfulness versus Nonspecific comparator	-	-	+	X	+	X	!
Multicomponent versus Nonspecific comparator	-	-	+	X	+	X	!
Music versus Nonspecific comparator	-	-	+	X	+	X	!
PMR versus Nonspecific comparator	-	-	+	X	+	+	!
Psychotherapy versus Nonspecific comparator	-	-	+	X	+	X	!
Breathing control versus Biofeedback	-	-	+	X	+	X	!
Hypnosis versus Biofeedback	-	-	-	X	+	X	!
Meditation versus Biofeedback	-	-	+	X	+	X	!
Meditative movement versus Biofeedback	-	-	+	X	+	X	!
Mindfulness versus Biofeedback	-	-	+	X	+	X	!
Multicomponent versus Biofeedback	-	-	+	X	+	+	!
Music versus Biofeedback	-	-	+	X	+	X	!
PMR versus Biofeedback	-	-	+	X	+	X	!
Psychotherapy versus Biofeedback	-	-	+	X	+	+	!
Hypnosis versus Breathing control	-	-	+	X	+	X	!
Meditation versus Breathing control	-	-	+	X	+	X	!
Meditative movement versus Breathing control	-	-	-	X	+	+	!
Mindfulness versus Breathing control	-	-	+	X	+	X	!
Multicomponent versus Breathing control	-	-	+	X	+	X	!
Music versus Breathing control	-	-	+	X	+	X	!
PMR versus Breathing control	-	-	+	X	+	X	!
Psychotherapy versus Breathing control	-	-	+	X	+	X	!
Meditation versus Hypnosis	-	-	+	X	+	X	!
Meditative movement versus Hypnosis	-	-	+	X	+	X	!
Mindfulness versus Hypnosis	-	-	+	X	+	X	!
Multicomponent versus Hypnosis	-	-	-	X	+	X	!
Music versus Hypnosis	-	-	+	X	+	X	!
PMR versus Hypnosis	-	-	+	X	+	X	!
Psychotherapy versus Hypnosis	-	-	+	X	+	X	!
Meditative movement versus Meditation	-	-	+	X	+	X	!
Mindfulness versus Meditation	-	-	+	X	+	X	!
Multicomponent versus Meditation	-	-	+	X	+	X	!
Music versus Meditation	-	-	+	X	+	X	!
PMR versus Meditation	-	-	+	X	+	+	!
Psychotherapy versus Meditation	-	-	+	X	+	X	!
Mindfulness versus Meditative movement	-	-	+	X	+	X	!
Multicomponent versus Meditative movement	-	-	+	X	+	X	!
Music versus Meditative movement	-	-	+	X	+	X	!
PMR versus Meditative movement	-	-	+	X	+	X	!
Psychotherapy versus Meditative movement	-	-	+	X	+	X	!
Multicomponent versus Mindfulness	-	-	+	X	+	X	!
Music versus Mindfulness	-	-	+	X	+	X	!
PMR versus Mindfulness	-	-	+	X	+	X	!
Psychotherapy versus Mindfulness	-	-	+	X	+	X	!
Music versus Multicomponent	-	-	+	X	+	X	!
PMR versus Multicomponent	-	-	+	X	+	X	!
Psychotherapy versus Multicomponent	-	-	-	X	+	+	!
PMR versus Music	-	-	+	X	+	X	!
Psychotherapy versus Music	-	-	+	X	+	X	!
Psychotherapy versus PMR	-	-	+	X	+	X	!

D1: Within-study bias
D2: Reporting bias
D3: Indirectness
D4: Imprecision
D5: Heterogeneity
D6: Incoherence

Judgement
! Very low certainty
X Major concerns
- Some concerns
+ No concerns

Hypertension: Long term follow-up (>12 months)

Due to the sparse data at this time point, we considered that NMA results may not be robust, and therefore chose to present study-level analyses in the main paper. Figure S5 and Figure S6 display the results for all studies (regardless of risk of bias) that compared interventions from the decision set with relevant comparators.

Figure S5: Change in systolic blood pressure for individuals with hypertension at over 12 months follow-up

Study	Intervention Total	Intervention Mean	Intervention SD	Comparator Total	Comparator Mean	Comparator SD	MD	95%-CI	A	B	C	D	E	O
**Autogenic training versus passive comparator**														
Aivazyan 1988b	44	-5.87	10.61	46	1.08	8.82	-6.95	[-10.99; -2.91]	?	+	+	?	?	?
**Biofeedback versus passive comparator**														
Chesney 1987	49	-7.03	12.03	40	-11.50	12.03	4.47	[-0.55; 9.49]	?	+	?	?	?	?
Hatch 1985	3	7.20	11.31	3	-7.40	12.44	14.60	[-4.42; 33.62]	-	+	-	?	+	-
**Hypnosis versus passive comparator**														
Gay 2007	15	-8.60	7.42	15	-1.80	15.37	-6.80	[-15.44; 1.84]	?	+	+	-	+	-
**Multicomponent intervention versus passive comparator**														
Chesney 1987	24	-12.20	12.03	40	-11.50	12.03	-0.70	[-6.79; 5.39]	?	+	?	?	?	?
**PMR versus passive comparator**														
Agras 1987	47	-9.20	16.80	49	-8.40	16.50	-0.80	[-7.46; 5.86]	?	+	?	+	+	?
Chesney 1987	24	-9.80	12.03	40	-11.50	12.03	1.70	[-4.39; 7.79]	?	+	?	?	?	?
Hatch 1985	4	3.80	10.77	3	-7.40	12.44	11.20	[-6.39; 28.79]	-	+	-	?	+	-
Southam 1982	12	-13.30	12.03	18	-4.10	12.03	-9.20	[-17.98; -0.42]	?	-	-	+	?	-
**Relaxation and lifestyle intervention versus passive comparator**														
Chesney 1987	21	-7.50	12.03	40	-11.50	12.03	4.00	[-2.35; 10.35]	?	+	?	?	?	?
**Biofeedback versus nonspecific comparator**														
Hatch 1985	3	7.20	11.31	2	-15.50	13.81	22.70	[-0.32; 45.72]	-	+	-	?	+	-
**PMR versus nonspecific comparator**														
Hatch 1985	4	3.80	10.77	2	-15.50	13.81	19.30	[-2.56; 41.16]	-	+	-	?	+	-
**Psychotherapy versus nonspecific comparator**														
Shapiro 1997	22	-2.37	12.14	17	-9.47	9.06	7.11	[0.45; 13.76]	?	+	+	+	?	?
**Multicomponent intervention versus biofeedback**														
Chesney 1987	24	-12.20	12.03	49	-7.03	12.03	-5.17	[-11.04; 0.70]	?	+	?	?	?	?
**Multicomponent intervention versus PMR**														
Chesney 1987	24	-12.20	12.03	24	-9.80	12.03	-2.40	[-9.20; 4.40]	?	+	?	?	?	?
**Multicomponent intervention versus relaxation and lifestyle intervention**														
Chesney 1987	24	-12.20	12.03	21	-7.50	12.03	-4.70	[-11.74; 2.34]	?	+	?	?	?	?
**PMR versus biofeedback**														
Chesney 1987	24	-9.80	12.03	49	-7.03	12.03	-2.77	[-8.64; 3.10]	?	+	?	?	?	?
Hatch 1985	4	3.80	10.77	3	7.20	11.31	-3.40	[-19.98; 13.18]	-	+	-	?	+	-
**PMR versus relaxation and lifestyle intervention**														
Chesney 1987	24	-9.80	12.03	21	-7.50	12.03	-2.30	[-9.34; 4.74]	?	+	?	?	?	?
**Relaxation and lifestyle intervention versus biofeedback**														
Chesney 1987	21	-7.50	12.03	49	-7.03	12.03	-0.47	[-6.62; 5.68]	?	+	?	?	?	?

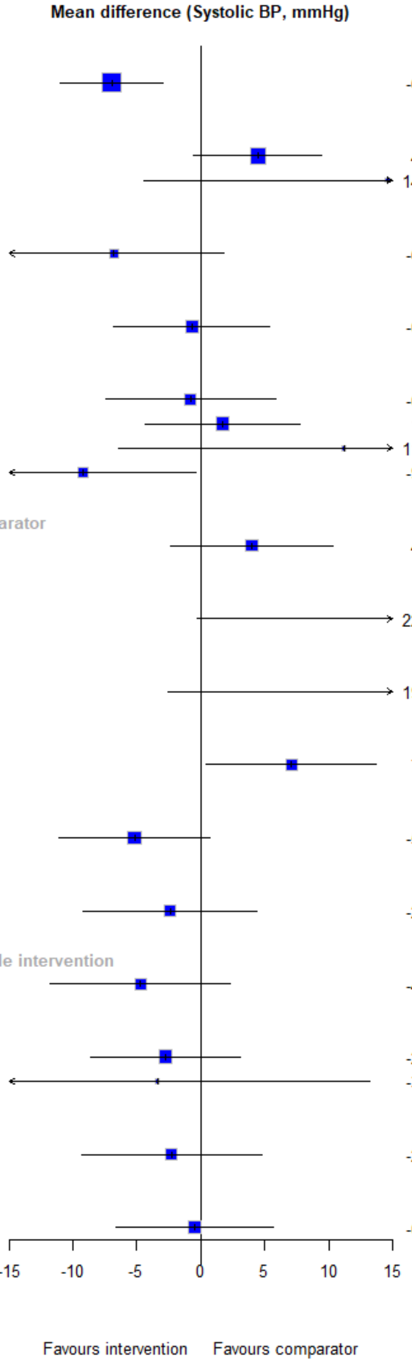

Risk of bias legend
(A) Bias arising from the randomization process
(B) Bias due to deviations from intended intervention
(C) Bias due to missing outcome data
(D) Bias in measurement of the outcome
(E) Bias in selection of the reported result
(O) Overall risk of bias

Figure S6: Change in diastolic blood pressure for individuals with hypertension at over 12 months follow-up

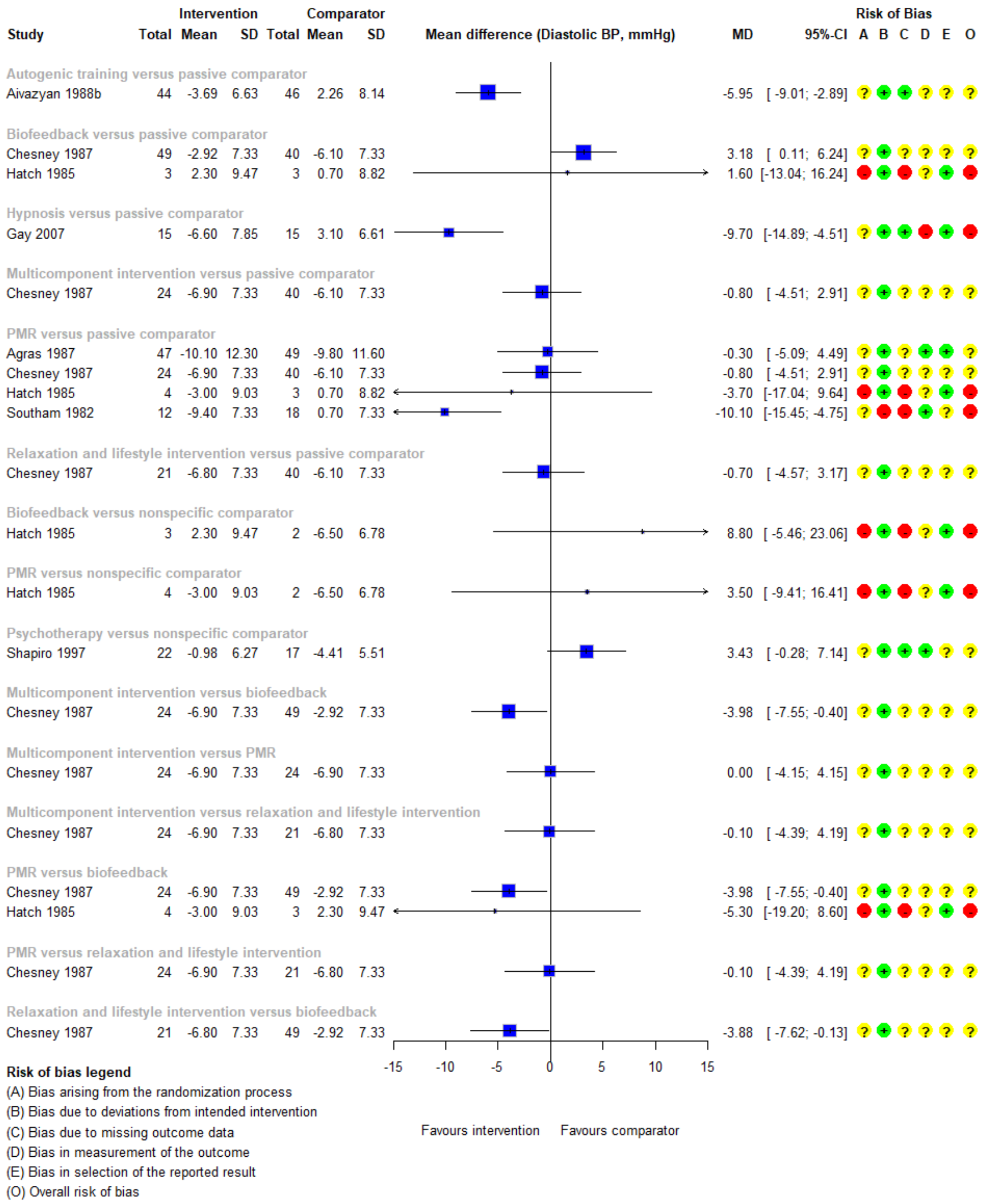

For completeness we also present the results of the NMA here.

Disconnected studies

One study (176) was disconnected from both systolic and diastolic BP evidence networks and was excluded from the NMA. This study compared psychotherapy to a nonspecific comparator and reported treatment effects in favour of the nonspecific comparator (MD = 7.11mmHg, 95% CI 0.45 to 13.76 for systolic BP; MD = 3.43mmHg, 95% CI -0.28 to 7.14 for diastolic BP; very low-certainty evidence). The network plot for the remaining 3 studies, comparing 6 interventions, is presented in Figure S7.

Figure S7: Network plot for hypertension, long-term follow-up

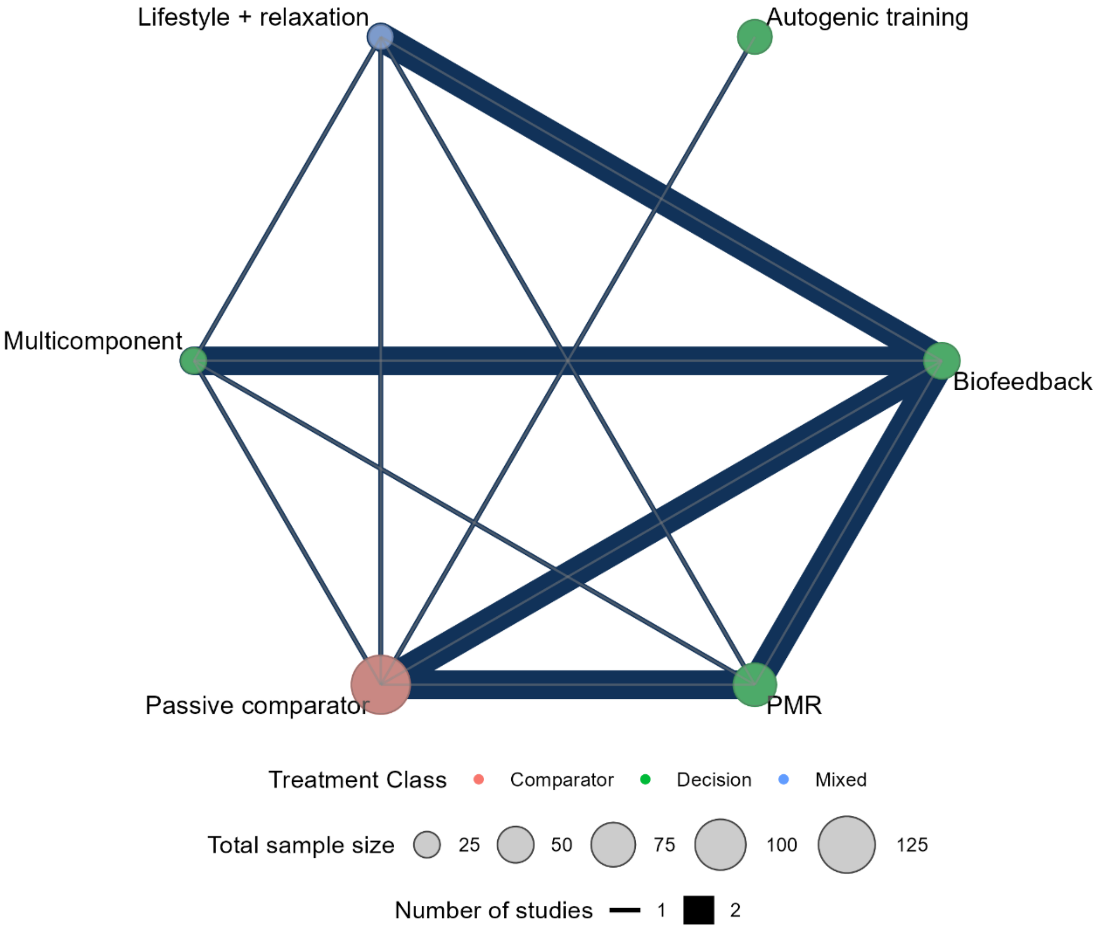

Network meta-analysis

We fitted random-effects NMA models for systolic and diastolic BP at >12 months' follow-up. R-hat values for all estimates were consistent with good convergence for both outcomes. There were no meaningful differences in model fit between fixed effect, random effects consistency models, and random-effects inconsistency models (Table S8, Table S9). Therefore, random-effects consistency models were preferred, with estimated median between-study heterogeneity of 3.84mmHg (95% CrI 0.17 to 16.55) for systolic BP, and 3.08mmHg (95% CrI 0.13 to 16.49) for diastolic BP.

Relative effect estimates based on the NMA models had very wide credible intervals, demonstrating considerable uncertainty in the overall effects (Table S10). Based on the point estimates alone, autogenic training may result in a reduction in systolic and diastolic blood pressure, that exceeds the MID. Multicomponent interventions and PMR had much smaller effects, consistent with a trivial impact on blood pressure. Furthermore, biofeedback appeared to be inferior to a passive comparator.

Hypertension: Sensitivity analyses

Including all studies, regardless of risk of bias

Short-term follow up (≤3 months)

At short follow-up, a further 85 studies were rated at high risk of bias and could be included in the NMA, in addition to those at lower risk of bias. In total, 139 studies reported systolic BP and 137 diastolic BP outcomes in participants with hypertension. Among 25 included intervention nodes, the most common comparisons were between passive comparator and meditative movement (n = 16), biofeedback (n = 11), breathing control (n = 11), PMR (n = 10) or nonspecific comparator (n = 9), between nonspecific comparator and biofeedback (n = 15 for systolic BP, n = 14 for diastolic BP), and between breathing control and music (n = 8). Six or fewer trials contributed direct evidence to the remaining comparisons. Effect estimates based on random-effects NMA consistency models show similar results to the primary analyses (see Table S4, Table S5 for model fit and Table S10 for relative effects). The magnitude of effects is typically slightly larger across the different comparisons, with narrower credible intervals. For most interventions, point estimates of the effect exceeded -5mmHg for systolic BP, and -3mmHg for diastolic BP, indicating that the magnitude of the effect may be important, and the credible intervals did not cross zero. Two additional interventions (hypnosis and massage therapy) were included in this analysis (as the only studies addressing these interventions were assessed to be at high risk of bias). Hypnosis also appeared to cause a reduction in systolic and diastolic blood pressure. Massage therapy had a smaller effect on systolic BP, but may have an important effect on diastolic BP.

Medium-term follow-up (>3-12 months)

For medium follow-up, systolic and diastolic BP were reported in 65 studies in participants with hypertension, 44 of which were considered to be at high risk of bias. The most frequent comparisons among the 24 included intervention nodes were between passive comparator and biofeedback (n = 10), meditative movement (n = 6), nonspecific comparator (n = 5) or PMR (n = 4), between nonspecific comparator and biofeedback (n = 13), PMR (n = 6) or multicomponent interventions, and between lifestyle interventions and PMR (n = 4). Three or fewer trials contributed direct evidence to the remaining comparisons.

Most effect estimates became slightly larger, and the credible intervals became more precise with this analysis (Table S10). Two additional interventions (autogenic training and massage therapy) were

included in the analysis (as the only studies addressing these interventions were assessed to be at high risk of bias). Point estimates for autogenic training indicated a potential benefit over passive comparator, although the credible intervals were wide. Massage therapy did not appear to reduce systolic or diastolic BP, but the credible intervals were also very wide.

Long-term follow-up (>12 months)

At long term follow-up, a further three studies (at high risk of bias) were included, giving 7 studies in total for this analysis. Among 9 included intervention nodes, the most common comparison was between passive comparator and PMR (n = 4), whereas the remaining comparisons were based on 3 or fewer trials. As with the primary analyses, credible intervals were very wide (Table S10), and it is difficult to draw conclusions about the effect of interventions.

Excluding studies with mixed hypertensive and pre-hypertensive participants

As specified in the review protocol, we also conducted a sensitivity analysis excluding studies with mixed hypertensive and pre-hypertensive participants for the primary analyses. We did not conduct this sensitivity analysis at long term follow-up, because all contributing studies were based on individuals with hypertension only. After excluding studies at high risk of bias and studies disconnected from the respective networks, there were 46 trials reporting systolic and diastolic BP at short, and 17 trials at medium follow-up (the study by Ziv *et al* 2013 (215) was disconnected).

In the random-effects NMA models for short and medium follow-up, the estimated between-study heterogeneity was slightly lower relative to the primary analysis (although for medium follow-up it was also less precise; Table S4, Table S5, Table S6, Table S7). There were few substantial changes in the effect estimates with this analysis (Table S10). There were subtle changes in the point estimates for some interventions, but the credible intervals are all very wide, and consistent across the two analyses. The only exception was the effect of psychotherapy on diastolic BP at the medium timepoint, where the wide credible intervals include the possibility of potential benefit or potential harm from the intervention, when studies recruiting a mixed population were excluded.

Biofeedback reclassification

For the primary analysis, any intervention using biofeedback as its main component was classified as biofeedback. However, in some studies, specific relaxation techniques were used to control the physiological signal during biofeedback sessions. We explored the impact of this classification approach in a sensitivity analysis where interventions including biofeedback were categorised primarily according to the relaxation techniques used, and only classified as biofeedback where no techniques were specified.

We fitted random-effects NMA models assuming consistency for systolic and diastolic BP outcomes at each follow-up timepoint in participants with hypertension. The alternative classification did not substantially change the geometry of the evidence networks, although there were no comparisons involving hypnosis at medium follow-up, and none involving biofeedback at long follow-up. At short term follow-up, 54 studies contributed to the comparison of 19 intervention nodes, at medium follow-up, 20 studies contributed to the comparison of 14 interventions, and at long follow-up, 3 studies contributed to the comparison of 5 interventions. The estimates of between-study heterogeneity were comparable to those in the primary analysis in short follow-up models an long follow-up systolic BP model, but were lower in the sensitivity analysis at medium follow-up for both outcomes (possibly due to reclassification of the hypnosis intervention), and higher at long follow-up for diastolic BP (potentially due to reclassification of biofeedback; see Table S4, Table S5, Table S6, Table S7, Table S8, Table S9). Relative effect estimates for relaxation interventions versus a passive

comparator (Table S10) were broadly consistent with those from the primary analyses, with very few exceptions. As before, the point estimates for some interventions did change slightly - in some cases, the estimates now fall just above or just below the MID. However, the credible intervals are wide and the conclusions are generally unaffected. The only intervention that changes noticeably is that for psychotherapy, where the narrower credible intervals now indicate that this intervention may be beneficial at medium follow-up.

Changing pre-post correlation

During preparation of the extracted outcome data for analysis, where our preferred SDs of change-from-baseline were not reliably reported, they were calculated from arm-level SDs at baseline and follow-up using an empirical estimate of pre-post correlation. This correlation was estimated from other studies included in our dataset where SDs of baseline, follow-up, and change-from-baseline estimates were available. Mean estimates ranged from 0.53 to 0.58 depending on exclusion of outliers. Therefore, a conservative estimate of 0.5 pre-post correlation was used for the primary analysis. However, we explored the sensitivity of the results to an alternative correlation value, based on external empirically derived estimates. Balk *et al* 2012 (7) reported median correlations for continuous outcomes measured with a device as 0.83, and for those in the domain of cardiovascular medicine as 0.59. Considering that both types of outcomes are applicable to BP measures, we used a mid-point pre-post correlation of 0.7 to estimate SDs of change from baseline for this sensitivity analysis.

Included studies and network geometry were identical to those in the primary analysis. Having fitted random-effects NMA models to the data with an alternative correlation estimate, at each follow-up timepoint the estimated between-study heterogeneity was comparable to the estimates obtained in the primary analysis (Table S4, Table S5, Table S6, Table S7, Table S8, Table S9). Overall, results were not sensitive to using an alternative pre-post correlation for BP outcomes in participants with hypertension (Table S10).

Imputed data

During data preparation, where either SD (CfB) or SE (CfB) were missing and could not be derived from other within-study statistics, these values were imputed using other studies within the dataset (where this information was available). Where the only available within-study statistics that could be used to estimate missing SDs of change from baseline were within-arm significance levels (i.e. non-exact p values for change from baseline), we also classified these cases as imputation for the purpose of the sensitivity analysis. Four studies were excluded from the short-term analysis and three from the medium-term analysis.

Overall, random-effects NMA models fitted to the data after excluding imputed outcomes provided results consistent with those from the primary analysis at both short and medium follow-up, including comparable or slightly higher estimates of between-study heterogeneity, and wider 95% CrIs around effect estimates. (Table S4, Table S5, Table S6, Table S7, Table S8, Table S9 and Table S10). Of note, there were no comparisons including hypnosis at medium follow-up for this sensitivity analysis

Table S17: Relative effects of relaxation interventions compared to a passive comparator and model fit statistics from a primary network meta-analysis and a post-hoc sensitivity analysis (excluding Achmon et al 1989 (15)) on systolic and diastolic blood pressure at medium term follow-up (>3 to ≤12 months) in people with hypertension, expressed as mean difference (95% credible intervals)

	**Systolic blood pressure**		**Diastolic blood pressure**	
	**Primary analysis**	**Sensitivity analysis (excluding Achmon *et al* 1989 (15))**	**Primary analysis**	**Sensitivity analysis (excluding Achmon *et al* 1989 (15))**
**Relative treatment effects**	**MD in mmHg (95% CrI)**	**MD in mmHg (95% CrI)**	**MD in mmHg (95% CrI)**	**MD in mmHg (95% CrI)**
Nonspecific comparator	-0.05 (-8.04 to 8.23)	5.07 (-0.88 to 10.71)	-0.92 (-6.69 to 5.07)	1.85 (-2.00 to 5.61)
Biofeedback	-5.57 (-14.80 to 3.84)	3.29 (-4.25 to 10.37)	-5.32 (-11.89 to 1.47)	-0.30 (-5.12 to 4.41)
Breathing control	-0.15 (-11.70 to 11.47)	0.48 (-6.21 to 7.15)	-1.60 (-10.79 to 7.54)	-1.35 (-6.92 to 4.27)
Exercise	7.47 (-5.26 to 20.16)	10.72 (3.24 to 17.98)	-0.64 (-9.93 to 8.74)	1.01 (-3.98 to 5.90)
Hypnosis	-15.27 (-33.05 to 2.37)	-6.46 (-19.55 to 6.53)	-8.04 (-20.48 to 4.55)	-3.10 (-11.57 to 5.25)
Lifestyle	2.93 (-6.13 to 13.07)	-2.89 (-8.06 to 2.78)	2.09 (-4.62 to 8.94)	-4.20 (-7.91 to 0.00)
Lifestyle + relaxation	-10.01 (-19.15 to 0.00)	**-12.94 (-16.90 to -8.39)**	-6.11 (-13.02 to 1.19)	**-9.33 (-12.29 to -5.88)**
Meditation	-0.05 (-12.94 to 13.45)	-3.06 (-10.15 to 4.19)	-1.91 (-11.69 to 7.84)	-5.12 (-10.17 to 0.15)
Meditative movement	-6.12 (-12.81 to 0.51)	**-4.80 (-8.59 to -1.08)**	-3.84 (-8.80 to 1.17)	**-3.33 (-5.92 to -0.66)**
Mindfulness	-8.98 (-26.04 to 9.01)	**-14.82 (-26.15 to -3.36)**	-8.02 (-22.81 to 6.77)	**-14.26 (-25.51 to -3.00)**
Multicomponent	-2.17 (-16.43 to 12.17)	-0.23 (-10.81 to 10.48)	-4.21 (-14.90 to 6.31)	-1.41 (-8.73 to 5.79)
Music	-7.89 (-21.23 to 5.53)	**-7.92 (-15.75 to -0.09)**	-2.82 (-13.35 to 7.66)	-2.89 (-9.52 to 3.68)
PMR	-2.98 (-11.13 to 4.97)	-3.32 (-7.82 to 1.20)	-1.74 (-7.64 to 3.92)	-2.16 (-5.31 to 0.93)
Psychotherapy	-4.16 (-13.94 to 5.36)	-5.67 (-13.16 to 1.99)	-4.87 (-11.70 to 2.03)	-2.97 (-7.80 to 1.82)
**Between-study heterogeneity (consistency / inconsistency)**				
tau (median [95% CrI])	5.18 (2.23 to 9.67) / 2.04 (0.09 to 8.82)	1.35 (0.06 to 5.02) / 2.03 (0.09 to 8.69)	4.06 (2.36 to 7.03) / 1.67 (0.08 to 5.75)	1.19 (0.07 to 3.53) / 1.71 (0.09 to 6.1)

**Model fit (consistency / inconsistency)**				
Residual deviance (on N datapoints)	49.8 (49) / 48.1 (49)	44 (46) / 45.2 (46)	49 (49) / 48.8 (49)	44.3 (46) / 45.9 (46)
pD	42.9 / 43.4	35.9 / 40.5	44 / 44.1	36.8 / 41.2
DIC	92.7 / 91.5	79.9 / 85.6	93 / 92.9	81.1 / 87.1

Note. The same sampling parameters were used for the post-hoc sensitivity models as for the primary analysis models, except for reducing step size (adapt_delta = 0.99) for sensitivity analysis consistency models due to divergent transitions warnings. Relative treatment effects are presented for relaxation (decision set) interventions relative to passive comparator. Relative effects where 95% credible intervals do not cross 0 (point of no effect) are highlighted in bold font. MD, mean difference; 95% CrI, 95% credible interval; PMR, progressive muscle relaxation; pD, number of effective parameters; DIC, deviance information criterion.

Hypertension: Subgroup analyses

Antihypertensive medication status

Short term follow-up (≤3 months)

Subgroup analyses of antihypertensive medication status at short term follow-up included 51 studies (3 studies were disconnected from the networks). Participants were unmedicated in 7 studies, some participants were taking antihypertensives in 21 studies, in 14 all participants were on antihypertensives, and 9 trials did not report antihypertensive medication status. According to the protocol, we assessed the effect of different relaxation interventions according to whether participants were receiving antihypertensive medication. We fitted random-effects NMA models to each subset of studies. Details of model fit are presented in Table S4 and Table S5.

Of the studies eligible for the primary analysis, one trial could not be categorised into any of the above groups, and was therefore excluded from the analysis. Perez *et al* 2009 (149) randomised unmedicated participants to receive antihypertensive medication or one of two psychotherapeutic interventions. We therefore considered it inappropriate to combine this study with others that including unmedicated participants only. Reported treatment effects favoured medication over behavioural psychotherapy (MD = 10.95mmHg, 95% CI 4.14 to 17.76 for systolic BP; MD = 6.35mmHg, 95% CI 2.15 to 10.55 for diastolic BP) and self-help psychotherapy (MD = 9.80mmHg, 95% CI 2.36 to 17.24 for systolic BP; MD = 5.35mmHg, 95% CI 0.76 to 9.94 for diastolic BP) at short follow-up.

Participants not taking antihypertensives

In the subgroup of unmedicated participants, 2 studies were disconnected from the network(140, 141). Palomba *et al* 2011 (140) reported small benefits of relaxation plus home BP monitoring relative to home BP monitoring alone on systolic BP (MD = -5.43mmHg, 95% CI -11.76 to 0.9) and diastolic BP (MD = -2.36mmHg, 95% CI -6.62 to 1.9). Pandey *et al* 2023 (141) compared relaxation (meditative movement) plus exercise to exercise alone, reporting meaningful effects of the combined intervention on both systolic BP (MD = -7.00mmHg, 95% CI -9.40 to -4.60) and diastolic BP (MD = -5.00mmHg, 95% CI -7.02 to -2.98). The remaining 7 studies compared 10 treatments in unmedicated participants with hypertension.

Some participants taking antihypertensives

In the subgroup where some participants were on medication, 1 study was disconnected from the network(117), and the remaining 21 studies compared 17 treatments.

All participants taking antihypertensives

In the subgroup including only medicated participants, a comparison between meditation and lifestyle intervention in Dusek *et al* 2008 (64) was disconnected from the network, and reported trivial effects of meditation on both systolic BP (MD = -0.60mmHg, 95% CI -4.94 to 3.74) and diastolic BP (MD = 0.90mmHg, 95% CI -1.43 to 3.23). The remaining 14 studies compared 9 treatments.

Antihypertensive status not reported

The set of 9 studies where medication status was not reported compared 7 treatments.

Summary

Comparing across these subgroups is challenging, not least because different interventions are included for different subgroups (due to the presence or absence of studies in these populations). Overall, it appears that most relaxation interventions have some effect on blood pressure regardless

of the medication status of the participants. The only intervention to show a potentially different effect across the groups is biofeedback, where the point estimates indicate that this may be ineffective for individuals taking medication. For exact relative effect estimates, see Table S10 and Figure S8.

Figure S8: Subgroup analysis of the effects of relaxation therapies compared to a passive comparator at up to 3 months' follow-up, according to medication status

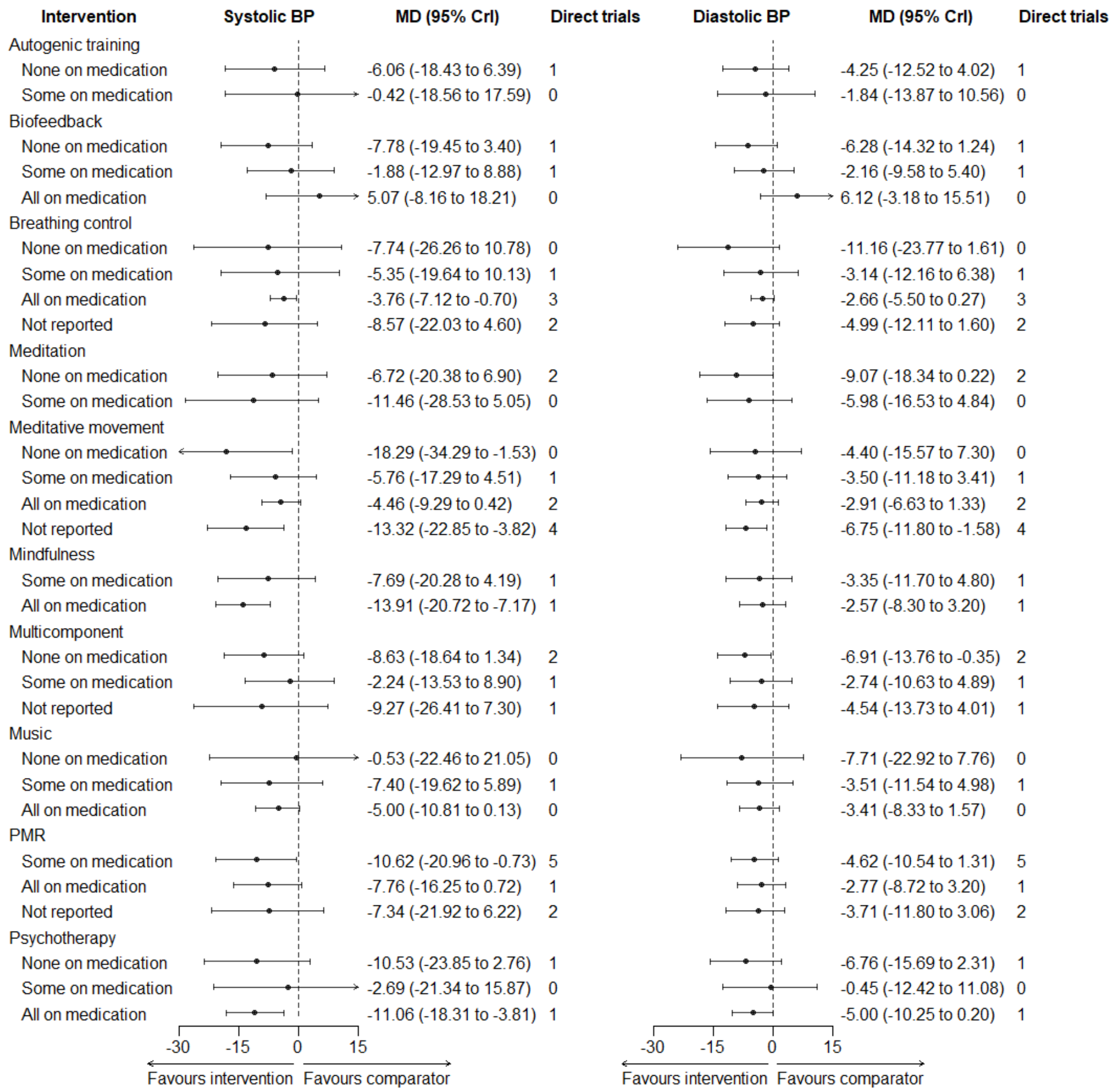

Medium term follow-up (>3-12 months)

At medium follow-up, 17 studies were included. Unmedicated participants were included in 2 studies, some participants were taking antihypertensives in 12 studies, all participants were taking antihypertensives in 5 studies, and 1 study did not report medication status (186). Details of model fit are presented in Table S6 and Table S7.

Participants not taking antihypertensives

For unmedicated participants, 1 study comparing a multicomponent intervention and psychotherapy (35) was disconnected from an already sparse network, therefore, we did not fit NMA models to this subgroup. The remaining 2 studies in unmedicated participants (81, 200) compared biofeedback to a

nonspecific comparator. Bennet *et al* 1991 (35) reported trivial effects favouring psychotherapy over multicomponent relaxation interventions for systolic BP (MD = 2.20mmHg, 95% CI -7.90 to 12.30) and diastolic BP (1.50mmHg, 95% CI -4.59 to 7.59). When compared to a nonspecific comparator, biofeedback was found to have a trivial effect on change in systolic BP (Henderson *et al* 1998 (81) MD = -4.00mmHg, 95% CI -13.43 to 5.43; Wang *et al* 2016 (200) 0.48mmHg, 95% CI -5.07 to 6.03) and uncertain effects on change in diastolic BP (Henderson *et al* 1998 (81) -4.00mmHg, 95% CI -10.17 to 2.17; Wang *et al* 2016 (200) MD = -1.46mmHg, 95% CI -5.46 to 2.54).

Some participants taking antihypertensives

In the subgroup where some participants were taking medication,1 study (117) was disconnected from the network, and the remaining 12 studies compared 14 treatments.

All participants taking antihypertensives

In the subgroup where all participants were on medication, 1 study (215) was disconnected from the network, and the remaining 5 studies compared 5 treatments.

Antihypertensive status not reported

For unreported medication status, Supriya *et al* 2018 (186) reported a trivial effect of meditative movement on systolic BP (MD = -3.12mmHg, 95% CI -8.50 to 2.65) and diastolic BP (MD = -0.78mmHg, 95% CI -4.45 to 2.89) relative to a passive comparator.

Summary

There were insufficient data to draw meaningful conclusions when comparing across the groups at this time point. For exact relative effect estimates, see Table S10 and Figure S9.

Figure S9: Subgroup analysis of the effects of relaxation therapies compared to a passive comparator at >3 to 12 months' follow-up, according to medication status

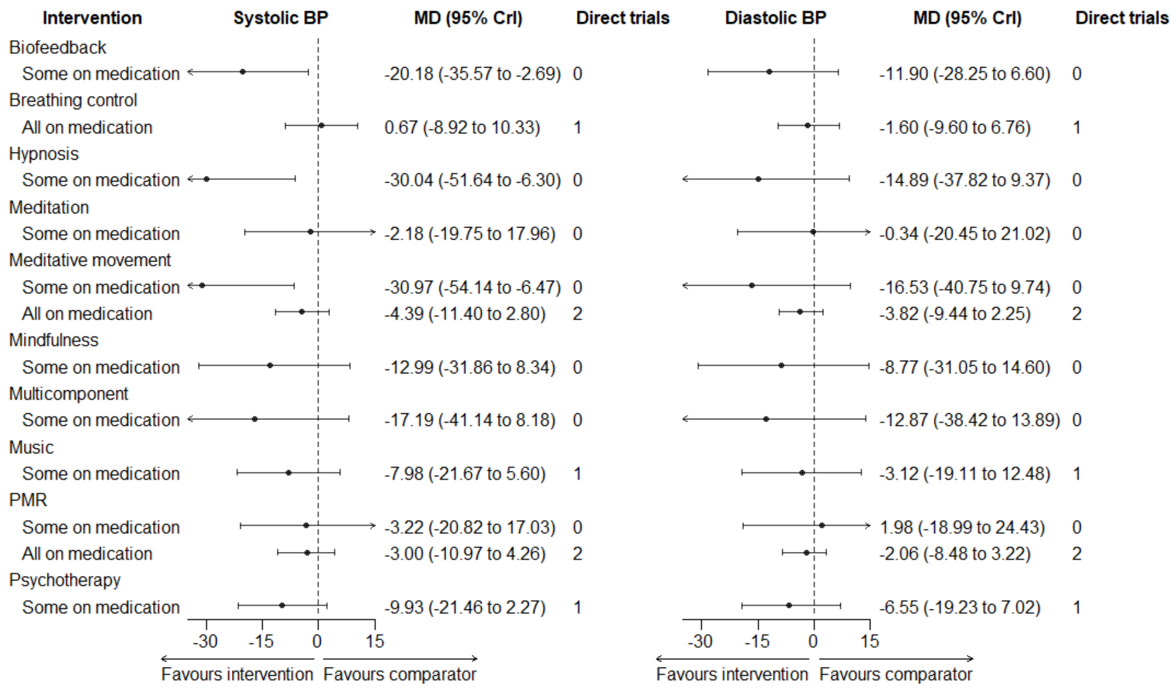

Long term follow-up (> 12 months)

At long follow-up, 1 study included only unmedicated (51), 2 studies only medicated participants (18, 176), whereas 1 study did not report medication status (21). Subgroup analysis was not feasible.

Country-level economic resource

Short-term follow-up (≤3 months)

49 trials reporting outcomes at short follow-up were conducted in higher income countries, and 7 trials in lower income countries. Random effects NMA models fitted to the subset of 47 studies (2 studies (117, 140) were disconnected from the networks) conducted in higher income countries with short follow-up provided very similar estimates of between-study heterogeneity and relative treatment effects to those from the primary analysis. While the subset of studies from higher income countries evaluated a broader range of relaxation interventions, the main difference to those from lower income countries appears to be a greater relative effect of mindfulness on diastolic BP at short follow-up (Table S10 and Figure S10).

Figure S10: Subgroup analysis of the effects of relaxation therapies compared to a passive comparator at up to 3 months' follow-up, according to country-level economic resource

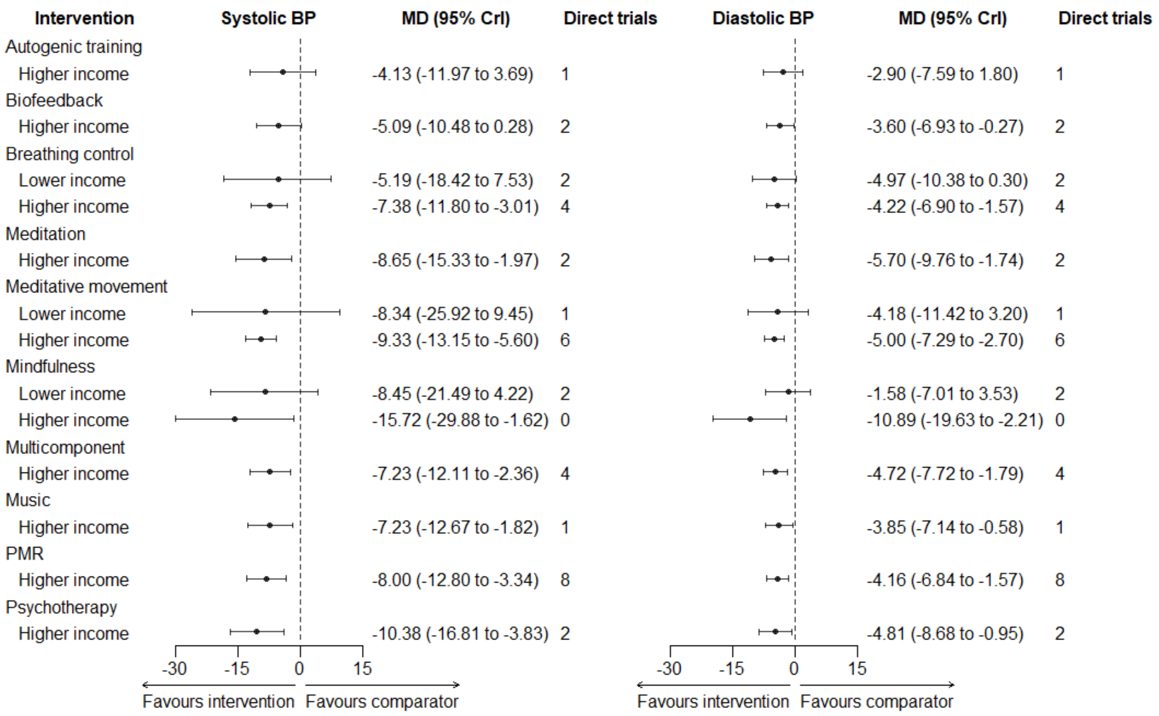

Medium-term follow-up (>3 -12 months)

21 trials reporting outcomes at medium follow-up were from higher income countries, and 2 trials from lower income countries. At medium follow-up, random-effects NMA models fitted to 19 studies (2 studies (117, 140) were disconnected from the networks) from higher income countries provided effect estimates similar to those obtained in the primary analysis, with similar between-study heterogeneity for systolic BP model, and reduced heterogeneity for diastolic BP model. Results of this subgroup analysis suggest that, relative to a passive comparator, hypnosis, meditative movement, and mindfulness may have meaningful effects on reduction in both systolic and diastolic BP. Additionally, music demonstrates a meaningful effect on systolic but a trivial effect on diastolic BP, whereas biofeedback and psychotherapeutic approaches demonstrate meaningful effects on

diastolic but trivial effects on systolic BP. Breathing control, meditation, multicomponent interventions, and PMR were found to have trivial effect on change in either outcome. The main differences from the primary results were that biofeedback no longer had a meaningful effect on systolic BP, and multicomponent interventions on diastolic BP (Table S10 and Figure S11). The number of studies from lower income countries reporting outcomes at medium follow-up was not sufficient to fit NMA models. Dhungana *et al* 2021 (62) compared a lifestyle intervention to the same intervention combined with relaxation (meditative movement) and reported meaningful effects of the combined intervention on systolic BP (MD = -9.20mmHg, 95% CI -12.65 to -5.75) and diastolic BP (MD = -4.30mmHg, 95% CI -6.47 to -2.13). Hasandokht *et al* 2015 (79) compared a passive comparator with a lifestyle intervention combined with a multicomponent relaxation, also reporting meaningful benefits of the combined intervention on systolic BP (-13.40mmHg, 95% CI -14.52 to -12.28) and diastolic BP (MD = -9.82mmHg, 95% CI -10.50 to -9.14).

Figure S11: Subgroup analysis of the effects of relaxation therapies compared to a passive comparator at 3 to 12 months' follow-up, according to country-level economic resource

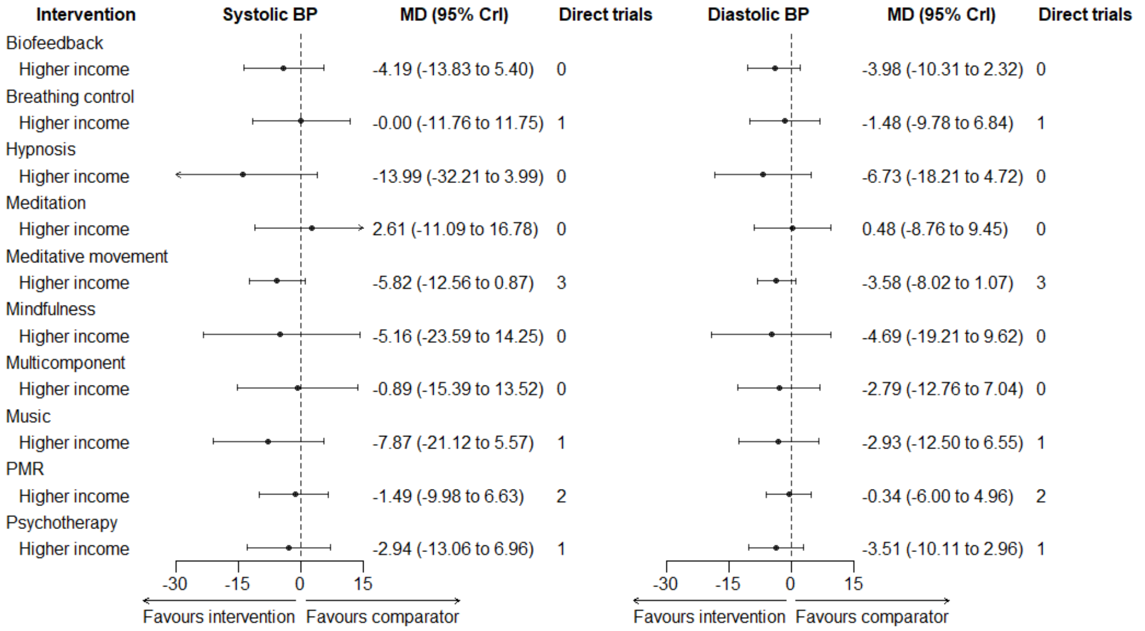

Long-term follow-up (>12 months)

All studies reporting at this time point were conducted in higher income countries, therefore no subgroup analysis was conducted.

Age

Only two of 163 eligible studies recruited participants that were on average ≥75 years old (87, 188). Both reported systolic and diastolic BP outcomes at short follow-up (1 month). Huijuan *et al* 2021 (87) compared meditative movement intervention to a passive comparator, reporting a small effect of meditative movement on systolic BP (MD = -3.09mmHg, 95% CI -8.49 to 2.31) and diastolic BP (MD = -2.20mmHg, 95% CI -6.20 to 1.80), in contrast to the larger effects of this intervention observed in the primary analysis. Teng *et al* 2007 (188) compared a music intervention to a nonspecific comparator, reporting meaningful effects of music on reduction in systolic BP (MD = -8.20mmHg, 95% CI -20.72 to 4.32) and diastolic BP (MD = -3.30mmHg, 95% CI -11.15 to 4.55). In the primary analysis, music relative to a nonspecific comparator had only a trivial effect on changes in BP (Table S11, Table S12). Note that the 95% CIs for the above estimates cross the point of no effect.

We fitted random-effects NMA models for the subset of studies including only participants <75 years old (i.e. excluding the above studies with age ≥75 years) at short term follow-up. After excluding the same disconnected studies as from the primary analysis (117, 140), 52 studies contributed to the comparison of 19 intervention nodes. Estimated between-study heterogeneity was nearly identical to that in the primary analysis (Table S4, Table S5). Relative effects of relaxation interventions compared to a passive comparator in participants <75 years old were also broadly consistent with the primary analysis for both systolic and diastolic BP (Table S10).

Severity of hypertension

For the planned subgroup analysis regarding severity of hypertension, only 10 of 163 studies reported sufficient detail to determine the proportion of participants belonging to each severity category (grade 1 versus grade 2). Six studies included participants with grade 1 hypertension only (29, 101, 103, 128, 148, 195), 2 studies included a combination of participants with grade 1 and grade 2 hypertension (21, 53), and 2 studies included a combination of participants with pre-hypertension and grade 1 hypertension (78, 192). The subgroup analysis was therefore not feasible.

Pre-hypertension analyses

Sixteen studies were identified that included participants with pre-hypertension only (16, 26, 30, 47, 50, 73, 86, 111, 112, 125, 153, 173, 180, 190, 199, 207). However, a number of these studies compared relaxation therapies to supplementary interventions, which were not the primary focus of this review (47, 125, 173, 180, 190).

As described in the article, the small number of studies and diverse interventions/comparators used means that we were unable to conduct network meta-analysis or pairwise meta-analysis for the pre-hypertensive population. We were also unable to conduct our planned subgroup and sensitivity analyses.

For completeness, the results of all studies (regardless of risk of bias) that compared interventions from the decision set with relevant comparators are shown below at short- (Figure S12, Figure S13), medium- (Figure S14, Figure S15) and long-term follow-up (Figure S16, Figure S17).

Figure S12: Change in systolic blood pressure for individuals with pre-hypertension at up to 3 months' follow-up

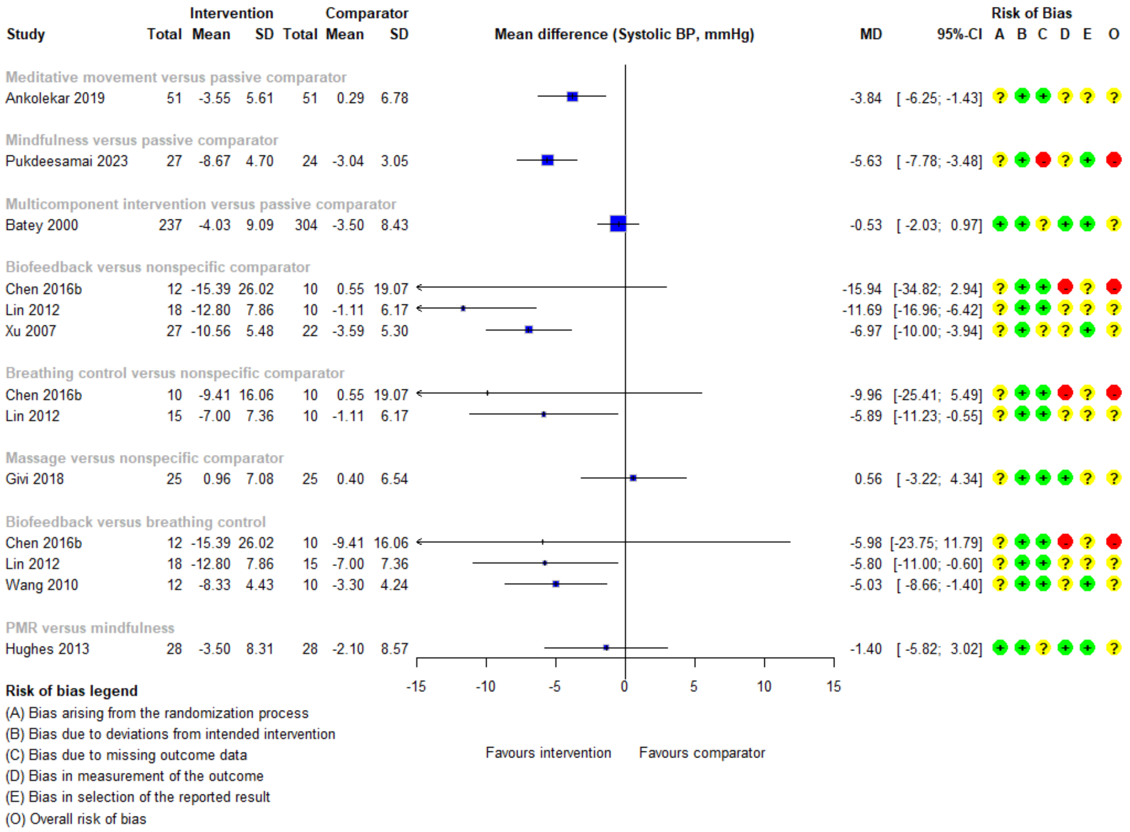

Figure S13: Change in diastolic blood pressure for individuals with pre-hypertension at up to 3 months' follow-up

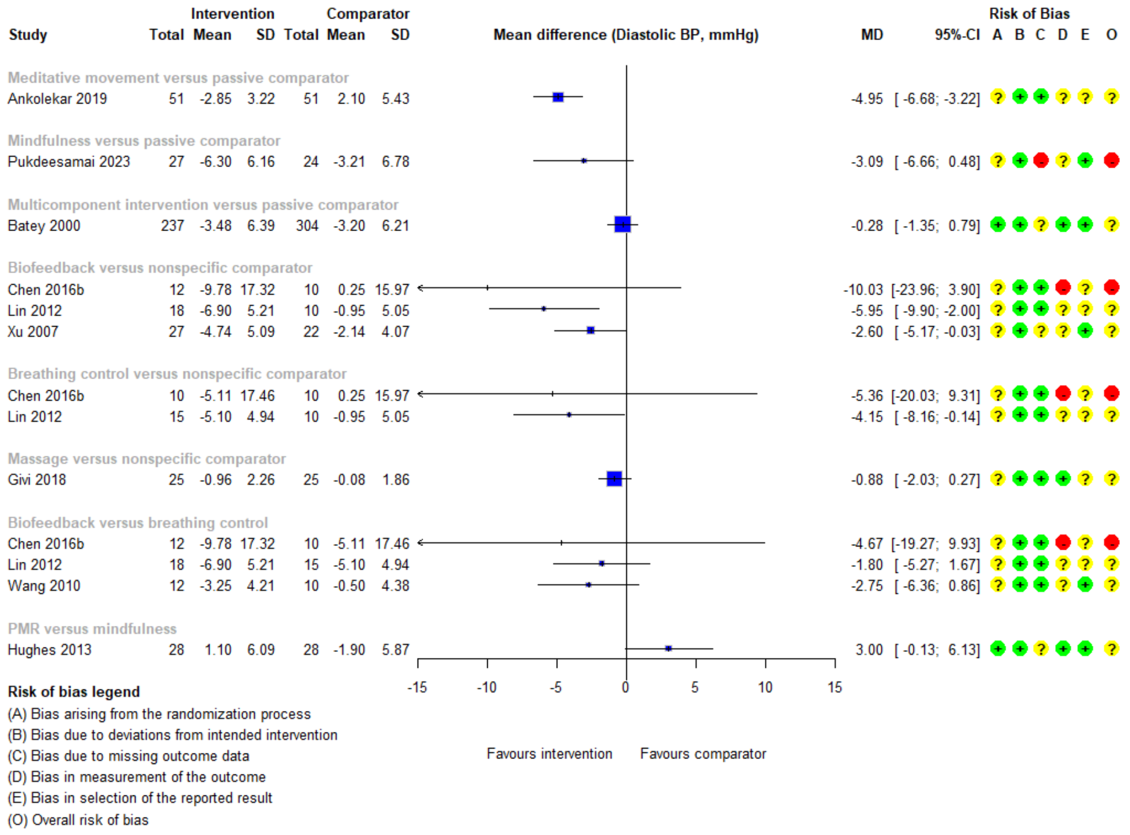

Figure S14: Change in systolic blood pressure for individuals with pre-hypertension at >3 to 12 months' follow-up

Study	Intervention Total	Intervention Mean	Intervention SD	Comparator Total	Comparator Mean	Comparator SD	MD	95%-CI	A	B	C	D	E	O
**Meditative movement versus passive comparator**														
Ankolekar 2019	51	-6.25	5.94	51	1.03	5.92	-7.28	[-9.58; -4.98]	?	+	+	?	?	?
**Mindfulness versus passive comparator**														
Pukdeesamai 2023	27	-11.16	5.43	24	-3.71	3.10	-7.45	[-9.85; -5.05]	?	+	-	?	+	-
**Multicomponent intervention versus passive comparator**														
Batey 2000	226	-4.25	8.23	300	-4.36	7.85	0.11	[-1.28; 1.50]	+	+	-	+	+	-
**Biofeedback versus nonspecific comparator**														
Xu 2007	27	-9.22	5.82	22	-2.55	4.82	-6.67	[-9.65; -3.69]	?	+	?	?	?	?
**Biofeedback versus breathing control**														
Wang 2010	12	-8.50	4.67	10	-2.10	4.09	-6.40	[-10.06; -2.74]	?	+	+	?	+	?

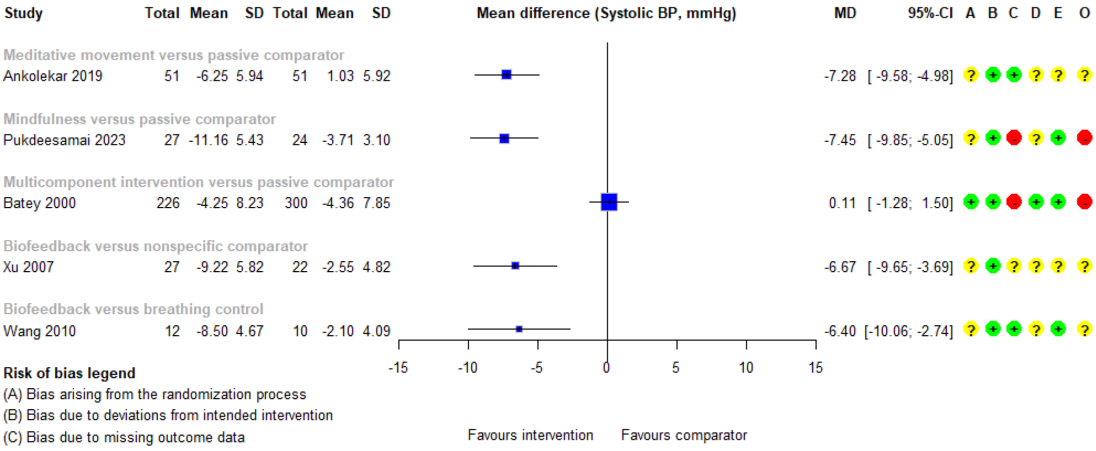

Risk of bias legend
(A) Bias arising from the randomization process
(B) Bias due to deviations from intended intervention
(C) Bias due to missing outcome data
(D) Bias in measurement of the outcome
(E) Bias in selection of the reported result
(O) Overall risk of bias

Figure S15: Change in diastolic blood pressure for individuals with pre-hypertension at >3 to 12 months' follow-up

Study	Intervention Total	Intervention Mean	Intervention SD	Comparator Total	Comparator Mean	Comparator SD	MD	95%-CI	A	B	C	D	E	O
**Meditative movement versus passive comparator**														
Ankolekar 2019	51	-3.90	3.09	51	2.94	5.16	-6.84	[-8.49; -5.19]	?	+	+	?	?	?
**Mindfulness versus passive comparator**														
Pukdeesamai 2023	27	-7.93	5.68	24	-5.75	7.17	-2.18	[-5.76; 1.40]	?	+	-	?	+	-
**Multicomponent intervention versus passive comparator**														
Batey 2000	226	-4.10	5.59	300	-4.48	5.65	0.38	[-0.59; 1.35]	+	+	-	+	+	-
**Biofeedback versus nonspecific comparator**														
Xu 2007	27	-3.78	5.12	22	-1.55	4.24	-2.23	[-4.85; 0.39]	?	+	?	?	?	?
**Biofeedback versus breathing control**														
Wang 2010	12	-2.75	4.18	10	1.40	4.22	-4.15	[-7.68; -0.62]	?	+	+	?	+	?

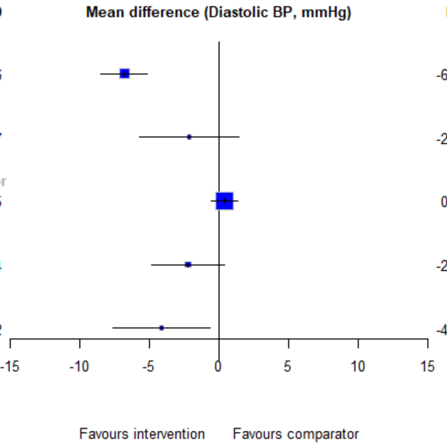

Risk of bias legend
(A) Bias arising from the randomization process
(B) Bias due to deviations from intended intervention
(C) Bias due to missing outcome data
(D) Bias in measurement of the outcome
(E) Bias in selection of the reported result
(O) Overall risk of bias

Figure S16: Change in systolic blood pressure for individuals with pre-hypertension at over 12 months' follow-up

Study	Intervention Total	Intervention Mean	Intervention SD	Comparator Total	Comparator Mean	Comparator SD	MD	95%-CI	A	B	C	D	E	O
**Multicomponent intervention versus passive comparator**														
Batey 2000	224	-4.19	9.21	295	-3.70	8.53	-0.49	[-2.04; 1.06]	+	+	-	+	+	-

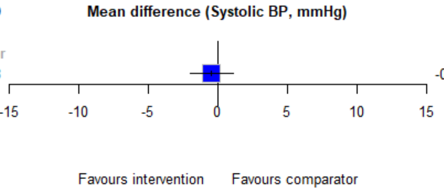

Risk of bias legend
(A) Bias arising from the randomization process
(B) Bias due to deviations from intended intervention
(C) Bias due to missing outcome data
(D) Bias in measurement of the outcome
(E) Bias in selection of the reported result
(O) Overall risk of bias

Figure S17: Change in diastolic blood pressure for individuals with pre-hypertension at over 12 months' follow-up

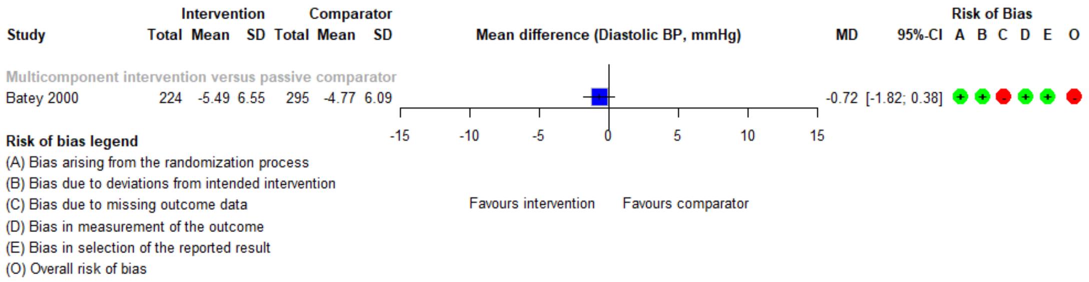

Sensitivity and subgroup analyses

Full GRADE assessments of the evidence, and effect estimates for all included studies (regardless of risk of bias) are shown in the supplementary materials.

All participants in studies including those with pre-hypertension were aged less than 75 years, therefore this subgroup analysis was not possible.

As no meta-analysis was conducted, we could not assess the impact of country-level economic resource on the results. However, for information, at short-term follow-up 7 studies were conducted in higher income countries (16, 30, 86, 112, 125, 199, 207), and 2 studies in lower income countries (26, 73). At medium-term follow-up, 6 studies were from higher income countries (30, 86, 112, 125, 199, 207), and 2 studies were from lower income countries (26, 73).

Analyses according to medication status (use of anti-hypertensives) and grade of hypertension were not appropriate for this population.

Secondary outcomes

Table S18: Narrative synthesis of studies reporting information on mortality, cardiovascular and cerebrovascular morbidity

Study	Comparison	Results	Notes
**Mortality**			
Batey 2000 (30)	Multicomponent relaxation intervention compared to a passive comparator.	Participants were followed-up for 18 months. The authors stated: "There were three deaths during the trial, one each in the active weight reduction, active magnesium nutritional supplement, and life-style change control groups; causes of death were thrombotic occlusion of the right coronary artery, undetermined, and pancreatic cancer, respectively".<br><br>For the purposes of this review, a subset of the 'life-style change control group' were included (only those who were randomized to either the control group or the 'stress management' (multicomponent intervention) group, therefore it is unclear whether any deaths actually occurred in the participants included in our review. Nonetheless, if this death was in the control group, then it would appear to be unrelated to hypertension (pancreatic cancer). Mortality would be 0/224 in the relaxation group, 1/296 in the control group (risk ratio 0.44, 95% CI 0.02 to 10.75)	This large study investigated the cardiovascular preventative effects of a number of different interventions, including weight reduction and various nutritional supplements. Only two relevant arms were included in this review: a 'stress management' (multicomponent relaxation) intervention compared to no treatment. The authors did report some mortality data; however, we are uncertain whether these data were systematically collected for all arms of the study.
Patel 1988 (146)	Multicomponent relaxation intervention compared to a passive comparator.	After 12-months' follow-up, one death was reported in the relaxation group (1/50; cause of death reported as colon cancer) compared to none in the control group (0/54) (risk ratio 3.24, 95% CI 0.13 to 77.63).	Participants in this trial were selected from a previous study, during which they had been allocated to received either antihypertensives or placebo. At the start of the new study, participants were randomized to either continue their current treatment (antihypertensives or placebo) or stop it. Within these four groups, participants were subsequently randomly allocated to receive relaxation therapy or no relaxation therapy, resulting in a total of eight intervention groups. However, mortality results were only

			reported for the overall comparison of relaxation versus no intervention.
Schneider 1995 (166)	Progressive muscle relaxation or transcendental meditation compared to a lifestyle intervention.	After five years' follow-up, all-cause mortality was 3/31 in the meditation group, 8/35 in the progressive muscle relaxation group and 6/32 in the lifestyle intervention group.<br><br>The risk ratio for meditation compared to lifestyle intervention was 0.52 (95% CI 0.14 to 1.88), for PMR compared to lifestyle intervention was 1.22 (95% CI 0.47 to 3.13), and for meditation compared to PMR was 0.42 (95% CI 0.12 to 1.46).	This was a three-armed study.
**Cerebrovascular disease**			
Patel 1988 (146)	Multicomponent relaxation intervention compared to a passive comparator.	After 12 months' follow-up, one stroke occurred in the group receiving relaxation therapy (1/50), compared to none in those receiving no intervention (0/54) (risk ratio 3.24, 95% CI 0.13 to 77.63).	(see notes above)
**Cardiovascular disease**			
Patel 1988 (146)	Multicomponent relaxation intervention compared to a passive comparator.	Occurrence of angina/myocardial infarction after 12-months' follow-up (as reported by general practitioners) was 0/49 for those receiving relaxation therapies, and 2/55 in the group receiving no intervention (risk ratio 0.22, 95% CI 0.01 to 4.55). The authors also assessed the occurrence of cardiovascular ischaemia/infarction using blindly coded ECG reports. Using this method, the occurrence of probable infarction or possible ischaemia was 3/54 in the control group and 0/49 in the relaxation group (risk ratio 0.16, 95% CI 0.01 to 2.97).	(see notes above)
Schneider 1995 (166)	Progressive muscle relaxation or transcendental meditation compared to a lifestyle intervention.	After five years' follow-up, the number of cardiovascular events was 3/28 in the meditation group, 7/31 in the progressive muscle relaxation group and 7/30 in the lifestyle intervention group.<br><br>The risk ratio for meditation compared to lifestyle intervention was 0.46 (95% CI 0.13 to 1.60), for PMR compared to lifestyle intervention was 0.97 (0.39 to 2.43), and for meditation compared to PMR was 0.47 (95% CI 0.14 to 1.66).	

Economic outcomes

Table S19: Narrative synthesis of studies reporting information on economic outcomes

Study	Comparison	Results	Notes
Charlesworth 1984 (48)	Multicomponent intervention compared to home BP monitoring	The authors report the "Mean claims in dollars for health insurance coverage over a 3-year period for 36 hypertensives before and after undergoing a stress management program. The values are half the annual means for 1979 [$225, before stress management] and 1980 [$97, during and after stress management] and the first 6 months of 1981 [$85, after stress management]"	No data are presented for the control group, therefore we are unable to present a comparison of the two study arms.<br><br>Note that these data should be regarded as at high risk of bias, as 4 participants were excluded from these analyses; 2 due to missing data, and 2 due to large claims for bypass surgery of >$10,000 that would have 'distorted the data for analysis'.
Henderson 1998 (81)	Biofeedback compared to non-specific comparator	During this trial, interventions were delivered first in a laboratory setting and subsequently in a home setting. The authors state: "Cost-effectiveness calculations showed the home setting to be 9.9 times more cost effective for the active group and 4.5 times more cost effective for the placebo group than was the laboratory setting. One hour of staff contact time was required for three biofeedback sessions at home compared with 4.5 h for two laboratory sessions."	Comparisons of laboratory versus home are not based on random allocation, but are analyses of the same participants in two different settings.
Hoelscher 1986 (83)	Progressive muscle relaxation compared to no intervention/standard care	This trial included three different methods of delivering progressive muscle relaxation (individual, group, group plus contingency contracting – where participants signed a behavioural contract to commit to practice).<br><br>The authors state: "One-way ANOVAS revealed significant group differences in cost effectiveness for both systolic and diastolic blood pressure, respective Fs $(3, 43) = 4.01$ and 4.97 [$p < 0.05$]. Post hoc comparisons indicated that GR [group relaxation] was significantly more cost effective than IR [individual relaxation] for systolic (by a factor of 5), whereas both GR and GRCC [group relaxation with contingency contracting] were more cost effective than IR for diastolic blood pressure. Significant group differences were also observed for amount of relaxation practice, $F(2, 31) = 18.73$, $p < .0001$. Follow-up tests	The analysis only considers the relative cost-effectiveness of the three progressive muscle relaxation interventions, there is no description of the control group.

		revealed that GR was more cost effective than GRCC (by a factor of 2), which in turn was superior to IR.”	
Kalmatayeva 2014 (97)	Relaxation (psychotherapy) plus medication compared to medication	The authors state: “group 1 [psychotherapy plus medication] required an average of 47.81USD expenditure, with the average number of visits to achieve the target blood pressure at 4.52 (over 7.05 weeks). Average expenses in group 2 [medication] were higher, namely 48.62USD, with an average number of visits at 6.11 or 7.97 weeks. The costs of SBP and DBP reduction per one unit were also lower in the first group compared with the second group.”<br><br>The cost of systolic BP reduction was 1.98 vs. 2.53USD per 1mmHg for group 1 and 2, respectively, and for diastolic BP reduction it was 3.19 vs. 3.73USD per 1mmHg for group 1 and 2, respectively.	The authors indicate that direct costs (the cost of medication and of consulting specialists) were included in these calculations. However, it is not clear whether the costs of the psychotherapy were included (three sessions per week for 6 weeks, lasting 60-90 minutes each).

Risk of Bias 2 Assessments

Table S20: RoB 2 assessments: Hypertension, short term follow-up, individually randomised trials

Study	Intervention	Comparator	Outcome measure	D1	D2	D3	D4	D5	Overall	Description of concerns
Adsett1989	Medication + lifestyle intervention	Lifestyle intervention	Home BP							The allocation sequence could have been predicted as a fixed block size was used and the trial was not blinded. Some data were missing, but this was not considered likely to be due to the true value of participants blood pressure. Outcome assessors were likely to be aware of the intervention received by study participants (although the nurse was stated to be blinded, they were able to correctly guess the group allocation for many participants). However, the use of a standardised blood pressure measuring protocol is likely to have minimised the risk of bias.
Adsett1989	Progressive muscle relaxation	Lifestyle intervention	Home BP							The allocation sequence could have been predicted as a fixed block size was used and the trial was not blinded. Some data were missing, but this was not considered likely to be due to the true value of participants blood pressure.
Adsett1989	Progressive muscle relaxation + medication	Lifestyle intervention	Home BP							The allocation sequence could have been predicted as a fixed block size was used and the trial was not blinded. Some data were missing, but this was not considered likely to be due to the true value of participants blood pressure. Outcome assessors were likely to be aware of the intervention received by study participants (although the nurse was stated to be blinded, they were able to correctly guess the group allocation for many participants). However, the use of a standardised blood pressure measuring protocol is likely to have minimised the risk of bias.
Adsett1989	Progressive muscle relaxation	Medication + lifestyle intervention	Home BP							The allocation sequence could have been predicted as a fixed block size was used and the trial was not blinded. Outcome assessors were likely to be aware of the intervention received by study participants (although the nurse was stated to be blinded, they were able to correctly guess the group allocation for many participants). However, the use of a standardised blood pressure measuring protocol is likely to have minimised the risk of bias.
Adsett1989	Progressive muscle relaxation + medication	Medication + lifestyle intervention	Home BP							The allocation sequence could have been predicted as a fixed block size was used and the trial was not blinded.
Adsett1989	Progressive muscle relaxation + medication	Progressive muscle relaxation	Home BP							The allocation sequence could have been predicted as a fixed block size was used and the trial was not blinded. Outcome assessors were likely to be aware of the intervention received by study participants (although the nurse was stated to be blinded, they were able to correctly guess the group allocation for many participants). However, the use of a standardised blood pressure measuring protocol is likely to have minimised the risk of bias.
Ahmadpanah2016	Mindfulness	Non-specific comparator	Office BP (attended)							Outcome assessors were likely to be aware of the intervention received by study participants, and no standardised protocol was used to measure blood pressure. The analysis plan outlined on the trial registry site appears to relate to a different trial.
Ahmadpanah2016	Multicomponent relaxation	Non-specific comparator	Office BP (attended)							Outcome assessors were likely to be aware of the intervention received by study participants, and no standardised protocol was used to measure blood pressure. The analysis plan outlined on the trial registry site appears to relate to a different trial.

Ahmadpanah2016	Multicomponent relaxation	Mindfulness	Office BP (attended)							Outcome assessors were likely to be aware of the intervention received by study participants, and no standardised protocol was used to measure blood pressure. The analysis plan outlined on the trial registry site appears to relate to a different trial.
Aivazyan1988a	Non-specific comparator	No intervention/ Standard care	Office BP							Allocation concealment and randomization were not clearly described. A per protocol analysis was conducted, but the impact of this on the overall result was considered to be small. Outcome assessors were likely to be aware of the intervention received by study participants. However, the use of a standardised blood pressure measuring protocol is likely to have minimised the risk of bias.
Aivazyan1988a	Autogenic training	No intervention/ Standard care	Office BP							Allocation concealment and randomization were not clearly described. A per protocol analysis was conducted, but the impact of this on the overall result was considered to be small. Outcome assessors were likely to be aware of the intervention received by study participants. However, the use of a standardised blood pressure measuring protocol is likely to have minimised the risk of bias.
Aivazyan1988a	Biofeedback	No intervention/ Standard care	Office BP							Allocation concealment and randomization were not clearly described. A per protocol analysis was conducted, but the impact of this on the overall result was considered to be small. Outcome assessors were likely to be aware of the intervention received by study participants. However, the use of a standardised blood pressure measuring protocol is likely to have minimised the risk of bias.
Aivazyan1988a	Multicomponent relaxation	No intervention/ Standard care	Office BP							Allocation concealment and randomization were not clearly described. A per protocol analysis was conducted, but the impact of this on the overall result was considered to be small. Outcome assessors were likely to be aware of the intervention received by study participants. However, the use of a standardised blood pressure measuring protocol is likely to have minimised the risk of bias.
Aivazyan1988a	Autogenic training	Non-specific comparator	Office BP							Allocation concealment and randomization were not clearly described. A per protocol analysis was conducted, but the impact of this on the overall result was considered to be small. Outcome assessors were likely to be aware of the intervention received by study participants. However, the use of a standardised blood pressure measuring protocol is likely to have minimised the risk of bias.
Aivazyan1988a	Biofeedback	Non-specific comparator	Office BP							Allocation concealment and randomization were not clearly described. A per protocol analysis was conducted, but the impact of this on the overall result was considered to be small. Outcome assessors were likely to be aware of the intervention received by study participants. However, the use of a standardised blood pressure measuring protocol is likely to have minimised the risk of bias.
Aivazyan1988a	Multicomponent relaxation	Non-specific comparator	Office BP							Allocation concealment and randomization were not clearly described. A per protocol analysis was conducted, but the impact of this on the overall result was considered to be small. Outcome assessors were likely to be aware of the intervention received by study participants. However, the use of a standardised blood pressure measuring protocol is likely to have minimised the risk of bias.
Aivazyan1988a	Biofeedback	Autogenic training	Office BP							Allocation concealment and randomization were not clearly described. A per protocol analysis was conducted, but the impact of this on the overall result was considered to be small. Outcome assessors were likely to be aware of the intervention received by study participants. However, the use of a standardised blood pressure measuring protocol is likely to have minimised the risk of bias.
Aivazyan1988a	Multicomponent relaxation	Autogenic training	Office BP							Allocation concealment and randomization were not clearly described. A per protocol analysis was conducted, but the impact of this on the overall result was considered to be small. Outcome assessors were likely to be aware of the intervention received by study participants. However, the use of a standardised blood pressure measuring protocol is likely to have minimised the risk of bias.
Aivazyan1988a	Multicomponent relaxation	Biofeedback	Office BP							Allocation concealment and randomization were not clearly described. A per protocol analysis was conducted, but the impact of this on the overall result was considered to be small. Outcome assessors were likely to be aware of the intervention received by study participants. However, the use of a standardised blood pressure measuring protocol is likely to have minimised the risk of bias.

Altena2009	Breathing intervention	Music	Office BP (attended)							Outcome assessors were likely to be aware of the intervention received by study participants. However, the use of a standardised blood pressure measuring protocol is likely to have minimised the risk of bias.
Amigo1997	Exercise	Non-specific comparator	Office BP (attended)							Allocation concealment and randomization were not clearly described. Baseline characteristics of the two groups indicated a problem with the randomization process (much younger participants were assigned to the exercise intervention).Some data were missing, but this was not considered likely to be due to the true value of participants blood pressure.
Amigo1997	Progressive muscle relaxation	Non-specific comparator	Office BP (attended)							Allocation concealment and randomization were not clearly described. Baseline characteristics of the two groups indicated a problem with the randomization process (much younger participants were assigned to the exercise intervention). Some data were missing, but this was not considered likely to be due to the true value of participants blood pressure.
Amigo1997	Progressive muscle relaxation	Exercise	Office BP (attended)							Allocation concealment and randomization were not clearly described. Baseline characteristics of the two groups indicated a problem with the randomization process (much younger participants were assigned to exercise intervention).
Anderson2010	Meditation	Breathing intervention	Office BP (attended)							Allocation concealment and randomization were not clearly described. A per protocol analysis was conducted, but the impact of this on the overall result was considered to be small.
Anjana2022	Meditation	No intervention/ Standard care	Office BP (attended)							Allocation concealment was not clearly described - the sequence could have been predicted if a fixed, small block size was used in this unblinded trial. Multiple participants dropped out of the study and were excluded from the analysis, but no reasons were provided. It is not clear whether this was due to the use of a per-protocol analysis. Some data were missing, but this was not considered likely to be due to the true value of participants blood pressure. Outcome assessors may have been aware of the intervention received by study participants. However, the use of a standardised blood pressure measuring protocol is likely to have minimised the risk of bias. The analysis plan outlined on the trial registry site/in the protocol appears to differ from this analysis because those with pre-hypertension and stage 2 hypertension should also have been included. However, no data are shown for these participants.
Balasubramanian2012	Breathing intervention	No intervention/ Standard care	Office BP (attended)							Allocation concealment was not clearly described.
Bekiroglu2013	Music	Non-specific comparator	Home BP							Allocation concealment was not clearly described. Although the first participants were randomly allocated, it appears that two additional participants (recruited later in the trial) were not randomly allocated to the groups. Outcome assessors were likely to be aware of the intervention received by study participants, and no standardised protocol was used to measure blood pressure.
Bennett1991	Psychotherapy	No intervention/ Standard care	Office BP (attended)							Allocation concealment and randomization were not clearly described. Outcome assessors were likely to be aware of the intervention received by study participants. However, the use of a standardised blood pressure measuring protocol is likely to have minimised the risk of bias.
Bennett1991	Multicomponent relaxation	No intervention/ Standard care	Office BP (attended)							Allocation concealment and randomization were not clearly described. Some data were missing, but this was not considered likely to be due to the true value of participants blood pressure. Outcome assessors were likely to be aware of the intervention received by study participants. However, the use of a standardised blood pressure measuring protocol is likely to have minimised the risk of bias.

Bennett1991	Multicomponent relaxation	Psychotherapy	Office BP (attended)							Allocation concealment and randomization were not clearly described. Some data were missing, but this was not considered likely to be due to the true value of participants blood pressure. Outcome assessors were likely to be aware of the intervention received by study participants. However, the use of a standardised blood pressure measuring protocol is likely to have minimised the risk of bias.
Blanchard1979	Biofeedback	Non-specific comparator	Office BP (attended)							Allocation concealment and randomization were not clearly described. Some data were missing, but this was not considered likely to be due to the true value of participants blood pressure.
Blanchard1984	PMR	Biofeedback	Home BP							Allocation concealment and randomization were not clearly described. A per protocol analysis was conducted, but the impact of this on the overall result was considered to be small. Some data were missing, but this was not considered likely to be due to the true value of participants blood pressure. Outcome assessors were likely to be aware of the intervention received by study participants, and no standardised protocol was used to measure blood pressure. No pre-specified analysis plan was available. The result may have been selected on the basis of the results because outcome measurements could potentially be averaged in different ways / over different time periods and it is not clear whether the reported approach was pre-specified.
Blanchard1988	Autogenic training	Non-specific comparator	Office BP (attended)							Allocation concealment and randomization were not clearly described. The allocation sequence could have been predicted as several dropouts were replaced by assigning new participants directly into the specific group. Outcome assessors were likely to be aware of the intervention received by study participants, and no standardised protocol was used to measure blood pressure.
Blanchard1988	Biofeedback	Non-specific comparator	Office BP (attended)							Allocation concealment and randomization were not clearly described. The allocation sequence could have been predicted as several dropouts were replaced by assigning new participants directly into the specific group. Outcome assessors were likely to be aware of the intervention received by study participants, and no standardised protocol was used to measure blood pressure.
Blanchard1988	Biofeedback	Autogenic training	Office BP (attended)							Allocation concealment and randomization were not clearly described. The allocation sequence could have been predicted as several dropouts were replaced by assigning new participants directly into the specific group. Outcome assessors were likely to be aware of the intervention received by study participants, and no standardised protocol was used to measure blood pressure.
Blanchard1993	Biofeedback	Home BP monitoring	Home BP							Allocation concealment and randomization were not clearly described. Some data were missing, but this was not considered likely to be due to the true value of participants blood pressure. Outcome assessors were likely to be aware of the intervention received by study participants, and no standardised protocol was used to measure blood pressure.
Blanchard1996	Biofeedback	Home BP monitoring	Office BP (attended)							Allocation concealment and randomization were not clearly described. Some data were missing, and this was considered likely to be due to the true value of participants blood pressure as three participants were excluded due to excessively high blood pressure. Outcome assessors were likely to be aware of the intervention received by study participants. However, the use of a standardised blood pressure measuring protocol is likely to have minimised the risk of bias. No pre-specified analysis plan was available. The result may have been selected on the basis of the results because home and ambulatory BP were also measured. However, home data were not reported, and ambulatory data were only reported as post-treatment event data.
Blom2014	Mindfulness	No intervention/ Standard care	Ambulatory 24 hour BP							Some data were missing, and this was considered likely to be due to the true value of participants blood pressure. Some participants did not provide outcome data as they started anti-hypertensive medication.

Bosley1989	Non-specific comparator	No intervention/ Standard care	Office BP (attended)	Allocation concealment and randomization were not clearly described. No pre-specified analysis plan was available. There was insufficient information to assess whether the result may have been selected preferentially.
Bosley1989	Psychotherapy	No intervention/ Standard care	Office BP (attended)	Allocation concealment and randomization were not clearly described. No pre-specified analysis plan was available. There was insufficient information to assess whether the result may have been selected preferentially.
Bosley1989	Psychotherapy	Non-specific comparator	Office BP (attended)	Allocation concealment and randomization were not clearly described. No pre-specified analysis plan was available. There was insufficient information to assess whether the result may have been selected preferentially.
Brauer1979	Progressive muscle relaxation	Non-specific comparator	Office BP (attended)	Allocation concealment and randomization were not clearly described. Some data were missing, but this was not considered likely to be due to the true value of participants blood pressure.
Canino1994	Non-specific comparator	No intervention/ Standard care	Office BP	Allocation concealment and randomization were not clearly described. The imbalanced size of the groups indicates a problem with the randomization process. Outcome assessors were likely to be aware of the intervention received by study participants. However, the use of a standardised blood pressure measuring protocol is likely to have minimised the risk of bias. No pre-specified analysis plan was available. There was insufficient information to assess whether the result may have been selected preferentially.
Canino1994	Multicomponent relaxation	No intervention/ Standard care	Office BP	Allocation concealment and randomization were not clearly described. The imbalanced size of the groups indicates a problem with the randomization process. Outcome assessors were likely to be aware of the intervention received by study participants. However, the use of a standardised blood pressure measuring protocol is likely to have minimised the risk of bias. No pre-specified analysis plan was available. There was insufficient information to assess whether the result may have been selected preferentially.
Canino1994	Multicomponent relaxation	Non-specific comparator	Office BP	Allocation concealment and randomization were not clearly described. The imbalanced size of the groups indicates a problem with the randomization process. Outcome assessors were likely to be aware of the intervention received by study participants. However, the use of a standardised blood pressure measuring protocol is likely to have minimised the risk of bias. No pre-specified analysis plan was available. There was insufficient information to assess whether the result may have been selected preferentially.
Chan2018	Exercise	Non-specific comparator	Office BP	Some data were missing, but this was not considered likely to be due to the true value of participants blood pressure.
Chan2018	Meditative movement	Non-specific comparator	Office BP	Some data were missing, but this was not considered likely to be due to the true value of participants blood pressure.
Chan2018	Meditative movement	Exercise	Office BP	Some data were missing, but this was not considered likely to be due to the true value of participants blood pressure.
Charlesworth1984	Multicomponent relaxation	Home BP monitoring	Home BP	The allocation sequence could have been predicted as the text indicates that participants were assigned a number, and then split into two groups. A per protocol analysis was conducted, but the impact of this on the overall result was considered to be small. Some data were missing, but this was not considered likely to be due to the true value of participants blood pressure. Outcome assessors were likely to be aware of the intervention received by study participants, and no standardised protocol was used to measure blood pressure. No pre-specified analysis plan was available. The result may have been selected on the basis of the results because the authors report that "Only statistics equal to or less than the P=0.05 level will be reported."

Cheung2005	Meditative movement	Non-specific comparator	Office BP (attended)							Allocation concealment and randomization were not clearly described. Baseline characteristics of the two groups indicate a problem with the randomization process, as systolic blood pressure was higher in the intervention group. Some data were missing, but this was not considered likely to be due to the true value of participants blood pressure. Outcome assessors were likely to be aware of the intervention received by study participants. However, the use of a standardised blood pressure measuring protocol is likely to have minimised the risk of bias.
Cohen1983	Meditation	Home BP monitoring	Office BP (attended)							Allocation concealment and randomization were not clearly described. Outcome assessors were likely to be aware of the intervention received by study participants, and no standardised protocol was used to measure blood pressure. No pre-specified analysis plan was available. The result may have been selected on the basis of the results because three methods of assessing blood pressure were used, but only one was reported. Despite a blinded outcome measurement being available, this was not prioritised for analysis.
Cohen1983	Biofeedback	Home BP monitoring	Office BP (attended)							Allocation concealment and randomization were not clearly described. Outcome assessors were likely to be aware of the intervention received by study participants, and no standardised protocol was used to measure blood pressure. No pre-specified analysis plan was available. The result may have been selected on the basis of the results because three methods of assessing blood pressure were used, but only one was reported. Despite a blinded outcome measurement being available, this was not prioritised for analysis.
Cohen1983	Biofeedback	Meditation	Office BP (attended)							Allocation concealment and randomization were not clearly described. Outcome assessors were likely to be aware of the intervention received by study participants, and no standardised protocol was used to measure blood pressure. No pre-specified analysis plan was available. The result may have been selected on the basis of the results because three methods of assessing blood pressure were used, but only one was reported. Despite a blinded outcome measurement being available, this was not prioritised for analysis.
Cohen2011	Meditative movement	Lifestyle intervention	Ambulatory 24 hour BP							Allocation concealment and randomization were not clearly described. There was considerable group imbalance in allocation (46 versus 32 participants). A per protocol analysis was conducted, and the impact of this on the overall result may be substantial, as 17% participants were not analyzed in their assigned group. Some data were missing, but this was not considered likely to be due to the true value of participants blood pressure.
Cohen2016	Meditative movement	Lifestyle intervention	Ambulatory 24 hour BP							Allocation concealment was not clearly described. Baseline characteristics of the two groups indicate a problem with the randomization process (imbalance in group allocation despite blocked randomisation). Participants and carers were aware of the assigned intervention during the trial, and this does appear to have led to deviations from the intended intervention (different number of participants dropped out due to dissatisfaction with their allocated interventions). Some data were missing, and this was considered likely to be due to the true value of participants blood pressure as some were excluded due to high BP and the need to initiate antihypertensive medication.
Cohen2016	Relaxation + lifestyle intervention	Lifestyle intervention	Ambulatory 24 hour BP							Allocation concealment was not clearly described. Baseline characteristics of the two groups indicate a problem with the randomization process (imbalance in group allocation despite blocked randomisation). Participants and carers were aware of the assigned intervention during the trial, and this does appear to have led to deviations from the intended intervention (participants dropped out due to dissatisfaction with their allocated interventions, but this was balanced across the groups). Some data were missing, and this was considered likely to be due to the true value of participants blood pressure as some were excluded due to high BP and the need to initiate antihypertensive medication.

Cohen2016	Relaxation + lifestyle intervention	Meditative movement	Ambulatory 24 hour BP							Allocation concealment was not clearly described. Baseline characteristics of the two groups indicate a problem with the randomization process (imbalance in group allocation despite blocked randomisation). Participants and carers were aware of the assigned intervention during the trial, and this does appear to have led to deviations from the intended intervention (different number of participants dropped out due to dissatisfaction with their allocated interventions). Some data were missing, and this was considered likely to be due to the true value of participants blood pressure as some were excluded due to high BP and the need to initiate antihypertensive medication.
Cramer2018	Breathing intervention	No intervention/ Standard care	Ambulatory 24 hour BP							Some data were missing, but this was not considered likely to be due to the true value of participants blood pressure.
Cramer2018	Meditative movement	No intervention/ Standard care	Ambulatory 24 hour BP							Some data were missing, but this was not considered likely to be due to the true value of participants blood pressure.
Cramer2018	Meditative movement	Breathing intervention	Ambulatory 24 hour BP							Some data were missing, but this was not considered likely to be due to the true value of participants blood pressure.
de Barros2017	Breathing intervention	Music	Office BP (attended)							Allocation concealment was not clearly described. A per protocol analysis was conducted, and the impact of this on the overall result may be substantial (full reasons for all missing data are not described).Some data were missing, but this was not considered likely to be due to the true value of participants blood pressure.
de Fatima Rosas Marchiori2015	Meditation	No intervention/ Standard care	Office BP							Allocation concealment and randomization were not clearly described. A per protocol analysis was conducted, and the impact of this on the overall result may be substantial, due to the number of participants excluded. Some data were missing, and this was considered likely to be due to the true value of participants blood pressure as participants with changes in their antihypertensives were excluded. Outcome assessors were likely to be aware of the intervention received by study participants. However, the use of a standardised blood pressure measuring protocol is likely to have minimised the risk of bias.
Drazen1982	Progressive muscle relaxation	Lifestyle intervention	Office BP (attended)							Baseline characteristics of the two groups indicate a problem with the randomization process. Outcome assessors were likely to be aware of the intervention received by study participants, and no standardised protocol was used to measure blood pressure.
Drazen1982	Psychotherapy	Lifestyle intervention	Office BP (attended)							Baseline characteristics of the two groups indicate a problem with the randomization process. Outcome assessors were likely to be aware of the intervention received by study participants, and no standardised protocol was used to measure blood pressure.
Drazen1982	Psychotherapy	Progressive muscle relaxation	Office BP (attended)							Baseline characteristics of the two groups indicate a problem with the randomization process.Outcome assessors were likely to be aware of the intervention received by study participants, and no standardised protocol was used to measure blood pressure.
Dusek2008	Meditation	Lifestyle intervention	Office BP (attended)							Some data were missing, but this was not considered likely to be due to the true value of participants blood pressure.
Elavally2022	Biofeedback	No intervention/ Standard care	Office BP (attended)							Allocation concealment was not clearly described. Some data were missing, and this was considered likely to be due to the true value of participants blood pressure, as those who required a change in antihypertensive medication were excluded from the analysis.
Elliot2004	Relaxation + Home BP monitoring	Home BP monitoring	Home BP							The allocation sequence could have been predicted as the first 29 participants enrolled were all specifically allocated to the intervention group. Some data were missing, and there was no information available to assess whether this may be related to the blood pressure.

Fetter2020	Meditative movement	Breathing intervention	not reported							Allocation concealment and randomization were not clearly described. A per protocol analysis was conducted, and the impact of this on the overall result may be substantial due to the number of participants who were excluded. Some data were missing, but this was not considered likely to be due to the true value of participants blood pressure.
Friedman1977a	Biofeedback	Hypnosis	Office BP (attended)							Allocation concealment and randomization were not clearly described. Outcome assessors were likely to be aware of the intervention received by study participants, and no standardised protocol was used to measure blood pressure.
Friedman1977b	Biofeedback	Non-specific comparator	Office BP (attended)							Allocation concealment and randomization were not clearly described. Outcome assessors were likely to be aware of the intervention received by study participants, and no standardised protocol was used to measure blood pressure.
GarciaVera1997	Multicomponent relaxation	No intervention/ Standard care	Home BP							Allocation concealment and randomization were not clearly described. Outcome assessors were likely to be aware of the intervention received by study participants. However, the use of a standardised blood pressure measuring protocol is likely to have minimised the risk of bias.
Gay2007	Hypnosis	No intervention/ Standard care	Office BP (attended)							Allocation concealment and randomization were not clearly described.Outcome assessors were likely to be aware of the intervention received by study participants, and no standardised protocol was used to measure blood pressure.
Grossman2001	Breathing intervention	Music	Home BP							Allocation concealment was not clearly described.
Hafner1982	Meditation	No intervention/ Standard care	Office BP							Allocation concealment and randomization were not clearly described. A per protocol analysis was conducted, and the impact of this on the overall result may be substantial as some participants were included in the analysis twice (in the group they were originally assigned to, and the group that they transferred to). Outcome assessors were likely to be aware of the intervention received by study participants, and no standardised protocol was used to measure blood pressure.
Hafner1982	Biofeedback	No intervention/ Standard care	Office BP							Allocation concealment and randomization were not clearly described. A per protocol analysis was conducted, and the impact of this on the overall result may be substantial as some participants were included in the analysis twice (in the group they were originally assigned to, and the group that they transferred to). Outcome assessors were likely to be aware of the intervention received by study participants, and no standardised protocol was used to measure blood pressure.
Hafner1982	Biofeedback	Meditation	Office BP							Allocation concealment and randomization were not clearly described. A per protocol analysis was conducted, and the impact of this on the overall result may be substantial as some participants were included in the analysis twice (in the group they were originally assigned to, and the group that they transferred to). Outcome assessors were likely to be aware of the intervention received by study participants, and no standardised protocol was used to measure blood pressure.
Haghighat2021	Psychotherapy	No intervention/ Standard care	not reported							Allocation concealment and randomization were not clearly described. Outcome assessors were likely to be aware of the intervention received by study participants, and no standardised protocol was used to measure blood pressure.
Hagins2014	Meditative movement	Non-specific comparator	Ambulatory 24 hour BP							Some data were missing, but this was not considered likely to be due to the true value of participants blood pressure.
Henderson1998	Biofeedback	Non-specific comparator	Office BP (attended)							Allocation concealment and randomization were not clearly described. Outcome assessors were likely to be aware of the intervention received by study participants. However, the use of a standardised blood pressure measuring protocol is likely to have minimised the risk of bias.

HernandezReif2000	Progressive muscle relaxation	Massage	Office BP (attended)	Allocation concealment was not clearly described. A per protocol analysis was conducted, and the impact of this on the overall result may be substantial due to the number of participants excluded (5/35).Outcome assessors were likely to be aware of the intervention received by study participants. However, the use of a standardised blood pressure measuring protocol is likely to have minimised the risk of bias.
Hoelscher1986	Progressive muscle relaxation	No intervention/ Standard care	Office BP (attended)	Allocation concealment and randomization were not clearly described. Some data were missing, but this was not considered likely to be due to the true value of participants blood pressure.
Hoelscher1987	Progressive muscle relaxation	No intervention/ Standard care	Office BP (attended)	Allocation concealment and randomization were not clearly described.
Howorka2013	Breathing intervention	No intervention/ Standard care	Ambulatory 24 hour BP	Allocation concealment and randomization were not clearly described.
Huijuan2021	Meditative movement	No intervention/ Standard care	Ambulatory 24 hour BP	Allocation concealment was not clearly described.
Im-Oun2018	Music	No intervention/ Standard care	Home BP	Baseline characteristics of the two groups indicate a problem with the randomization process. Outcome assessors were aware of the intervention received by study participants. However, the use of a standardised blood pressure measuring protocol is likely to have minimised the risk of bias.
Irvine1986	Biofeedback	Non-specific comparator	Home BP	Allocation concealment was not clearly described. Outcome assessors (participants themselves) were likely to be aware of the intervention received, and no standardised protocol was used to measure blood pressure.
Irvine1991	Biofeedback	Non-specific comparator	Office BP (attended)	Allocation concealment was not clearly described.
Ismail2023	Breathing intervention	No intervention/ Standard care	not reported	The method used to measure blood pressure was not described.
Jacob1985	Relaxation + diet	No intervention/ Standard care	Office BP (attended)	Allocation concealment was not clearly described. Participants and carers were aware of the assigned intervention during the trial, and this does appear to have led to deviations from the intended intervention. Eight of the 18 control participants reported engaging in relaxation techniques during the trial. Some data were missing, but this was not considered likely to be due to the true value of participants blood pressure.
Jacob1992	Biofeedback	Non-specific comparator	Ambulatory 24 hour BP	Allocation concealment and randomization were not clearly described. Outcomes were measured by an automated ambulatory method without involvement of outcome assessors, but unblinded study authors then processed the data to remove artifacts, which could be subject to bias.
Jones2010	Breathing intervention	No intervention/ Standard care	Home BP	Allocation concealment was not clearly described. A per protocol analysis was conducted, and the impact of this on the overall result may be substantial due to the number of participants excluded. Outcome assessors were likely to be aware of the intervention received by study participants. However, the use of a standardised blood pressure measuring protocol is likely to have minimised the risk of bias.
Jorgensen1981	Progressive muscle relaxation	No intervention/ Standard care	Office BP (attended)	Allocation concealment and randomization were not clearly described. Outcome assessors were likely to be aware of the intervention received by study participants. However, the use of a standardised blood pressure measuring protocol is likely to have minimised the risk of bias.

Kalmatayeva2014	Relaxation + medication	Medication	not reported							Allocation concealment and randomization were not clearly described. The method used to measure blood pressure was not described. Outcome assessors were likely to be aware of the intervention received by study participants, and no standardised protocol was used to measure blood pressure. No pre-specified analysis plan was available. The result may have been selected on the basis of the results because of incomplete/missing results reporting for timepoints of interest.
Katsarou2014	Relaxation + diet	No intervention/ Standard care	Office BP (attended)							Allocation concealment and randomization were not clearly described. A per protocol analysis was conducted, and the impact of this on the overall result may be substantial due to the number of participants excluded.
Kow2018	Breathing intervention	Music	Office BP (attended)							Some data were missing, but this was not considered likely to be due to the true value of participants blood pressure.
Kretzer2013	Psychotherapy	Home BP monitoring	Home BP							Allocation concealment was not clearly described. Some data were missing, and there was no information available to assess whether this may be related to the blood pressure. Outcome assessors were likely to be aware of the intervention received by study participants, and no standardised protocol was used to measure blood pressure. No pre-specified analysis plan was available. There was insufficient information to assess whether the result may have been selected preferentially.
Kumar2017	Mindfulness	No intervention/ Standard care	Office BP (attended)							Allocation concealment and randomization were not clearly described. A per protocol analysis was conducted, but the impact of this on the overall result was considered to be small. Outcome assessors were likely to be aware of the intervention received by study participants. However, the use of a standardised blood pressure measuring protocol is likely to have minimised the risk of bias.
Kunikullaya2015	Relaxation + lifestyle intervention	Lifestyle intervention	Ambulatory 24 hour BP							No pre-specified analysis plan was available (only retrospective trial registration). The result may have been selected on the basis of the results because there is a discrepancy between two articles based on the same study, reporting identical numerical outcome data for different numbers of participants.
Lagrone1988	Lifestyle intervention	No intervention/ Standard care	Office BP (attended)							Allocation concealment was not clearly described. A per protocol analysis was conducted, and the impact of this on the overall result may be substantial as a considerable proportion of participants were excluded due to non-compliance.
Lagrone1988	Relaxation + lifestyle intervention	No intervention/ Standard care	Office BP (attended)							Allocation concealment was not clearly described. A per protocol analysis was conducted, and the impact of this on the overall result may be substantial as a considerable proportion of participants were excluded due to non-compliance.
Lagrone1988	Relaxation + lifestyle intervention	Lifestyle intervention	Office BP (attended)							Allocation concealment was not clearly described. A per protocol analysis was conducted, and the impact of this on the overall result may be substantial as a considerable proportion of participants were excluded due to non-compliance.
Landman2013	Breathing intervention	Non-specific comparator	Office BP (attended)							Some data were missing, but this was not considered likely to be due to the true value of participants blood pressure.
Lee1988	Relaxation + lifestyle intervention	Lifestyle intervention	not reported							Allocation concealment and randomization were not clearly described. Some data were missing, but this was not considered likely to be due to the true value of participants blood pressure. Outcome assessors were likely to be aware of the intervention received by study participants, and no standardised protocol was used to measure blood pressure.
Lee2003	Meditative movement	No intervention/ Standard care	Office BP (attended)							Allocation concealment was not clearly described. Outcome assessors were likely to be aware of the intervention received by study participants. However, the use of a standardised blood pressure measuring protocol is likely to have minimised the risk of bias.

Lin2022	Meditative movement	No intervention/ Standard care	Office BP (attended)							Allocation concealment and randomization were not clearly described.
Linden2001	Psychotherapy	No intervention/ Standard care	Ambulatory 24 hour BP							Allocation concealment and randomization were not clearly described. Some data were missing, and this was considered likely to be due to the true value of participants blood pressure as 7% of participants (all in the control group) were excluded due to starting BP medication.
Logtenberg2007	Breathing intervention	Music	Home BP							Outcome assessors were likely to be aware of the intervention received by study participants. However, the use of a standardised blood pressure measuring protocol is likely to have minimised the risk of bias.
Lokesh2017	Relaxation + exercise	Exercise	not reported							Allocation concealment and randomization were not clearly described. Outcome assessors were likely to be aware of the intervention received by study participants, and no standardised protocol was used to measure blood pressure.
Loucks2023	Relaxation + Home BP monitoring	Home BP monitoring	Office BP (unattende d)							Participants and carers were aware of the assigned intervention during the trial, and this does appear to have led to deviations from the intended intervention, as some of the control participants reported engaging in mindfulness. However, these deviations were considered unlikely to affect the outcome.
McCaffrey2005	Meditative movement	No intervention/ Standard care	not reported							Allocation concealment was not clearly described. A per protocol analysis was conducted, and the impact of this on the overall result may be substantial due to the number of participants excluded. Some data were missing, but this was not considered likely to be due to the true value of participants blood pressure. Outcome assessors were likely to be aware of the intervention received by study participants, and no standardised protocol was used to measure blood pressure.
McGrady1981	Biofeedback	Non-specific comparator	Office BP (attended)							Allocation concealment and randomization were not clearly described. Imbalance in the number of participants allocated to each group may suggest some problems with randomisation, and baseline characteristics were not presented for all participants randomised. Some data were missing, and this was considered likely to be due to the true value of participants blood pressure, as 7% of randomized participants were excluded due to changes in anti-hypertensive medication. Outcome assessors were likely to be aware of the intervention received by study participants. However, the use of a standardised blood pressure measuring protocol is likely to have minimised the risk of bias.
McGrady1994	Biofeedback	No intervention/ Standard care	Office BP (attended)							Allocation concealment and randomization were not clearly described. Some data were missing, and this was considered likely to be due to the true value of participants blood pressure, as a proportion of participants was excluded due to changes in anti-hypertensive medication (not specified, but between 1-26%). Outcome assessors were likely to be aware of the intervention received by study participants, and no standardised protocol was used to measure blood pressure.
Misra2019	Breathing intervention	No intervention/ Standard care	Office BP (attended)							Allocation concealment was not clearly described. Baseline characteristics of the two groups indicate a problem with the randomization process. Some data were missing, and there was no information available to assess whether this may be related to the blood pressure.
Modesti2015	Breathing intervention	Music	Ambulatory 24 hour BP							Allocation concealment and randomization were not clearly described. Some data were missing, but this was not considered likely to be due to the true value of participants blood pressure.
MohammedElsheikh2 023	Breathing intervention	Breathing intervention	not reported							Allocation concealment and randomization were not clearly described. Outcome assessors were likely to be aware of the intervention received by study participants, and no standardised protocol was used to measure blood pressure.

Mohebbi2014	Massage	Non-specific comparator	Office BP (attended)							Allocation concealment was not clearly described. Outcome assessors were likely to be aware of the intervention received by study participants, and no standardised protocol was used to measure blood pressure.
Momeni2016	Mindfulness	No intervention/ Standard care	Office BP							Allocation concealment was not clearly described. No pre-specified analysis plan was available, as blood pressure appears to have been added to the trial registration retrospectively. There was insufficient information to assess whether the result may have been selected preferentially.
Murugesan2000	Meditative movement	No intervention/ Standard care	Office BP (attended)							Allocation concealment and randomization were not clearly described. Some data were missing, but this was not considered likely to be due to the true value of participants blood pressure. Outcome assessors were likely to be aware of the intervention received by study participants, and no standardised protocol was used to measure blood pressure.
Murugesan2000	Meditative movement	Medication	Office BP (attended)							Allocation concealment and randomization were not clearly described. Some data were missing, but this was not considered likely to be due to the true value of participants blood pressure. Outcome assessors were likely to be aware of the intervention received by study participants, and no standardised protocol was used to measure blood pressure.
Murugesan2000	Medication	No intervention/ Standard care	Office BP (attended)							Allocation concealment and randomization were not clearly described. Some data were missing, but this was not considered likely to be due to the true value of participants blood pressure. Outcome assessors were likely to be aware of the intervention received by study participants, and no standardised protocol was used to measure blood pressure.
Nakao1997	Relaxation + Home BP monitoring	Home BP monitoring	Home BP							Allocation concealment and randomization were not clearly described. Outcome assessors were likely to be aware of the intervention received by study participants, and no standardised protocol was used to measure blood pressure.
Nakao2000	Relaxation + Home BP monitoring	Home BP monitoring	Home BP							Allocation concealment and randomization were not clearly described. Outcome assessors were likely to be aware of the intervention received by study participants, and no standardised protocol was used to measure blood pressure.
Nejati2015	Mindfulness	No intervention/ Standard care	not reported							Allocation concealment and randomization were not clearly described. A per protocol analysis was conducted, and the impact of this on the overall result may be substantial, as no details are provided on the number of participants who were excluded. Outcome assessors were likely to be aware of the intervention received by study participants, and no standardised protocol was used to measure blood pressure.
Nolan2010	Biofeedback	Autogenic training	Ambulatory 24 hour BP							Allocation concealment was not clearly described.
Olson2010	Biofeedback	No intervention/ Standard care	Office BP (attended)							Outcome assessors were likely to be aware of the intervention received by study participants. However, the use of a standardised blood pressure measuring protocol is likely to have minimised the risk of bias.
Palomba2011	Relaxation + Home BP monitoring	Home BP monitoring	Home BP							Allocation concealment and randomization were not clearly described. Outcome assessors were likely to be aware of the intervention received by study participants. However, the use of a standardised blood pressure measuring protocol is likely to have minimised the risk of bias.
Pandey2023	Relaxation + exercise	Exercise	Office BP (attended)							Allocation concealment and randomization were not clearly described. Outcome assessors were likely to be aware of the intervention received by study participants. However, the use of a standardised blood pressure measuring protocol is likely to have minimised the risk of bias.
Pandic2008	Breathing intervention	Music	Office BP (attended)							Outcome assessors were likely to be aware of the intervention received by study participants, and no standardised protocol was used to measure blood pressure.

Park2017	Meditative movement	No intervention/ Standard care	Office BP (attended)							Some data were missing, but this was not considered likely to be due to the true value of participants blood pressure. Outcome assessors were likely to be aware of the intervention received by study participants, and no standardised protocol was used to measure blood pressure.
Patel1975	Biofeedback	Non-specific comparator	Office BP (attended)							Allocation concealment and randomization were not clearly described.
Patel1988	Biofeedback	Medication	Office BP (attended)							Allocation concealment was not clearly described. Baseline characteristics of the two groups indicate a problem with the randomization process. Some data were missing, and there was no information available to assess whether this may be related to the blood pressure. Outcome assessors were likely to be aware of the intervention received by study participants, and no standardised protocol was used to measure blood pressure.
Patel1988	Biofeedback	No intervention/ Standard care	Office BP (attended)							Allocation concealment was not clearly described. Baseline characteristics of the two groups indicate a problem with the randomization process. Some data were missing, and there was no information available to assess whether this may be related to the blood pressure. Outcome assessors were likely to be aware of the intervention received by study participants, and no standardised protocol was used to measure blood pressure.
Patel1988	Biofeedback	Non-specific comparator	Office BP (attended)							Allocation concealment was not clearly described. Baseline characteristics of the two groups indicate a problem with the randomization process. Some data were missing, and there was no information available to assess whether this may be related to the blood pressure. Outcome assessors were likely to be aware of the intervention received by study participants, and no standardised protocol was used to measure blood pressure.
Patel1988	Biofeedback	Relaxation + medication	Office BP (attended)							Allocation concealment was not clearly described. Baseline characteristics of the two groups indicate a problem with the randomization process. Some data were missing, and there was no information available to assess whether this may be related to the blood pressure. Outcome assessors were likely to be aware of the intervention received by study participants, and no standardised protocol was used to measure blood pressure.
Patel1988	Relaxation + medication	Medication	Office BP (attended)							Allocation concealment was not clearly described. Baseline characteristics of the two groups indicate a problem with the randomization process. Some data were missing, and there was no information available to assess whether this may be related to the blood pressure. Outcome assessors were likely to be aware of the intervention received by study participants, and no standardised protocol was used to measure blood pressure.
Patel1988	Relaxation + medication	No intervention/ Standard care	Office BP (attended)							Allocation concealment was not clearly described. Baseline characteristics of the two groups indicate a problem with the randomization process. Some data were missing, and there was no information available to assess whether this may be related to the blood pressure. Outcome assessors were likely to be aware of the intervention received by study participants, and no standardised protocol was used to measure blood pressure.
Patel1988	Relaxation + medication	Non-specific comparator	Office BP (attended)							Allocation concealment was not clearly described. Baseline characteristics of the two groups indicate a problem with the randomization process. Some data were missing, and there was no information available to assess whether this may be related to the blood pressure. Outcome assessors were likely to be aware of the intervention received by study participants, and no standardised protocol was used to measure blood pressure.
Patel1988	No intervention/Standard care	Medication	Office BP (attended)							Allocation concealment was not clearly described. Baseline characteristics of the two groups indicate a problem with the randomization process. Some data were missing, and there was no information available to assess whether this may be related to the blood pressure. Outcome assessors were likely to be aware of the intervention received by study participants, and no standardised protocol was used to measure blood pressure.

Patel1988	No intervention/Standard care	Non-specific comparator	Office BP (attended)							Allocation concealment was not clearly described. Baseline characteristics of the two groups indicate a problem with the randomization process. Some data were missing, and there was no information available to assess whether this may be related to the blood pressure. Outcome assessors were likely to be aware of the intervention received by study participants, and no standardised protocol was used to measure blood pressure.
Patel1988	Non-specific comparator	Medication	Office BP (attended)							Allocation concealment was not clearly described. Baseline characteristics of the two groups indicate a problem with the randomization process. Some data were missing, and there was no information available to assess whether this may be related to the blood pressure. Outcome assessors were likely to be aware of the intervention received by study participants, and no standardised protocol was used to measure blood pressure.
Pathan2023	Multicomponent intervention	No intervention/Standard care	Office BP (attended)							Allocation concealment was not clearly described.
Pathan2023	Multicomponent intervention	Breathing intervention	Office BP (attended)							Allocation concealment was not clearly described.
Pathan2023	Multicomponent intervention	Progressive muscle relaxation	Office BP (attended)							Allocation concealment was not clearly described.
Pathan2023	Progressive muscle relaxation	No intervention/Standard care	Office BP (attended)							Allocation concealment was not clearly described.
Pathan2023	Progressive muscle relaxation	Breathing intervention	Office BP (attended)							Allocation concealment was not clearly described.
Pathan2023	Breathing intervention	No intervention/Standard care	Office BP (attended)							Allocation concealment was not clearly described.
Patil2014	Meditative movement	Non-specific comparator	Office BP (attended)							Allocation concealment was not clearly described. Outcome assessors were likely to be aware of the intervention received by study participants. However, the use of a standardised blood pressure measuring protocol is likely to have minimised the risk of bias.
Perez2009	Psychotherapy	Medication	Ambulatory 24 hour BP							A per protocol analysis was conducted, but the impact of this on the overall result was considered to be small. Some data were missing, but this was not considered likely to be due to the true value of participants blood pressure. No pre-specified analysis plan was available. The result may have been selected on the basis of the results because the retrospective trial registration lists daytime ambulatory BP as primary outcome. This is not reported in the article - instead, 24h ambulatory BP is presented as the primary outcome.
Plaugher2002	Massage	Non-specific comparator	Office BP (attended)							Participants and carers were aware of the assigned intervention during the trial, and this does appear to have led to deviations from the intended intervention. Some participants left the study due to dissatisfaction with their group allocation. Some data were missing, and this was considered likely to be due to the true value of participants blood pressure as some exclusions were due to the need for anti-hypertensive medication.

PonteMarquez2019	Mindfulness	Lifestyle intervention	Office BP (attended)							Allocation concealment and randomization were not clearly described. Some data were missing, but this was not considered likely to be due to the true value of participants blood pressure. Outcome assessors were likely to be aware of the intervention received by study participants. However, the use of a standardised blood pressure measuring protocol is likely to have minimised the risk of bias.
Punita2016	Meditative movement	No intervention/ Standard care	Office BP							A per protocol analysis was conducted, but the impact of this on the overall result was considered to be small. Some data were missing, and there was no information available to assess whether this may be related to the blood pressure. Outcome assessors were likely to be aware of the intervention received by study participants, and no standardised protocol was used to measure blood pressure.
Ranjbar2007	Progressive muscle relaxation	No intervention/ Standard care	Office BP (attended)							Allocation concealment and randomization were not clearly described. A per protocol analysis was conducted, but the impact of this on the overall result was considered to be small. Some data were missing, and there was no information available to assess whether this may be related to the blood pressure.
Rudy1995	Psychotherapy	Non-specific comparator	not reported							Allocation concealment and randomization were not clearly described. Some data were missing, and this was considered likely to be due to the true value of participants blood pressure, as some participants were excluded due to hypertension. Outcome assessors were likely to be aware of the intervention received by study participants, and no standardised protocol was used to measure blood pressure. No pre-specified analysis plan was available. There was insufficient information to assess whether the result may have been selected preferentially (three different intervention groups were combined for analysis, and it is unclear whether this was pre-specified).
Saensak2013	Progressive muscle relaxation	Lifestyle intervention	Office BP (attended)							Some data were missing, and there was no information available to assess whether this may be related to the blood pressure. Outcome assessors were likely to be aware of the intervention received by study participants, and no standardised protocol was used to measure blood pressure.
Sangthong2016	Relaxation + Home BP monitoring	Home BP monitoring	Home BP							Allocation concealment and randomization were not clearly described. A per protocol analysis was conducted, but the impact of this on the overall result was considered to be small. Outcome assessors were likely to be aware of the intervention received by study participants, and no standardised protocol was used to measure blood pressure.
Saptharishi2009	Meditative movement	No intervention/ Standard care	Office BP (attended)							Allocation concealment was not clearly described. Participants and carers were aware of the assigned intervention during the trial, but this does not appear to have led to deviations from the intended intervention. Multiple participants dropped out of the study and were excluded from the analysis but no reasons were provided. It is not clear whether this was due to the use of a per-protocol analysis.<br>Some data were missing, and there was no information available to assess whether this may be related to the blood pressure. Outcome assessors were likely to be aware of the intervention received by study participants, and no standardised protocol was used to measure blood pressure.
Saptharishi2009	Meditative movement	Exercise	Office BP (attended)							Allocation concealment was not clearly described. Participants and carers were aware of the assigned intervention during the trial, but this does not appear to have led to deviations from the intended intervention. Multiple participants dropped out of the study and were excluded from the analysis but no reasons were provided. It is not clear whether this was due to the use of a per-protocol analysis.<br>Some data were missing, and there was no information available to assess whether this may be related to the blood pressure. Outcome assessors were likely to be aware of the intervention received by study participants, and no standardised protocol was used to measure blood pressure.

Saptharishi2009	Meditative movement	Diet	Office BP (attended)							Allocation concealment was not clearly described. Participants and carers were aware of the assigned intervention during the trial, but this does not appear to have led to deviations from the intended intervention. Multiple participants dropped out of the study and were excluded from the analysis but no reasons were provided. It is not clear whether this was due to the use of a per-protocol analysis.<br>Some data were missing, and there was no information available to assess whether this may be related to the blood pressure. Outcome assessors were likely to be aware of the intervention received by study participants, and no standardised protocol was used to measure blood pressure.
Saptharishi2009	Diet	No intervention/ Standard care	Office BP (attended)							Allocation concealment was not clearly described. Participants and carers were aware of the assigned intervention during the trial, but this does not appear to have led to deviations from the intended intervention. Multiple participants dropped out of the study and were excluded from the analysis but no reasons were provided. It is not clear whether this was due to the use of a per-protocol analysis.<br>Some data were missing, and there was no information available to assess whether this may be related to the blood pressure. Outcome assessors were likely to be aware of the intervention received by study participants, and no standardised protocol was used to measure blood pressure.
Saptharishi2009	Diet	Exercise	Office BP (attended)							Allocation concealment was not clearly described. Participants and carers were aware of the assigned intervention during the trial, but this does not appear to have led to deviations from the intended intervention. Multiple participants dropped out of the study and were excluded from the analysis but no reasons were provided. It is not clear whether this was due to the use of a per-protocol analysis.<br>Some data were missing, and there was no information available to assess whether this may be related to the blood pressure. Outcome assessors were likely to be aware of the intervention received by study participants, and no standardised protocol was used to measure blood pressure.
Saptharishi2009	Exercise	No intervention/ Standard care	Office BP (attended)							Allocation concealment was not clearly described. Participants and carers were aware of the assigned intervention during the trial, but this does not appear to have led to deviations from the intended intervention. Multiple participants dropped out of the study and were excluded from the analysis but no reasons were provided. It is not clear whether this was due to the use of a per-protocol analysis.<br>Some data were missing, and there was no information available to assess whether this may be related to the blood pressure. Outcome assessors were likely to be aware of the intervention received by study participants, and no standardised protocol was used to measure blood pressure.
Schein2001	Breathing intervention	Music	Office BP (attended)							Allocation concealment was not clearly described. Multiple participants dropped out of the study and were excluded from the analysis but no reasons were provided. It is not clear whether this was due to the use of a per-protocol analysis. No pre-specified analysis plan was available. The result may have been selected on the basis of the results because this outcome timepoint was selected based on analysis of the data from the participants recruited during the first part of the study.
Schein2009	Breathing intervention	No intervention/ Standard care	Office BP (attended)							The allocation sequence could have been predicted. Although the initial participants appear to have been randomly allocated to groups, drop-outs were replaced by a new participant (for whom the group allocation would have been known). Some data were missing, and this was considered likely to be due to the true value of participants blood pressure, as participants with large fluctuations in blood pressure were excluded. Outcome assessors were likely to be aware of the intervention received by study participants. However, the use of a standardised blood pressure measuring protocol is likely to have minimised the risk of bias.

Schneider1995	Progressive muscle relaxation	Lifestyle intervention	Office BP (attended)							Allocation concealment was not clearly described. No pre-specified analysis plan was available. The result may have been selected on the basis of the results because both office and home BP were measured, but only very limited results are reported for home BP.
Schneider1995	Progressive muscle relaxation	Meditation	Office BP (attended)							Allocation concealment was not clearly described. No pre-specified analysis plan was available. The result may have been selected on the basis of the results because both office and home BP were measured, but only very limited results are reported for home BP.
Schneider1995	Meditation	Lifestyle intervention	Office BP (attended)							Allocation concealment was not clearly described. No pre-specified analysis plan was available. The result may have been selected on the basis of the results because both office and home BP were measured, but only very limited results are reported for home BP.
Schneider2005	Meditation	Lifestyle intervention	Office BP (attended)							Allocation concealment was not clearly described. Some data were missing, and there was no information available to assess whether this may be related to the blood pressure.
Schneider2005	Meditation	Progressive muscle relaxation	Office BP (attended)							Allocation concealment was not clearly described. Some data were missing, and there was no information available to assess whether this may be related to the blood pressure.
Schneider2005	Progressive muscle relaxation	Lifestyle intervention	Office BP (attended)							Allocation concealment was not clearly described. Some data were missing, and there was no information available to assess whether this may be related to the blood pressure.
Seer1980	Meditation	No intervention/ Standard care	Office BP (attended)							Allocation concealment and randomization were not clearly described.
Selvam2020	Meditative movement	No intervention/ Standard care	not reported							Allocation concealment and randomization were not clearly described. The method used to measure blood pressure was not described. No pre-specified analysis plan was available. There was insufficient information to assess whether the result may have been selected preferentially.
Shapiro1997	Psychotherapy	Non-specific comparator	Ambulatory daytime BP							Allocation concealment and randomization were not clearly described. No pre-specified analysis plan was available. There was insufficient information to assess whether the result may have been selected preferentially.
Shetty2017	Breathing intervention	Non-specific comparator	Office BP (attended)							The allocation sequence could have been predicted as it appears that allocation to groups was based on the use of odd and even numbers. No pre-specified analysis plan was available. The result (systolic BP) may have been selected preferentially because diastolic blood pressure is not reported, as the authors state that the difference was non-significant.
Shetty2022	Meditative movement	No intervention/ Standard care	Office BP (attended)							Some data were missing, but this was not considered likely to be due to the true value of participants blood pressure.
Shou2019	Meditative movement	Lifestyle intervention	Office BP (attended)							Allocation concealment and randomization were not clearly described. Some data were missing, and there was no information available to assess whether this may be related to the blood pressure. Outcome assessors were likely to be aware of the intervention received by study participants. However, the use of a standardised blood pressure measuring protocol is likely to have minimised the risk of bias.

Southam1982	Progressive muscle relaxation	No intervention/ Standard care	Ambulatory daytime BP							Allocation concealment was not clearly described. Participants and carers were aware of the assigned intervention during the trial, and this does appear to have led to deviations from the intended intervention. Some participants in the control group took up relaxation techniques, reduced salt in their diet and stopped smoking during the course of the trial. A per protocol analysis was conducted, but the impact of this on the overall result was considered to be small. Some data were missing, and this was considered likely to be due to the true value of participants blood pressure (one participant missing due to a heart attack).
Sujatha2014	Meditative movement	No intervention/ Standard care	Office BP (attended)							Allocation concealment and randomization were not clearly described. Outcome assessors were likely to be aware of the intervention received by study participants, and no standardised protocol was used to measure blood pressure.
Taylor1977	Progressive muscle relaxation	No intervention/ Standard care	Office BP (attended)							Allocation concealment and randomization were not clearly described. Some data were missing, and there was no information available to assess whether this may be related to the blood pressure.
Taylor1977	Progressive muscle relaxation	Non-specific comparator	Office BP (attended)							Allocation concealment and randomization were not clearly described. Some data were missing, and there was no information available to assess whether this may be related to the blood pressure.
Taylor1977	Non-specific comparator	No intervention/ Standard care	Office BP (attended)							Allocation concealment and randomization were not clearly described. Some data were missing, and there was no information available to assess whether this may be related to the blood pressure.
Teng2007	Music	Non-specific comparator	Home BP							Allocation concealment was not clearly described. Outcome assessors were likely to be aware of the intervention received by study participants. However, the use of a standardised blood pressure measuring protocol is likely to have minimised the risk of bias.
Thanalakshmi2020	Breathing intervention	No intervention/ Standard care	Office BP							A per protocol analysis was conducted, and the impact of this on the overall result may be substantial as the additional ITT analysis shows smaller intervention effects. Some data were missing, but this was not considered likely to be due to the true value of participants blood pressure. Outcome assessors were likely to be aware of the intervention received by study participants. However, the use of a standardised blood pressure measuring protocol is likely to have minimised the risk of bias.
TolbanosRoche2014	Meditative movement	No intervention/ Standard care	Office BP (attended)							Allocation concealment and randomization were not clearly described. Multiple participants dropped out of the study and were excluded from the analysis but no reasons were provided. It is not clear whether this was due to the use of a per-protocol analysis. Some data were missing, and this was considered likely to be due to the true value of participants blood pressure as 8% of participants were excluded due to changes in medication or diagnosis. Outcome assessors were likely to be aware of the intervention received by study participants, and no standardised protocol was used to measure blood pressure.
TolbanosRoche2017	Meditative movement	Lifestyle intervention	Office BP (attended)							Allocation concealment and randomization were not clearly described. Multiple participants dropped out of the study and were excluded from the analysis but no reasons were provided. It is not clear whether this was due to the use of a per-protocol analysis. Some data were missing, and there was no information available to assess whether this may be related to the blood pressure. Outcome assessors were likely to be aware of the intervention received by study participants, and no standardised protocol was used to measure blood pressure.

TolbanosRoche2017	Meditative movement	Meditation	Office BP (attended)							Allocation concealment and randomization were not clearly described. Multiple participants dropped out of the study and were excluded from the analysis but no reasons were provided. It is not clear whether this was due to the use of a per-protocol analysis. Some data were missing, and there was no information available to assess whether this may be related to the blood pressure. Outcome assessors were likely to be aware of the intervention received by study participants, and no standardised protocol was used to measure blood pressure.
TolbanosRoche2017	Meditative movement	Breathing intervention	Office BP (attended)							Allocation concealment and randomization were not clearly described. Multiple participants dropped out of the study and were excluded from the analysis but no reasons were provided. It is not clear whether this was due to the use of a per-protocol analysis. Some data were missing, and there was no information available to assess whether this may be related to the blood pressure. Outcome assessors were likely to be aware of the intervention received by study participants, and no standardised protocol was used to measure blood pressure.
TolbanosRoche2017	Breathing intervention	Lifestyle intervention	Office BP (attended)							Allocation concealment and randomization were not clearly described. Multiple participants dropped out of the study and were excluded from the analysis but no reasons were provided. It is not clear whether this was due to the use of a per-protocol analysis. Some data were missing, and there was no information available to assess whether this may be related to the blood pressure. Outcome assessors were likely to be aware of the intervention received by study participants, and no standardised protocol was used to measure blood pressure.
TolbanosRoche2017	Breathing intervention	Meditation	Office BP (attended)							Allocation concealment and randomization were not clearly described. Multiple participants dropped out of the study and were excluded from the analysis but no reasons were provided. It is not clear whether this was due to the use of a per-protocol analysis. Some data were missing, and there was no information available to assess whether this may be related to the blood pressure. Outcome assessors were likely to be aware of the intervention received by study participants, and no standardised protocol was used to measure blood pressure.
TolbanosRoche2017	Meditation	Lifestyle intervention	Office BP (attended)							Allocation concealment and randomization were not clearly described. Multiple participants dropped out of the study and were excluded from the analysis but no reasons were provided. It is not clear whether this was due to the use of a per-protocol analysis. Some data were missing, and there was no information available to assess whether this may be related to the blood pressure. Outcome assessors were likely to be aware of the intervention received by study participants, and no standardised protocol was used to measure blood pressure.
Tosi1992	Multicomponent intervention	Non-specific comparator	Office BP (attended)							Allocation concealment and randomization were not clearly described. Some data were missing, and this was considered likely to be due to the true value of participants blood pressure as 5% of participants were excluded due to extreme BP elevations.
Tosi1992	Multicomponent intervention	Psychotherapy	Office BP (attended)							Allocation concealment and randomization were not clearly described. Some data were missing, and this was considered likely to be due to the true value of participants blood pressure as 5% of participants were excluded due to extreme BP elevations.
Tosi1992	Multicomponent intervention	Hypnosis	Office BP (attended)							Allocation concealment and randomization were not clearly described. Some data were missing, and this was considered likely to be due to the true value of participants blood pressure as 5% of participants were excluded due to extreme BP elevations.
Tosi1992	Hypnosis	Non-specific comparator	Office BP (attended)							Allocation concealment and randomization were not clearly described. Some data were missing, and this was considered likely to be due to the true value of participants blood pressure as 5% of participants were excluded due to extreme BP elevations.
Tosi1992	Hypnosis	Psychotherapy	Office BP (attended)							Allocation concealment and randomization were not clearly described. Some data were missing, and this was considered likely to be due to the true value of participants blood pressure as 5% of participants were excluded due to extreme BP elevations.

Tosi1992	Psychotherapy	Non-specific comparator	Office BP (attended)							Allocation concealment and randomization were not clearly described. Some data were missing, and this was considered likely to be due to the true value of participants blood pressure as 5% of participants were excluded due to extreme BP elevations.
Tsai2003	Meditative movement	No intervention/ Standard care	Office BP (attended)							Allocation concealment was not clearly described.
Tsai2007	Biofeedback	Non-specific comparator	Office BP							Baseline characteristics of the two groups indicate a problem with the randomization process. Some data were missing, and this was considered likely to be due to the true value of participants blood pressure, as two participants were excluded due to taking anti-hypertensive medication. No pre-specified analysis plan was available. The outcomes may have been selected on the basis of the results because only systolic blood pressure was reported (no diastolic data).
Venturelli2015	Breathing intervention	No intervention/ Standard care	Office BP (attended)							Allocation concealment was not clearly described.
Venturelli2015	Breathing intervention	Exercise	Office BP (attended)							Allocation concealment was not clearly described.
Venturelli2015	Exercise	No intervention/ Standard care	Office BP (attended)							Allocation concealment was not clearly described.
Wadden1984	Psychotherapy	Relaxation + lifestyle intervention	Office BP (attended)							Allocation concealment was not clearly described. A per protocol analysis was conducted, and the impact of this on the overall result may be substantial because 10% of participants were excluded due to non-adherence. Some data were missing, and this was considered likely to be due to the true value of participants blood pressure because 3 participants were excluded due to having BP lower than a certain threshold (different from eligibility criteria). Outcome assessors were likely to be aware of the intervention received by study participants. However, the use of a standardised blood pressure measuring protocol is likely to have minimised the risk of bias. No pre-specified analysis plan was available. The result may have been selected on the basis of the results because only 2/3 weeks of the baseline period were selected to estimate baseline BP.
Walsh1977	Progressive muscle relaxation	Biofeedback	not reported							Allocation concealment was not clearly described. The method used to measure blood pressure was not described.
Wang2016	Biofeedback	Non-specific comparator	Office BP							A per protocol analysis was conducted, and the impact of this on the overall result may be substantial as a considerable proportion of participants was excluded (19%), even those who did provide outcome data at this timepoint. Some data were missing, and this was considered likely to be due to the true value of participants blood pressure because one participant was excluded due to starting anti-hypertensive medication. No pre-specified analysis plan was available. The result may have been selected on the basis of the results because an unusual analysis approach is reported, where participants who did not complete the later outcome assessment timepoint were excluded from the analysis for the earlier timepoint.
Webb2006	Psychotherapy	Non-specific comparator	Office BP							Allocation concealment and randomization were not clearly described. Outcome assessors were likely to be aware of the intervention received by study participants. However, the use of a standardised blood pressure measuring protocol is likely to have minimised the risk of bias.

Webb2006	Psychotherapy	Multicomponent intervention	Office BP							Allocation concealment and randomization were not clearly described. Outcome assessors were likely to be aware of the intervention received by study participants. However, the use of a standardised blood pressure measuring protocol is likely to have minimised the risk of bias.
Webb2006	Multicomponent intervention	Non-specific comparator	Office BP							Allocation concealment and randomization were not clearly described. Outcome assessors were likely to be aware of the intervention received by study participants. However, the use of a standardised blood pressure measuring protocol is likely to have minimised the risk of bias.
Wen2021	Meditative movement	Meditative movement	Office BP (attended)							Outcome assessors were likely to be aware of the intervention received by study participants. However, the use of a standardised blood pressure measuring protocol is likely to have minimised the risk of bias. The result may have been selected preferentially because the article only reports post-treatment 6 weeks timepoint, while the trial registration defined the primary BP outcome as being assessed at 6 and 12 weeks.
Wolff2016	Meditative movement	No intervention/ Standard care	Office BP (attended)							Allocation concealment was not clearly described. Some data were missing, but this was not considered likely to be due to the true value of participants blood pressure.
Wright2021	Relaxation + diet	No intervention/ Standard care	Office BP (attended)							Allocation concealment and randomization were not clearly described. Participants and carers were aware of the assigned intervention during the trial, and this does appear to have led to deviations from the intended intervention - one participant appears to have refused the randomisation, but this was considered unlikely to affect the outcome. A per protocol analysis was conducted, but the impact of this on the overall result was considered to be small. Some data were missing, and there was no information available to assess whether this may be related to the blood pressure. Outcome assessors were likely to be aware of the intervention received by study participants. However, the use of a standardised blood pressure measuring protocol is likely to have minimised the risk of bias.
Wright2021	Relaxation + diet	Non-specific comparator	Office BP (attended)							Allocation concealment and randomization were not clearly described. Participants and carers were aware of the assigned intervention during the trial, and this does appear to have led to deviations from the intended intervention - one participant appears to have refused the randomisation, but considering it was only a single participant, it was considered unlikely to affect the outcome. A per protocol analysis was conducted, but the impact of this on the overall result was considered to be small. Outcome assessors were likely to be aware of the intervention received by study participants. However, the use of a standardised blood pressure measuring protocol is likely to have minimised the risk of bias.
Wright2021	Non-specific comparator	No intervention/ Standard care	Office BP (attended)							Allocation concealment and randomization were not clearly described. Some data were missing, and there was no information available to assess whether this may be related to the blood pressure. Outcome assessors were likely to be aware of the intervention received by study participants. However, the use of a standardised blood pressure measuring protocol is likely to have minimised the risk of bias.
Wu2023	Meditative movement	Exercise	Office BP (attended)							A per protocol analysis was conducted, but the impact of this on the overall result was considered to be small. Outcome assessors were likely to be aware of the intervention received by study participants, and no standardised protocol was used to measure blood pressure. No pre-specified analysis plan was available. The result may have been selected on the basis of the results because inconsistencies in the reported data (across different tables/figures) suggest that multiple analyses may have been conducted and were not explicitly described.

Yan2022	Meditative movement	No intervention/ Standard care	Office BP (attended)							A per protocol analysis was conducted, and the impact of this on the overall result may be substantial due to a considerable proportion of participants excluded due to non-adherence. Some data were missing, but this was not considered likely to be due to the true value of participants blood pressure. Outcome assessors were likely to be aware of the intervention received by study participants, and no standardised protocol was used to measure blood pressure - blinding was unclear and it is possible that measurement may have differed between groups.
Yan2022	Meditative movement	Non-specific comparator	Office BP (attended)							A per protocol analysis was conducted, and the impact of this on the overall result may be substantial due to a considerable proportion of participants excluded due to non-adherence. Some data were missing, but this was not considered likely to be due to the true value of participants blood pressure. Outcome assessors were likely to be aware of the intervention received by study participants, and no standardised protocol was used to measure blood pressure - blinding was unclear and it is possible that measurement may have differed between groups.
Yan2022	Non-specific comparator	No intervention/ Standard care	Office BP (attended)							A per protocol analysis was conducted, and the impact of this on the overall result may be substantial due to a considerable proportion of participants excluded due to non-adherence. Some data were missing, but this was not considered likely to be due to the true value of participants blood pressure. Outcome assessors were likely to be aware of the intervention received by study participants, and no standardised protocol was used to measure blood pressure - blinding was unclear and it is possible that measurement may have differed between groups.
Yau2022	Relaxation + diet	No intervention/ Standard care	Office BP (attended)							Allocation concealment was not clearly described. Some data were missing, but this was not considered likely to be due to the true value of participants blood pressure.
Yau2022	Relaxation + diet	Diet	Office BP (attended)							Allocation concealment was not clearly described.
Yau2022	Diet	No intervention/ Standard care	Office BP (attended)							Allocation concealment was not clearly described. Some data were missing, but this was not considered likely to be due to the true value of participants blood pressure.
Young1999	Meditative movement	Exercise	Office BP (attended)							Allocation concealment was not clearly described. The allocation sequence could have been predicted as a fixed block size was used and it is not clear if the size was known to study staff. Some data were missing, but this was not considered likely to be due to the true value of participants blood pressure.
Yuenyongchaiwat2024	Breathing intervention	Non-specific comparator	not reported							Allocation concealment was not clearly described. Some data were missing, but this was not considered likely to be due to the true value of participants blood pressure. The method used to measure blood pressure was not described.
Yung2001	Autogenic training	Progressive muscle relaxation	Office BP (attended)							Allocation concealment and randomization were not clearly described. Baseline characteristics of the two groups indicate a problem with the randomization process. Outcome assessors were likely to be aware of the intervention received by study participants, and no standardised protocol was used to measure blood pressure. The result may have been selected on the basis of the results because both home and office BP were measured but only office data were reported.
Zanini2009	Music	No intervention/ Standard care	Office BP							Allocation concealment was not clearly described. Outcome assessors were likely to be aware of the intervention received by study participants, and no standardised protocol was used to measure blood pressure.
Zurawski1987	Multicomponent intervention	Biofeedback	Office BP							Allocation concealment was not clearly described. Some data were missing, but this was not considered likely to be due to the true value of participants blood pressure. Outcome assessors were likely to be aware of the intervention received by study participants. However, the use of a standardised blood pressure measuring protocol is likely to have minimised the risk of bias.

Table S21: Hypertension, short follow-up, cluster-randomised trials

Study	Intervention	Comparator	Outcome measure	D1a	D1b	D2	D3	D4	D5	Overall	Description of concerns
Hasandokht2015	Relaxation + lifestyle intervention	No intervention/standard care	Office BP (attended)								Allocation concealment and randomization were not clearly described. Some data were missing, but this was not considered likely to be due to the true value of participants blood pressure.
Kohn2023a	Meditative movement	Lifestyle intervention	Office BP (attended)								Allocation concealment was not clearly described. Some data were missing, but this was not considered likely to be due to the true value of participants blood pressure.
Yen1996	Multicomponent intervention	No intervention/standard care	Home BP								Allocation concealment and randomization were not clearly described. Some data were missing, and this was considered likely to be due to the true value of participants blood pressure as those who dropped out had lower baseline blood pressure than those who completed the trial.
Yen1996	Multicomponent intervention	Lifestyle intervention	Home BP								Allocation concealment and randomization were not clearly described. Some data were missing, and this was considered likely to be due to the true value of participants blood pressure as those who dropped out had lower baseline blood pressure than those who completed the trial.
Yen1996	Multicomponent intervention	Non-specific comparator	Home BP								Allocation concealment and randomization were not clearly described. Some data were missing, and this was considered likely to be due to the true value of participants blood pressure as those who dropped out had lower baseline blood pressure than those who completed the trial.
Yen1996	Non-specific comparator	No intervention/standard care	Home BP								Allocation concealment and randomization were not clearly described. Some data were missing, and this was considered likely to be due to the true value of participants blood pressure as those who dropped out had lower baseline blood pressure than those who completed the trial.
Yen1996	Non-specific comparator	Lifestyle intervention	Home BP								Allocation concealment and randomization were not clearly described. Some data were missing, and this was considered likely to be due to the true value of participants blood pressure as those who dropped out had lower baseline blood pressure than those who completed the trial.
Yen1996	Lifestyle intervention	No intervention/standard care	Home BP								Allocation concealment and randomization were not clearly described. Some data were missing, and this was considered likely to be due to the true value of participants blood pressure as those who dropped out had lower baseline blood pressure than those who completed the trial.

Table S22: Hypertension population, medium term follow-up, individually randomised trials

Study	Intervention	Comparator	Outcome measure	D1	D2	D3	D4	D5	Overall	Description of concerns
Achmon1989	Psychotherapy	Lifestyle intervention	Office BP (attended)							Allocation concealment and randomization were not clearly described. Some data were missing, but this was not considered likely to be due to the true value of participants blood pressure.
Achmon1989	Biofeedback	Lifestyle intervention	Office BP (attended)							Allocation concealment and randomization were not clearly described. Some data were missing, but this was not considered likely to be due to the true value of participants blood pressure.
Achmon1989	Biofeedback	Psychotherapy	Office BP (attended)							Allocation concealment and randomization were not clearly described. Some data were missing, but this was not considered likely to be due to the true value of participants blood pressure.
Adsett1989	Medication + lifestyle intervention	Lifestyle intervention	Home BP							The allocation sequence could have been predicted as a fixed block size was used and the trial was not blinded. Some data were missing, but this was not considered likely to be due to the true value of participants blood pressure. Outcome assessors were likely to be aware of the intervention received by study participants (although the nurse was stated to be blinded, they were able to correctly guess the group allocation for many participants). However, the use of a standardised blood pressure measuring protocol is likely to have minimised the risk of bias.
Adsett1989	Progressive muscle relaxation	Lifestyle intervention	Home BP							The allocation sequence could have been predicted as a fixed block size was used and the trial was not blinded. Some data were missing, but this was not considered likely to be due to the true value of participants blood pressure.
Adsett1989	Progressive muscle relaxation + medication	Lifestyle intervention	Home BP							The allocation sequence could have been predicted as a fixed block size was used and the trial was not blinded. Some data were missing, but this was not considered likely to be due to the true value of participants blood pressure. Outcome assessors were likely to be aware of the intervention received by study participants (although the nurse was stated to be blinded, they were able to correctly guess the group allocation for many participants). However, the use of a standardised blood pressure measuring protocol is likely to have minimised the risk of bias.
Adsett1989	Progressive muscle relaxation	Medication + lifestyle intervention	Home BP							The allocation sequence could have been predicted as a fixed block size was used and the trial was not blinded. Outcome assessors were likely to be aware of the intervention received by study participants (although the nurse was stated to be blinded, they were able to correctly guess the group allocation for many participants). However, the use of a standardised blood pressure measuring protocol is likely to have minimised the risk of bias.
Adsett1989	Progressive muscle relaxation + medication	Medication + lifestyle intervention	Home BP							The allocation sequence could have been predicted as a fixed block size was used and the trial was not blinded.
Adsett1989	Progressive muscle relaxation + medication	Progressive muscle relaxation	Home BP							The allocation sequence could have been predicted as a fixed block size was used and the trial was not blinded. Outcome assessors were likely to be aware of the intervention received by study participants (although the nurse was stated to be blinded, they were able to correctly guess the group allocation for many participants). However, the use of a standardised blood pressure measuring protocol is likely to have minimised the risk of bias.

Agras1987	Progressive muscle relaxation	No intervention/Standard care	Office BP (attended)							Allocation concealment was not clearly described.
Ahmadpanah2016	Mindfulness	Non-specific comparator	Office BP (attended)							Outcome assessors were likely to be aware of the intervention received by study participants, and no standardised protocol was used to measure blood pressure. The analysis plan outlined on the trial registry site appears to relate to a different trial.
Ahmadpanah2016	Multicomponent relaxation	Non-specific comparator	Office BP (attended)							Outcome assessors were likely to be aware of the intervention received by study participants, and no standardised protocol was used to measure blood pressure. The analysis plan outlined on the trial registry site appears to relate to a different trial.
Ahmadpanah2016	Multicomponent relaxation	Mindfulness	Office BP (attended)							Outcome assessors were likely to be aware of the intervention received by study participants, and no standardised protocol was used to measure blood pressure. The analysis plan outlined on the trial registry site appears to relate to a different trial.
Aivazyan1988a	Non-specific comparator	No intervention/Standard care	Office BP							Allocation concealment and randomization were not clearly described. Multiple participants dropped out of the study and were excluded from the analysis but no reasons were provided. It is not clear whether this was due to the use of a per-protocol analysis. Outcome assessors were likely to be aware of the intervention received by study participants. However, the use of a standardised blood pressure measuring protocol is likely to have minimised the risk of bias. No pre-specified analysis plan was available. There was insufficient information to assess whether the result may have been selected preferentially.
Aivazyan1988a	Autogenic training	No intervention/Standard care	Office BP							Allocation concealment and randomization were not clearly described. Multiple participants dropped out of the study and were excluded from the analysis but no reasons were provided. It is not clear whether this was due to the use of a per-protocol analysis. Outcome assessors were likely to be aware of the intervention received by study participants. However, the use of a standardised blood pressure measuring protocol is likely to have minimised the risk of bias. No pre-specified analysis plan was available. There was insufficient information to assess whether the result may have been selected preferentially.
Aivazyan1988a	Biofeedback	No intervention/Standard care	Office BP							Allocation concealment and randomization were not clearly described. Multiple participants dropped out of the study and were excluded from the analysis but no reasons were provided. It is not clear whether this was due to the use of a per-protocol analysis. Outcome assessors were likely to be aware of the intervention received by study participants. However, the use of a standardised blood pressure measuring protocol is likely to have minimised the risk of bias. No pre-specified analysis plan was available. There was insufficient information to assess whether the result may have been selected preferentially.
Aivazyan1988a	Multicomponent relaxation	No intervention/Standard care	Office BP							Allocation concealment and randomization were not clearly described. Multiple participants dropped out of the study and were excluded from the analysis but no reasons were provided. It is not clear whether this was due to the use of a per-protocol analysis. Outcome assessors were likely to be aware of the intervention received by study participants. However, the use of a standardised blood pressure measuring protocol is likely to have minimised the risk of bias. No pre-specified analysis plan was available. There was insufficient information to assess whether the result may have been selected preferentially.
Aivazyan1988a	Autogenic training	Non-specific comparator	Office BP							Allocation concealment and randomization were not clearly described. Multiple participants dropped out of the study and were excluded from the analysis but no reasons were provided. It is not clear whether this was due to the use of a per-protocol analysis. Outcome assessors were likely to be aware of the intervention received by study participants. However, the use of a standardised blood pressure measuring protocol is likely to have minimised the risk of bias. No

										pre-specified analysis plan was available. There was insufficient information to assess whether the result may have been selected preferentially.
Aivazyan1988a	Biofeedback	Non-specific comparator	Office BP							Allocation concealment and randomization were not clearly described. Multiple participants dropped out of the study and were excluded from the analysis but no reasons were provided. It is not clear whether this was due to the use of a per-protocol analysis. Outcome assessors were likely to be aware of the intervention received by study participants. However, the use of a standardised blood pressure measuring protocol is likely to have minimised the risk of bias. No pre-specified analysis plan was available. There was insufficient information to assess whether the result may have been selected preferentially.
Aivazyan1988a	Multicomponent relaxation	Non-specific comparator	Office BP							Allocation concealment and randomization were not clearly described. Multiple participants dropped out of the study and were excluded from the analysis but no reasons were provided. It is not clear whether this was due to the use of a per-protocol analysis. Outcome assessors were likely to be aware of the intervention received by study participants. However, the use of a standardised blood pressure measuring protocol is likely to have minimised the risk of bias. No pre-specified analysis plan was available. There was insufficient information to assess whether the result may have been selected preferentially.
Aivazyan1988a	Biofeedback	Autogenic training	Office BP							Allocation concealment and randomization were not clearly described. Multiple participants dropped out of the study and were excluded from the analysis but no reasons were provided. It is not clear whether this was due to the use of a per-protocol analysis. Outcome assessors were likely to be aware of the intervention received by study participants. However, the use of a standardised blood pressure measuring protocol is likely to have minimised the risk of bias. No pre-specified analysis plan was available. There was insufficient information to assess whether the result may have been selected preferentially.
Aivazyan1988a	Multicomponent relaxation	Autogenic training	Office BP							Allocation concealment and randomization were not clearly described. Multiple participants dropped out of the study and were excluded from the analysis but no reasons were provided. It is not clear whether this was due to the use of a per-protocol analysis. Outcome assessors were likely to be aware of the intervention received by study participants. However, the use of a standardised blood pressure measuring protocol is likely to have minimised the risk of bias. No pre-specified analysis plan was available. There was insufficient information to assess whether the result may have been selected preferentially.
Aivazyan1988a	Multicomponent relaxation	Biofeedback	Office BP							Allocation concealment and randomization were not clearly described. Multiple participants dropped out of the study and were excluded from the analysis but no reasons were provided. It is not clear whether this was due to the use of a per-protocol analysis. Outcome assessors were likely to be aware of the intervention received by study participants. However, the use of a standardised blood pressure measuring protocol is likely to have minimised the risk of bias. No pre-specified analysis plan was available. There was insufficient information to assess whether the result may have been selected preferentially.
Amigo1997	Exercise	Non-specific comparator	Office BP (attended)							Allocation concealment and randomization were not clearly described. Baseline characteristics of the two groups indicated a problem with the randomization process (much younger participants were assigned to the exercise intervention).Some data were missing, but this was not considered likely to be due to the true value of participants blood pressure. No pre-specified analysis plan was available. There was insufficient information to assess whether the result may have been selected preferentially.

Amigo1997	Progressive muscle relaxation	Non-specific comparator	Office BP (attended)	Allocation concealment and randomization were not clearly described. Baseline characteristics of the two groups indicated a problem with the randomization process (much younger participants were assigned to the exercise intervention). Some data were missing, but this was not considered likely to be due to the true value of participants blood pressure. No pre-specified analysis plan was available. There was insufficient information to assess whether the result may have been selected preferentially.
Amigo1997	Progressive muscle relaxation	Exercise	Office BP (attended)	Allocation concealment and randomization were not clearly described. Baseline characteristics of the two groups indicated a problem with the randomization process (much younger participants were assigned to the exercise intervention). No pre-specified analysis plan was available. There was insufficient information to assess whether the result may have been selected preferentially.
Babak2022	Mindfulness	No intervention/Standard care	Office BP (attended)	Allocation concealment was not clearly described. A per protocol analysis was conducted, but the impact of this on the overall result was considered to be small. Some data were missing, and this was considered likely to be due to the true value of participants blood pressure - two participants who suffered a hypertensive crisis were excluded. Outcome assessors were likely to be aware of the intervention received by study participants. However, the use of a standardised blood pressure measuring protocol is likely to have minimised the risk of bias.
Bennett1991	Multicomponent relaxation	Psychotherapy	Office BP (attended)	Allocation concealment and randomization were not clearly described. Some data were missing, but this was not considered likely to be due to the true value of participants blood pressure. Outcome assessors were likely to be aware of the intervention received by study participants. However, the use of a standardised blood pressure measuring protocol is likely to have minimised the risk of bias. No pre-specified analysis plan was available. There was insufficient information to assess whether the result may have been selected preferentially.
Blanchard1988	Autogenic training	Non-specific comparator	Office BP (attended)	Allocation concealment and randomization were not clearly described. The allocation sequence could have been predicted as several dropouts were replaced by assigning new participants directly into the specific group. Some data were missing, and this was considered likely to be due to the true value of participants blood pressure as participants were excluded if their BP was not under control. Outcome assessors were likely to be aware of the intervention received by study participants, and no standardised protocol was used to measure blood pressure. No pre-specified analysis plan was available. The result may have been purposefully selected because the authors indicate that this timepoint was chosen because half of the sample had their BP under control at that time.
Blanchard1988	Biofeedback	Non-specific comparator	Office BP (attended)	Allocation concealment and randomization were not clearly described. The allocation sequence could have been predicted as several dropouts were replaced by assigning new participants directly into the specific group. Some data were missing, and this was considered likely to be due to the true value of participants blood pressure as participants were excluded if their BP was not under control. Outcome assessors were likely to be aware of the intervention received by study participants, and no standardised protocol was used to measure blood pressure. No pre-specified analysis plan was available. The result may have been purposefully selected because the authors indicate that this timepoint was chosen because half of the sample had their BP under control at that time.
Blanchard1988	Biofeedback	Autogenic training	Office BP (attended)	Allocation concealment and randomization were not clearly described. The allocation sequence could have been predicted as several dropouts were replaced by assigning new participants directly into the specific group. Some data were missing, and this was considered likely to be due to the true value of participants blood pressure as participants were excluded if their BP was not

										under control. Outcome assessors were likely to be aware of the intervention received by study participants, and no standardised protocol was used to measure blood pressure. No pre-specified analysis plan was available. The result may have been purposefully selected because the authors indicate that this timepoint was chosen because half of the sample had their BP under control at that time.
Brauer1979	Progressive muscle relaxation	Non-specific comparator	Office BP (attended)							Allocation concealment and randomization were not clearly described. Some data were missing, but this was not considered likely to be due to the true value of participants blood pressure. No pre-specified analysis plan was available. There was insufficient information to assess whether the result may have been selected preferentially.
Chan2018	Exercise	Non-specific comparator	Office BP							Some data were missing, but this was not considered likely to be due to the true value of participants blood pressure.
Chan2018	Meditative movement	Non-specific comparator	Office BP							Some data were missing, but this was not considered likely to be due to the true value of participants blood pressure.
Chan2018	Meditative movement	Exercise	Office BP							Some data were missing, but this was not considered likely to be due to the true value of participants blood pressure.
Chen2016a	Meditative movement	No intervention/Standard care	not reported							Allocation concealment and randomization were not clearly described. Outcome assessors were likely to be aware of the intervention received by study participants, and no standardised protocol was used to measure blood pressure.
Cheung2005	Meditative movement	Non-specific comparator	Ambulatory 24 hour BP							Allocation concealment and randomization were not clearly described. Baseline differences between groups may suggest problems with randomization. Participants and carers were aware of the assigned intervention during the trial, but this does not appear to have led to deviations from the intended intervention. A per protocol analysis was conducted, and the impact of this on the overall result may be substantial as considerable proportion of participants randomized was not analysed (16%). Some data were missing, but this was not considered likely to be due to the true value of participants blood pressure.
Clemow2018	Psychotherapy	No intervention/Standard care	Office BP (attended)							Outcome assessors were likely to be aware of the intervention received by study participants. However, the use of a standardised blood pressure measuring protocol is likely to have minimised the risk of bias. No pre-specified analysis plan was available, as the trial was retrospectively registered. The result may have been selected on the basis of the results because the outcome was not assessed at the end of the intervention.
Cohen1983	Meditation	Home BP monitoring	Office BP (attended)							Allocation concealment and randomization were not clearly described. Outcome assessors were likely to be aware of the intervention received by study participants, and no standardised protocol was used to measure blood pressure. No pre-specified analysis plan was available. The result may have been selected on the basis of the results because three methods of assessing blood pressure were used, but only one was reported. Despite a blinded outcome measurement being available, this was not prioritised for analysis.
Cohen1983	Biofeedback	Home BP monitoring	Office BP (attended)							Allocation concealment and randomization were not clearly described. Outcome assessors were likely to be aware of the intervention received by study participants, and no standardised protocol was used to measure blood pressure. No pre-specified analysis plan was available. The result may have been selected on the basis of the results because three methods of assessing blood pressure were used, but only one was reported. Despite a blinded outcome measurement being available, this was not prioritised for analysis.
Cohen1983	Biofeedback	Meditation	Office BP (attended)							Allocation concealment and randomization were not clearly described. Outcome assessors were likely to be aware of the intervention received by study participants, and no standardised protocol was used to measure blood pressure. No pre-specified analysis plan was available. The

										result may have been selected on the basis of the results because three methods of assessing blood pressure were used, but only one was reported. Despite a blinded outcome measurement being available, this was not prioritised for analysis.
Cohen2016	Meditative movement	Lifestyle intervention	Ambulatory 24 hour BP							Allocation concealment was not clearly described. Baseline characteristics of the two groups indicate a problem with the randomization process (imbalance in group allocation despite blocked randomisation). Participants and carers were aware of the assigned intervention during the trial, and this does appear to have led to deviations from the intended intervention (different number of participants dropped out due to dissatisfaction with their allocated interventions). Some data were missing, and this was considered likely to be due to the true value of participants blood pressure as some were excluded due to high BP and the need to initiate antihypertensive medication.
Cohen2016	Relaxation + lifestyle intervention	Lifestyle intervention	Ambulatory 24 hour BP							Allocation concealment was not clearly described. Baseline characteristics of the two groups indicate a problem with the randomization process (imbalance in group allocation despite blocked randomisation). Participants and carers were aware of the assigned intervention during the trial, and this does appear to have led to deviations from the intended intervention (participants dropped out due to dissatisfaction with their allocated interventions, but this was balanced across the groups). Some data were missing, and this was considered likely to be due to the true value of participants blood pressure as some were excluded due to high BP and the need to initiate antihypertensive medication.
Cohen2016	Relaxation + lifestyle intervention	Meditative movement	Ambulatory 24 hour BP							Allocation concealment was not clearly described. Baseline characteristics of the two groups indicate a problem with the randomization process (imbalance in group allocation despite blocked randomisation). Participants and carers were aware of the assigned intervention during the trial, and this does appear to have led to deviations from the intended intervention (different number of participants dropped out due to dissatisfaction with their allocated interventions). Some data were missing, and this was considered likely to be due to the true value of participants blood pressure as some were excluded due to high BP and the need to initiate antihypertensive medication.
Cottier1984	Progressive muscle relaxation	Non-specific comparator	Office BP (attended)							Allocation concealment was not clearly described. Some data were missing, and this was considered likely to be due to the true value of participants blood pressure, as participants with high blood pressure were excluded. Outcome assessors were likely to be aware of the intervention received by study participants. However, the use of a standardised blood pressure measuring protocol is likely to have minimised the risk of bias.
Cramer2018	Breathing intervention	No intervention/Standard care	Ambulatory 24 hour BP							Some data were missing, but this was not considered likely to be due to the true value of participants blood pressure.
Cramer2018	Meditative movement	No intervention/Standard care	Ambulatory 24 hour BP							Some data were missing, but this was not considered likely to be due to the true value of participants blood pressure.
Cramer2018	Meditative movement	Breathing intervention	Ambulatory 24 hour BP							Some data were missing, but this was not considered likely to be due to the true value of participants blood pressure.
Dhungana2021	Relaxation + lifestyle intervention	Lifestyle intervention	Office BP (attended)							Outcome assessors were likely to be aware of the intervention received by study participants. However, the use of a standardised blood pressure measuring protocol is likely to have minimised the risk of bias.

Drazen1982	Progressive muscle relaxation	Lifestyle intervention	Office BP (attended)							Baseline characteristics of the two groups indicate a problem with the randomization process. Outcome assessors were likely to be aware of the intervention received by study participants, and no standardised protocol was used to measure blood pressure. No pre-specified analysis plan was available. There was insufficient information to assess whether the result may have been selected preferentially.
Drazen1982	Psychotherapy	Lifestyle intervention	Office BP (attended)							Baseline characteristics of the two groups indicate a problem with the randomization process. Outcome assessors were likely to be aware of the intervention received by study participants, and no standardised protocol was used to measure blood pressure. No pre-specified analysis plan was available. There was insufficient information to assess whether the result may have been selected preferentially.
Drazen1982	Psychotherapy	Progressive muscle relaxation	Office BP (attended)							Baseline characteristics of the two groups indicate a problem with the randomization process. Outcome assessors were likely to be aware of the intervention received by study participants, and no standardised protocol was used to measure blood pressure. No pre-specified analysis plan was available. There was insufficient information to assess whether the result may have been selected preferentially.
Elavally2022	Biofeedback	No intervention/Standard care	Office BP (attended)							Allocation concealment was not clearly described. Some data were missing, and this was considered likely to be due to the true value of participants blood pressure, as those who required a change in antihypertensive medication were excluded from the analysis.
Frankel1978	Biofeedback	Non-specific comparator	Office BP (attended)							Allocation concealment was not clearly described.
Friedman1977a	Biofeedback	Hypnosis	Office BP (attended)							Allocation concealment and randomization were not clearly described. Outcome assessors were likely to be aware of the intervention received by study participants. However, the use of a standardised blood pressure measuring protocol is likely to have minimised the risk of bias.
Friedman1977b	Biofeedback	Non-specific comparator	Office BP (attended)							Allocation concealment and randomization were not clearly described. Outcome assessors were likely to be aware of the intervention received by study participants. However, the use of a standardised blood pressure measuring protocol is likely to have minimised the risk of bias.
GarciaVera1997	Multicomponent relaxation	No intervention/Standard care	Home BP							Allocation concealment and randomization were not clearly described. Some data were missing, and this was considered likely to be due to the true value of participants blood pressure, as those with changes in anti-hypertensives were excluded. Outcome assessors were likely to be aware of the intervention received by study participants. However, the use of a standardised blood pressure measuring protocol is likely to have minimised the risk of bias. No pre-specified analysis plan was available. There was insufficient information to assess whether the result may have been selected preferentially.
Gay2007	Hypnosis	No intervention/Standard care	Office BP (attended)							Allocation concealment and randomization were not clearly described. Outcome assessors were likely to be aware of the intervention received by study participants, and no standardised protocol was used to measure blood pressure.
Hafner1982	Meditation	No intervention/Standard care	Office BP							Allocation concealment and randomization were not clearly described. A per protocol analysis was conducted, and the impact of this on the overall result may be substantial as some participants were included in the analysis twice (in the group they were originally assigned to, and the group that they transferred to). Outcome assessors were likely to be aware of the intervention received by study participants, and no standardised protocol was used to measure blood pressure. No pre-specified analysis plan was available. There was insufficient information to assess whether the result may have been selected preferentially.

Hafner1982	Biofeedback	No intervention/Standard care	Office BP							Allocation concealment and randomization were not clearly described. A per protocol analysis was conducted, and the impact of this on the overall result may be substantial as some participants were included in the analysis twice (in the group they were originally assigned to, and the group that they transferred to). Outcome assessors were likely to be aware of the intervention received by study participants, and no standardised protocol was used to measure blood pressure. No pre-specified analysis plan was available. There was insufficient information to assess whether the result may have been selected preferentially.
Hafner1982	Biofeedback	Meditation	Office BP							Allocation concealment and randomization were not clearly described. A per protocol analysis was conducted, and the impact of this on the overall result may be substantial as some participants were included in the analysis twice (in the group they were originally assigned to, and the group that they transferred to). Outcome assessors were likely to be aware of the intervention received by study participants, and no standardised protocol was used to measure blood pressure. No pre-specified analysis plan was available. There was insufficient information to assess whether the result may have been selected preferentially.
Hatch1985	Non-specific comparator	No intervention/Standard care	Home BP							Allocation concealment was not clearly described. Baseline characteristics of the two groups indicate a problem with the randomization process. Some data were missing, and there was no information available to assess whether this may be related to the blood pressure. Outcome assessors were likely to be aware of the intervention received by study participants. However, the use of a standardised blood pressure measuring protocol is likely to have minimised the risk of bias.
Hatch1985	Progressive muscle relaxation	No intervention/Standard care	Home BP							Allocation concealment was not clearly described. Baseline characteristics of the two groups indicate a problem with the randomization process. Some data were missing, and there was no information available to assess whether this may be related to the blood pressure. Outcome assessors were likely to be aware of the intervention received by study participants. However, the use of a standardised blood pressure measuring protocol is likely to have minimised the risk of bias.
Hatch1985	Biofeedback	No intervention/Standard care	Home BP							Allocation concealment was not clearly described. Baseline characteristics of the two groups indicate a problem with the randomization process. Some data were missing, and there was no information available to assess whether this may be related to the blood pressure. Outcome assessors were likely to be aware of the intervention received by study participants. However, the use of a standardised blood pressure measuring protocol is likely to have minimised the risk of bias.
Hatch1985	Progressive muscle relaxation	Non-specific comparator	Home BP							Allocation concealment was not clearly described. Baseline characteristics of the two groups indicate a problem with the randomization process. Some data were missing, and there was no information available to assess whether this may be related to the blood pressure. Outcome assessors were likely to be aware of the intervention received by study participants. However, the use of a standardised blood pressure measuring protocol is likely to have minimised the risk of bias.
Hatch1985	Biofeedback	Non-specific comparator	Home BP							Allocation concealment was not clearly described. Baseline characteristics of the two groups indicate a problem with the randomization process. Some data were missing, and there was no information available to assess whether this may be related to the blood pressure. Outcome assessors were likely to be aware of the intervention received by study participants. However, the use of a standardised blood pressure measuring protocol is likely to have minimised the risk of bias.

Hatch1985	Biofeedback	Progressive muscle relaxation	Home BP							Allocation concealment was not clearly described. Baseline characteristics of the two groups indicate a problem with the randomization process. Some data were missing, and there was no information available to assess whether this may be related to the blood pressure. Outcome assessors were likely to be aware of the intervention received by study participants. However, the use of a standardised blood pressure measuring protocol is likely to have minimised the risk of bias.
Henderson1998	Biofeedback	Non-specific comparator	Office BP (attended)							Allocation concealment and randomization were not clearly described. Outcome assessors were likely to be aware of the intervention received by study participants. However, the use of a standardised blood pressure measuring protocol is likely to have minimised the risk of bias.
Im-Oun2018	Music	No intervention/Standard care	Office BP (attended)							Baseline characteristics of the two groups indicate a problem with the randomization process. Outcome assessors were likely to be aware of the intervention received by study participants. However, the use of a standardised blood pressure measuring protocol is likely to have minimised the risk of bias. No pre-specified analysis plan was available (the study was registered retrospectively). The result may have been selected on the basis of the results because this follow-up time-point differed from other analyses.
Irvine1986	Biofeedback	Non-specific comparator	Home BP							Allocation concealment was not clearly described. Outcome assessors (participants themselves) were likely to be aware of the intervention received, and no standardised protocol was used to measure blood pressure. No pre-specified analysis plan was available. There was insufficient information to assess whether the result may have been selected preferentially.
Irvine1991	Biofeedback	Non-specific comparator	Office BP (attended)							Allocation concealment was not clearly described. Some data were missing, and this was considered likely to be due to the true value of participants blood pressure - some participants were excluded as they received antihypertensives.
Jacob1985	Relaxation + diet	No intervention/Standard care	Office BP (attended)							Allocation concealment was not clearly described. Participants and carers were aware of the assigned intervention during the trial, and this does appear to have led to deviations from the intended intervention. Eight of the 18 control participants reported engaging in relaxation techniques during the trial. Some data were missing, but this was not considered likely to be due to the true value of participants blood pressure.
Johnston1993	Multicomponent relaxation	Non-specific comparator	Ambulatory daytime BP							Allocation concealment was not clearly described. Some data were missing, and this was considered likely to be due to the true value of participants blood pressure. Missing data included those who started anti-hypertensives, those who "considered the treatment unhelpful" and people with increased BMI (due to problems in collecting ambulatory BP data).
Kalmatayeva2014	Relaxation + medication	Medication	not reported							Allocation concealment and randomization were not clearly described. The method used to measure blood pressure was not described. Outcome assessors were likely to be aware of the intervention received by study participants, and no standardised protocol was used to measure blood pressure. No pre-specified analysis plan was available. The result may have been selected on the basis of the results because of incomplete/missing results reporting for timepoints of interest.
Latha1991	Biofeedback	Non-specific comparator	Office BP (attended)							The allocation sequence could have been predicted as participants appear to have been allocated alternately to the groups. Some data were missing, and there was no information available to assess whether this may be related to the blood pressure.
Loucks2023	Relaxation + Home BP monitoring	Home BP monitoring	Office BP (unattended)							Participants and carers were aware of the assigned intervention during the trial, and this does appear to have led to deviations from the intended intervention, as some of the control participants reported engaging in mindfulness. However, these deviations were considered unlikely to affect the outcome.

Ma2018	Meditative movement	No intervention/Standard care	Office BP (attended)	Some data were missing, but this was not considered likely to be due to the true value of participants blood pressure.
McCraty2003	Psychotherapy	No intervention/Standard care	Office BP (attended)	Allocation concealment and randomization were not clearly described. Participants and carers were aware of the assigned intervention during the trial, and this does appear to have led to deviations from the intended intervention, as 2 participants crossed over to another group and 2 were excluded due to starting another non-protocol intervention. A per protocol analysis was conducted, and the impact of this on the overall result may be substantial, as a considerable proportion of randomized participants (11%) were not analysed in the assigned group. Some data were missing, and this was considered likely to be due to the true value of participants blood pressure, as participants who reduced their anti-hypertensive medication were not analysed.
Modesti2010	Breathing intervention	Non-specific comparator	Office BP (attended)	Allocation concealment and randomization were not clearly described. Outcome assessors were likely to be aware of the intervention received by study participants, and no standardised protocol was used to measure blood pressure.
Modesti2010	Breathing intervention	Music	Office BP (attended)	Allocation concealment and randomization were not clearly described. Outcome assessors were likely to be aware of the intervention received by study participants, and no standardised protocol was used to measure blood pressure.
Modesti2010	Music	Non-specific comparator	Office BP (attended)	Allocation concealment and randomization were not clearly described. Outcome assessors were likely to be aware of the intervention received by study participants, and no standardised protocol was used to measure blood pressure.
Mourya2009	Breathing intervention	No intervention/Standard care	Office BP (attended)	Allocation concealment and randomization were not clearly described. A per protocol analysis was conducted, and the impact of this on the overall result may be substantial due to the number of affected participants.
Nejati2015	Mindfulness	No intervention/Standard care	not reported	Allocation concealment and randomization were not clearly described. A per protocol analysis was conducted, and the impact of this on the overall result may be substantial, as no details are provided on the number of participants who were excluded. Outcome assessors were likely to be aware of the intervention received by study participants, and no standardised protocol was used to measure blood pressure.
Pandic2008	Breathing intervention	Music	Office BP (attended)	Outcome assessors were likely to be aware of the intervention received by study participants, and no standardised protocol was used to measure blood pressure.
Park2017	Meditative movement	No intervention/Standard care	Office BP (attended)	Some data were missing, but this was not considered likely to be due to the true value of participants blood pressure. Outcome assessors were likely to be aware of the intervention received by study participants, and no standardised protocol was used to measure blood pressure.
Patel1988	Biofeedback	Medication	Office BP (attended)	Allocation concealment was not clearly described. Baseline characteristics of the two groups indicate a problem with the randomization process. Some data were missing, and there was no information available to assess whether this may be related to the blood pressure. Outcome assessors were likely to be aware of the intervention received by study participants, and no standardised protocol was used to measure blood pressure.
Patel1988	Biofeedback	No intervention/Standard care	Office BP (attended)	Allocation concealment was not clearly described. Baseline characteristics of the two groups indicate a problem with the randomization process. Some data were missing, and there was no information available to assess whether this may be related to the blood pressure. Outcome assessors were likely to be aware of the intervention received by study participants, and no standardised protocol was used to measure blood pressure.

Patel1988	Biofeedback	Non-specific comparator	Office BP (attended)							Allocation concealment was not clearly described. Baseline characteristics of the two groups indicate a problem with the randomization process. Some data were missing, and there was no information available to assess whether this may be related to the blood pressure. Outcome assessors were likely to be aware of the intervention received by study participants, and no standardised protocol was used to measure blood pressure.
Patel1988	Biofeedback	Relaxation + medication	Office BP (attended)							Allocation concealment was not clearly described. Baseline characteristics of the two groups indicate a problem with the randomization process. Some data were missing, and there was no information available to assess whether this may be related to the blood pressure. Outcome assessors were likely to be aware of the intervention received by study participants, and no standardised protocol was used to measure blood pressure.
Patel1988	Relaxation + medication	Medication	Office BP (attended)							Allocation concealment was not clearly described. Baseline characteristics of the two groups indicate a problem with the randomization process. Some data were missing, and there was no information available to assess whether this may be related to the blood pressure. Outcome assessors were likely to be aware of the intervention received by study participants, and no standardised protocol was used to measure blood pressure.
Patel1988	Relaxation + medication	No intervention/Standard care	Office BP (attended)							Allocation concealment was not clearly described. Baseline characteristics of the two groups indicate a problem with the randomization process. Some data were missing, and there was no information available to assess whether this may be related to the blood pressure. Outcome assessors were likely to be aware of the intervention received by study participants, and no standardised protocol was used to measure blood pressure.
Patel1988	Relaxation + medication	Non-specific comparator	Office BP (attended)							Allocation concealment was not clearly described. Baseline characteristics of the two groups indicate a problem with the randomization process. Some data were missing, and there was no information available to assess whether this may be related to the blood pressure. Outcome assessors were likely to be aware of the intervention received by study participants, and no standardised protocol was used to measure blood pressure.
Patel1988	No intervention/Standard care	Medication	Office BP (attended)							Allocation concealment was not clearly described. Baseline characteristics of the two groups indicate a problem with the randomization process. Some data were missing, and there was no information available to assess whether this may be related to the blood pressure. Outcome assessors were likely to be aware of the intervention received by study participants, and no standardised protocol was used to measure blood pressure.
Patel1988	No intervention/Standard care	Non-specific comparator	Office BP (attended)							Allocation concealment was not clearly described. Baseline characteristics of the two groups indicate a problem with the randomization process. Some data were missing, and there was no information available to assess whether this may be related to the blood pressure. Outcome assessors were likely to be aware of the intervention received by study participants, and no standardised protocol was used to measure blood pressure.
Patel1988	Non-specific comparator	Medication	Office BP (attended)							Allocation concealment was not clearly described. Baseline characteristics of the two groups indicate a problem with the randomization process. Some data were missing, and there was no information available to assess whether this may be related to the blood pressure. Outcome assessors were likely to be aware of the intervention received by study participants, and no standardised protocol was used to measure blood pressure.
Plaugher2002	Massage	Non-specific comparator	Office BP (attended)							Participants and carers were aware of the assigned intervention during the trial, and this does appear to have led to deviations from the intended intervention. Some participants left the study due to dissatisfaction with their group allocation. Some data were missing, and this was considered likely to be due to the true value of participants blood pressure as some exclusions were due to the need for anti-hypertensive medication.

PonteMarquez2019	Mindfulness	Lifestyle intervention	Office BP (attended)							Allocation concealment and randomization were not clearly described. Some data were missing, but this was not considered likely to be due to the true value of participants blood pressure. Outcome assessors were likely to be aware of the intervention received by study participants. However, the use of a standardised blood pressure measuring protocol is likely to have minimised the risk of bias. No pre-specified analysis plan was available. There was insufficient information to assess whether the result may have been selected preferentially.
Saensak2013	Progressive muscle relaxation	Lifestyle intervention	Office BP (attended)							Some data were missing, and there was no information available to assess whether this may be related to the blood pressure. Outcome assessors were likely to be aware of the intervention received by study participants, and no standardised protocol was used to measure blood pressure.
Sangthong2016	Relaxation + Home BP monitoring	Home BP monitoring	Home BP							Allocation concealment and randomization were not clearly described. A per protocol analysis was conducted, but the impact of this on the overall result was considered to be small. Outcome assessors were likely to be aware of the intervention received by study participants, and no standardised protocol was used to measure blood pressure.
Schein2001	Breathing intervention	Music	Office BP (attended)							Allocation concealment was not clearly described. Multiple participants dropped out of the study and were excluded from the analysis but no reasons were provided. It is not clear whether this was due to the use of a per-protocol analysis. Some data were missing, and there was no information available to assess whether this may be related to the blood pressure. No pre-specified analysis plan was available, but the authors state that the current follow-up was not planned. There was insufficient information to assess whether the result may have been selected preferentially.
Schneider2005	Meditation	Lifestyle intervention	Office BP (attended)							Allocation concealment was not clearly described. Some data were missing, and there was no information available to assess whether this may be related to the blood pressure.
Schneider2005	Meditation	Progressive muscle relaxation	Office BP (attended)							Allocation concealment was not clearly described. Some data were missing, and there was no information available to assess whether this may be related to the blood pressure.
Schneider2005	Progressive muscle relaxation	Lifestyle intervention	Office BP (attended)							Allocation concealment was not clearly described. Some data were missing, and there was no information available to assess whether this may be related to the blood pressure.
Schneider2019	Meditation	Lifestyle intervention	Office BP (attended)							Some data were missing, and there was no information available to assess whether this may be related to the blood pressure.
Seer1980	Meditation	No intervention/Standard care	Office BP (attended)							Allocation concealment and randomization were not clearly described. Some data were missing, and this was considered likely to be due to the true value of participants blood pressure because participants were excluded due to starting antihypertensive medication. No pre-specified analysis plan was available. There was insufficient information to assess whether the result may have been selected preferentially.
Southam1982	Progressive muscle relaxation	No intervention/Standard care	Office BP (attended)							Allocation concealment was not clearly described. Participants and carers were aware of the assigned intervention during the trial, and this does appear to have led to deviations from the intended intervention. Some participants in the control group took up relaxation techniques, reduced salt in their diet and stopped smoking during the course of the trial. A per protocol analysis was conducted, but the impact of this on the overall result was considered to be small. Some data were missing, and this was considered likely to be due to the true value of participants blood pressure (one participant missing due to a heart attack).
Sun2015	Meditative movement	Non-specific comparator	not reported							Allocation concealment and randomization were not clearly described. A per protocol analysis was conducted, but the impact of this on the overall result was considered to be small. Some

										data were missing, and there was no information available to assess whether this may be related to the blood pressure. The analysis plan outlined on the trial registry site/in the protocol appears to differ from this analysis because data from a second site (in Australia) was planned to be included. It is unclear why these data were not presented.
Supriya2018	Meditative movement	No intervention/Standard care	Office BP (attended)							Allocation concealment was not clearly described. Outcome assessors were likely to be aware of the intervention received by study participants. However, the use of a standardised blood pressure measuring protocol is likely to have minimised the risk of bias. No pre-specified analysis plan was available. There was insufficient information to assess whether the result may have been selected preferentially. This was a subgroup analysis of a larger primary trial, likely not prospectively planned, but selection of participants with high BP was justified in the introduction.
Taylor1977	Progressive muscle relaxation	No intervention/Standard care	Office BP (attended)							Allocation concealment and randomization were not clearly described. Some data were missing, but this was not considered likely to be due to the true value of participants blood pressure. No pre-specified analysis plan was available. There was insufficient information to assess whether the result may have been selected preferentially.
Taylor1977	Progressive muscle relaxation	Non-specific comparator	Office BP (attended)							Allocation concealment and randomization were not clearly described. Some data were missing, but this was not considered likely to be due to the true value of participants blood pressure. No pre-specified analysis plan was available. There was insufficient information to assess whether the result may have been selected preferentially.
Taylor1977	Non-specific comparator	No intervention/Standard care	Office BP (attended)							Allocation concealment and randomization were not clearly described. Some data were missing, but this was not considered likely to be due to the true value of participants blood pressure. No pre-specified analysis plan was available. There was insufficient information to assess whether the result may have been selected preferentially.
Tosi1992	Multicomponent intervention	Non-specific comparator	Office BP (attended)							Allocation concealment and randomization were not clearly described. Some data were missing, and this was considered likely to be due to the true value of participants blood pressure as 5% of participants were excluded due to extreme BP elevations.
Tosi1992	Multicomponent intervention	Psychotherapy	Office BP (attended)							Allocation concealment and randomization were not clearly described. Some data were missing, and this was considered likely to be due to the true value of participants blood pressure as 5% of participants were excluded due to extreme BP elevations.
Tosi1992	Multicomponent intervention	Hypnosis	Office BP (attended)							Allocation concealment and randomization were not clearly described. Some data were missing, and this was considered likely to be due to the true value of participants blood pressure as 5% of participants were excluded due to extreme BP elevations.
Tosi1992	Hypnosis	Non-specific comparator	Office BP (attended)							Allocation concealment and randomization were not clearly described. Some data were missing, and this was considered likely to be due to the true value of participants blood pressure as 5% of participants were excluded due to extreme BP elevations.
Tosi1992	Hypnosis	Psychotherapy	Office BP (attended)							Allocation concealment and randomization were not clearly described. Some data were missing, and this was considered likely to be due to the true value of participants blood pressure as 5% of participants were excluded due to extreme BP elevations.
Tosi1992	Psychotherapy	Non-specific comparator	Office BP (attended)							Allocation concealment and randomization were not clearly described. Some data were missing, and this was considered likely to be due to the true value of participants blood pressure as 5% of participants were excluded due to extreme BP elevations.
vanMontfrans1990	Multicomponent intervention	Non-specific comparator	other*							Allocation concealment was not clearly described. Some data were missing, and this was considered likely to be due to the true value of participants blood pressure, as 2 control participants were excluded due to chest pain or an increase in diastolic blood pressure. *BP assessed using invasive daytime ambulatory readings (intra-arterial readings).

Wadden1984	Psychotherapy	Relaxation + lifestyle intervention	Office BP (attended)							Allocation concealment was not clearly described. A per protocol analysis was conducted, and the impact of this on the overall result may be substantial because 10% of participants were excluded due to non-adherence. Some data were missing, and this was considered likely to be due to the true value of participants blood pressure because 3 participants were excluded due to having BP lower than a certain threshold (different from eligibility criteria). Outcome assessors were likely to be aware of the intervention received by study participants. However, the use of a standardised blood pressure measuring protocol is likely to have minimised the risk of bias. No pre-specified analysis plan was available. The result may have been selected on the basis of the results because only 2/3 weeks of the baseline period were selected to estimate baseline BP.
Wang2016	Biofeedback	Non-specific comparator	Office BP							Some data were missing, and this was considered likely to be due to the true value of participants blood pressure because one participant was excluded due to starting anti-hypertensive medication. However, the overall impact on the result is likely to be small.
Xiao2016	Meditative movement	No intervention/Standard care	not reported							Allocation concealment and randomization were not clearly described. The method used to measure blood pressure was not described.
Ziv2013	Relaxation + multicomponent supplementary intervention	Multicomponent supplementary intervention	Ambulatory 24 hour BP							Allocation concealment and randomization were not clearly described. Some data were missing, but this was not considered likely to be due to the true value of participants blood pressure.
Zurawski1987	Multicomponent intervention	Biofeedback	Office BP							Allocation concealment was not clearly described. Some data were missing, but this was not considered likely to be due to the true value of participants blood pressure. Outcome assessors were likely to be aware of the intervention received by study participants. However, the use of a standardised blood pressure measuring protocol is likely to have minimised the risk of bias. No pre-specified analysis plan was available. There was insufficient information to assess whether the result may have been selected preferentially.

Table S23: Hypertension, medium follow-up, cluster randomised trials

**Study**	**Intervention**	**Comparator**	**Outcome measure**	**D1a**	**D1b**	**D2**	**D3**	**D4**	**D5**	**Overall**	**Description of concerns**
Hasandokht2015	Relaxation + lifestyle intervention	No intervention/standard care	Office BP (attended)								Allocation concealment and randomization were not clearly described. Some data were missing, but this was not considered likely to be due to the true value of participants blood pressure.

Table S24: Hypertension population, long follow-up, individually randomised trials

Study	Intervention	Comparator	Outcome measure	D1	D2	D3	D4	D5	Overall	Description of concerns
Agras1987	Progressive muscle relaxation	No intervention/Standard care	Office BP (attended)							Allocation concealment was not clearly described. Some data were missing, but this was not considered likely to be due to the true value of participants blood pressure.
Aivazyan1988b	Autogenic training	No intervention/ Standard care	Office BP (attended)							Allocation concealment and randomization were not clearly described. Outcome assessors were likely to be aware of the intervention received by study participants. However, the use of a standardised blood pressure measuring protocol is likely to have minimised the risk of bias. No pre-specified analysis plan was available. The result may have been selected on the basis of the results because 5-year follow-up was reported after a 6-month intervention. It was unclear when and why this particular timepoint was specified.
Chesney1987	Progressive muscle relaxation	No intervention/ Standard care	Office BP (attended)							Allocation concealment was not clearly described. Some data were missing, but this was not considered likely to be due to the true value of participants blood pressure. Outcome assessors were likely to be aware of the intervention received by study participants. However, the use of a standardised blood pressure measuring protocol is likely to have minimised the risk of bias. No pre-specified analysis plan was available. There was insufficient information to assess whether the result may have been selected preferentially.
Chesney1987	Multicomponent relaxation	No intervention/ Standard care	Office BP (attended)							Allocation concealment was not clearly described. Some data were missing, but this was not considered likely to be due to the true value of participants blood pressure. Outcome assessors were likely to be aware of the intervention received by study participants. However, the use of a standardised blood pressure measuring protocol is likely to have minimised the risk of bias. No pre-specified analysis plan was available. There was insufficient information to assess whether the result may have been selected preferentially.
Chesney1987	Biofeedback	No intervention/ Standard care	Office BP (attended)							Allocation concealment was not clearly described. Some data were missing, but this was not considered likely to be due to the true value of participants blood pressure. Outcome assessors were likely to be aware of the intervention received by study participants. However, the use of a standardised blood pressure measuring protocol is likely to have minimised the risk of bias. No pre-specified analysis plan was available. There was insufficient information to assess whether the result may have been selected preferentially.
Chesney1987	Relaxation + lifestyle intervention	No intervention/Standard care	Office BP (attended)							Allocation concealment was not clearly described. Some data were missing, but this was not considered likely to be due to the true value of participants blood pressure. Outcome assessors were likely to be aware of the intervention received by study participants. However, the use of a standardised blood pressure measuring protocol is likely to have minimised the risk of bias. No pre-specified analysis plan was available. There was insufficient information to assess whether the result may have been selected preferentially.
Chesney1987	Multicomponent relaxation	Progressive muscle relaxation	Office BP (attended)							Allocation concealment was not clearly described. Some data were missing, but this was not considered likely to be due to the true value of participants blood pressure. Outcome assessors were likely to be aware of the intervention received by study participants. However, the use of a standardised blood pressure measuring protocol is likely to have minimised the risk of bias.

										No pre-specified analysis plan was available. There was insufficient information to assess whether the result may have been selected preferentially.
Chesney1987	Biofeedback	Progressive muscle relaxation	Office BP (attended)							Allocation concealment was not clearly described. Some data were missing, but this was not considered likely to be due to the true value of participants blood pressure. Outcome assessors were likely to be aware of the intervention received by study participants. However, the use of a standardised blood pressure measuring protocol is likely to have minimised the risk of bias. No pre-specified analysis plan was available. There was insufficient information to assess whether the result may have been selected preferentially.
Chesney1987	Relaxation + lifestyle intervention	Progressive muscle relaxation	Office BP (attended)							Allocation concealment was not clearly described. Some data were missing, but this was not considered likely to be due to the true value of participants blood pressure. Outcome assessors were likely to be aware of the intervention received by study participants. However, the use of a standardised blood pressure measuring protocol is likely to have minimised the risk of bias. No pre-specified analysis plan was available. There was insufficient information to assess whether the result may have been selected preferentially.
Chesney1987	Biofeedback	Multicomponent relaxation	Office BP (attended)							Allocation concealment was not clearly described. Some data were missing, but this was not considered likely to be due to the true value of participants blood pressure. Outcome assessors were likely to be aware of the intervention received by study participants. However, the use of a standardised blood pressure measuring protocol is likely to have minimised the risk of bias. No pre-specified analysis plan was available. There was insufficient information to assess whether the result may have been selected preferentially.
Chesney1987	Relaxation + lifestyle intervention	Multicomponent relaxation	Office BP (attended)							Allocation concealment was not clearly described. Some data were missing, but this was not considered likely to be due to the true value of participants blood pressure. Outcome assessors were likely to be aware of the intervention received by study participants. However, the use of a standardised blood pressure measuring protocol is likely to have minimised the risk of bias. No pre-specified analysis plan was available. There was insufficient information to assess whether the result may have been selected preferentially.
Chesney1987	Relaxation + lifestyle intervention	Biofeedback	Office BP (attended)							Allocation concealment was not clearly described. Some data were missing, but this was not considered likely to be due to the true value of participants blood pressure. Outcome assessors were likely to be aware of the intervention received by study participants. However, the use of a standardised blood pressure measuring protocol is likely to have minimised the risk of bias. No pre-specified analysis plan was available. There was insufficient information to assess whether the result may have been selected preferentially.
Gay2007	Hypnosis	No intervention/ Standard care	Office BP (attended)							Allocation concealment and randomization were not clearly described. Outcome assessors were likely to be aware of the intervention received by study participants, and no standardised protocol was used to measure blood pressure.
Hatch1985	Non-specific comparator	No intervention/ Standard care	Home BP							Allocation concealment was not clearly described. Baseline characteristics of the two groups indicate a problem with the randomization process. Some data were missing, and there was no information available to assess whether this may be related to the blood pressure. Outcome assessors were likely to be aware of the intervention received by study participants. However, the use of a standardised blood pressure measuring protocol is likely to have minimised the risk of bias.
Hatch1985	Progressive muscle relaxation	No intervention/Standard care	Home BP							Allocation concealment was not clearly described. Baseline characteristics of the two groups indicate a problem with the randomization process. Some data were missing, and there was no information available to assess whether this may be related to the blood pressure. Outcome assessors were likely to be aware of the intervention received by study participants. However,

										the use of a standardised blood pressure measuring protocol is likely to have minimised the risk of bias.
Hatch1985	Biofeedback	No intervention/Standard care	Home BP							Allocation concealment was not clearly described. Baseline characteristics of the two groups indicate a problem with the randomization process. Some data were missing, and there was no information available to assess whether this may be related to the blood pressure. Outcome assessors were likely to be aware of the intervention received by study participants. However, the use of a standardised blood pressure measuring protocol is likely to have minimised the risk of bias.
Hatch1985	Progressive muscle relaxation	Non-specific comparator	Home BP							Allocation concealment was not clearly described. Baseline characteristics of the two groups indicate a problem with the randomization process. Some data were missing, and there was no information available to assess whether this may be related to the blood pressure. Outcome assessors were likely to be aware of the intervention received by study participants. However, the use of a standardised blood pressure measuring protocol is likely to have minimised the risk of bias.
Hatch1985	Biofeedback	Non-specific comparator	Home BP							Allocation concealment was not clearly described. Baseline characteristics of the two groups indicate a problem with the randomization process. Some data were missing, and there was no information available to assess whether this may be related to the blood pressure. Outcome assessors were likely to be aware of the intervention received by study participants. However, the use of a standardised blood pressure measuring protocol is likely to have minimised the risk of bias.
Hatch1985	Biofeedback	Progressive muscle relaxation	Home BP							Allocation concealment was not clearly described. Baseline characteristics of the two groups indicate a problem with the randomization process. Some data were missing, and there was no information available to assess whether this may be related to the blood pressure. Outcome assessors were likely to be aware of the intervention received by study participants. However, the use of a standardised blood pressure measuring protocol is likely to have minimised the risk of bias.
Shapiro1997	Psychotherapy	Non-specific comparator	Ambulatory daytime BP							Allocation concealment and randomization were not clearly described. No pre-specified analysis plan was available. There was insufficient information to assess whether the result may have been selected preferentially.
Southam1982	Progressive muscle relaxation	No intervention/Standard care	Ambulatory daytime BP							Allocation concealment was not clearly described. Participants and carers were aware of the assigned intervention during the trial, and this does appear to have led to deviations from the intended intervention. Some participants in the control group took up relaxation techniques, reduced salt in their diet and stopped smoking during the course of the trial. A per protocol analysis was conducted, but the impact of this on the overall result was considered to be small. Some data were missing, and this was considered likely to be due to the true value of participants blood pressure (one participant missing due to a heart attack). No pre-specified analysis plan was available. The result may have been selected on the basis of the results because this outcome assessment time point (15 months) was not mentioned in an earlier paper reporting the same study.

Table S25: Prehypertension, short follow-up, individually randomised trials

Study	Intervention	Comparator	Outcome measure	D1	D2	D3	D4	D5	Overall	Description of concerns
Adams2018	Breathing intervention (10 minutes)	Breathing intervention (5 minutes)	Office BP (attended)							Allocation concealment was not clearly described. A per protocol analysis was conducted, but the impact of this on the overall result was considered to be small. Some data were missing, but this was not considered likely to be due to the true value of participants blood pressure.
Adams2018	Breathing intervention (15 minutes)	Breathing intervention (5 minutes)	Office BP (attended)							Allocation concealment was not clearly described. A per protocol analysis was conducted, but the impact of this on the overall result was considered to be small. Some data were missing, but this was not considered likely to be due to the true value of participants blood pressure.
Adams2018	Breathing intervention (15 minutes)	Breathing intervention (10 minutes)	Office BP (attended)							Allocation concealment was not clearly described. A per protocol analysis was conducted, but the impact of this on the overall result was considered to be small. Some data were missing, but this was not considered likely to be due to the true value of participants blood pressure.
Ankolekar2019	Meditative movement	No intervention/ Standard care	Office BP (attended)							Allocation concealment was not clearly described. Outcome assessors were likely to be aware of the intervention received by study participants. However, the use of a standardised blood pressure measuring protocol is likely to have minimised the risk of bias. No pre-specified analysis plan was available. There was insufficient information to assess whether the result may have been selected preferentially.
Batey2000	Multicomponent relaxation	No intervention/ Standard care	Office BP (attended)							Some data were missing, and this was considered likely to be due to the true value of participants blood pressure. Data were imputed for participants who suffered a hypertensive crisis, or who started anti-hypertensives. However, the amount of data missing at this time point is relatively small.
Chandler2020	Breathing intervention	Lifestyle intervention	Office BP (attended)							Allocation concealment and randomization were not clearly described. The analysis plan outlined on the trial registry site appears to differ from this analysis because a specific subgroup of participants were selected for presentation in this article. Full data from the trial do not appear to have been published. The trial registration also lists ambulatory BP as a secondary outcome measure, which is not reported.
Chen2016b	Breathing intervention	Non-specific comparator	Office BP							Allocation concealment and randomization were not clearly described. Outcome assessors were likely to be aware of the intervention received by study participants, and no standardised protocol was used to measure blood pressure. No pre-specified analysis plan was available. There was insufficient information to assess whether the result may have been selected preferentially.
Chen2016b	Biofeedback	Non-specific comparator	Office BP							Allocation concealment and randomization were not clearly described. Outcome assessors were likely to be aware of the intervention received by study participants, and no standardised protocol was used to measure blood pressure. No pre-specified analysis plan was available. There was insufficient information to assess whether the result may have been selected preferentially.
Chen2016b	Biofeedback	Breathing intervention	Office BP							Allocation concealment and randomization were not clearly described. Outcome assessors were likely to be aware of the intervention received by study participants, and no standardised protocol was used to measure blood pressure. No pre-specified analysis plan was available. There was insufficient information to assess whether the result may have been selected preferentially.
Givi2018	Massage	Non-specific comparator	Office BP (attended)							Allocation concealment and randomization were not clearly described. No pre-specified analysis plan was available. There was insufficient information to assess whether the result may have been selected preferentially.

Hughes2013	Mindfulness	Progressive muscle relaxation	Office BP							Some data were missing, but this was not considered likely to be due to the true value of participants blood pressure.
Lin2012	Breathing intervention	Non-specific comparator	Office BP (attended)							Allocation concealment was not clearly described. Outcome assessors were likely to be aware of the intervention received by study participants. However, the use of a standardised blood pressure measuring protocol is likely to have minimised the risk of bias. No pre-specified analysis plan was available. There was insufficient information to assess whether the result may have been selected preferentially.
Lin2012	Biofeedback	Non-specific comparator	Office BP (attended)							Allocation concealment was not clearly described. Outcome assessors were likely to be aware of the intervention received by study participants. However, the use of a standardised blood pressure measuring protocol is likely to have minimised the risk of bias. No pre-specified analysis plan was available. There was insufficient information to assess whether the result may have been selected preferentially.
Lin2012	Biofeedback	Breathing intervention	Office BP (attended)							Allocation concealment was not clearly described. Outcome assessors were likely to be aware of the intervention received by study participants. However, the use of a standardised blood pressure measuring protocol is likely to have minimised the risk of bias. No pre-specified analysis plan was available. There was insufficient information to assess whether the result may have been selected preferentially.
Mir2021	Relaxation + diet	Diet	Office BP (attended)							Allocation concealment and randomization were not clearly described. Outcome assessors were likely to be aware of the intervention received by study participants. However, the use of a standardised blood pressure measuring protocol is likely to have minimised the risk of bias.
Pukdeesamai2023	Mindfulness	No intervention/ Standard care	Office BP (attended)							Allocation concealment and randomization were not clearly described. Some data were missing, and there was no information available to assess whether this may be related to the blood pressure. Outcome assessors were likely to be aware of the intervention received by study participants. However, the use of a standardised blood pressure measuring protocol is likely to have minimised the risk of bias.
Singh2022	Relaxation + lifestyle intervention	Lifestyle intervention	not reported							Baseline characteristics of the two groups indicate a problem with the randomization process. The method used to measure blood pressure was not described. No pre-specified analysis plan was available. There was insufficient information to assess whether the result may have been selected preferentially.
Thiyagarajan2015	Relaxation + lifestyle intervention	Lifestyle intervention	Office BP							Allocation concealment was not clearly described. A per protocol analysis was conducted, and the impact of this on the overall result may be substantial due to the number of participants affected. Some data were missing, but this was not considered likely to be due to the true value of participants blood pressure. Outcome assessors were likely to be aware of the intervention received by study participants. However, the use of a standardised blood pressure measuring protocol is likely to have minimised the risk of bias.
Wang2010	Biofeedback	Breathing intervention	Office BP (attended)							Allocation concealment and randomization were not clearly described. Outcome assessors were likely to be aware of the intervention received by study participants. However, the use of a standardised blood pressure measuring protocol is likely to have minimised the risk of bias.
Xu2007	Biofeedback	Non-specific comparator	Office BP (attended)							Allocation concealment was not clearly described. Multiple participants dropped out of the study and were excluded from the analysis but no reasons were provided. It is not clear whether this was due to the use of a per-protocol analysis. Some data were missing, but this was not considered likely to be due to the true value of participants blood pressure. Outcome assessors were likely to be aware of the intervention received by study participants. However, the use of a standardised blood pressure measuring protocol is likely to have minimised the risk of bias.

Table S26: Prehypertension, medium follow-up, individually randomised trials

Study	Intervention	Comparator	Outcome measure	D1	D2	D3	D4	D5	Overall	Description of concerns
Adams2018	Breathing intervention (10 minutes)	Breathing intervention (5 minutes)	Office BP (attended)							Allocation concealment was not clearly described.<br>A per protocol analysis was conducted, but the impact of this on the overall result was considered to be small. Some data were missing, and there was no information available to assess whether this may be related to the blood pressure.
Adams2018	Breathing intervention (15 minutes)	Breathing intervention (5 minutes)	Office BP (attended)							Allocation concealment was not clearly described. A per protocol analysis was conducted, but the impact of this on the overall result was considered to be small. Some data were missing, and there was no information available to assess whether this may be related to the blood pressure.
Adams2018	Breathing intervention (15 minutes)	Breathing intervention (10 minutes)	Office BP (attended)							Allocation concealment was not clearly described. A per protocol analysis was conducted, but the impact of this on the overall result was considered to be small. Some data were missing, and there was no information available to assess whether this may be related to the blood pressure.
Ankolekar2019	Meditative movement	No intervention/ Standard care	Office BP (attended)							Allocation concealment was not clearly described. Outcome assessors were likely to be aware of the intervention received by study participants. However, the use of a standardised blood pressure measuring protocol is likely to have minimised the risk of bias. No pre-specified analysis plan was available. There was insufficient information to assess whether the result may have been selected preferentially.
Batey2000	Multicompon ent relaxation	No intervention/ Standard care	Office BP (attended)							Some data were missing, and this was considered likely to be due to the true value of participants blood pressure. Data were imputed for participants who suffered a hypertensive crisis, or who started anti-hypertensives.
Li2024	Meditative movement	Exercise	Ambulatory 24 hour BP							Some data were missing, but this was not considered likely to be due to the true value of participants blood pressure.
Pukdeesamai2023	Mindfulness	No intervention/ Standard care	Office BP (attended)							Allocation concealment and randomization were not clearly described. Some data were missing, and there was no information available to assess whether this may be related to the blood pressure. Outcome assessors were likely to be aware of the intervention received by study participants. However, the use of a standardised blood pressure measuring protocol is likely to have minimised the risk of bias.
Singh2022	Relaxation + lifestyle intervention	Lifestyle intervention	not reported							Baseline characteristics of the two groups indicate a problem with the randomization process. The method used to measure blood pressure was not described. No pre-specified analysis plan was available. There was insufficient information to assess whether the result may have been selected preferentially.
Wang2010	Biofeedback	Breathing intervention	Office BP (attended)							Allocation concealment and randomization were not clearly described. Outcome assessors were likely to be aware of the intervention received by study participants. However, the use of a standardised blood pressure measuring protocol is likely to have minimised the risk of bias.
Xu2007	Biofeedback	Non-specific comparator	Office BP (attended)							Allocation concealment was not clearly described. Multiple participants dropped out of the study and were excluded from the analysis but no reasons were provided. It is not clear whether this was due to the use of a per-protocol analysis. Some data were missing, but this was not considered likely to be due to the true value of participants blood pressure. Outcome assessors were likely to be aware of

										the intervention received by study participants. However, the use of a standardised blood pressure measuring protocol is likely to have minimised the risk of bias. No pre-specified analysis plan was available. There was insufficient information to assess whether the result may have been selected preferentially.

Table S27: Prehypertension, long follow-up, individually randomised trials

Study	Intervention	Comparator	Outcome measure	D1	D2	D3	D4	D5	Overall	Description of concerns
Batey2000	Multicomponent relaxation	No intervention/ Standard care	Office BP (attended)							Some data were missing, and this was considered likely to be due to the true value of participants blood pressure. Data were imputed for participants who suffered a hypertensive crisis, or who started anti-hypertensives.
Chandler2020	Breathing intervention	Lifestyle intervention	Office BP (attended)							Allocation concealment and randomization were not clearly described. The analysis plan outlined on the trial registry site appears to differ from this analysis because a specific subgroup of participants were selected for presentation in this article. Full data from the trial do not appear to have been published. The trial registration also lists ambulatory BP as a secondary outcome measure, which is not reported.
Schneider2021	Meditation	Lifestyle intervention	Office BP (attended)							Some data were missing, and there was no information available to assess whether this may be related to the blood pressure. No pre-specified analysis plan was available. There was insufficient information to assess whether the result may have been selected preferentially.

Table S28: Secondary outcomes (hypertension and pre-hypertension), individually randomised trials

Study	Population	Intervention	Comparator	Outcome	D1	D2	D3	D4	D5	Overall	Description of concerns
Batey 2000	Pre-hypertension	Multicomponent intervention	Passive comparator	Mortality							Some data were missing, but this was not considered likely to be due to the outcome. No information is reported to indicate how mortality data were collected - it was unclear if this was a systematic process. The trial protocol does not indicate that mortality data would be collected, therefore this may not be a pre-specified analysis.
Patel 1988	Hypertension	Relaxation	Passive comparator	Mortality							Allocation concealment was not clearly described. Baseline characteristics of the two groups indicate a problem with the randomization process. Some data were missing, but this was not considered likely to be due to the outcome.
Patel 1988	Hypertension	Relaxation	Passive comparator	CV morbidity							Allocation concealment was not clearly described. Baseline characteristics of the two groups indicate a problem with the randomization process. Some data were missing, but this was not considered likely to be due to the outcome.

Schneider 1995	Hypertension	Progressive muscle relaxation	Lifestyle intervention	Mortality							Allocation concealment was not clearly described. A per protocol analysis was conducted, and the impact of this on the overall result may be substantial, as the text indicates that a weaker effect was seen when conducting an intention-to-treat analysis (data are reported incompletely). Some data were missing, but this was not considered likely to be due to the outcome. No pre-specified analysis plan was available. There was insufficient information to assess whether the result may have been selected preferentially.
Schneider 1995	Hypertension	Progressive muscle relaxation	Meditation	Mortality							Allocation concealment was not clearly described. A per protocol analysis was conducted, and the impact of this on the overall result may be substantial, as the text indicates that a weaker effect was seen when conducting an intention-to-treat analysis (data are reported incompletely). Some data were missing, but this was not considered likely to be due to the outcome. No pre-specified analysis plan was available. There was insufficient information to assess whether the result may have been selected preferentially.
Schneider 1995	Hypertension	Meditation	Lifestyle intervention	Mortality							Allocation concealment was not clearly described. A per protocol analysis was conducted, and the impact of this on the overall result may be substantial, as the text indicates that a weaker effect was seen when conducting an intention-to-treat analysis (data are reported incompletely). Some data were missing, but this was not considered likely to be due to the outcome. No pre-specified analysis plan was available. There was insufficient information to assess whether the result may have been selected preferentially.
Schneider 1995	Hypertension	Progressive muscle relaxation	Lifestyle intervention	CV morbidity							Allocation concealment was not clearly described. A per protocol analysis was conducted, and the impact of this on the overall result may be substantial, as the text indicates that a weaker effect was seen when conducting an intention-to-treat analysis (data are reported incompletely). Some data were missing, but this was not considered likely to be due to the outcome. No pre-specified analysis plan was available. There was insufficient information to assess whether the result may have been selected preferentially.
Schneider 1995	Hypertension	Progressive muscle relaxation	Meditation	CV morbidity							Allocation concealment was not clearly described. A per protocol analysis was conducted, and the impact of this on the overall result may be substantial, as the text indicates that a weaker effect was seen when conducting an intention-to-treat analysis (data are reported incompletely). Some data were missing, but this was not considered likely to be due to the outcome. No pre-specified analysis plan was available. There was insufficient information to assess whether the result may have been selected preferentially.
Schneider 1995	Hypertension	Meditation	Lifestyle intervention	CV morbidity							Allocation concealment was not clearly described. A per protocol analysis was conducted, and the impact of this on the overall result may be substantial, as the text indicates that a weaker effect was seen when conducting an intention-to-treat analysis (data are reported incompletely). Some data were missing, but this was not considered likely to be due to the outcome. No pre-specified analysis plan was available. There was insufficient information to assess whether the result may have been selected preferentially.

GRADE Assessments

Table S29: Hypertension GRADE profile: Relaxation intervention versus comparator/alternative relaxation intervention at >12 months' follow-up

Certainty assessment							№ of patients		Effect	Certainty
**№ of studies**	**Study design**	**Risk of bias**	**Heterogeneity**	**Indirectness**	**Imprecision**	**Other considerations**	**Relaxation intervention**	**Comparator**	**Absolute (95% CI)**	
**Relaxation intervention versus passive comparator, > 12 months follow-up**										
**Systolic BP - Autogenic training versus passive comparator**										
1<br>Aivazyan 1988b (21)	randomised trial	serious[a]	not serious	not serious	not serious	publication bias strongly suspected[b]	44	46	MD **6.95 mmHg lower** (10.99 lower to 2.91 lower)	⨁⨁◯◯ Low
**Systolic BP - Biofeedback versus passive comparator**										
1<br>Chesney 1987 (51)	randomised trial	serious[a]	not serious	not serious	serious[c]	publication bias strongly suspected[b]	49	40	MD **4.47 mmHg higher** (0.55 lower to 9.49 higher)	⨁◯◯◯ Very low
**Systolic BP - Multicomponent intervention versus passive comparator**										
1<br>Chesney 1987 (51)	randomised trial	serious[a]	not serious	not serious	very serious[d]	publication bias strongly suspected[b]	24	40	MD **0.7 mmHg lower** (6.79 lower to 5.39 higher)	⨁◯◯◯ Very low

**Systolic BP - PMR versus passive comparator**										
2<br><br>Agras 1987 (18), Chesney 1987 (51)	randomised trials	serious[a]	not serious	not serious	very serious[d]	publication bias strongly suspected[b]	71	89	One study showed a MD **0.80 mmHg lower** (7.46 lower to 5.86 higher); one study showed a MD **1.70 mmHg higher** (4.39 lower to 7.79 higher)	⊕◯◯◯<br>Very low
**Diastolic BP - Autogenic training versus passive comparator**										
1<br><br>Aivazyan 1988b (21)	randomised trial	serious[a]	not serious	not serious	not serious	publication bias strongly suspected[b]	44	46	MD **5.95 mmHg lower**<br>(9.01 lower to 2.89 lower)	⊕⊕◯◯<br>Low
**Diastolic BP - Biofeedback versus passive comparator**										
1<br><br>Chesney 1987 (51)	randomised trial	serious[a]	not serious	not serious	not serious	publication bias strongly suspected[b]	49	40	MD **3.18 mmHg higher**<br>(0.11 higher to 6.24 higher)	⊕⊕◯◯<br>Low
**Diastolic BP - Multicomponent intervention versus passive comparator**										
1<br><br>Chesney 1987 (51)	randomised trial	serious[a]	not serious	not serious	serious[e]	publication bias strongly suspected[b]	24	40	MD **0.8 mmHg lower**<br>(4.51 lower to 2.91 higher)	⊕◯◯◯<br>Very low
**Diastolic BP - PMR versus passive comparator**										

2<br><br>Agras 1987 (18), Chesney 1987 (51)	randomised trials	serious[a]	not serious	not serious	very serious[d]	publication bias strongly suspected[b]	71	89	One study showed a MD **0.30 mmHg lower** (5.09 lower to 4.49 higher); one study showed a MD **0.80 mmHg lower** (4.51 lower to 2.91 higher)	⊕◯◯◯<br>Very low
**Relaxation intervention versus nonspecific comparator, > 12 months follow-up**										
**Systolic BP - Psychotherapy versus nonspecific comparator**										
1<br><br>Shapiro 1997 (176)	randomised trial	serious[a]	not serious	serious[f]	not serious	publication bias strongly suspected[b]	22	17	MD **7.11 mmHg higher**<br>(0.45 higher to 13.76 higher)	⊕◯◯◯<br>Very low
**Diastolic BP - Psychotherapy versus nonspecific comparator**										
1<br><br>Shapiro 1997 (176)	randomised trial	serious[a]	not serious	serious[f]	serious[c]	publication bias strongly suspected[b]	22	17	MD **3.43 mmHg higher**<br>(0.28 lower to 7.14 higher)	⊕◯◯◯<br>Very low
**Relaxation intervention versus alternative relaxation intervention, >12 months follow-up**										
**Systolic BP - Multicomponent intervention versus biofeedback**										
1<br><br>Chesney 1987 (51)	randomised trial	serious[a]	not serious	not serious	serious[e]	publication bias strongly suspected[b]	24	49	MD **5.17 mmHg lower**<br>(11.04 lower to 0.7 higher)	⊕◯◯◯<br>Very low
**Systolic BP - Multicomponent intervention versus PMR**										

1<br><br>Chesney 1987 (51)	randomised trial	serious[a]	not serious	not serious	serious[e]	publication bias strongly suspected[b]	24	24	MD **2.4 mmHg lower**<br>(9.2 lower to 4.4 higher)	⊕◯◯◯<br>Very low
**Systolic BP - PMR versus biofeedback**										
1<br><br>Chesney 1987 (51)	randomised trial	serious[a]	not serious	not serious	serious[g]	publication bias strongly suspected[b]	24	49	MD **2.77 mmHg lower**<br>(8.64 lower to 3.1 higher)	⊕◯◯◯<br>Very low
**Diastolic BP - Multicomponent intervention versus biofeedback**										
1<br><br>Chesney 1987 (51)	randomised trial	serious[a]	not serious	not serious	not serious	publication bias strongly suspected[b]	24	49	MD **3.98 mmHg lower**<br>(7.55 lower to 0.4 lower)	⊕⊕◯◯<br>Low
**Diastolic BP - Multicomponent intervention versus PMR**										
1<br><br>Chesney 1987 (51)	randomised trial	serious[a]	not serious	not serious	very serious[d]	publication bias strongly suspected[b]	24	24	MD **0 mmHg**<br>(4.15 lower to 4.15 higher)	⊕◯◯◯<br>Very low
**Diastolic BP - PMR versus biofeedback**										
1<br><br>Chesney 1987 (51)	randomised trial	serious[a]	not serious	not serious	not serious	publication bias strongly suspected[b]	24	49	MD **3.98 mmHg lower**<br>(7.55 lower to 0.4 lower)	⊕⊕◯◯<br>Low

CI: confidence interval; **MD:** mean difference

Explanations

a. Overall risk of bias rated as some concerns.

b. Considered likely that some evidence may be missing from the results.

c. Confidence interval includes the possibility of either a meaningful harm, or a trivial effect.

d. Confidence interval includes the possibility of either benefit or harm from the intervention.

e. Confidence interval includes the possibility of either a meaningful benefit from the multicomponent intervention, or a trivial effect.

f. Study included an unusual medication step-down procedure during follow-up. This would not be considered part of standard care.

g. Confidence interval includes the possibility of either a meaningful benefit from PMR, or a trivial effect.

Table S30: Hypertension GRADE profile: Comparisons that are disconnected from the networks

Certainty assessment							№ of patients		Effect	Certainty
№ of studies	Study design	Risk of bias	Heterogeneity	Indirectness	Imprecision	Other considerations	Relaxation intervention	Comparator	Absolute (95% CI)	
**Relaxation intervention plus supplementary intervention versus supplementary intervention alone, short timepoint (up to 3 months' follow-up)**										
**Systolic BP - Relaxation plus home BP monitoring versus home BP monitoring alone, up to 3 months' follow-up**										
2<br><br>Loucks 2023 (117), Palomba 2011 (140)	randomised trials	serious[a]	serious[b]	not serious	serious[c]	publication bias strongly suspected[d]	95	93	One study showed a MD of **2.8 lower** (from 7.1 lower to 1.5 higher). One study showed a MD of **5.43 lower** (from 11.76 lower to 0.9 lower).	⊕◯◯◯<br>Very low
**Diastolic BP - Relaxation plus home BP monitoring versus home BP monitoring alone, up to 3 months' follow-up**										
2<br><br>Loucks 2023 (117), Palomba 2011 (140)	randomised trials	serious[a]	not serious	not serious	serious[e]	publication bias strongly suspected[d]	95	93	One study showed a MD of **1.1 lower** (from 3.7 lower to 1.4 higher). One study showed a MD of **2.36 lower** (from 6.62 lower to 1.9 higher).	⊕◯◯◯<br>Very low
**Relaxation intervention plus supplementary intervention versus supplementary intervention alone, medium timepoint (up to 12 months' follow-up)**										
**Systolic BP - Relaxation plus home BP monitoring versus home BP monitoring alone, up to 12 months' follow-up**										
1<br><br>Loucks 2023 (117)	randomised trial	serious[a]	not serious	not serious	not serious	publication bias strongly suspected[d]	83	83	MD **4.5 lower**<br>(9 lower to 0.1 lower)	⊕⊕◯◯<br>Low
**Diastolic BP - Relaxation plus home BP monitoring versus home BP monitoring alone, up to 12 months' follow-up**										
1<br><br>Loucks 2023 (117)	randomised trial	serious[a]	not serious	not serious	serious[f]	publication bias strongly suspected[d]	83	83	MD **0.3 higher**<br>(2.5 lower to 3.1 higher)	⊕◯◯◯<br>Very low

**Relaxation intervention plus multicomponent supplementary intervention versus multicomponent supplementary intervention alone, medium timepoint (up to 12 months' follow-up)**										
**Systolic BP - Relaxation plus home BP monitoring versus home BP monitoring alone, up to 12 months' follow-up**										
1<br><br>Ziv 2013 (215)	randomised trial	serious[a]	not serious	not serious	not serious	publication bias strongly suspected[d]	58	55	MD **0.3 lower**<br>(4.45 lower to 3.85 higher)	⊕⊕◯◯<br>Low
**Diastolic BP - Relaxation plus home BP monitoring versus home BP monitoring alone, up to 12 months' follow-up**										
1<br><br>Ziv 2013 (215)	randomised trial	serious[a]	not serious	not serious	serious[g]	publication bias strongly suspected[d]	58	55	MD **1.2 lower**<br>(3.96 lower to 1.56 higher)	⊕◯◯◯<br>Very low

CI: confidence interval; **MD:** mean difference

Explanations

a. Overall risk of bias rated as some concerns.

b. One trial indicates a clinically meaningful change, the other indicates a trivial difference

c. Confidence interval of one study includes the possibility of either a meaningful benefit, or a trivial effect.

d. Considered likely that some evidence may be missing from the results.

e. Confidence interval of both studies includes the possibility of either a meaningful benefit, or a trivial effect.

f. Confidence interval includes the possibility of either a trivial difference, or a meaningful harm.

g. Confidence interval includes the possibility of either a meaningful benefit, or a trivial effect.

Table S31: Prehypertension GRADE profile

Certainty assessment							№ of patients		Effect	Certainty
№ of studies	Study design	Risk of bias	Heterogeneity	Indirectness	Imprecision	Other considerations	Relaxation intervention	Comparator	Absolute (95% CI)	
**Relaxation intervention versus passive comparator, short timepoint (up to 3 months' follow-up)**										
**Systolic BP - Meditative movement versus passive comparator, up to 3 months' follow-up**										
1<br><br>Ankolekar 2019 (26)	randomised trial	serious[a]	not serious	not serious	not serious	publication bias strongly suspected[b]	51	51	MD **3.84 lower**<br>(6.25 lower to 1.43 lower)	⊕⊕◯◯<br>Low
**Systolic BP - Multicomponent relaxation versus passive comparator, up to 3 months' follow-up**										
1<br><br>Batey 2000 (30)	randomised trial	serious[a]	not serious	not serious	not serious	publication bias strongly suspected[b]	237	304	MD **0.53 lower**<br>(2.03 lower to 0.97 higher)	⊕⊕◯◯<br>Low
**Diastolic BP - Meditative movement versus passive comparator, up to 3 months' follow-up**										
1<br><br>Ankolekar 2019 (26)	randomised trial	serious[a]	not serious	not serious	not serious	publication bias strongly suspected[b]	51	51	MD **4.95 lower**<br>(6.68 lower to 3.22 lower)	⊕⊕◯◯<br>Low
**Diastolic BP - Multicomponent relaxation versus passive comparator, up to 3 months' follow-up**										
1<br><br>Batey 2000 (30)	randomised trial	serious[a]	not serious	not serious	not serious	publication bias strongly suspected[b]	237	304	MD **0.28 lower**<br>(1.35 lower to 0.79 higher)	⊕⊕◯◯<br>Low
**Relaxation intervention versus passive comparator, medium timepoint (up to 12 months' follow-up)**										
**Systolic BP - Meditative movement versus passive comparator, up to 12 months' follow-up**										

1<br><br>Ankolekar 2019 (26)	randomised trial	serious[a]	not serious	not serious	not serious	publication bias strongly suspected[b]	51	51	MD **7.28 lower**<br>(9.58 lower to 4.98 lower)	⊕⊕◯◯<br>Low
**Diastolic BP - Meditative movement versus passive comparator, up to 12 months' follow-up**										
1<br><br>Ankolekar 2019 (26)	randomised trial	serious[a]	not serious	not serious	not serious	publication bias strongly suspected[b]	51	51	MD **6.84 lower**<br>(8.49 lower to 5.19 lower)	⊕⊕◯◯<br>Low
**Relaxation intervention versus non-specific comparator, short timepoint (up to 3 months' follow-up)**										
**Systolic BP - Breathing intervention versus non-specific comparator, up to 3 months' follow-up**										
1<br><br>Lin 2012 (112)	randomised trial	serious[a]	not serious	not serious	not serious	publication bias strongly suspected[b]	15	10	MD **5.89 lower**<br>(11.23 lower to 0.55 lower)	⊕⊕◯◯<br>Low
**Systolic BP - Biofeedback versus non-specific comparator, up to 3 months' follow-up**										
2<br><br>Lin 2012 (112),<br><br>Xu 2007 (207)	randomised trials	serious[a]	not serious	serious[c]	not serious	publication bias strongly suspected[b]	45	32	One study showed a MD **11.69 lower** (16.96 lower to 6.42 lower); one study showed a **MD 6.97 lower** (10 lower to 3.94 lower)	⊕◯◯◯<br>Very low
**Systolic BP - Massage versus non-specific comparator, up to 3 months' follow-up**										
1<br><br>Givi 2018 (73)	randomised trial	serious[a]	not serious	serious[d]	not serious	publication bias strongly suspected[b]	25	25	MD **0.56 higher**<br>(3.22 lower to 4.34 higher)	⊕◯◯◯<br>Very low
**Diastolic BP - Breathing intervention versus non-specific comparator, up to 3 months' follow-up**										
1<br><br>Lin 2012 (112)	randomised trial	serious[a]	not serious	not serious	not serious	publication bias strongly suspected[b]	15	10	MD **4.15 lower**<br>(8.16 lower to 0.14 lower)	⊕⊕◯◯<br>Low
**Diastolic BP - Biofeedback versus non-specific comparator, up to 3 months' follow-up**										

2<br><br>Lin 2012 (112),<br><br>Xu 2007 (207)	randomised trials	serious[a]	not serious	serious[c]	not serious	publication bias strongly suspected[b]	45	32	One study showed a MD **5.95 lower** (9.90 lower to 2.00 lower); one study showed a **MD 2.6 lower** (5.16 lower to 0.04 lower)	⊕◯◯◯<br>Very low
**Diastolic BP - Massage versus non-specific comparator, up to 3 months' follow-up**										
1<br><br>Givi 2018 (73)	randomised trial	serious[a]	not serious	serious[d]	not serious	publication bias strongly suspected[b]	25	25	MD **0.88 lower**<br>(2.03 lower to 0.27 higher)	⊕◯◯◯<br>Very low
**Relaxation intervention versus non-specific comparator, medium timepoint (up to 12 months' follow-up)**										
**Systolic BP - Biofeedback versus non-specific comparator, up to 12 months' follow-up**										
1<br><br>Xu 2007 (207)	randomised trial	serious[a]	not serious	not serious	not serious	publication bias strongly suspected[b]	27	22	MD **6.67 lower**<br>(9.65 lower to 3.69 lower)	⊕⊕◯◯<br>Low
**Diastolic BP - Biofeedback versus non-specific comparator, up to 12 months' follow-up**										
1<br><br>Xu 2007 (207)	randomised trial	serious[a]	not serious	not serious	serious[e]	publication bias strongly suspected[b]	27	22	MD **2.23 lower**<br>(4.85 lower to 0.39 higher)	⊕◯◯◯<br>Very low
**Relaxation intervention versus alternative relaxation intervention, short timepoint (up to 3 months' follow-up)**										
**Systolic BP - Biofeedback (intervention) versus breathing control (comparator), short timepoint, up to 3 months' follow-up**										
2<br><br>Lin 2012 (112),<br><br>Wang 2010 (199)	randomised trials	serious[a]	not serious	serious[f]	not serious	publication bias strongly suspected[b]	30	25	One study showed a MD **5.80 lower** (11.00 lower to 0.60 lower); one study showed a **MD 5.03 lower** (8.66 lower to 1.40 lower)	⊕◯◯◯<br>Very low
**Systolic BP - Progressive muscle relaxation (intervention) versus mindfulness (comparator), short timepoint, up to 3 months' follow-up**										
1	randomised trial	serious[a]	not serious	not serious	serious[g]	publication bias strongly suspected[b]	28	28	MD **1.4 lower**<br>(5.82 lower to 3.02 higher)	⊕◯◯◯<br>Very low

Hughes 2013 (86)										
**Diastolic BP - Biofeedback (intervention) versus breathing control (comparator), short timepoint, up to 3 months' follow-up**										
2<br><br>Lin 2012 (112),<br><br>Wang 2010 (199)	randomised trials	serious[a]	not serious	serious[f]	serious[h]	publication bias strongly suspected[b]	30	25	One study showed a MD **1.80 lower** (5.27 lower to 1.67 higher); one study showed a **MD 2.75 lower** (6.36 lower to 0.86 higher)	⊕◯◯◯ Very low
**Diastolic BP - Progressive muscle relaxation (intervention) versus mindfulness (comparator), short timepoint, up to 3 months' follow-up**										
1<br><br>Hughes 2013 (86)	randomised trial	serious[a]	not serious	not serious	serious[i]	publication bias strongly suspected[b]	28	28	MD **3 higher** (0.13 lower to 6.13 higher)	⊕◯◯◯ Very low
**Relaxation intervention versus alternative relaxation intervention, medium timepoint (up to 12 months' follow-up)**										
**Systolic BP - Biofeedback (intervention) versus breathing control (comparator), up to 12 months' follow-up**										
1<br><br>Wang 2010 (199)	randomised trial	serious[a]	not serious	serious[d]	not serious	publication bias strongly suspected[b]	12	10	MD **6.4 lower** (10.07 lower to 2.74 lower)	⊕◯◯◯ Very low
**Diastolic BP - Breathing intervention (intervention) versus biofeedback (comparator), up to 12 months' follow-up**										
1<br><br>Wang 2010 (199)	randomised trial	serious[a]	not serious	serious[d]	not serious	publication bias strongly suspected[b]	12	10	MD **4.15 lower** (7.68 lower to 0.62 lower)	⊕◯◯◯ Very low

CI: confidence interval; **MD:** mean difference

Explanations

a. Overall risk of bias rated as some concerns.

b. Considered likely that some evidence may be missing from the results.

c. One study included very young participants (aged 19-23). Assessment of blood pressure may have occurred immediately after a biofeedback session (assessing acute BP changes, rather than persistent BP changes).

d. Study only included women.

e. Confidence interval includes the possibility of either a meaningful benefit, or a trivial effect.

f. One study included only women.

g. Confidence intervals include the possibility of benefit from progressive muscle relaxation, as well as the possibility of a trivial difference between progressive muscle relaxation and mindfulness.

h. Confidence intervals include the possibility of benefit from biofeedback, as well as the possibility of a trivial difference between breathing and biofeedback.

i. Confidence intervals include the possibility of benefit from mindfulness, as well as the possibility of a trivial difference between progressive muscle relaxation and mindfulness.

Table S32: Hypertension and prehypertension GRADE profile: Secondary outcomes

Certainty assessment							№ of patients		Effect		Certainty
№ of studies	Study design	Risk of bias	Heterogeneity	Indirectness	Imprecision	Other considerations	Relaxation intervention	Comparator	Relative (95% CI)	Absolute (95% CI)	
**Mortality**											
**Mortality: Multicomponent intervention versus passive comparator, hypertensive population, long-term follow-up (>12 months)**											
1<br><br>Patel 1988 (146)	randomised trials	very serious[a]	not serious	not serious	very serious[b]	publication bias strongly suspected[c]	1/50 (2.0%)	0/54 (0.0%)	**RR 3.24**<br>(0.13 to 77.63)	(not estimable; no events in comparator group)	⊕◯◯◯<br>Very low
								Assumed 0.1%		**2 more per 1,000**<br>(from 1 fewer to 77 more)	
								Assumed 0.5%		**11 more per 1,000**<br>(from 4 fewer to 383 more)	
**Mortality: Meditation versus lifestyle intervention, hypertensive population, long-term follow-up (>12 months)**											
1<br><br>Schneider 1995 (166)	randomised trials	very serious[a]	not serious	not serious	very serious[b]	publication bias strongly suspected[c]	3/31 (9.7%)	6/32 (18.8%)	**RR 0.52**<br>(0.14 to 1.88)	**90 fewer per 1,000**<br>(from 161 fewer to 165 more)	⊕◯◯◯<br>Very low
**Mortality: PMR versus lifestyle intervention, hypertensive population, long-term follow-up (>12 months)**											
1<br><br>Schneider 1995 (166)	randomised trials	very serious[a]	not serious	not serious	very serious[b]	publication bias strongly suspected[c]	8/35 (22.9%)	6/32 (18.8%)	**RR 1.22**<br>(0.47 to 3.13)	**41 more per 1,000**<br>(from 99 fewer to 399 more)	⊕◯◯◯<br>Very low

Certainty assessment							№ of patients		Effect		Certainty
№ of studies	Study design	Risk of bias	Heterogeneity	Indirectness	Imprecision	Other considerations	Relaxation intervention	Comparator	Relative (95% CI)	Absolute (95% CI)	
**Mortality: Meditation versus PMR, hypertensive population, long-term follow-up (>12 months)**											
1<br>Schneider 1995 (166)	randomised trials	very serious[a]	not serious	not serious	very serious[b]	publication bias strongly suspected[c]	3/31 (9.7%)	8/35 (22.9%)	**RR 0.42**<br>(0.12 to 1.46)	**133 fewer per 1,000**<br>(from 201 fewer to 105 more)	⊕◯◯◯<br>Very low
**Mortality: Multicomponent intervention versus passive comparator, pre-hypertensive population, long-term follow-up (>12 months)**											
1<br>Batey 2000 (30)	randomised trials	serious[d]	not serious	not serious	very serious[b]	publication bias strongly suspected[c]	0/224 (0.0%)	1/296 (0.3%)	**RR 0.44**<br>(0.02 to 10.75)	**2 fewer per 1,000**<br>(from 3 fewer to 33 more)	⊕◯◯◯<br>Very low
**Cerebrovascular disease**											
**Cerebrovascular disease: Multicomponent intervention versus passive comparator, hypertensive population, long-term follow-up (>12 months)**											
1<br>Patel 1988 (146)	randomised trials	very serious[a]	not serious	not serious	very serious[b]	publication bias strongly suspected[b]	1/50 (2.0%)	0/54 (0.0%)	**RR 3.24**<br>(0.13 to 77.63)	(not estimable; no events in comparator group)	⊕◯◯◯<br>Very low
								Assumed 0.1%		**2 more per 1,000**<br>(from 1 fewer to 77 more)	
								Assumed 0.5%		**11 more per 1,000**<br>(from 4 fewer to 383 more)	
**Cardiovascular disease**											

Certainty assessment							№ of patients		Effect		Certainty
№ of studies	Study design	Risk of bias	Heterogeneity	Indirectness	Imprecision	Other considerations	Relaxation intervention	Comparator	Relative (95% CI)	Absolute (95% CI)	
**Cardiovascular disease: Multicomponent intervention versus passive comparator, hypertensive population, long-term follow-up (>12 months)**											
1<br>Patel 1988 (146)	randomised trials	very serious[a]	not serious	not serious	very serious[b]	publication bias strongly suspected[c]	0/49 (0.0%)	2/55 (3.6%)	**RR 0.22**<br>(0.01 to 4.55)	**28 fewer per 1,000**<br>(from 36 fewer to 129 more)	⨁◯◯◯<br>Very low
**Cardiovascular disease: Meditation versus lifestyle intervention, hypertensive population, long-term follow-up (>12 months)**											
1<br>Schneider 1995 (166)	randomised trials	very serious[a]	not serious	not serious	very serious[b]	publication bias strongly suspected[c]	3/28 (10.7%)	7/30 (23.3%)	**RR 0.46**<br>(0.13 to 1.60)	**126 fewer per 1,000**<br>(from 203 fewer to 140 more)	⨁◯◯◯<br>Very low
**Cardiovascular disease: PMR versus lifestyle intervention, hypertensive population, long-term follow-up (>12 months)**											
1<br>Schneider 1995 (166)	randomised trials	very serious[a]	not serious	not serious	very serious[b]	publication bias strongly suspected[c]	7/31 (22.6%)	7/30 (23.3%)	**RR 0.97**<br>(0.39 to 2.43)	**7 fewer per 1,000**<br>(from 142 fewer to 334 more)	⨁◯◯◯<br>Very low
**Cardiovascular disease: Meditation versus PMR, hypertensive population, long-term follow-up (>12 months)**											
1<br>Schneider 1995 (166)	randomised trials	very serious[a]	not serious	not serious	very serious[b]	publication bias strongly suspected[c]	3/28 (10.7%)	7/31 (22.6%)	**RR 0.47**<br>(0.14 to 1.66)	**120 fewer per 1,000**<br>(from 194 fewer to 149 more)	⨁◯◯◯<br>Very low

CI: confidence interval; **RR:** risk ratio

Explanations

a. Overall risk of bias rated as high

b. Confidence interval includes the possibility of both substantial benefit and substantial harm.

c. Considered likely that some evidence may be missing from the results.

d. Overall risk of bias rated as some concerns.

Equality, Diversity and Inclusion (EDI) perspectives

We followed the PROGRESS+ guidance when extracting data from included studies (217, 218). However, there was a paucity of information regarding EDI characteristics in the studies. The most well reported characteristics were age and sex.

Age

The mean age of included participants was 52.12 years, across 159 studies in which this was reported. Only three studies failed to provide any information regarding the age of participants (26, 76, 77). The remaining 21 studies did report some information on either the range of ages of participants, or specific age-restrictions for inclusion in the study.

Sex

Across 171 studies, on average, 52% participants were male. However, this varied widely, with a number of studies recruiting exclusively male (17, 20, 35, 39, 43, 71, 96, 125, 148, 175) or female participants (19, 27, 28, 67, 73, 79, 102, 160, 199, 201). Twelve studies did not provide information on the sex of participants (26, 49, 77, 84, 108-110, 116, 134, 177, 206, 207).

Residence

Eighteen studies provided some information on the residence of participants. The majority of these indicated that participants were from urban communities, although two studies included those from more rural communities (103, 180), and one included people from both urban and rural locations (95).

Ethnicity/Race/Language

Fifty-five studies reported some information on ethnicity of participants, or language requirements for inclusion. In the studies that reported ethnicity or race, the majority of participants were described as White, although a small number of studies exclusively recruited participants described as Black/African American (43, 166, 169, 170, 173, 201, 204) or Chinese (111, 185, 186, 209, 213).

Employment

Thirty-two studies reported some information on the employment status of participants. Of the studies that reported employment, the majority of participants who were working, except for four studies that included predominantly retired/unemployed participants (46, 58, 185, 209). Eleven studies specifically recruited workers at their place of employment (17, 18, 26, 48, 51, 53, 63, 90, 122, 179, 201) and 2 studies recruited university students (112, 207).

Religion

A single study presented information on the religious background of participants (46).

Education

Forty-five studies reported some information on educational background of participants. This was very varied across the included studies, although we noted that eight studies limited participation in the trial to those with certain minimum educational attainment (15, 19, 61, 85, 132, 136, 137, 193).

Socioeconomic status

Twelve studies provided information on the annual income of participants (16, 46, 47, 53, 62, 119, 153, 169, 170, 173, 185, 204). Five studies provided some information on socioeconomic status, describing their participants as “blue collar workers”, “white collar workers” or “middle class” (17, 48, 63, 82, 191). One study indicated the number of participants receiving social security (34), and another reported the Hollingshead Index (219) to provide some information on socioeconomic status (82).

Social capital

Thirty studies reported on marital status of the participants (28, 30, 34, 43, 46, 47, 51, 53, 58, 62, 72, 75, 90, 92, 93, 99, 103, 114, 119, 126, 130, 136, 137, 153, 169, 170, 176, 185, 193, 209). Most studies included a majority of married participants. In only five studies were the majority of participants unmarried/widowed/divorced (34, 53, 99, 169, 170). In one study, participants were required to have a spouse or family member with who they could attend relaxation sessions (196).

Additional characteristics

A number of studies specifically excluded those with mental health problems or psychiatric disorders (23, 30, 39, 40, 58, 61, 66, 80, 89, 97, 99, 132, 139, 142, 164, 165, 169, 170, 173, 176, 178, 193, 209, 213). Two studies exclude those with alcoholism (170, 178).

Six studies excluded individuals who were unable to operate devices required for the study, such as a smartphone, CD player or individuals without internet access (16, 47, 66, 101, 126, 139). Seven studies excluded individuals with any form of cognitive impairment (22, 44, 46, 61, 115, 126, 186). Individuals with sight or hearing impairments were excluded from three studies (16, 142, 165), and those with physical disorders that may affect relaxation techniques used were excluded from four studies (179, 186, 203, 213).

Three studies intentionally recruited individuals from communities that may be under-represented in research: two recruited residents of elderly care homes (34, 188), and one recruited individuals with mild cognitive impairment (204).

Table S33: PROGRESS-plus characteristics

Study	Age, mean years (SD)	Age range/other details	Residence	Ethnicity/Race/Language	Occupation	Percentage male	Religion	Education	Socioeconomic status	Social capital	Additional characteristics	Notes
Achmon 1989 (15)	40.66 (8.58)	25-60	NR	NR	NR	73%	NR	Mean 13.03 (SD 3.19) school years; minimum of 8 years for inclusion	NR	NR	NR	
Adams 2018 (16)	35.1 (12.5)	>/=21 years	Southeastern US coastal city	0.484 African American;<br>0.516 Non-Hispanic white.<br>Excluded those unable to speak/hear/ understand English	NR	55%	NR	0.094 high school; 0.203 trade school; 0.516 college;<br>0.188 not reported.	Annual income <$15000 0.328; $15-<30K 0.359;<br>$30-<50K 0.188;<br>$50-<75K 0.031;<br>>$75K 0.016; not reported 0.078	NR	Excluded those who could not hear/see app, were unable to use a smartphone, or with poor home cellular coverage	
Adsett 1989 (17)	46.57 (8.23)	30-65	NR	English language (inclusion criterion)	Employed	100%	NR	NR	Described as 'blue collar' workers.	NR	NR	All workers (employed) in a steel mill.
Agras 1987 (18)	52.82 (NR)	NR	NR	0.89 white	Employed	82%	NR	NR	NR	NR	NR	All workers (employed).

Ahmadpanah 2016 (19)	46.49 (2.33)	NR	NR	NR	NR	0%	NR	Inclusion criterion: "upper diploma"	NR	NR	NR	
Aivazyan 1988a (20)	35.76 (8.84)	20-45	NR	NR	NR	100%	NR	NR	NR	NR	NR	
Aivazyan 1988b (21)	39.9 (1.46)	20-50	NR	NR	NR	78%	NR	NR	NR	NR	NR	
Altena 2009 (22)	59.5 (11.17)	NR	NR	Dutch language (inclusion criterion)	NR	50%	NR	NR	NR	NR	Required sufficient knowledge of Dutch and cognitive abilities to handle devices.	
Amigo 1997 (23)	43 (12.59)	18-60	NR	NR	NR	53%	NR	NR	NR	NR	Excluded participants with psychiatric illness.	
Anderson 2010 (24)	53.15 (2.78)	NR	NR	NR	NR	53%	NR	NR	NR	NR	NR	Outcomes reported separately for men and women.
Anjana 2022 (25)	46.64 (8.99)	25-60	NR	NR	NR	45%	NR	NR	NR	NR	NR	
Ankolekar 2019 (26)	NR (NR)	NR	NR	NR	Employed (security personnel)	NR	NR	NR	NR	NR	NR	
Arslan 2021 (27)	NR (NR)	0.067 aged 30-39; 0.056 aged 40-49; 0.367 aged 50-59; 0.322 aged 60-69;	"Residing in the city center"; excluded those living	NR	NR	0%	NR	NR	NR	NR	NR	

		0.144 aged 70-79; 0.044 aged 80-89.	in the country.									
Babak 2022 (28)	49 (1.94)	30-59	NR	NR	0.973 (intervention) 0.947 (control) unemployed	0%	NR	Literacy: 0.784 (intervention) 0.846 (control)	NR	Married: 0.881 (intervention) 0.872 (control)	NR	
Balasubramanian 2012 (29)	52.57 (4.96)	35-60	NR	NR	NR	65%	NR	NR	NR	NR	NR	
Batey 2000 (30)<br><br>(Related articles: Whelton 1992 (31), The Trials of Hypertension Prevention Collaborative Research Group 1992 (32), Whelton 1997 (33))	43.17 (6.73)	30-54	NR	White: 0.839 (intervention) 0.838 (control); Black: 0.132 (intervention) 0.15 (control)	Employed full time: 0.917 (intervention) 0.897 (control)	71%	NR	College graduates: 0.566 (intervention) 0.506 (control)	NR	Married: 0.777 (intervention) 0.769 (control)	Excluded people with psychiatric disorders, taking anti-psychotics/ anti-depressants and those with disabilities e.g. severe arthritis or blindness.	
Bekiroglu 2013 (34)	NR (NR)	Range 60-89.<br><br>0.133 aged 60-69, 0.5 aged 70-79, 0.367 aged 80-89	NR	NR	NR	57%	NR	0.317 illiterate, 0.033 literate, 0.417 primary school education, 0.133 secondary school education, 0.10 university education	0.717 social security	0.167 married, 0.833 single	Residents of elderly home.	
Bennett 1991 (35)	46 (NR)	NR	NR	NR	NR	100%	NR	NR	NR	NR	NR	
Blanchard 1979 (36)	39.5 (NR)	23-56	NR	NR	NR	48%	NR	NR	NR	NR	NR	
Blanchard 1984 (37)	48.6 (9.2)	NR	NR	NR	NR	55%	NR	NR	NR	NR	NR	

(Related article: Blanchard 1986 (38))												
Blanchard 1988 (39)	38.26 (9.87)	21-61		All white	NR	100%	NR	NR	NR	NR	Excluded those with psychiatric illness.	
Blanchard 1993 (40)	51.55 (NR)	NR	NR	NR	NR	61%	NR	NR	NR	NR	Excluded those with psychiatric illness.	
Blanchard 1996 (41)	50.5 (6.99)	32-62	NR	"minority" 0.095	NR	67%	NR	NR	NR	NR	NR	
Blom 2014 (42)	55.99 (11.49)	20-75	NR	0.83 white	0.81 employed	37%	NR	Education post-secondary or greater: 0.82 (intervention) 0.90 (control)	NR	NR	NR	
Bosley 1989 (43)	57 (NR)	42-68	NR	Black	0.66 employed, 0.24 retired, 0.17 unemployed	100%	NR	NR	NR	0.66 married, 0.1 divorced, 0.07 widowed, 0.02 single, 0.15 unknown	NR	Assumed all veterans (recruitment from Veteran hospital).
Brauer 1979 (44)	57.23 (NR)	NR	NR	NR	NR	86%	NR	NR	NR	NR	Excluded people with major memory or learning defects.	Assumed all veterans (recruitment from Veteran hospital).
Canino 1994 (45)	35 (2)	25-46	NR	NR	NR	67%	NR	NR	NR	NR	NR	
Chan 2018 (46)	64.4 (9.75)	30-91	NR	NR	0.44 retired, 0.25 full-time employment, 0.12 part-time employment, 0.15	46%	0.66 no religion, 0.11 Christ-ian, 0.04	NR	monthly income </=10,000 Hong Kong dollars 0.55; 10,001-	0.679 married; 0.1 single, 0.05 separated, 0.17 widowed	Excluded those with severe sensory or cognitive impairment	

					housewife, 0.04 unemployed		Catholic, 0.17 Buddhist, 0.02 Taoist		20,000 HKD 0.17; 20001-30000 HKD 0.08; ≤30001 0.08 (some missing data); 0.17 financial support from allowance		or musculoskeletal problems; 0.14 living alone.	
Chandler 2020 (47)	45.05 (13.43)	18-90	NR	Non-Hispanic White: 0.487 (intervention) 0.579 (control),<br><br>African American 0.513 (intervention) 0.421 (control); English language required for inclusion.	Employed full time 0.667 (intervention) / 0.733 (control), part time 0.133 (intervention / 0 (control), retired/ disabled 0.2 (intervention) / 0.266 (control)	49%	NR	Education to high school or less 0.067, partial/college graduate 0.933	income $0–25,000: 0.2 (intervention) 0.333 (control), $25–50,000: 0.133 (intervention) 0.133 (control) >$50,000: 0.533 (intervention) 0.533 (control), NR 0.133 (intervention) 0 (control)	single: 0.067 (intervention) 0.2 (control), married or living with significant other: 0.733 (intervention) 0.667 (control) separated or divorced: 0.133 (intervention) 0.067 (control); widowed 0.067 (intervention) 0.067 (control)	Excluded those unable to use a smartphone.	
Charlesworth 1984 (48)	51 (9.74)	23-65	NR	NR	All employed	80%	NR	NR	“White collar workers”	NR	All employees of a local corporation.	
Chen 2016a (49)	66.3 (5.8)	NR	NR	NR	NR	NR	NR	NR	NR	NR	NR	
Chen 2016b (50)	21.5 (0.18)	19-23	NR	NR	NR	75%	NR	NR	NR	NR	NR	
Chesney 1987 (51)	47.38 (NR)	0.42 aged 50-69 years	NR	0.89 White, 0.03 Black, 0.07 Asian	All employed	89%	NR	4-year college degree: 0.603	NR	Married: 0.741 (intervention) 0.737 (control)	NR	All workers (employed).

								(intervention) 0.526 (control)				
Cheung 2005 (52)	54.4 (9.05)	18-75	NR	NR	NR	42%	NR	NR	NR	NR	NR	
Clemow 2018 (53)	48.5 (8.7)	18-70	NR	0.15 White non-Hispanic, 0.15 White Hispanic, 0.46 Black non-Hispanic, 0.09 Black Hispanic, 0.04 Asian/Indian, 0.04 Asian/Pacific Islander, 0.07 Other; Ethnicity: 0.28 Latino, 0.72 non-Latino	employed	23%	NR	0.51 some college or less, 0.49 college graduate or graduate school	0.56 income =<$50,000, 0.44 income >$50,000	0.22 never married, 0.46 currently married, 0.32 separated, divorced, or widowed	NR	
Cohen 1983 (54)	44.47 (NR)	26-72	NR	NR	NR	43%	NR	NR	NR	NR	NR	
Cohen 2011 (55)	48.24 (11.96)	22-69	NR	African American: 0.39 (intervention) 0.44 (control); Asian/native Hawaiian/ Pacific Islander: 0.07 (intervention) 0.03 (control); Caucasian: 0.46 (intervention) 0.5 (control); Hispanic: 0.07 (intervention), 0 (control); Other: 0.02	NR	50%	NR	NR	NR	NR	NR	

				(intervention) 0.03 (control)								
Cohen 2016 (56)	47.45 (12.96)	18-80	NR	African American: 0.42 (intervention) 0.46 (lifestyle intervention), 0.61 (combination intervention); Caucasian 0.53 (intervention) 0.48 (lifestyle intervention) 0.39 (combination intervention); Asian 0.02 (intervention) 0.02 (lifestyle intervention) 0 (combination intervention); Hispanic: 0.02 (intervention) 0.02 (lifestyle intervention) 0 (combination intervention)	NR	49%	NR	NR	NR	NR	NR	
Cottier 1984 (57)	34.69 (7.71)	18-50	NR	NR	NR	73%	NR	NR	NR	NR	NR	
Cramer 2018 (58)<br><br>(Related article: Guaman 2022 (59))	58.7 (9.5)	Minimum 18	NR	NR	Employed full-time: 0.08 (meditative movement) 0.2 (breathing) 0.32 (control); Employed part-time 0.32 (meditative	28%	NR	Educated to year 9/ lower secondary school: 0.2 (meditative movement) 0.12 (breathing) 0.32 (control); Educated to year 10/ intermediate	NR	Married or stable partnership 0.16 (meditative movement) 0.16	Excluded those with psychiatric co-morbidities.	

					movement) 0.36 (breathing) 0.16 (control); Unemployed 0.6 (meditative movement) 0.44 (breathing) 0.52 (control)			secondary school: 0.56 (meditative movement) 0.4 (breathing) 0.28 (control); University entrance qualification: 0.08 (meditative movement) 0.16 (breathing) 0.2 (control); University/ technical University degree 0.16 (meditative movement) 0.32 (breathing) 0.16 (control)		(breathing) 0.28 (control)		
de Barros 2017 (60)	50.41 (9.94)	NR	NR	NR	NR	41%	NR	NR	NR	NR	NR	
de Fatima Rosas Marchiori 2015 (61)	67.09 (5.11)	Minimum 60	NR	NR	NR	36%	NR	Minimum of 4 years of formal school education for inclusion.<br>Higher education: 0.742 (control) 0.673 (intervention)	NR	NR	Excluded those with psychiatric disorders or severe cognitive impairment.	
Dhungana 2021 (62)	47.7 (10.7)	18-70	NR	NR	Employee: 0.25 (intervention) 0.15 (control); Self-employed: 0.33 (intervention) 0.42 (control); Home-maker 0.39	52%	NR	Median 5 years (IQR 11) intervention, 6 years (IQR 11.5) control	Annual household income median 300,000 Nepalese rupees (IQR 400000) (intervent-ion) 200,000	Married: 0.90 (intervention) 0.93 (control); Other (unmarried, widow): 0.1 (intervention) 0.7 (control)	NR	

					(intervention) 0.27 (control); Other 0.03 (intervention) 0.17 (control)				(IQR 325000) (control)			
Drazen 1982 (63)	40.3 (NR)	22-62	NR	NR	All employed	73%	NR	NR	"White collar workers"	NR	NR	
Dusek 2008 (64)	66.8 (7.23)	Minimum 55 years	NR	White 0.9 (intervention) 0.84 (control); African-American 0.07 (intervention) 0.10 (control); Other 0.06 (intervention) 0.06 (control)	NR	45%	NR	NR	NR	NR	NR	
Elavally 2020 (65)	NR (NR)	Aged 35-75; 0.14 36-45, 0.10 46-55, 0.29 56-65, 0.47 66-75	NR	NR	NR	56%	NR	NR	NR	NR	NR	
Elliot 2004 (66)	59 (10)	40-75	NR	NR	NR	50%	NR	NR	NR	NR	Excluded those with a major psychiatric disorder/ panic disorder, and those unable to operate a portable music player.	
Fetter 2020 (67)	59.09 (3.81)	45-68	NR	NR	NR	0%	NR	NR	NR	NR	NR	

Frankel 1978 (68)	45.82 (10.24)	NR	NR	0.36 Black, 0.64 White	NR	55%	NR	NR	NR	NR	NR	
Friedman 1977a (69)<br><br>(Related article Friedman 1978 (70))	47.58 (NR)	23-60	NR	NR	NR	83%	NR	NR	NR	NR	NR	
Friedman 1977b (69)<br><br>(Related article Friedman 1978 (70))	47.73 (NR)	29-59	NR	NR	NR	80%	NR	NR	NR	NR	NR	
Garcia Vera 1997 (71)	45.36 (8.83)	NR	NR	NR	NR	100%	NR	NR	NR	NR	NR	
Gay 2007 (72)	47.2 (11.76)	22-60	NR	NR	0.63 worked "in a profession"	40%	NR	NR	NR	0.83 married, 0.07 divorced, 0.1 single	NR	
Givi 2018 (73)	NR (NR)	18-60	NR	NR	NR	0%	NR	NR	NR	NR	NR	
Grossman 2001 (74)	51.09 (9.19)	25-75	NR	NR	NR	70%	NR	NR	NR	NR	NR	
Hafner 1982 (75)	48.9 (NR)	25-68	NR	NR	NR	57%	NR	NR	NR	0.86 married (reported only for treatment groups, not controls)	NR	Sex based on two treatment groups only, not reported for controls
Hager 1978 (76)	NR (NR)	NR	NR	NR	NR	50%	NR	NR	NR	NR	NR	
Haghighat 2021 (77)	NR (NR)	NR	NR	NR	NR	NR	NR	NR	NR	NR	NR	
Hagins 2014 (78)	54.54 (11.08)	21-70	NR	English speaking for inclusion.	NR	14%	NR	NR	NR	NR	NR	

				African American: 0.862 (intervention) 0.844 (control); Non-Hispanic white: 0.027 (intervention) 0.031 (control); Other: 0.111 (intervention) 0.125 (control)								
Hasandokht 2015 (79)	54.65 (4.87)	NR	NR	NR	NR	0%	NR	Years of education completed: mean 8.2 (SD2.4) (intervention), mean 8.5 (SD 2.9) (control)	NR	NR	NR	"we will try to control the socioeconomic state as a confounder in the statistical analysis" but no relevant information reported.
Hatch 1985 (80)	51.1 (NR)	21-70	NR	0.81 “Anglo”, 0.17 Hispanic, 0.02 Black	NR	40%	NR	NR	NR	NR	Excluded those with psychiatric disorders.	
Henderson 1998 (81)	54 (NR)	38-67	NR	NR	NR	60%	NR	NR	NR	NR	NR	
Hernandez Reif 2000 (82)	51.6 (8.8)	NR	NR	0.6 Caucasian, 0.27 Hispanic, 0.13 African American	NR	30%	NR	NR	middle socio-economic status (mean 2.3 on the ref. Hollingshead index(219))	NR	NR	
Hoelscher 1986 (83)	51.1 (13)	NR	NR	NR	NR	52%	NR	NR	NR	NR	NR	

Hoelscher 1987 (84)	51.9 (11.4)	NR	NR	NR	NR	NR	NR	NR	NR	NR	NR	
Howorka 2013 (85)	49.3 (11.7)	18–78	NR	NR	NR	53%	NR	"structured education" stated as an inclusion criterion. Unclear if this refers to general education level, or specific health-related education.	NR	NR	NR	
Hughes 2013 (86)	50.3 (6.5)	30-60	NR	0.91 white	0.66 employed	43%	NR	Mean 15.7 (SD 2.6) years of education	NR	NR	NR	
Huijuan 2021 (87)	77.2 (4.55)	50-90	NR	NR	NR	40%	NR	NR	NR	NR	NR	
Im-Oun 2018 (88)	51.65 (8.77)	40-80	NR	NR	Farmer: 0.035 (intervention) 0 (control); Merchant: 0.14 (intervention) 0.088 (control); Housework: 0.158 (intervention) 0.053 (control); Self-employed: 0.105 (intervention) 0.123 (control); Government officer:0.281 (intervention) 0.526 (control); No	39%	NR	NR	NR	NR	NR	

					occupation: 0.281 (intervention) 0.211 (control)							
Irvine 1986 (89)	47.75 (8.5)	34-65	NR	NR	NR	53%	NR	NR	NR	NR	Excluded those with a significant psychiatric disorder.	
Irvine 1991 (90)	46.25 (8.28)	25-64	NR	NR	All employed	82%	NR	College education or higher 0.74 (intervention) 0.7 (control)	"The worksites were predomin-antly white collar"	Married 0.8 (intervention) 0.83 (control)	NR	All workers.
Ismail 2023 (91)	69.75 (4.16)	min. 65	NR	Language requirement: Arabic	NR	37%	NR	NR	NR	NR	NR	
Jacob 1985 (92)	54 (NR)	37-65	NR	NR	NR	54%	NR	NR	NR	0.28 single, 0.02 widowed, 0.7 married	NR	
Jacob 1992 (93)	48.82 (10.1)	38-68, median 48	NR	0.89 White, 0.11 Black	NR	68%	NR	NR	NR	0.68 married	NR	
Johnston 1993 (94)	46.6 (8.41)	23-59	NR	NR	NR	48%	NR	NR	NR	NR	NR	
Jones 2010 (95)	51.5 (4.67)	35-65	Mixed urban and rural	NR	NR	35%	NR	NR	NR	NR	NR	
Jorgensen 1981 (96)	54.35 (NR)	NR	NR	NR	NR	100%	NR	NR	NR	NR	NR	
Kalmatayeva 2014 (97)	48.03 (3.94)	NR	NR	NR	NR	49%	NR	NR	NR	NR	Excluded those with psychiatric illness.	

Katsarou 2014 (98)	63.43 (11.34)	min. 18	NR	NR	NR	39%	NR	NR	NR	NR	NR	
Kohn 2023a (99)<br><br>(Related article: Kohn 2023b (100), additional data supplied by the author)	72.6 (7.9)	60-93	NR	White: 0.82 (control) 0.90 (intervention).<br><br>Excluded those who could not communicate in English	NR	28%	NR	College educated: 0.51 (control) 0.61 (intervention)	NR	Married/ partnered: 0.33 (control) 0.35 (intervention)	Excluded people with various mental health disorders (major depressive disorder, psychosis, substance-use disorder, use of anti-psychotics or suicidality).	
Kow 2018 (101)	61.06 (9.65)	NR	NR	Ethnicity: Malay: 0.067 (control) 0.048 (intervention; Chinese: 0.889 (control) 0.857 (intervention) Indian: 0.022 (control) 0.071 (intervention); Other: 0.022 (control) 0.024 (intervention)	NR	53%	NR	Primary education 0.133 (control) 0.19 (intervention; Secondary education: 0.644 (control) 0.524 (intervention; Tertiary 0.156 (control) 0.214 (intervention); No education: 0.067 (control) 0.071 (intervention)	NR	NR	Required access to and ability to operate a CD player.	
Kretzer 2013 (102)	NR (NR)	Minimum 21 years	NR	0.89 Caucasian, 0.06 Black, 0.01 Hispanic, 0.03 other	NR	0%	NR	NR	NR	NR	NR	
Kumar 2017 (103)	NR (NR)	35-60; "Majority of subjects in the experiment-al	rural	NR	Labourers: 0.67 (intervention) 0.55 (control)	40%	NR	Primary education: 0.61 (intervention) 0.5 (control)	NR	All married; 'Living in nuclear family': 0.89	NR	

		group (38.89%) and in the control group (30%) belonged to the age group of 45-49 years"								(intervention) 0.87 (control)		
Kunikullaya 2015 (104)<br><br>(Related article: Kunikullaya 2016 (105))	46.69 (8.46)	30-60	NR	NR	NR	67%	NR	NR	NR	NR	NR	
LaGrone 1988 (106)	50.8 (NR)	33-66	NR	NR	NR	17%	NR	NR	NR	NR	NR	
Landman 2013 (107)	64.45 (8.09)	min. 18	NR	Language requirement: Dutch.<br><br>All white.	NR	63%	NR	NR	NR	NR	NR	
Latha 1991 (108)	NR (NR)	45-70	NR	NR	NR	NR	NR	NR	NR	NR	NR	Almost no baseline information reported
Lee 1988 (109)	44 (6)	32-51	NR	NR	NR	NR	NR	NR	NR	NR	NR	
Lee 2003 (110)	56.25 (6.53)	NR	NR	NR	NR	NR	NR	NR	NR	NR	NR	
Li 2024 (111)	49.3 (11.9)	18-65	NR	Chinese	NR	49%	NR	NR	NR	NR	NR	
Lin 2012 (112)	22.3 (NR)	Young adults (sopho-mores)	NR	NR	University students	84%	NR	NR	NR	NR	NR	
Lin 2022 (113)	64 (4.21)	58-70	NR	NR	NR	66%	NR	NR	NR	NR	NR	

Linden 2001 (114)	54.82 (NR)	28-75	NR	White: 0.85 (intervention) 0.91 (control); Asian: 0.15 (intervention) 0.06 (control); African 0 (intervention) 0.03 (control)	Currently employed: 0.81 (intervention) 0.67 (control)	72%	NR	NR	NR	Married/co-habiting: 0.78 (intervention) 0.82 (control)	NR	
Logtenberg 2007 (115)	61.85 (6.73)	Minimum 18 years	NR	NR	NR	44%	NR	NR	NR	NR	Excluded people with insufficient cognitive abilities to operate study device.	
Lokesh 2017 (116)	NR (NR)	25-50	NR	NR	NR	NR	NR	NR	NR	NR	NR	
Loucks 2023 (117)<br><br>(Related article: Polcari 2022 (118))	59.75 (12.9)	22-84, minimum 18 years.	NR	White: 0.83 (control) 0.80 intervention); Asian: 0.02 (control) 0.03 (intervention); Black/African American: 0.02 (control) 0.069 (intervention); Native American: 0 (control) 0.03 (intervention); Hispanic: 0.05 (control) 0.03 (intervention); Other: 0.05 (control) 0.05 (intervention); Unknown; 0.03 (control) 0 (intervention).	NR	61%	NR	0.517 college degree; High school education: 0.122 (control) 0.102 (intervention); Associates degree: 0.051 (control) 0.031 (intervention); College: 0.286 (control) 0.347 (intervention); Graduate school: 0.439 (control) 0.418 (intervention); Other: 0.102 (control) 0.102 (intervention).	NR	NR	NR	"Classes were held in Providence, Rhode Island, at Brown University, and at a local community health center located in a low-income, urban neighborhood." (This may not reflect the sample characteristics).

				All required to speak English.								
Ma 2018 (119)	69 (9.37)	≥60 years	All living in Tianhe District, Guangzhou (urban)	NR	NR	69%	NR	Secondary school and below: 0.49 (intervention) 0.50 (control); High school and above: 0.51 (intervention) 0.50 (control).	Income per month (Yuan) <3000:) 0.242 (intervent-ion 0.329 (control); 3000-4999: 0.481 (intervent-ion) 0.506 (control); ≥5000: 0.177 (intervent-ion) 0.165 (control)	Married: 0.873 (intervention) 0.886 (control); Living with children: 0.709 (intervention), 0.722 (control).	NR	
Manikonda 2008 (120)	NR (NR)	29-71; median 53 range 42-71 (intervent-ion), median 52 range 29-69 (control)	NR	NR	NR	65%	NR	NR	NR	NR	NR	
McCaffrey 2005 (121)	56.45 (NR)	NR	Resident or Songkhla Province, Southern Thailand (urban)	NR	NR	35%	NR	Primary school: 0.296 (intervention) 0.296 (control); Secondary school: 0.148 (intervention) 0.111 (control); Diploma: 0.148 (intervention) 0.111 (control); Bachelor's or higher: 0.407	NR	NR	NR	

								(intervention) 0.48 (control)				
McCraty 2003 (122)	45.97 (6.55)	35-59	NR	NR	All employed by global IT company	72%	NR	NR	NR	NR	NR	
McGrady 1981 (123)	49.53 (NR)	NR	NR	Black: 0.05 (intervention) 0 (control); White: 0.95 (intervention) 1 (control)	NR	32%	NR	NR	NR	NR	NR	
McGrady 1994 (124)	48.31 (NR)	NR	NR	Black: 0.27 (intervention) 0.19 (control); White: 0.73 (intervention) 0.81 (control)	NR	39%	NR	NR	NR	NR	NR	
Mir 2021 (125)	21.03 (1.66)	18-25	NR	NR	NR	100%	NR	NR	NR	NR	NR	
Misra 2019 (126)	60.8 (11.5)	Minimum 18	NR	0.89 white. Excluded non-English speakers;	0.61 employed part-/full-time	52%	NR	0.63 college/ graduates	NR	Married 0.82	Excluded participants with cognitive problems and those without internet/DVD access.	
Modesti 2010 (127)	59.08 (12.21)	40-75	NR	NR	NR	60%	NR	NR	NR	NR	NR	
Modesti 2015 (128)	52.25 (9.22)	30-75	NR	NR	NR	30%	NR	NR	NR	NR	NR	
Mohammed Elsheikh 2023 (129)	65.85 (4.03)	60-75	NR	NR	NR	43%	NR	NR	NR	NR	NR	

Mohebbi 2014 (130)<br><br>(Related article: Moghadasi 2021 (131))	57.99 (8.37)	30-70	NR	NR	0.044 employee, 0.111 self-employed, 0.267 retired, 0.578 housewife	42%	NR	0.111 above diploma, 0.267 diploma, 0.489 below diploma, 0.133 illiterate	NR	0.922 married, 0.067 widowed, 0.011 single	NR	
Momeni 2016 (132)	47 (7)	35-60 years	NR	NR	NR	58%	NR	Diploma: 0.867 (intervention) 0.8 (control); Bachelor 0.133 (intervention) 0.20 (control). All had at least a high school diploma (for inclusion).	NR	NR	Excluded people with history of drug abuse, and those receiving psycho-logical therapies	
Mourya 2009 (133)	NR (NR)	20-60	NR	NR	NR	52%	NR	NR	NR	NR	NR	
Murugesan 2000 (134)	NR (NR)	35-65	NR	NR	NR	NR	NR	NR	NR	NR	NR	
Nakao 1997 (135)	56 (8)	35-65	NR	NR	NR	33%	NR	NR	NR	NR	NR	
Nakao 2000 (136)	56.09 (9.15)	30-65 years	NR	NR	NR	30%	NR	All high school or college graduates with basic calculation skills	NR	All married	NR	
Nejati 2015 (137)	43.4 (5.01)	38-48	NR	NR	NR	53%	NR	Included only those with high school diploma or higher.<br><br>3rd grade of high school: 0.2 (intervention) 0.2 (control); High school diploma: 0.46 (intervention) 0.4 (control); Bachelor: 0.33	NR	All married	NR	

								(intervention) 0.4 (control)				
Nolan 2010 (138)	55.42 (6.82)	35-64	NR	Included only English/French speakers	NR	43%	NR	NR	NR	NR	NR	
Olsson 2010 (139)	56.55 (8.84)	25-75	NR	NR	NR	39%	NR	NR	NR	NR	Excluded those without computer/ internet access and ability. Excluded people with psychiatric co-morbidity.	
Palomba 2011 (140)	36.18 (9.25)	22-55	NR	NR	NR	86%	NR	NR	NR	NR	NR	
Pandey 2023 (141)	63.05 (11.76)	Minimum 18	NR	NR	NR	70%	NR	NR	NR	NR	NR	
Pandic 2008 (142)	68.79 (8.68)	35-85	NR	Must have understood Swedish.	NR	26%	NR	NR	NR	NR	Excluded those with blindness or deafness and those with a major psychiatric diagnosis.	
Park 2014 (143)	NR (NR)	19-65; median 52 (IQR 43-61) intervention, median 54 (IQR 45-62) control	NR	NR	NR	65%	NR	NR	NR	NR	NR	
Park 2017 (144)	53.69 (7.74)	19-65	NR	NR	NR	67%	NR	NR	NR	NR	NR	

Patel 1975 (145)	59.05 (NR)	34-75	NR	NR	NR	38%	NR	NR	NR	NR	NR	
Patel 1988 (146)	NR (NR)	35-64;<br><br>0.20 35-44, 0.32 45-54, 0.48 >55	NR	NR	NR	50%	NR	NR	NR	NR	NR	
Pathan 2023 (147)	47.8 (6.78)	30-60	NR	NR	NR	72%	NR	NR	NR	NR	NR	
Patil 2014 (148)	68.93 (5.47)	60-80	NR	NR	NR	100%	NR	NR	NR	NR	NR	
Perez 2009 (149)	57.23 (8.94)	Minimum 18	NR	NR	NR	48%	NR	NR	NR	NR	NR	
Plaugher 2002 (150)	37.61 (8.29)	Maximum 50	NR	NR	NR	43%	NR	NR	NR	NR	NR	
Ponte Marquez 2019 (151)<br><br>(Related article: Concepcion 2022 (152))	56.5 (7.77)	18-70	NR	NR	0.738 'occupation-ally active'/ employed	43%	NR	NR	NR	NR	NR	
Pukdeesamai 2023 (153)	50.16 (5.82)	35-60	NR	NR	NR	86%	NR	Primary or no education: 0.296 (intervention) 0.333 (control); Secondary or higher: 0.704 (intervention) 0.667 (control)	Income per month (Thai Baht) <5000: 0.889 (inter-vention) 0.875 (control);<br><br>≥ 5000: 0.111 (inter-vention) 0.125 (control)	Married: 0.926 (intervention) 0.792 (control); Single/ divorced: 0.074 (intervention) 0.208 (control)	NR	
Punita 2016 (154)	43.38 (7.56)	35-55	NR	NR	NR	80%	NR	NR	NR	NR	NR	

(Related article: Pushpanathan 2015 (155))												
Ranjbar 2007 (156)	55 (NR)	NR	NR	NR	NR	49%	NR	NR	NR	NR	NR	
Roche 2014 (157)	57.8 (8.04)	40-71	NR	NR	NR	45%	NR	NR	NR	NR	NR	
Roche 2017 (158)	57.69 (9.42)	40-70	NR	NR	NR	36%	NR	NR	NR	NR	NR	
Rudy 1995 (159)	NR (NR)	28-60	NR	0.98 Caucasian, 0.02 Black	NR	65%	NR	NR	NR	NR	NR	
Saensak 2013 (160)	55.95 (5.35)	45-65	NR	Excluded those with 'language or geographical barriers'	NR	0%	NR	NR	NR	NR	NR	
Sangthong 2016 (161)	NR (NR)	60-79; median 68 (intervent-ion), median 65 (control)	NR	NR	NR	21%	NR	NR	NR	NR	NR	
Saptharishi 2009 (162)<br><br>(Related article: Subramanian 2011 (163))	22.5 (1.3)	20-25	Described as 'urban service area of JIPMER', but unclear if all participants would have resided in this location.	Appears that all participants spoke Tamil ("... was explained to the participants in the local language, Tamil..."). Not clear if this was an inclusion criterion.	NR	67%	NR	NR	NR	NR	NR	
Schein 2001 (164)	57.14 (8.67)	25-75	Setting was an urban family practice clinic sites,	NR	NR	47%	NR	NR	NR	NR	Excluded those with major psychiatric diseases or	

			but unclear if this also represents participants' residence.								panic disorder.	
Schein 2009 (165)	62.5 (8.46)	40-79	NR	0.3 born in Israel, 0.32 in Europe/ America, 0.2 in Asia, 0.18 in North Africa	NR	62%	NR	Mean 13 (range 6-20) years of education	NR	NR	Excluded those with major psychiatric disorder, or sight or hearing impairment.	
Schneider 1995 (166)<br><br>(Related articles: Alexander 1996 (167), Barnes 1997 (168))	66.8 (7.67)	55-85 years	Described as 'inner city health centre' but unclear if this relates to residence for all participants	All African American	NR	43%	NR	NR	NR	NR	NR	
Schneider 2005 (169)	48.5 (10.1)	NR	NR	All African American	NR	47%	NR	0.62 some college education	0.619 Income <$10,000	0.225 married	Excluded those with psychiatric disorders	
Schneider 2019 (170)<br><br>(Related articles: Castillo Richmond 2000 (171), Schneider 2001 (172))	52.77 (10.26)	20-75	Urban (residents of Los Angeles or surrounding areas)	All African American	NR	35%	NR	Educated to high school level: 0.158 (intervention) 0.095 (control)	Annual income <$20,000: 0.316 (inter-vention) 0.286 (control)	Married: 0.342 (intervention) 0.439 (control)	Excluded those with major psychiatric/ behavioural disorders or alcoholism.	
Schneider 2021 (173)	42.65 (9.59)	21-75	NR	All African American	NR	56%	NR	Education (mean years) in high-normal BP group: 12.1 (SD 3.3)	Annual income in high normal BP group:	NR	Excluded those with major psychiatric	Data reported for individuals with 'high-normal BP' and

								(intervention), 12.8 SD 3.2 (control);<br><br>Education (mean years) in normal BP group: 12.1 (intervention) 12.8 (control) [no SDs reported]	mean $18,650 (inter-vention) $22,400 (control);<br><br>Annual income in normal BP group: mean $23,490 (inter-vention) $20,833 (control)		disorders/ substance abuse disorders.	‘normal BP’. Both groups are equivalent to the pre-hypertension range for this review, and were pooled for analysis.
Seer 1980 (174)	43.24 (9.84)	20-62	NR	NR	NR	56%	NR	NR	NR	NR	NR	
Selvam 2020 (175)	NR (NR)	40-50	Residing in Chennai	NR	NR	100%	NR	NR	NR	NR	NR	
Shapiro 1997 (176)	51.19 (8.47)	NR	NR	0.67 White, 0.15 Black, 0.18 Asian	NR	56%	NR	Education, mean total years: 15.8 SD 3 (intervention), 16.6 SD 3.2 (control)	NR	0.72 married	Excluded those undergoing psychiatric treatment.	
Shetty 2017 (177)	NR (NR)	25-65 years	NR	NR	NR	NR	NR	NR	NR	NR	Excluded those with history of alcoholism.	
Shetty 2022 (178)	49.48 (7.93)	≥18 years	NR	NR	NR	52%	NR	NR	NR	NR	Excluded severe psychiatric co-morbidities.	
Shou 2019 (179)	51.52 (7.58)	18-60	NR	NR	"in service" Unclear if this refers to military	52%	NR	NR	NR	NR	Excluded those who could not provide	

					personnel / civil service, or those who were employed						informed consent due to mental illness; excluded those with physical activity disorder.	
Singh 2022 (180)	49.79 (10.3)	>18 years	population of Sikkim, a hilly state in the Himalayan region	Excluded those who required an interpreter, or who had language difficulties which may preclude participation	Skilled worker* 0.097; clerk, shop owner, farmer 0.139; semi-professional 0.269; professional 0.235; skilled worker* 0.261	50%	NR	Highest stage completed: middle school 0.113; high school 0.139; intermediate 0.181; graduate/post-graduate 0.29; professional 0.277	NR	NR	NR	*two categories reported as 'skilled worker'; unclear whether there is a difference between these.
Southam 1982 (181)<br><br>(Related articles: Agras 1983 (182), Agras 1984 (183))	50.7 (NR)	NR	NR	NR	NR	67%	NR	NR	NR	NR	NR	
Sujatha 2014 (184)	NR (NR)	30-60	NR	NR	NR	46%	NR	No formal education: 0.22 (intervention) 0.21 (control); Complete elementary education: 0.53 (intervention) 0.45 (control); Complete secondary education: 0.22 (intervention)	NR	NR	NR	

								[data missing for control group]				
Sun 2015 (185)	NR (NR)	45-80 years. Aged 45-64: 0.64 (inter-vention) 0.70 (control); Aged >/=65: 0.36 (inter-vention) 0.30 (control)	"Participa-nts also had to be resident in Changshu city of Jiangsu Province or in the Fangshan District of Beijing, within 20 km of the metro-politan area."	"All participants in the Tai Chi intervention group were of Han ethnic origin, the main ethnic group in China". No information on control group, but separately indicates that demographics were not different between groups.	Retired 0.97 intervention, 0.98 control	18%	NR	Years of education <10: 0.45 (intervention) 0.39 (control); 10-12: 0.31 (intervention) 0.46 (control); Bachelor and above: 0.24 (intervention) 0.15 (control)	Income <20,000 RMB: 0.42 (inter-vention) 0.39 (control); 20,000-39,999 RMB: 0.43 (inter-vention) 0.42 (control); >/= 40,000 RMB: 0.15 (inter-vention) 0.19 (control)	Married: 0.94 (intervention) 0.86 (control); widowed 0.03 (intervention) 0.09 (control); Never married 0.03 (intervention) 0.05 (control)	NR	
Supriya 2018 (186)	57.57 (9.1)	30-80	NR	Chinese	NR	35%	NR	NR	NR	NR	Excluded those with dementia or mental disorders, wheelchair users, those with mobility issues not appropriate for yoga exercise.	
Taylor 1977 (187)	48.14 (NR)	NR	NR	NR	NR	74%	NR	NR	NR	NR	NR	
Teng 2007 (188)	81.4 (8)	63-93	NR	NR	NR	27%	NR	NR	NR	NR	All elderly home residents.	

Thanalakshmi 2020 (189)	38.5 (11.48)	18-60	NR	NR	NR	71%	NR	NR	NR	NR	NR	
Thiyagarajan 2015 (190)	43.29 (9.21)	20-60	NR	NR	NR	62%	NR	NR	NR	NR	NR	
Tosi 1992 (191)	47 (NR)	NR	NR	All Caucasian	NR	79%	NR	NR	Described as "predomin-antly middle class"	NR	NR	
Tsai 2003 (192)	51.04 (13.29)	35-65	NR	NR	NR	50%	NR	NR	NR	NR	NR	
Tsai 2007 (193)	43.1 (10.9)	20-55	NR	NR	NR	63%	NR	≤ High school: 0.35 (intervention) 0.22 (control); College: 0.55 (intervention) 0.66 (control); ≥ Graduate school 0.1 (intervention) 0.12 (control); All able to read and write for inclusion	NR	Married: 0.80 (intervention) 0.72 (control)	Excluded those with psychiatric disorders.	
van Montfrans 1990 (194)	41.46 (NR)	24-60	Amsterdam region (urban)	NR	NR	51%	NR	NR	NR	NR	NR	
Venturelli 2015 (195)	67.5 (SE 5.16)	"elderly"	NR	NR	NR	50%	NR	NR	NR	NR	NR	
Wadden 1984 (196)<br><br>(Related article: Wadden 1983 (197))	46.36 (10.41)	21-65	NR	NR	NR	53%	NR	NR	NR	Required to have a spouse or family member (to attend sessions with them).	NR	
Walsh 1977 (198)	NR (NR)	24-69	NR	NR	NR	63%	NR	NR	NR	NR	NR	

Wang 2010 (199)	52.55 (3.81)	45-60	From communi-ties in Guangzhou (urban)	NR	NR	0%	NR	Mean years of education 10.83 SD 3.12 (biofeedback) 10.62 SD 3.68 (breathing intervention)	NR	NR	NR	
Wang 2016 (200)	45.27 (11.26)	18-64	NR	NR	NR	83%	NR	NR	NR	NR	NR	
Webb 2006 (201)	44.3 (7.9)	NR	NR	All African American	All employed	0%	NR	NR	NR	NR	NR	
Wen 2021 (202)	57.49 (9.16)	40-75	NR	NR	NR	60%	NR	Education (mean years: 9.33 SD 2.84 (Wu-style Tai chi) 9.03 SD 2.5 (Tai Chi)	NR	NR	NR	
Wolff 2016 (203)	64.75 (8.42)	34-79	NR	Excluded those requiring interpreter	NR	48%	NR	NR	NR	NR	Excluded those with physical/ mental incapacity to undertake yoga.	
Wright 2021 (204)	72.27 (5.2)	NR	Midwestern urban setting	All African American	NR	18%	NR	Below 12th grade: 0.307 (intervention) 0.153 (non-specific comparator) 0 (usual care). High school graduate/GED 0.153 (intervention) 0.233 (non-specific comparator) 0.166 (usual	Monthly income, US dollars, mean: 1996.3 SD 1478.1 (inter-vention) 1588.3 SD 870.7 (non-specific comparator)1 596.1 SD 426.5 (usual care)	NR	All participants had mild cognitive impairment.	

								care). Some college education: 0.153 (intervention) 0.307 (non-specific comparator) 0.251 (usual care). Associate degree or higher: 0.385 (intervention) 0.307 (non-specific comparator) 0.583 (usual care)				
Wu 2023 (205)	63 (7.54)	NR	NR	NR	NR	32%	NR	NR	NR	NR	NR	
Xiao 2016 (206)	65.6 (7.8)	NR	NR	NR	NR	NR	NR	NR	NR	NR	NR	
Xu 2007 (207)	NR (NR)	19-23	NR	NR	All students	NR	NR	NR	NR	NR	NR	
Yan 2022 (208)	59.87 (6.88)	45-75	NR	NR	NR	35%	NR	NR	NR	NR	NR	Mean age: very minor discrepancy in the abstract (59.89 SD 6.85)
Yau 2022 (209)	66.9 (9.7)	>50 years	NR	Chinese ethnicity. Excluded those who could not speak/ understand Chinese.	Employed full time: 0.13 (diet) 0.2 (diet + relaxation) 0.208 (control); Employed part-time: 0.087 (diet) 0.04 (diet + relaxation) 0 (control); Homemaker: 0.217 (diet)	24%	NR	0.472 completed secondary education overall. Primary education: 0.348 (diet) 0.12 (diet + relaxation) 0.5 (control); Secondary education: 0.435 (diet) 0.56 (diet + relaxation) 0.417 (control); Associate degree:	NR	Married: 0.652 (diet) 0.60 (diet + relaxation) 0.50 (control)	Excluded those with mental disorders.	

					0.24 (diet + relaxation) 0.125 (control); Retired: 0.565 (diet) 0.52 (diet + relaxation) 0.667 (control)			0.043 (diet) 0.12 (diet + relaxation) 0 (control); Bachelor's degree: 0.043 (diet) 0.12 (diet + relaxation) 0 (control); Master's degree: 0.13 (diet) 0.08 (diet + relaxation) 0.042 (control); Doctorate degree: 0 (diet) 0 (diet + relaxation) 0.042 (control)				
Yen 1996 (210)	53.67 (14.63)	Adults, no further information	50 commun-ities in northern Taiwan; mean level of urbanization 5.2 (2.8) (complet-ers) 4.8 (SD 2.6) (dropouts)	0.74 born in Taiwan, 0.26 other	NR	65%	NR	Education >6 years: 0.345 (completers) 0.376 (dropouts)	NR	NR	NR	
Young 1999 (211)	66.7 (5.2)	60-80	Described as "a suburban clinic in the Baltimore area", but this may not apply to the residence for all participants	0.452 Black	NR	21%	NR	NR	NR	NR	NR	

Yuenyongchaiwat 2024 (212)	61.49 (5.17)	40-70	NR	NR	NR	11%	NR	NR	NR	NR	NR	
Yung 2001 (213)	43 (NR)	31-55 years	NR	All Chinese	NR	44%	NR	NR	NR	NR	Excluded those with psychiatric disorders or physical issues that could affect relaxation practices.	
Zanini 2009 (214)	67.1 (9.28)	All >50 years. 0.696 aged ≥ 60 years	NR	NR	NR	42%	NR	0.29 illiterate, 0.53 incomplete primary school education, 0.07 completed primary school, 0.09 high school, 0.02 college education	NR	NR	NR	
Ziv 2013 (215)	57 (9)	22-75	NR	NR	NR	49%	NR	NR	NR	NR	NR	
Zurawski 198 (216)	46.86 (10.55)	18-60 years	NR	NR	NR	32%	NR	NR	NR	NR	NR	

IQR interquartile range; SD standard deviation; SE standard error; NR not reported

References

1. Freedland KE, King AC, Ambrosius WT, Mayo-Wilson E, Mohr DC, Czajkowski SM, et al. The selection of comparators for randomized controlled trials of health-related behavioral interventions: recommendations of an NIH expert panel. Journal of Clinical Epidemiology. 2019;110:74-81.
2. Gold SM, Enck P, Hasselmann H, Friede T, Hegerl U, Mohr DC, et al. Control conditions for randomised trials of behavioural interventions in psychiatry: a decision framework. Lancet Psychiatry. 2017;4(9):725-32.
3. Whelton PK. Evolution of Blood Pressure Clinical Practice Guidelines: A Personal Perspective. Canadian Journal of Cardiology. 2019;35(5):570-81.
4. Turner KM, Huntley A, Yardley T, Dawson S, Dawson S. Defining usual care comparators when designing pragmatic trials of complex health interventions: a methodology review. Trials. 2024;25(1):117.
5. Higgins J, Li, T., Deeks, JJ (editors). Chapter 6: Choosing effect measures and computing estimates of effect. In: Higgins J, Thomas J, Chandler J, Cumpston M, Li T, Page M, et al., editors. Cochrane Handbook for Systematic Reviews of Interventions 64 (updated August 2023). Chapter 6: Choosing effect measures and computing estimates of effect: Cochrane; 2023.
6. Daly C, Welton NJ, Dias S, Anwer S, Ades AE. NICE Guidelines Technical Support Unit: Meta-analysis of continuous outcomes. Guideline Methodology Document 2 2021 [Available from: https://www.bristol.ac.uk/media-library/sites/social-community-medicine/documents/mpes/gmd-2-continuous-jan2021.pdf.
7. Balk E, Earley A, Patel K, Trikalinos T, Dahabreh I. Empirical Assessment of Within-Arm Correlation Imputation in Trials of Continuous Outcomes: Rockville (MD): Agency for Healthcare Research and Quality (US); 2012.
8. Higgins J, Eldridge, S., Li, T. (editors). Chapter 23: Including variants on randomized trials. In: Higgins J, Thomas J, Chandler J, Cumpston M, Li T, Page M, et al., editors. Cochrane Handbook for Systematic Reviews of Interventions 64 (updated August 2023). Chapter 23: Including variants on randomized trials: Cochrane; 2023.
9. Staplin N, de la Sierra A, Ruilope LM, Emberson JR, Vinyoles E, Gorostidi M, et al. Relationship between clinic and ambulatory blood pressure and mortality: an observational cohort study in 59124 patients. The Lancet. 2023;401(10393):2041-50.
10. Green BB, Anderson ML, Cook AJ, Ehrlich K, Hall YN, Hsu C, et al. Clinic, Home, and Kiosk Blood Pressure Measurements for Diagnosing Hypertension: a Randomized Diagnostic Study. Journal of General Internal Medicine. 2022;37(12):2948-56.
11. Stergiou GS, Bliziotis IA. Home Blood Pressure Monitoring in the Diagnosis and Treatment of Hypertension: A Systematic Review. American Journal of Hypertension. 2011;24(2):123-34.
12. Carpenter B, Gelman A, Hoffman MD, Lee D, Goodrich B, Betancourt M, et al. Stan: A Probabilistic Programming Language. Journal of Statistical Software. 2017;76(1):1 - 32.
13. Spiegelhalter DJ, Best NG, Carlin BP, Van Der Linde A. Bayesian measures of model complexity and fit. Journal of the Royal Statistical Society: Series B (Statistical Methodology). 2002;64(4):583-639.
14. Dias S, Welton NJ, Sutton AJ, Ades AE. NICE DSU Technical Support Document 2: A Generalised Linear Modelling Framework for Pairwise and Network Meta-Analysis of Randomised Controlled Trials2014. Available from: https://www.ncbi.nlm.nih.gov/books/NBK310366/pdf/Bookshelf_NBK310366.pdf.
15. Achmon J, Granek M, Golomb M, Hart J. Behavioral treatment of essential hypertension: a comparison between cognitive therapy and biofeedback of heart rate. Psychosomatic Medicine. 1989;51(2):152-64.

16. Adams ZW, Sieverdes JC, Brunner-Jackson B, Mueller M, Ch, ler J, et al. Meditation smartphone application effects on prehypertensive adults' blood pressure: Dose-response feasibility trial. Health Psychology. 2018;37(9):850-60.
17. Adsett CA, Bellissimo A, Mitchell A, Wilczynski N, Haynes RB. Behavioral and physiological effects of a beta blocker and relaxation therapy on mild hypertensives. Psychosomatic Medicine. 1989;51(5):523-36.
18. Agras WS, Taylor CB, Kraemer HC, Southam MA, Schneider JA. Relaxation training for essential hypertension at the worksite: II. The poorly controlled hypertensive. Psychosomatic Medicine. 1987;49(3):264-73.
19. Ahmadpanah M, Paghale SJ, Bakhtyari A, Kaikhavani S, Aghaei E, Nazaribadie M, et al. Effects of psychotherapy in combination with pharmacotherapy, when compared to pharmacotherapy only on blood pressure, depression, and anxiety in female patients with hypertension. Journal of Health Psychology. 2016;21(7):1216-27.
20. Aivazyan TA, Zaitsev VP, Salenko BB, Yurenev AP, Patrusheva IF. Efficacy of relaxation techniques in hypertensive patients. Health Psychology. 1988;7:193-200.
21. Aivazyan TA, Zaitsev VP, Yurenev AP. Autogenic training in the treatment and secondary prevention of essential hypertension: five-year follow-up. Health Psychology. 1988;7:201-8.
22. Altena MR, Kleefstra N, Logtenberg SJ, Groenier KH, Houweling ST, Bilo HJ. Effect of device-guided breathing exercises on blood pressure in patients with hypertension: a randomized controlled trial. Blood Pressure. 2009;18(5):273-9.
23. Amigo I, Gonzalez A, Herrera J. Comparison of physical exercise and muscle relaxation training in the treatment of mild essential hypertension. Stress Medicine. 1997;13(1):59-65.
24. Anderson DE, McNeely JD, Windham BG. Regular slow-breathing exercise effects on blood pressure and breathing patterns at rest. Journal of Human Hypertension. 2010;24(12):807-13.
25. Anjana K, Archana R, Mukkadan JK. Effect of om chanting and yoga nidra on blood pressure and lipid profile in hypertension - A randomized controlled trial. Journal of Ayurveda and Integrative Medicine. 2022;13(4):100657.
26. Ankolekar VHR, Govardhan G; Sanju, Chidananda SV; Mamatha, H;. Role of yoga intervention on quality of life and prehypertension. Indian Journal of Traditional Knowledge. 2019;18(2):351-55.
27. Arslan G, Ceyhan O, Mollaoglu M. The influence of foot and back massage on blood pressure and sleep quality in females with essential hypertension: a randomized controlled study. Journal of Human Hypertension. 2021;35(7):627-37.
28. Babak A, Motamedi N, Mousavi SZ, Ghasemi Darestani N. Effects of Mindfulness-Based Stress Reduction on Blood Pressure, Mental Health, and Quality of Life in Hypertensive Adult Women: A Randomized Clinical Trial Study. The Journal of Tehran Heart Center. 2022;17(3):127-33.
29. Balasubramanian S, Purvesh JM, Varghese John P, Vijay Pratap S. Slow breathing training on cardio-respiratory control and exercise capacity in persons with essential hypertension -- a randomized controlled trial. Indian Journal of Physiotherapy & Occupational Therapy. 2012;6:17-21.
30. Batey DM, Kaufmann PG, Raczynski JM, Hollis JF, Murphy JK, Rosner B, et al. Stress management intervention for primary prevention of hypertension: detailed results from Phase I of Trials of Hypertension Prevention (TOHP-I). Annals of Epidemiology. 2000;10(1):45-58.
31. Whelton PK, Hebert PR, Cutler J, Applegate WB, Eberlein KA, Klag MJ, et al. Baseline characteristics of participants in phase I of the Trials of Hypertension Prevention. Annals of Epidemiology. 1992;2(3):295-310.
32. The Trials of Hypertension Prevention Collaborative Research Group. The effects of nonpharmacologic interventions on blood pressure of persons with high normal levels. Results of the Trials of Hypertension Prevention, Phase I. JAMA. 1992;267(9):1213-20.

33. Whelton PK, Kumanyika SK, Cook NR, Cutler JA, Borhani NO, Hennekens CH, et al. Efficacy of nonpharmacologic interventions in adults with high-normal blood pressure: results from phase 1 of the Trials of Hypertension Prevention. Trials of Hypertension Prevention Collaborative Research Group. American Journal of Clinical Nutrition. 1997;65(2):652S-60S.
34. Bekiroglu T, Ovayolu N, Ergun Y, Ekerbicer HC. Effect of Turkish classical music on blood pressure: a randomized controlled trial in hypertensive elderly patients. Complementary Therapies in Medicine. 2013;21(3):147-54.
35. Bennett P, Wallace L, Carroll D, Smith N. Treating Type A behaviours and mild hypertension in middle-aged men. Journal of Psychosomatic Research. 1991;35(2):209-23.
36. Blanchard EB, Miller ST, Abel GG, Haynes MR, Wicker R. Evaluation of biofeedback in the treatment of borderline essential hypertension. Journal of Applied Behavior Analysis. 1979;12(1):99-109.
37. Blanchard EB, McCoy GC, Andrasik F, Acerra M, Pallmeyer TP, Gerardi R, et al. Preliminary results from a controlled evaluation of thermal biofeedback as a treatment for essential hypertension. Biofeedback & Self Regulation. 1984;9(4):471-95.
38. Blanchard EB, McCoy GC, Musso A, Gerardi MA, Pallmeyer TP, Gerardi RJ, et al. A controlled comparison of thermal biofeedback and relaxation training in the treatment of essential hypertension: I. Short-term and long-term outcome. Behavior Therapy. 1986;17(5):563-79.
39. Blanchard EB, Khramelashvili VV, McCoy GC, Aivazyan TA, McCaffrey RJ, Salenko BB, et al. The USA-USSR collaborative cross-cultural comparison of autogenic training and thermal biofeedback in the treatment of mild hypertension. Health Psychology. 1988;7:175-92.
40. Blanchard EB, Eisele G, Gordon MA, Cornish PJ, Wittrock DA, Gilmore L, et al. Thermal biofeedback as an effective substitute for sympatholytic medication in moderate hypertension: a failure to replicate. Biofeedback & Self Regulation. 1993;18(4):237-53.
41. Blanchard EB, Eisele G, Vollmer A, Payne A, Gordon M, Cornish P, et al. Controlled evaluation of thermal biofeedback in treatment of elevated blood pressure in unmedicated mild hypertension. Biofeedback & Self Regulation. 1996;21(2):167-90.
42. Blom K, Baker B, How M, Dai M, Irvine J, Abbey S, et al. Hypertension analysis of stress reduction using mindfulness meditation and yoga: results from the HARMONY randomized controlled trial. American Journal of Hypertension. 2014;27(1):122-9.
43. Bosley F, Allen TW. Stress management training for hypertensives: cognitive and physiological effects. Journal of Behavioral Medicine. 1989;12(1):77-89.
44. Brauer AP, Horlick L, Nelson E, Farquhar JW, Agras WS. Relaxation therapy for essential hypertension: a Veterans Administration Outpatient study. Journal of Behavioral Medicine. 1979;2(1):21-9.
45. Canino E, Cardona R, Monsalve P, Perez Acuna F, Lopez B, Fragachan F. A behavioral treatment program as a therapy in the control of primary hypertension. Acta Cientifica Venezolana. 1994;45(1):23-30.
46. Chan AWK, Chair SY, Lee DTF, Leung DYP, Sit JWH, Cheng HY, et al. Tai Chi exercise is more effective than brisk walking in reducing cardiovascular disease risk factors among adults with hypertension: A randomised controlled trial. International Journal of Nursing Studies. 2018;88:44-52.
47. Chandler J, Sox L, Diaz V, Kellam K, Neely A, Nemeth L, et al. Impact of 12-Month Smartphone Breathing Meditation Program upon Systolic Blood Pressure among Non-Medicated Stage 1 Hypertensive Adults. International Journal of Environmental Research & Public Health [Electronic Resource]. 2020;17(6):17.
48. Charlesworth EA, Williams BJ, Baer PE. Stress management at the worksite for hypertension: compliance, cost-benefit, health care and hypertension-related variables. Psychosomatic Medicine. 1984;46(5):387-97.

49. Chen D. Effect of Health Qigong Mawangdui Daoyinshu on Blood Pressure of Individuals with Essential Hypertension. Journal of the American Geriatrics Society. 2016;64(7):1513-5.
50. Chen S, Sun P, Wang S, Lin G, Wang T. Effects of heart rate variability biofeedback on cardiovascular responses and autonomic sympathovagal modulation following stressor tasks in prehypertensives. Journal of Human Hypertension. 2016;30(2):105-11.
51. Chesney MA, Black GW, Swan GE, Ward MM. Relaxation training for essential hypertension at the worksite: I. The untreated mild hypertensive. Psychosomatic Medicine. 1987;49(3):250-63.
52. Cheung BM, Lo JL, Fong DY, Chan MY, Wong SH, Wong VC, et al. Randomised controlled trial of qigong in the treatment of mild essential hypertension. Journal of Human Hypertension. 2005;19(9):697-704.
53. Clemow LP, Pickering TG, Davidson KW, Schwartz JE, Williams VP, Shaffer JA, et al. Stress management in the workplace for employees with hypertension: a randomized controlled trial. Translational Behavioral Medicine. 2018;8(5):761-70.
54. Cohen J, Sedlacek K. Attention and autonomic self-regulation. Psychosomatic Medicine. 1983;45(3):243-57.
55. Cohen DL, Bloedon LT, Rothman RL, Farrar JT, Galantino ML, Volger S, et al. Iyengar Yoga versus Enhanced Usual Care on Blood Pressure in Patients with Prehypertension to Stage I Hypertension: a Randomized Controlled Trial. Evidence-Based Complementary & Alternative Medicine: eCAM. 2011;2011:546428.
56. Cohen DL, Boudhar S, Bowler A, Townsend RR. Blood Pressure Effects of Yoga, Alone or in Combination With Lifestyle Measures: Results of the Lifestyle Modification and Blood Pressure Study (LIMBS). Journal of Clinical Hypertension. 2016;18(8):809-16.
57. Cottier C, Shapiro K, Julius S. Treatment of mild hypertension with progressive muscle relaxation. Predictive value of indexes of sympathetic tone. Archives of Internal Medicine. 1984;144(10):1954-8.
58. Cramer H, Sellin C, Schumann D, Dobos G. Yoga in Arterial Hypertension. Deutsches Arzteblatt International. 2018;115(50):833-9.
59. Guaman MIG, Guerrero OEA, Betancourt MIF, Martinez RM. Evaluation of the effect of yoga postures on blood press. Journal of pharmaceutical negative results. 2022;13:3034-41.
60. de Barros S, da Silva GV, de Gusmao JL, de Araujo TG, de Souza DR, Cardoso CG, Jr., et al. Effects of long term device-guided slow breathing on sympathetic nervous activity in hypertensive patients: a randomized open-label clinical trial. Blood Pressure. 2017;26(6):359-65.
61. de Fatima Rosas Marchiori M, Kozasa EH, Mir, a RD, Monezi Andrade AL, Perrotti TC, et al. Decrease in blood pressure and improved psychological aspects through meditation training in hypertensive older adults: A randomized control study. Geriatrics & Gerontology International. 2015;15(10):1158-64.
62. Dhungana RR, Pedisic Z, Joshi S, Khanal MK, Kalauni OP, Shakya A, et al. Effects of a health worker-led 3-month yoga intervention on blood pressure of hypertensive patients: a randomised controlled multicentre trial in the primary care setting. BMC Public Health. 2021;21(1):550.
63. Drazen M, Nevid JS, Pace N, O'Brien RM. Worksite based behavioral treatment of mild hypertension. Journal of Occupational Medicine. 1982;24(7):511-4.
64. Dusek JA, Hibberd PL, Buczynski B, Chang BH, Dusek KC, Johnston JM, et al. Stress management versus lifestyle modification on systolic hypertension and medication elimination: a randomized trial. Journal of Alternative & Complementary Medicine. 2008;14(2):129-38.
65. Elavally S, Ramamurthy MT, Subash J, Meleveedu R, Venkatasalu MR. Effect of nurse-led home-based biofeedback intervention on the blood pressure levels among patients with hypertension: Pretest-posttest study. Journal of Family Medicine & Primary Care. 2020;9(9):4833-40.

66. Elliot WJ, Izzo JL, Jr., White WB, Rosing DR, Snyder CS, Alter A, et al. Graded blood pressure reduction in hypertensive outpatients associated with use of a device to assist with slow breathing. Journal of Clinical Hypertension. 2004;6(10):553-9; quiz 60-1.
67. Fetter C, Marques JR, de Souza LA, Dartora DR, Eibel B, Boll LFC, et al. Additional Improvement of Respiratory Technique on Vascular Function in Hypertensive Postmenopausal Women Following Yoga or Stretching Video Classes: The YOGINI Study. Frontiers in Physiology. 2020;11:898.
68. Frankel BL, Patel DJ, Horwitz D, Friedewald WT, Gaarder KR. Treatment of hypertension with biofeedback and relaxation techniques. Psychosomatic Medicine. 1978;40(4):276-93.
69. Friedman HT, Harvey A;. The use of hypnosis and biofeedback procedures for essential hypertension. International Journal of Clinical and Experimental Hypnosis. 1977;25(4):335-47.
70. Friedman H, Taub HA. A six-month follow-up of the use of hypnosis and biofeedback procedures in essential hypertension. American Journal of Clinical Hypnosis. 1978;20(3):184-8.
71. Garcia-Vera MP, Labrador FJ, Sanz J. Stress-management training for essential hypertension: a controlled study. Applied Psychophysiology & Biofeedback. 1997;22(4):261-83.
72. Gay MC. Effectiveness of hypnosis in reducing mild essential hypertension: a one-year follow-up. International Journal of Clinical & Experimental Hypnosis. 2007;55(1):67-83.
73. Givi M, Sadeghi M, Garakyaraghi M, Eshghinezhad A, Moeini M, Ghasempour Z. Long-term effect of massage therapy on blood pressure in prehypertensive women. Journal of Education & Health Promotion. 2018;7:54.
74. Grossman E, Grossman A, Schein MH, Zimlichman R, Gavish B. Breathing-control lowers blood pressure. Journal of Human Hypertension. 2001;15(4):263-9.
75. Hafner RJ. Psychological treatment of essential hypertension: a controlled comparison of meditation and meditation plus biofeedback. Biofeedback & Self Regulation. 1982;7(3):305-16.
76. Hager JL, Surwit RS. Hypertension self-control with a portable feedback unit or meditation-relaxation. Biofeedback & Self Regulation. 1978;3(3):269-76.
77. Haghighat A, Haghighat R. The role of physical psychotherapy on quality of life in patients with hypertension with alexithymia. European Journal of Molecular and Clinical Medicine. 2021;8(2):2154-62.
78. Hagins M, Rundle A, Consedine NS, Khalsa SB. A randomized controlled trial comparing the effects of yoga with an active control on ambulatory blood pressure in individuals with prehypertension and stage 1 hypertension. Journal of Clinical Hypertension. 2014;16(1):54-62.
79. Hasandokht T, Farajzadegan Z, Siadat ZD, Paknahad Z, Rajati F. Lifestyle interventions for hypertension treatment among Iranian women in primary health-care settings: Results of a randomized controlled trial. Journal of Research in Medical Sciences. 2015;20(1):54-61.
80. Hatch JP, Klatt KD, Supik JD, Rios N, Fisher JG, Bauer RL, et al. Combined behavioral and pharmacological treatment of essential hypertension. Biofeedback & Self Regulation. 1985;10(2):119-38.
81. Henderson RJ, Hart MG, Lal SK, Hunyor SN. The effect of home training with direct blood pressure biofeedback of hypertensives: a placebo-controlled study. Journal of Hypertension. 1998;16(6):771-8.
82. Hernandez-Reif M, Field T, Krasnegor J, Theakston H, Hossain Z, Burman I. High blood pressure and associated symptoms were reduced by massage therapy. Journal of Bodywork and Movement Therapies. 2000;4(1):31-8.
83. Hoelscher TJ, Lichstein KL, Rosenthal TL. Home relaxation practice in hypertension treatment: objective assessment and compliance induction. Journal of Consulting & Clinical Psychology. 1986;54(2):217-21.
84. Hoelscher TJ, Lichstein KL, Fischer S, Hegarty TB. Relaxation treatment of hypertension: Do home relaxation tapes enhance treatment outcome? Behavior Therapy. 1987;18(1):33-7.

85. Howorka K, Pumprla J, Tamm J, Schabmann A, Klomfar S, Kostineak E, et al. Effects of guided breathing on blood pressure and heart rate variability in hypertensive diabetic patients. Autonomic Neuroscience-Basic & Clinical. 2013;179(1):131-7.
86. Hughes JW, Fresco DM, Myerscough R, van Dulmen MH, Carlson LE, Josephson R. Randomized controlled trial of mindfulness-based stress reduction for prehypertension. Psychosomatic Medicine. 2013;75(8):721-8.
87. Huijuan Y, Zhexin Y, Jing C, Pan L, Min X, Xingting D, et al. Observation on the clinical effect of tcm tone-breathing exercise therapy on hyperactivity of liver-yang type of hypertension. Acta Medica Mediterranea. 2021;37(6):3583-8.
88. Im-Oun S, Kotruchin P, Thinsug P, Mitsungnern T, Techa-Atik P, Pongchaiyakul C. Effect of Thai instrumental folk music on blood pressure: A randomized controlled trial in stage-2 hypertensive patients. Complementary Therapies in Medicine. 2018;39:43-8.
89. Irvine MJ, Johnston DW, Jenner DA, Marie GV. Relaxation and stress management in the treatment of essential hypertension. Journal of Psychosomatic Research. 1986;30(4):437-50.
90. Irvine MJ, Logan AG. Relaxation behavior therapy as sole treatment for mild hypertension. Psychosomatic Medicine. 1991;53(6):587-97.
91. Ismail AMA, Saif H, Taha MM. Effect of alternate nostril breathing exercise on autonomic functions, ocular hypertension, and quality of life in elderly with systemic hypertension and high-tension primary open-angle glaucoma. Geriatric Nursing. 2023;52:91-7.
92. Jacob RG, Fortmann SP, Kraemer HC. Combining behavioral treatments to reduce blood pressure. A controlled outcome study. Behavior Modification. 1985;9(1):32-54.
93. Jacob RG, Shapiro AP, O'Hara P, Portser S, Kruger A, Gatsonis C, et al. Relaxation therapy for hypertension: setting-specific effects. Psychosomatic Medicine. 1992;54(1):87-101.
94. Johnston DW, Gold A, Kentish J, Smith D, Vallance P, Shah D, et al. Effect of stress management on blood pressure in mild primary hypertension. BMJ. 1993;306(6883):963-6.
95. Jones CU, Sangthong B, Pachirat O. An inspiratory load enhances the antihypertensive effects of home-based training with slow deep breathing: a randomised trial. Journal of Physiotherapy. 2010;56(3):179-86.
96. Jorgensen RS, Houston BK, Zurawski RM. Anxiety management training in the treatment of essential hypertension. Behaviour Research & Therapy. 1981;19(6):467-74.
97. Kalmatayeva Z, Zholamanova A. Cost-effectiveness analysis of psychotherapy in treatment of essential hypertension in primary care. Archives of Psychiatry and Psychotherapy. 2014;16(4):57-64.
98. Katsarou AL, Vryonis MM, Protogerou AD, Alexopoulos EC, Achimastos A, Papadogiannis D, et al. Stress management and dietary counseling in hypertensive patients: a pilot study of additional effect. Primary Health Care Research & Development. 2014;15(1):38-45.
99. Kohn JN, Lobo JD, Troyer EA, Ang G, Wilson KL, Walker AL, et al. Tai Chi versus health education as a frailty intervention for community-dwelling older adults with hypertension. Aging Clinical & Experimental Research. 2023;17:17.
100. Kohn JN, Lobo JD, Troyer EA, Wilson KL, Ang G, Walker AL, et al. Tai chi or health education for older adults with hypertension: effects on mental health and psychological resilience to COVID-19. Aging & Mental Health. 2023;27(3):496-504.
101. Kow FP, Adlina B, Sivasangari S, Punithavathi N, Ng KK, Ang AH, et al. The impact of music guided deep breathing exercise on blood pressure control - A participant blinded randomised controlled study. Medical Journal of Malaysia. 2018;73(4):233-8.
102. Kretzer K, Evelo AJ, Durham RL. Lessons learned from a study of a complementary therapy for self-managing hypertension and stress in women. Holistic Nursing Practice. 2013;27(6):336-43.
103. Kumar S, Lathif F, Raghavan V. Effects of Mindfulness-Based Stress Reduction on Blood Pressure (MBSR) Among Patients with Type-2 Diabetes - A Randomised Pilot Study. Nursing Journal of India. 2017;108(2):61-3.

104. Kunikullaya KU, Goturu J, Muradi V, Hukkeri PA, Kunnavil R, Doreswamy V, et al. Music versus lifestyle on the autonomic nervous system of prehypertensives and hypertensives--a randomized control trial. Complementary Therapies in Medicine. 2015;23(5):733-40.
105. Kunikullaya KU, Goturu J, Muradi V, Hukkeri PA, Kunnavil R, Doreswamy V, et al. Combination of music with lifestyle modification versus lifestyle modification alone on blood pressure reduction - A randomized controlled trial. Complementary Therapies in Clinical Practice. 2016;23:102-9.
106. LaGrone R, Jeffrey TB, Ferguson CL. Effects of education and relaxation training with essential hypertension patients. Journal of Clinical Psychology. 1988;44(2):271-6.
107. Landman GW, Drion I, van Hateren KJ, van Dijk PR, Logtenberg SJ, Lambert J, et al. Device-guided breathing as treatment for hypertension in type 2 diabetes mellitus: a randomized, double-blind, sham-controlled trial. JAMA Internal Medicine. 2013;173(14):1346-50.
108. Latha, Kaliappan KV. Yoga, Pranayama, Thermal Biofeedback techniques in the management of stress and high blood pressure. Journal of Indian Psychology. 1991;9:36-46.
109. Lee DD, DeQuattro V, Allen J, Kimura S, Aleman E, Konugres G, et al. Behavioral vs beta-blocker therapy in patients with primary hypertension: effects on blood pressure, left ventricular function and mass, and the pressor surge of social stress anger. American Heart Journal. 1988;116(2):637-44.
110. Lee MS, Lee MS, Kim HJ, Moon SR. Qigong reduced blood pressure and catecholamine levels of patients with essential hypertension. International Journal of Neuroscience. 2003;113(12):1691-701.
111. Li X, Chang P, Wu M, Jiang Y, Gao Y, Chen H, et al. Effect of Tai Chi vs Aerobic Exercise on Blood Pressure in Patients With Prehypertension: A Randomized Clinical Trial. JAMA Network Open. 2024;7(2):e2354937.
112. Lin G, Xiang Q, Fu X, Wang S, Wang S, Chen S, et al. Heart rate variability biofeedback decreases blood pressure in prehypertensive subjects by improving autonomic function and baroreflex. Journal of Alternative & Complementary Medicine. 2012;18(2):143-52.
113. Lin B, Jin Q, Liu C, Zhao W, Ji R. Effect and mechanism of tai chi on blood pressure of patients with essential hypertension: a randomized controlled study. Journal of Sports Medicine & Physical Fitness. 2022;62(9):1272-7.
114. Linden W, Lenz JW, Con AH. Individualized stress management for primary hypertension: a randomized trial. Archives of Internal Medicine. 2001;161(8):1071-80.
115. Logtenberg SJ, Kleefstra N, Houweling ST, Groenier KH, Bilo HJ. Effect of device-guided breathing exercises on blood pressure in hypertensive patients with type 2 diabetes mellitus: a randomized controlled trial. Journal of Hypertension. 2007;25(1):241-6.
116. Lokesh R, Sudhan SG. A Comparitive Study on the Effect of Macqueen's Progressive Resisted Exercise and Breathing Control Training on Hypertensives. Indian Journal of Physiotherapy & Occupational Therapy. 2017;11(3):132-5.
117. Loucks EB, Schuman-Olivier Z, Saadeh FB, Scarpaci MM, Nardi WR, Proulx JA, et al. Effect of Adapted Mindfulness Training in Participants With Elevated Office Blood Pressure: The MB-BP Study: A Randomized Clinical Trial. Journal of the American Heart Association. 2023;12(11):e028712.
118. Polcari JJ, Cali RJ, Nephew BC, Lu S, Rashkovskii M, Wu J, et al. Effects of the Mindfulness-Based Blood Pressure Reduction (MB-BP) program on depression and neural structural connectivity. Journal of Affective Disorders. 2022;311:31-9.
119. Ma C, Zhou W, Tang Q, Huang S. The impact of group-based Tai chi on health-status outcomes among community-dwelling older adults with hypertension. Heart & Lung. 2018;47(4):337-44.

120. Manikonda JP, Stork S, Togel S, Lobmuller A, Grunberg I, Bedel S, et al. Contemplative meditation reduces ambulatory blood pressure and stress-induced hypertension: a randomized pilot trial. Journal of Human Hypertension. 2008;22(2):138-40.
121. McCaffrey R, Ruknui P, Hatthakit U, Kasetsomboon P. The effects of yoga on hypertensive persons in Thailand. Holistic Nursing Practice. 2005;19(4):173-80.
122. McCraty R, Atkinson M, Tomasino D. Impact of a workplace stress reduction program on blood pressure and emotional health in hypertensive employees. Journal of Alternative & Complementary Medicine. 2003;9(3):355-69.
123. McGrady AV, Yonker R, Tan SY, Fine TH, Woerner M. The effect of biofeedback-assisted relaxation training on blood pressure and selected biochemical parameters in patients with essential hypertension. Biofeedback & Self Regulation. 1981;6(3):343-53.
124. McGrady A. Effects of group relaxation training and thermal biofeedback on blood pressure and related physiological and psychological variables in essential hypertension. Biofeedback & Self Regulation. 1994;19(1):51-66.
125. Mir IA, Chowdhury M, Islam RM, Ling GY, Chowdhury A, Hasan ZM, et al. Relaxing music reduces blood pressure and heart rate among pre-hypertensive young adults: A randomized control trial. Journal of Clinical Hypertension. 2021;23(2):317-22.
126. Misra S, Smith J, Wareg N, Hodges K, Gandhi M, McElroy JA. Take a deep breath: A randomized control trial of Pranayama breathing on uncontrolled hypertension. Advances in Integrative Medicine. 2019;6(2):66-72.
127. Modesti PA, Ferrari A, Bazzini C, Costanzo G, Simonetti I, Taddei S, et al. Psychological predictors of the antihypertensive effects of music-guided slow breathing. Journal of Hypertension. 2010;28(5):1097-103.
128. Modesti PA, Ferrari A, Bazzini C, Boddi M. Time sequence of autonomic changes induced by daily slow-breathing sessions. Clinical Autonomic Research. 2015;25(2):95-104.
129. Mohammed Elsheikh SE, Mohamed Elnahas NG, Mohammed Lotfy Mohammed Soliman AW, Ali Ismail AM. Effect of Bhramari versus Sheetali pranayama on quality of life in hypertensive patients. Advances in Rehabilitation. 2023;37(2):1-8.
130. Mohebbi Z, Moghadasi M, Homayouni K, Nikou MH. The effect of back massage on blood pressure in the patients with primary hypertension in 2012-2013: a randomized clinical trial. International Journal of Community Based Nursing & Midwifery. 2014;2(4):251-8.
131. Moghadasi M, Mohebbi Z, Homayouni K, Nikoo MH. Cardiorespiratory effect of Swedish back massage in hypertensive patients: a randomized clinical trial. Arterial Hypertension (Poland). 2021;25(4):159-63.
132. Momeni J, Omidi A, Raygan F, Akbari H. The effects of mindfulness-based stress reduction on cardiac patients' blood pressure, perceived stress, and anger: a single-blind randomized controlled trial. Journal of the American Society of Hypertension. 2016;10(10):763-71.
133. Mourya M, Mahajan AS, Singh NP, Jain AK. Effect of slow- and fast-breathing exercises on autonomic functions in patients with essential hypertension. Journal of Alternative & Complementary Medicine. 2009;15(7):711-7.
134. Murugesan R, Govindarajulu N, Bera TK. Effect of selected yogic practices on the management of hypertension. Indian Journal of Physiology & Pharmacology. 2000;44(2):207-10.
135. Nakao M, Nomura S, Shimosawa T, Yoshiuchi K, Kumano H, Kuboki T, et al. Clinical effects of blood pressure biofeedback treatment on hypertension by auto-shaping. Psychosomatic Medicine. 1997;59(3):331-8.
136. Nakao M, Nomura S, Shimosawa T, Fujita T, Kuboki T. Blood pressure biofeedback treatment of white-coat hypertension. Journal of Psychosomatic Research. 2000;48(2):161-9.
137. Nejati S, Zahiroddin A, Afrookhteh G, Rahmani S, Hoveida S. Effect of Group Mindfulness-Based Stress-Reduction Program and Conscious Yoga on Lifestyle, Coping

Strategies, and Systolic and Diastolic Blood Pressures in Patients with Hypertension. The Journal of Tehran Heart Center. 2015;10(3):140-8.
138. Nolan RP, Floras JS, Harvey PJ, Kamath MV, Picton PE, Chessex C, et al. Behavioral neurocardiac training in hypertension: a randomized, controlled trial. Hypertension. 2010;55(4):1033-9.
139. Olsson EM, El Alaoui S, Carlberg B, Carlbring P, Ghaderi A. Internet-based biofeedback-assisted relaxation training in the treatment of hypertension: a pilot study. Applied Psychophysiology & Biofeedback. 2010;35(2):163-70.
140. Palomba D, Ghisi M, Scozzari S, Sarlo M, Bonso E, Dorigatti F, et al. Biofeedback-assisted cardiovascular control in hypertensives exposed to emotional stress: a pilot study. Applied Psychophysiology & Biofeedback. 2011;36(3):185-92.
141. Pandey A, Pandey A, Pandey AS, Bonsignore A, Auclair A, Poirier P. Impact of Yoga on Global Cardiovascular Risk as an Add-On to a Regular Exercise Regimen in Patients With Hypertension. Canadian Journal of Cardiology. 2023;39(1):57-62.
142. Pandic S, Ekman I, Nord L, Kjellgren KI. Device-guided breathing exercises in the treatment of hypertension - perceptions and effects. CVD prevention and control. 2008;3(3):163-9.
143. Park JE, Hong S, Lee M, Park T, Kang K, Jung H, et al. Randomized, controlled trial of qigong for treatment of prehypertension and mild essential hypertension. Alternative Therapies in Health & Medicine. 2014;20(4):21-30.
144. Park JE, Kim JE, Jung S, Kim A, Park H, Hong S. The Effect of Dongeui Qigong for Prehypertension and Mild Essential Hypertension. Evidence-Based Complementary & Alternative Medicine: eCAM. 2017;2017:4274538.
145. Patel C, North WR. Randomised controlled trial of yoga and bio-feedback in management of hypertension. The Lancet. 1975;2(7925):93-5.
146. Patel C, Marmot M. Can general practitioners use training in relaxation and management of stress to reduce mild hypertension? British Medical Journal Clinical Research Ed. 1988;296(6614):21-4.
147. Pathan FKM, Pandian JS, Shaikh AI, Ahsan M, Nuhmani S, Iqbal A, et al. Effect of slow breathing exercise and progressive muscle relaxation technique in the individual with essential hypertension: A randomized controlled trial. Medicine (Baltimore). 2023;102(47):e35792.
148. Patil SG, Dhanakshirur GB, Aithala MR, Naregal G, Das KK. Effect of yoga on oxidative stress in elderly with grade-I hypertension: a randomized controlled study. Journal of Clinical and Diagnostic Research JCDR. 2014;8(7):BC04-7.
149. Perez MI, Linden W, Perry T, Jr., Puil LJ, Wright JM. Failure of psychological interventions to lower blood pressure: a randomized controlled trial. Open Medicine : A Peer-reviewed, Independent, Open-access Journal. 2009;3(2):e92-e100.
150. Plaugher G, Long CR, Alcantara J, Silveus AD, Wood H, Lotun K, et al. Practice-based randomized controlled-comparison clinical trial of chiropractic adjustments and brief massage treatment at sites of subluxation in subjects with essential hypertension: pilot study. Journal of Manipulative & Physiological Therapeutics. 2002;25(4):221-39.
151. Ponte Marquez PH, Feliu-Soler A, Sole-Villa MJ, Matas-Pericas L, Filella-Agullo D, Ruiz-Herrerias M, et al. Benefits of mindfulness meditation in reducing blood pressure and stress in patients with arterial hypertension. Journal of Human Hypertension. 2019;33(3):237-47.
152. Concepcion IP, Gabriela SLA, Valdiviezo WV. Benefits of meditation in reducing blood pressure and stress in patients with high blood pressure. NeuroQuantology. 2022;20(13):2152-8.
153. Pukdeesamai R, Srihatrai P, N I. Effects of a Mindfulness Meditation on Blood Pressure in Prehypertension Patients: A Randomized Controlled Trial. Journal of the Medical Association Thailand. 2023;106(9):843-8.

154. Punita P, Trakroo M, Palamalai SR, Subramanian SK, Bhavanani AB, Madhavan C. Randomized controlled trial of 12-week yoga therapy as lifestyle intervention in patients of essential hypertension and cardiac autonomic function tests. National Journal of Physiology, Pharmacy and Pharmacology. 2016;6(1):19-26.
155. Pushpanathan P, Trakroo M, Swaminathan RP, Madhavan C. Heart rate variability by Poincare plot analysis in patients of essential hypertension and 12-week yoga therapy. National journal of physiology, pharmacy and pharmacology. 2015;5(3):174-80.
156. Ranjbar F, Akbarzadeh F, Kazemi B, Safaeiyan A. Relaxation therapy in the background of standard antihypertensive drug treatment is effective in management of moderate to severe essential hypertension. Saudi Medical Journal. 2007;28(9):1353-6.
157. Roche LT, Hesse BM. Application of an integrative yoga therapy programme in cases of essential arterial hypertension in public healthcare. Complementary Therapies in Clinical Practice. 2014;20(4):285-90.
158. Roche LT, Barrachina MTM, Fernandez II, Betancort M. YOGA and self-regulation in management of essential arterial hypertension and associated emotional symptomatology: A randomized controlled trial. Complementary Therapies in Clinical Practice. 2017;29:153-61.
159. Rudy DR, Tosi DJ, Lewis JW. Renin Status in Association with Responsiveness to Behavioral Therapy in Hypertension. American Journal of Therapeutics. 1995;2(6):378-87.
160. Saensak S, Vutyavanich T, Somboonporn W, Srisurapanont M. Modified relaxation technique for treating hypertension in Thai postmenopausal women. Journal of Multidisciplinary Healthcare. 2013;6:373-8.
161. Sangthong B, Ubolsakka-Jones C, Pachirat O, Jones DA. Breathing Training for Older Patients with Controlled Isolated Systolic Hypertension. Medicine & Science in Sports & Exercise. 2016;48(9):1641-7.
162. Saptharishi L, Soudarssanane M, Thiruselvakumar D, Navasakthi D, Mathanraj S, Karthigeyan M, et al. Community-based Randomized Controlled Trial of Non-pharmacological Interventions in Prevention and Control of Hypertension among Young Adults. Indian Journal of Community Medicine. 2009;34(4):329-34.
163. Subramanian H, Soudarssanane MB, Jayalakshmy R, Thiruselvakumar D, Navasakthi D, Sahai A, et al. Non-pharmacological Interventions in Hypertension: A Community-based Cross-over Randomized Controlled Trial. Indian Journal of Community Medicine. 2011;36(3):191-6.
164. Schein MH, Gavish B, Herz M, Rosner-Kahana D, Naveh P, Knishkowy B, et al. Treating hypertension with a device that slows and regularises breathing: a randomised, double-blind controlled study. Journal of Human Hypertension. 2001;15(4):271-8.
165. Schein MH, Gavish B, Baevsky T, Kaufman M, Levine S, Nessing A, et al. Treating hypertension in type II diabetic patients with device-guided breathing: a randomized controlled trial. Journal of Human Hypertension. 2009;23(5):325-31.
166. Schneider RH, Staggers F, Alexander CN, Sheppard W, Rainforth M, Kondwani K, et al. A randomized controlled trial of stress reduction for hypertension in older African Americans. Hypertension. 1995;26(5):820-7.
167. Alexander CN, Schneider RH, Staggers F, Sheppard W, Clayborne BM, Rainforth M, et al. Trial of stress reduction for hypertension in older African Americans. II. Sex and risk subgroup analysis. Hypertension. 1996;28(2):228-37.
168. Barnes VA. Reduced cardiovascular and all-cause mortality in older African Americans practicing the Transcendental Meditation. Dissertation Abstracts International: Section B: The Sciences and Engineering. 1997;57(8):4999.
169. Schneider RH, Alexander CN, Staggers F, Orme-Johnson DW, Rainforth M, Salerno JW, et al. A randomized controlled trial of stress reduction in African Americans treated for hypertension for over one year. American Journal of Hypertension. 2005;18(1):88-98.
170. Schneider RH, Myers HF, Marwaha K, Rainforth MA, Salerno JW, Nidich SI, et al. Stress Reduction in the Prevention of Left Ventricular Hypertrophy: A Randomized Controlled Trial of

Transcendental Meditation and Health Education in Hypertensive African Americans. Ethnicity & Disease. 2019;29(4):577-86.
171. Castillo-Richmond A, Schneider RH, Alexander CN, Cook R, Myers H, Nidich S, et al. Effects of stress reduction on carotid atherosclerosis in hypertensive African Americans. Stroke. 2000;31(3):568-73.
172. Schneider RH, Castillo-Richmond A, Alexander CN, Myers H, Kaushik V, Aranguri C, et al. Behavioral treatment of hypertensive heart disease in African Americans: rationale and design of a randomized controlled trial. Behavioral Medicine. 2001;27(2):83-95.
173. Schneider RH, Grim C, Kotchen T, Marwaha K, Kotchen J, Salerno JW, et al. Randomized controlled trial of stress reduction with meditation and health education in black men and women with high normal and normal blood pressure. American Journal Of Preventive Cardiology. 2021;8:100279.
174. Seer P, Raeburn JM. Meditation training and essential hypertension: a methodological study. Journal of Behavioral Medicine. 1980;3(1):59-71.
175. Selvam ASB, G.; Vasanth, S.; Kumar, A. S.;. Effect of yoga therapy with varma therapy practices on systolic blood pressure and diastolic blood pressure variables among hypertensive middle aged men. Biochemical and Cellular Archives. 2020;20(1):2669-701.
176. Shapiro D, Hui KK, Oakley ME, Pasic J, Jamner LD. Reduction in drug requirements for hypertension by means of a cognitive-behavioral intervention. American Journal of Hypertension. 1997;10(1):9-17.
177. Shetty P, Reddy BK, Lakshmeesha DR, Shetty SP, Kumar GS, Bradley R. Effects of Sheetali and Sheetkari Pranayamas on Blood Pressure and Autonomic Function in Hypertensive Patients. Integrative Medicine. 2017;16(5):32-7.
178. Shetty S, Nandeesh NS, Shetty P. The Role of Integrated Approach to Yoga Therapy-Based Yoga Module in Improving Cardiovascular Functions and Lipid Profile in Hypertensive Patients: A Randomized Controlled Trial. International Journal of Yoga. 2022;15(3):215-21.
179. Shou XL, Wang L, Jin XQ, Zhu LY, Ren AH, Wang QN. Effect of T'ai Chi Exercise on Hypertension in Young and Middle-Aged In-Service Staff. Journal of Alternative & Complementary Medicine. 2019;25(1):73-8.
180. Singh VP, Khandelwal B. Effectiveness Of Yoga and Lifestyle Modification On Prehypertensive Subjects-A Randomized Controlled Trial. NeuroQuantology. 2022;20(17):1323-37.
181. Southam MA, Agras WS, Taylor CB, Kraemer HC. Relaxation training. Blood pressure lowering during the working day. Archives of General Psychiatry. 1982;39(6):715-7.
182. Agras WS, Southam MA, Taylor CB. Long-term persistence of relaxation-induced blood pressure lowering during the working day. Journal of Consulting & Clinical Psychology. 1983;51(5):792-4.
183. Agras WS, Schneider JA, Taylor CB. Relaxation training in essential hypertension: A failure of retraining in relaxation procedures. Behavior Therapy. 1984;15(2):191-6.
184. Sujatha T, Judie A. Effectiveness of a 12-week yoga program on physiopsychological parameters in patients with hypertension. International Journal of Pharmaceutical and Clinical Research. 2014;6(4):329-35.
185. Sun J, Buys N. Community-Based Mind-Body Meditative Tai Chi Program and Its Effects on Improvement of Blood Pressure, Weight, Renal Function, Serum Lipoprotein, and Quality of Life in Chinese Adults With Hypertension. American Journal of Cardiology. 2015;116(7):1076-81.
186. Supriya R, Yu AP, Lee PH, Lai CW, Cheng KK, Yau SY, et al. Yoga training modulates adipokines in adults with high-normal blood pressure and metabolic syndrome. Scandinavian Journal of Medicine & Science in Sports. 2018;28(3):1130-8.
187. Taylor CB, Farquhar JW, Nelson E, Agras S. Relaxation therapy and high blood pressure. Archives of General Psychiatry. 1977;34(3):339-42.

188. Teng XF, Wong MY, Zhang YT. The effect of music on hypertensive patients. Annual International Conference Of The IEEE Engineering In Medicine And Biology Society. 2007;2007:4649-51.
189. Thanalakshmi J, Maheshkumar K, Kannan R, Sundareswaran L, Venugopal V, Poonguzhali S. Effect of Sheetali pranayama on cardiac autonomic function among patients with primary hypertension - A randomized controlled trial. Complementary Therapies in Clinical Practice. 2020;39:101138.
190. Thiyagarajan R, Pal P, Pal GK, Subramanian SK, Trakroo M, Bobby Z, et al. Additional benefit of yoga to standard lifestyle modification on blood pressure in prehypertensive subjects: a randomized controlled study. Hypertension Research - Clinical & Experimental. 2015;38(1):48-55.
191. Tosi DJ, Rudy DR, Lewis J, Murphy MA. The psychobiological effects of cognitive experiential therapy, hypnosis, cognitive restructuring, and attention placebo control in the treatment of essential hypertension. Psychotherapy: Theory, Research, Practice, Training. 1992;29(2):274-84.
192. Tsai JC, Wang WH, Chan P, Lin LJ, Wang CH, Tomlinson B, et al. The beneficial effects of Tai Chi Chuan on blood pressure and lipid profile and anxiety status in a randomized controlled trial. Journal of Alternative & Complementary Medicine. 2003;9(5):747-54.
193. Tsai PS, Chang NC, Chang WY, Lee PH, Wang MY. Blood pressure biofeedback exerts intermediate-term effects on blood pressure and pressure reactivity in individuals with mild hypertension: a randomized controlled study. Journal of Alternative & Complementary Medicine. 2007;13(5):547-54.
194. van Montfrans GA, Karemaker JM, Wieling W, Dunning AJ. Relaxation therapy and continuous ambulatory blood pressure in mild hypertension: a controlled study. BMJ. 1990;300(6736):1368-72.
195. Venturelli M, Ce E, Limonta E, Schena F, Caimi B, Carugo S, et al. Effects of endurance, circuit, and relaxing training on cardiovascular risk factors in hypertensive elderly patients. Age. 2015;37(5):101.
196. Wadden TA. Relaxation therapy for essential hypertension: specific or nonspecific effects? Journal of Psychosomatic Research. 1984;28(1):53-61.
197. Wadden TA. Predicting treatment response to relaxation therapy for essential hypertension. Journal of Nervous and Mental Disease. 1983;171(11):683-9.
198. Walsh P, Dale A, Anderson DE. Comparison of biofeedback pulse wave velocity and progressive relaxation on essential hypertensives. Perceptual & Motor Skills. 1977;44(3):839-43.
199. Wang SZ, Li S, Xu XY, Lin GP, Shao L, Zhao Y, et al. Effect of slow abdominal breathing combined with biofeedback on blood pressure and heart rate variability in prehypertension. Journal of Alternative & Complementary Medicine. 2010;16(10):1039-45.
200. Wang MY, Chang NC, Hsieh MH, Su CT, Liu JC, Shyu YK, et al. Effect of Feedback Signal on Blood Pressure Self-regulation Capability in Individuals With Prehypertension or Stage I Hypertension: A Randomized Controlled Study. Journal of Cardiovascular Nursing. 2016;31(2):166-72.
201. Webb M, Beckstead J, Meininger J, Robinson S. Stress management for African American women with elevated blood pressure: a pilot study. Biological Research for Nursing. 2006;7(3):187-96.
202. Wen J, Su M. A Randomized Trial of Tai Chi on Preventing Hypertension and Hyperlipidemia in Middle-Aged and Elderly Patients. International Journal of Environmental Research & Public Health [Electronic Resource]. 2021;18(10):20.
203. Wolff M, Rogers K, Erdal B, Chalmers JP, Sundquist K, Midlov P. Impact of a short home-based yoga programme on blood pressure in patients with hypertension: a randomized controlled trial in primary care. Journal of Human Hypertension. 2016;30(10):599-605.

204. Wright KD, Klatt MD, Adams IR, Nguyen CM, Mion LC, Tan A, et al. Mindfulness in Motion and Dietary Approaches to Stop Hypertension (DASH) in Hypertensive African Americans. Journal of the American Geriatrics Society. 2021;69(3):773-8.
205. Wu S, Zheng C, Liu N, Deng T, Wang J, Qi L, et al. Liuzijue training improves hypertension and modulates gut microbiota profile. Frontiers in Cardiovascular Medicine. 2023;10:1075084.
206. Xiao C, Yang Y, Zhuang Y. Effect of Health Qigong Ba Duan Jin on Blood Pressure of Individuals with Essential Hypertension. Journal of the American Geriatrics Society. 2016;64(1):211-3.
207. Xu XY, Gao J, Ling D, Wang TH. Biofeedback treatment of prehypertension: analyses of efficacy, heart rate variability and EEG approximate entropy. Journal of Human Hypertension. 2007;21(12):973-5.
208. Yan ZW, Yang Z, Yang JH, Song CL, Zhao Z, Gao Y. Comparison between Tai Chi and square dance on the antihypertensive effect and cardiovascular disease risk factors in patients with essential hypertension: a 12-week randomized controlled trial. Journal of Sports Medicine & Physical Fitness. 2022;62(11):1568-75.
209. Yau KY, Law PS, Wong CN. Cardiac and Mental Benefits of Mediterranean-DASH Intervention for Neurodegenerative Delay (MIND) Diet plus Forest Bathing (FB) versus MIND Diet among Older Chinese Adults: A Randomized Controlled Pilot Study. International Journal of Environmental Research & Public Health [Electronic Resource]. 2022;19(22):08.
210. Yen LL, Patrick WK, Chie WC. Comparison of relaxation techniques, routine blood pressure measurements, and self-learning packages in hypertension control. Preventive Medicine. 1996;25(3):339-45.
211. Young DR, Appel LJ, Jee S, Miller ER, 3rd. The effects of aerobic exercise and T'ai Chi on blood pressure in older people: results of a randomized trial. Journal of the American Geriatrics Society. 1999;47(3):277-84.
212. Yuenyongchaiwat K, Changsri K, Harnmanop S, Namdaeng P, Aiemthaisong M, Pongpanit K, et al. Effects of slow breathing training on hemodynamic changes, cardiac autonomic function and neuroendocrine response in people with high blood pressure: A randomized control trial. Journal of Bodywork and Movement Therapies. 2024;37:136-41.
213. Yung P, French P, Leung B. Relaxation training as complementary therapy for mild hypertension control and the implications of evidence-based medicine. Complementary Therapies in Nursing & Midwifery. 2001;7(2):59-65.
214. Zanini CR, Jardim PC, Salgado CM, Nunes MC, Urzeda FL, Carvalho MV, et al. Music therapy effects on the quality of life and the blood pressure of hypertensive patients. Arquivos Brasileiros de Cardiologia. 2009;93(5):534-40.
215. Ziv A, Vogel O, Keret D, Pintov S, Bodenstein E, Wolkomir K, et al. Comprehensive Approach to Lower Blood Pressure (CALM-BP): a randomized controlled trial of a multifactorial lifestyle intervention. Journal of Human Hypertension. 2013;27(10):594-600.
216. Zurawski RM, Smith TW, Houston BK. Stress management for essential hypertension: comparison with a minimally effective treatment, predictors of response to treatment, and effects on reactivity. Journal of Psychosomatic Research. 1987;31(4):453-62.
217. Evans T, Brown H. Road traffic crashes: operationalizing equity in the context of health sector reform. Injury Control and Safety Promotion. 2003;10(1-2):11-2.
218. O'Neill J, Tabish H, Welch V, Petticrew M, Pottie K, Clarke M, et al. Applying an equity lens to interventions: using PROGRESS ensures consideration of socially stratifying factors to illuminate inequities in health. Journal of Clinical Epidemiology. 2014;67(1):56-64.
219. Hollingshead A. Four Factor Index of Social Status, Unpublished working paper. 1975.
