# Supplementary material for: Effectiveness of stress management and relaxation interventions for management of hypertension and prehypertension: systematic review and network meta-analysis
Source: BMJ Med. 2025 Apr 8;4(1):e001098. doi: 10.1136/bmjmed-2024-001098 (PMC12164322; doi:10.1136/bmjmed-2024-001098)
Supplement: Supplementary file 3 [file bmjmed-2024-001098.reviewer_comments.pdf]

PEER REVIEW HISTORY

BMJ Medicine publishes all reviews undertaken for accepted manuscripts. Reviewers are asked to complete a checklist review form and are provided with free text boxes to elaborate on their assessment. These free text comments are reproduced below.

ARTICLE DETAILS

**TITLE (PROVISIONAL)**	Effectiveness of stress management and relaxation interventions for the management of hypertension and pre-hypertension: a systematic review and network meta-analysis
**AUTHORS**	Webster, Katie; Halicka, Monika; Bowater, Russell; Parkhouse, Thomas; Stanescu, Dara; Punniyakotty, Athitya Vel; Savović, Jelena; Huntley, Alyson; Dawson, Sarah; Clark, Christopher; Johnson, Rachel; Higgins, Julian Piers Thomas; Caldwell, Deborah

VERSION 1 - REVIEW

**REVIEWER NAME**	Fujiwara, Takeshi
**REVIEWER AFFILIATION**	University of Oxford, Nuffield Department of Primary Care Health Sciences
**REVIEWER CONFLICT OF INTEREST**	None
**DATE REVIEW RETURNED**	17-Oct-2024

**GENERAL COMMENTS**	Summary<br>The aim of this systematic review and meta-analysis was to investigate the impact of relaxation and stress management for individuals with hypertension and pre-hypertension. This meta-analysis revealed that most relaxation interventions had beneficial effect on blood pressure for individuals with hypertension and pre-hypertension at up to 3 months' follow-up.<br><br>Comments<br>I enjoyed reading this paper of this great work. I think this manuscript is well-written and provides an important contribution for the effect of the relaxation or stress management on blood pressure levels. Taking these points into consideration, I would like to make some comments.<br><br>・I understand that relaxation or stress management is effective over the short period of 3 months, but as stated in the limitations of this study, I think the clinical impact of these results should be described in more detail.<br>・In the stratified analysis, the authors have set 75 years old as the cutoff value. What is the rational for this cutoff value? I think that the relaxation or stress management possible and its intensity would differ depending on age. Why not set a younger age, such as 65 years old, as the cutoff age point?<br>Also, is there a significant difference in the effects between those with high and low cardiovascular disease risk?<br>・This is mentioned in the later data extraction, but I think it would be better to provide more details in the Interventions section. In addition, the details of interventions should be described. I think this point is important.

	・How did this study examine ethnical differences in the effects of relaxation or stress management on blood pressure?

**REVIEWER NAME**	Barbato, Antonio
**REVIEWER AFFILIATION**	University of Naples Federico II School of Medicine and Surgery
**REVIEWER CONFLICT OF INTEREST**	None
**DATE REVIEW RETURNED**	26-Oct-2024

**GENERAL COMMENTS**	Dear Authors,<br>This review aims to evaluate the effectiveness of non-pharmacological stress management and relaxation interventions on hypertension or pre-hypertension and was conducted according to a protocol published in the PROSPERO database.<br><br>The review with its comprehensive methodology fails to find strong scientific evidence to support the role of relaxation and stress management techniques on blood pressure for people with hypertension, apart from a possible short-term beneficial effect.<br><br>The strengths and limitations of this meta-analysis are clearly reported. The level of English is good. This meta-analysis in summary highlights the importance of research in this field as most of the available research lacks a solid methodological basis. I have no particular comments to make: in my opinion this meta-analysis is well conducted and the results are clearly reported, I congratulate the authors for the scientific rigor with which they conducted this study.

**REVIEWER NAME**	Lai, Tai-Shuan
**REVIEWER AFFILIATION**	National Taiwan University Hospital, Department of Internal Medicine
**REVIEWER CONFLICT OF INTEREST**	None
**DATE REVIEW RETURNED**	11-Nov-2024

**GENERAL COMMENTS**	This systematic review provides valuable insights into how relaxation and stress management interventions affect blood pressure in adults with hypertension and pre-hypertension. While the analysis is comprehensive, including 182 studies, and employs sound statistical methods, several areas warrant attention:<br>1. Intervention Standardization:<br>The study would benefit from more precise definitions of relaxation and stress management techniques. While Table 1 lists these interventions, it lacks detailed protocols for implementation and specific metrics for measuring outcomes. This information is crucial for reproducibility and practical application.<br>2. Quality Assessment Considerations:<br>Given that the quality assessment revealed many studies to be of lower methodological rigor, it would be valuable to conduct separate subgroup analyses comparing results between high- and low-quality studies. This would help understand how study quality might influence the reported outcomes.<br>3. Language Restriction Justification:<br>The authors should provide a clear rationale for including only

	English-language publications, as this limitation could introduce language bias and potentially exclude relevant studies from non-English speaking regions.<br>4. Network Meta-Analysis Assumptions:<br>The primary analyses rely heavily on passive and non-specific comparators as key network connections. To ensure the validity of the transitivity assumption, it's essential to examine and report the variation in baseline blood pressure measurements, particularly among these crucial comparator groups. This would strengthen the reliability of the indirect effect calculations.<br>5. Treatment Ranking Analysis:<br>To enhance the comparative effectiveness analysis, incorporating SUCRA (Surface Under the Cumulative Ranking) calculations would provide more precise treatment rankings. This additional analysis would help clinicians and researchers better understand the relative effectiveness of different interventions.

**REVIEWER NAME**	Riley, Richard
**REVIEWER AFFILIATION**	University of Birmingham, Institute of Applied Health Research
**REVIEWER CONFLICT OF INTEREST**	None
**DATE REVIEW RETURNED**	20-Jan-2025

**GENERAL COMMENTS**	This is a very well written and conducted study. The authors have clearly worked hard to identify and synthesise this body of evidence, and have communicated their methodology and results very well. The statistical methods are appropriate and generally well detailed. My generally minor comments are as follows:<br>1) Abstract: there is no mention of heterogeneity and no results about heterogeneity, or what meta-analysis model assumptions were made (common-effect, random-effects etc)<br>2) Abstract: the results section is very unclear as to whether the summary results are based on a pairwise comparison or a NMA model<br>3) What synthesising the treatment effect results, it is not clear what the priority order is in terms of taking a study's effect based on the end point data results, or the change score results. Also, often studies report an ANCOVA model (linear regression adjusted for baseline) which is an even better approach to derive effect estimates for continuous outcomes; was this not extracted or prioritised?<br>4) Great to see the PPI but I was surprised that the PPI group were not involved in the choice about grouping of interventions into 'similar interventions'. Was this done but not mentioned perhaps?<br>5) I appreciate more details of the analyses are given in the supp material, but we do need the main choice of prior distributions to be reported in the main article. Also, are the 95% CrIs derived using HDPI approach or a standard 2.5% and 97.5% approach.<br>6) Further, the authors should clarify their approach for the pairwise meta-analysis: they simply say they used meta in R, but we need to know if this was a random effects model, how it was estimated, the approach to derive 95% CIs, etc.<br>7) The authors specify subgroup analyses in the methods section, but not how the subgroup estimates were extracted. For example age of >=75 and <75 is mentioned, but what if studies did not report an age subgroup result, or used a 65 cut-point? It is further confused by the results section saying that they sought subgroups of studies where ON AVERAGE patients were >= 75 years.

	8) The authors appear to adhere to PRISMA-NMA, but this is not explicitly mentioned in the methods section.<br>9) It is good to see that transitivity was considered, but the authors are not clear what steps followed after they consider this was likely a concern. They say in regards to different timepoints used across studies that 'Transitivity across treatment comparisons was considered plausible.' – but not what the consequences of this were. For example, did they not consider grouping into similar timepoints to help address this? That seems to be what happened based on the sub-sections of the results.<br>10) I struggled to ascertain how many studies were contributing to each NMA at each time-point. Fig 3 gives the number of direct trials, but not the total number on the NMA.<br>11) I would help to have some information on how many trials provided results toward all three time-groupings. For further research, would it rather be beneficial to model the correlation amongst time-points (e.g. in a multivariate NMA accounting for correlation of time-points in the same analysis)? Something for the discussion perhaps.<br>12) Lastly, I suggest the supplementary results tables are labelled, e.g., Table S1 rather than Table 1 etc to avoid confusion<br><br>In summary, this is an excellent manuscript and I hope these comments are constructive for the authors moving forwards.

VERSION 1 – AUTHOR RESPONSE

Reviewer 1	
I understand that relaxation or stress management is effective over the short period of 3 months, but as stated in the limitations of this study, I think the clinical impact of these results should be described in more detail.	Thanks for the comment. We have added some discussion around this in the final section (page 13): "Hypertension is a chronic condition, likely to require long-term medications or behavioural changes. As such, interventions that are used for a brief period, or provide only short-term benefits are unlikely to be clinically useful. At present, however, there are too few studies to assess whether any beneficial effects of relaxation are maintained when the techniques are practised for longer then three months"
In the stratified analysis, the authors have set 75 years old as the cutoff value. What is the rational for this cutoff value? I think that the relaxation or stress management possible and its intensity would differ depending on age. Why not set a younger age, such as 65 years old, as the cutoff age point?	Thank you for the comment. We considered that age may moderate the effect of relaxation treatments, and hence included this as a pre-specified subgroup analysis. The rationale for this cut-off was to align with current NICE guidelines for England and Wales, where subgrouping according to age ≥75 was conducted.
Also, is there a significant difference in the effects between those with high and low cardiovascular disease risk?<br>・This is mentioned in the later data extraction, but I think it would be better to provide more	We agree that the use of QRISK scores (or similar) to stratify individuals with lower and higher cardiovascular risk would be of interest. We did not specify this in our review protocol, therefore did not systematically extract data on

details in the Interventions section. In addition, the details of interventions should be described. I think this point is important.	cardiovascular risk scores. Nonetheless, we can comment that data on cardiovascular risk scores were not widely reported across the body of evidence – and would not be available for many of the studies, which were conducted in the late 20th century, before development of these tools. We have added a statement regarding this to the review (page 12):<br><br>“Over time, clinical assessment of hypertension has moved away from focusing on a single risk factor (blood pressure), towards an approach that includes multiple risk factors in an assessment of overall cardiovascular risk (using tools such as QRISK). Ideally, we would have assessed the impact of relaxation techniques for those at different levels of cardiovascular risk, as assessed with these tools. However, many of the studies included in this review were published before the emergence of these tools, and more recent studies did not routinely report information on cardiovascular risk.”.<br><br>Table S1 does provide detailed descriptions of the intervention categories.
How did this study examine ethnical differences in the effects of relaxation or stress management on blood pressure?	Information on ethnicity was extracted as part of the assessment of PROGRESS+ characteristics. These data are reported narratively in the EDI section of the paper (page 11) and in further detail in the supplementary materials (p180-181 and table S33). Surprisingly, only 55 studies provided information on ethnicity – those that did included mainly white participants, with 7 studies that exclusively recruited black/African American participants, and 6 studies that only recruited Chinese participants. For this paper, subgroup analyses were planned at the study level and would not have been informative given the small number of studies and a variety of relaxation techniques included.<br><br>The Cochrane handbook advises that analysis of subgroups should be clinically relevant, and that investigations of heterogeneity when there are few studies is of questionable value (Deeks JJ, Higgins JPT, Altman DG, McKenzie JE, Veroniki AA (editors). Chapter 10: Chapter 10: Analysing data and undertaking meta-analyses. In: Higgins JPT, Thomas J, Chandler J, Cumpston M, Li T, Page MJ, Welch VA (editors). Cochrane

	Handbook for Systematic Reviews of Interventions version 6.5. Cochrane, 2024.Sections 10.11.5.1 and 10.11.5.4). We are aware that current UK recommendations for first-line anti-hypertensive treatment differs according to ethnicity, and that this may be due to different responses to medication. However, we are uncertain that a person's ethnicity would modify the physiological effect of a yoga or breathing intervention, for example, within the context of a clinical study.
Reviewer 2	
I have no particular comments to make: in my opinion this meta-analysis is well conducted and the results are clearly reported, I congratulate the authors for the scientific rigor with which they conducted this study.	Many thanks for the positive feedback.
Reviewer 3	
Intervention Standardization:<br>The study would benefit from more precise definitions of relaxation and stress management techniques. While Table 1 lists these interventions, it lacks detailed protocols for implementation and specific metrics for measuring outcomes. This information is crucial for reproducibility and practical application.	Thank you for highlighting this important point. We agree that defining the different techniques is important, both for understanding the paper and for implementation of the interventions. Unfortunately, detailed descriptions, protocols for intervention implementation, and specific metrics for measuring outcomes were insufficiently reported in the majority of the primary studies. As our paper is secondary research, our ability to provide this level of detail is limited by the original study authors' own descriptions. We have added this limitation to the discussion section (page 12): "Descriptions of relaxation interventions were sometimes incomplete or sparse, meaning that it was challenging to group studies that used equivalent relaxation techniques. We were unable to contact all study authors to establish full details of the interventions used."<br><br>As the reviewer notes, Table S1 does include descriptions of the intervention and comparator groups. As with any meta-analysis, there is a balance between grouping similar interventions in meaningful ways and artificially creating an intervention group lacking clinical relevance, for the sake of analysis. Our grouping definitions are fairly inclusive, to ensure that an analysis was

	feasible, and that the results would also be clinically relevant.<br><br>There was no variation in the metrics for measuring outcomes, as systolic and/or diastolic blood pressure was expressed in mmHg. Table S3 specifies the BP measurement methods used (e.g. office attended / unattended, home-based measurements). We agree that it is important for authors of primary studies to describe their measurement methods clearly, but don't consider that this impacts on the reproducibility of the work in the review.
Quality Assessment Considerations:<br>Given that the quality assessment revealed many studies to be of lower methodological rigor, it would be valuable to conduct separate subgroup analyses comparing results between high- and low-quality studies. This would help understand how study quality might influence the reported outcomes.	Thanks – we agree that the risk of bias in the primary studies is of concern. Indeed, our primary analysis only included studies we judged to be at lower risk of bias, and the conclusions we report in the main text of our paper are based on the primary analysis. However, we note that - as no study was judged to be at low risk of bias - the primary analysis was based on those judged to have 'some concerns' regarding risk of bias. We examined the impact of including all studies - combining those at some concerns and high risk of bias – in a sensitivity analysis and reported the findings in the supplementary materials (table S10). We noted in the original manuscript (page 10) that the effect estimates from the sensitivity analyses including studies at high risk of bias were similar to the primary analyses, but were estimated with more precision.<br><br>As an example, when considering short-term data for systolic blood pressure in hypertension, 56 studies were included in the main analyses (some concern regarding risk of bias, with 2 studies disconnected from the network; mean sample size 62.7 participants). A further 83 studies were added for the sensitivity analysis incorporating all studies (mean sample size 66.4 participants).
Language Restriction Justification:<br>The authors should provide a clear rationale for including only English-language publications, as this limitation could introduce language bias and potentially exclude relevant studies from non-English speaking regions.	We agree this is a limitation of the review, and have noted this in the discussion section of our revised manuscript (page 12). Unfortunately, resources for this review did not allow us to extend the search to non-English language papers.

	However, there is recent evidence that restricting evidence syntheses of interventions to English-language publications does not necessarily introduce reporting biases and can be a viable methodological 'shortcut' (https://doi.org/10.1016/j.jclinepi.2021.04.012). In addition, although ours is the first to compare multiple relaxation interventions in a NMA and there are analytical differences between our paper and previously published systematic reviews, our results are comparable to those that did not restrict by language. For example:<br><br>do Amoral 2016 http://dx.doi.org/10.1016/j.ijcard.2016.03.197 : music versus passive comparator: MD –6.58mmHg (-9.38, -3.79)<br><br>Wang 2016 http://dx.doi.org/10.1161/JAHA.115.002562 : Traditional Chinese exercise [meditative movement] versus passive comparator : MD -9.12mmHg (-16.38, 1.86)
Network Meta-Analysis Assumptions:<br>The primary analyses rely heavily on passive and non-specific comparators as key network connections. To ensure the validity of the transitivity assumption, it's essential to examine and report the variation in baseline blood pressure measurements, particularly among these crucial comparator groups. This would strengthen the reliability of the indirect effect calculations.	The reviewer raises two important points about (i) transitivity and (ii) the potential implications of variation in baseline BP measurements for relative effect estimates. We have added new text to page 8 of the revised manuscript to state: "The transitivity assumption across treatment comparisons was considered plausible after assessment of baseline characteristics, including blood pressure."<br><br>However, it is not clear why the reviewer considers the passive and non-specific comparators to be of particular concern. Our review included only randomized controlled trials, where randomization should ensure similar baseline blood pressure between treatment arms within each study, regardless of the type of comparator used. Furthermore, our analyses were performed on intervention effects on the change from baseline which, in part, accounts for differences in baseline scores between studies.

	Of course, the relative intervention effects on the change from baseline may still depend on baseline values and this could be investigated using meta-regression. However, we are reluctant to use baseline blood pressure to inform analysis decisions, as blood pressure among people diagnosed with hypertension would be expected to vary for many reasons – for example, depending on their medication status. However, we did examine this in subgroup analysis (see page 10 for results) and found that most relaxation techniques appeared to have beneficial effects on blood pressure in the short-term, regardless of medication status.<br><br>As a starting point for our assessment of transitivity, we considered the principle of joint randomisability. That is, that each participant could have been randomised to any of the interventions or trials included – analogous to a multi-arm trial being feasible within population. We anticipated that baseline BP may be an important factor to consider here, and consequently we pre-specified separate analyses for pre-hypertensive and hypertensive populations. This distinction was operationalised according to our review eligibility criteria (see page 4 of revised manuscript) and we did not rely on trialist definitions or their statements of trial eligibility criteria. Therefore, the same criteria were applied across all the studies included in the review.<br><br>Where reported, baseline BP was extracted for each study. It was not reported in 4 (systolic) and 5 (diastolic) studies out of 179 eligible for analysis. Prior to analysis we examined baseline blood pressure across trials and comparisons, across the body of evidence. Within each population (hypertension and pre-hypertension) this was assessed as being comparable. Within each population, we also examined factors that may affect variability of baseline BP, such as medication status, and we judged the distribution across comparisons to be comparable. The dataset used for analysis will be available on request, if readers wish to explore this further. [Summary baseline characteristics are reported

	in table S3, and we have now added data on baseline BP to this table].
Treatment Ranking Analysis:<br>To enhance the comparative effectiveness analysis, incorporating SUCRA (Surface Under the Cumulative Ranking) calculations would provide more precise treatment rankings. This additional analysis would help clinicians and researchers better understand the relative effectiveness of different interventions.	Thanks for this suggestion. However, we did not report treatment rankings in our submission and it is therefore not clear why the reviewer thinks that SUCRA would provide 'more precise treatment rankings'. Although we do agree that rankings may be useful to clinicians they can also be very misleading, especially when heterogeneity is present and this has been the topic of much methodological research (http://dx.doi.org/10.2147/clep.S69660, http://dx.doi.org/10.1016/j.jclinepi.2018.02.009, http://dx.doi.org/10.1177/2047487318767199). On a practical level, we did not pre-specify that we would report treatment rankings in our prospectively registered protocol and we are not aware of reporting guidance that mandates rankings. As such, we have not amended the manuscript.
Reviewer 4	
Abstract: there is no mention of heterogeneity and no results about heterogeneity, or what meta-analysis model assumptions were made (common-effect, random-effects etc)	Thank you for highlighting this. We have now added a description of model assumptions and heterogeneity to the abstract.
Abstract: the results section is very unclear as to whether the summary results are based on a pairwise comparison or a NMA model	Thank you – we have added a comment to indicate that reported results are from a NMA model (page 2, results paragraph 2).
What synthesising the treatment effect results, it is not clear what the priority order is in terms of taking a study's effect based on the end point data results, or the change score results. Also, often studies report an ANCOVA model (linear regression adjusted for baseline) which is an even better approach to derive effect estimates for continuous outcomes; was this not extracted or prioritised?	Thank you, and we apologise that this was not clearer in the main text. We have now added the following [page 5]:<br><br>"For synthesis, we prioritised estimates of arm-level mean change from baseline (CfB) and standard errors (SE) in accordance with guidance from the NICE guidelines Technical Support Unit."<br><br>The priority order can be found in the supplementary materials (Data extraction and missing data, page 35). The TSU guidance does indeed suggest that extraction of estimates from ANCOVA models be prioritised. We did extract reported model-based effect estimates, but these were uncommon (only ten, of 180, studies

	reported such data). In addition, where ANCOVA results were reported, models also adjusted for additional factors (not just baseline BP), and these factors were not consistent across studies. In such cases we considered that it was preferable to analyse the next-recommended arm-based mean change-from-baseline data.
Great to see the PPI but I was surprised that the PPI group were not involved in the choice about grouping of interventions into 'similar interventions'. Was this done but not mentioned perhaps?	Thanks for the comment. Our PPI group were recruited as people with lived experience of hypertension and not necessarily because they had experience of relaxation interventions. Our PPI group were enrolled to provide input into the review protocol and to inform the conclusions and summarising stages of the work, and therefore their allocated time on the project was limited. Unfortunately we did not involve them in grouping of interventions. This process took extensive work amongst the review team to read and re-read the articles, in order to identify the main themes. With hindsight, this process could have been usefully informed by the views of complementary medicine practitioners (such as yoga and tai chi instructors or meditation facilitators), and we will consider this for future work.
I appreciate more details of the analyses are given in the supp material, but we do need the main choice of prior distributions to be reported in the main article. Also, are the 95% CrIs derived using HDPI approach or a standard 2.5% and 97.5% approach.	We agree and apologise for the omission in the main text. We have now added details on the prior distributions and calculation of the 95% CrI to the text of the methods [on page 6]:<br><br>"We specified uninformative ('vague') prior distributions for the intercept (Normal[μ=0, σ=100]), treatment effect (Normal[μ=0, σ=100]), and a weakly informative between study heterogeneity parameter (Half-Normal[μ=0, σ=10]). Further information on checking assumptions, convergence, and the statistical models fitted are reported in the supplementary materials (tables S4 to S9). Intervention effects are reported as mean differences in change from baseline in systolic or diastolic blood pressure relative to the reference intervention ('passive comparator') with the limits of the 95% credible intervals (CrI) defined as the 2.5th and 97.5th percentiles of the posterior distribution concerned."
Further, the authors should clarify their approach for the pairwise meta-analysis: they	We apologise for the lack of clarity here. Although we intended to conduct pairwise meta-

simply say they used meta in R, but we need to know if this was a random effects model, how it was estimated, the approach to derive 95% CIs, etc.	analyses, due to the paucity of data we considered that estimates of between-study heterogeneity would be inaccurate. Therefore, we have presented results from individual studies only, without conducting meta-analysis. We have amended the text to indicate this:<br><br>"If network meta-analysis was not possible, we aimed to present pairwise random effects meta-analyses instead, using meta in R(25). However, for comparisons informed by three or fewer studies we did not present the pooled result due to concerns that the heterogeneity estimate may be unreliable."
The authors specify subgroup analyses in the methods section, but not how the subgroup estimates were extracted. For example age of >=75 and <75 is mentioned, but what if studies did not report an age subgroup result, or used a 65 cut-point? It is further confused by the results section saying that they sought subgroups of studies where ON AVERAGE patients were >= 75 years.	We have clarified in the manuscript [page 7] that we took a study-level approach to subgroup analysis , i.e. grouping together studies where the average age of participants was 75 years or over, and studies where the average age was less than 75 years. We have amended the description in the methods section to make this clear:<br><br>"Age. Studies were grouped according to the average age of participants; 75 years or over versus <75 years."
The authors appear to adhere to PRISMA-NMA, but this is not explicitly mentioned in the methods section.	Thanks – we have now added this to the methods section [page 4]
It is good to see that transitivity was considered, but the authors are not clear what steps followed after they consider this was likely a concern. They say in regards to different timepoints used across studies that 'Transitivity across treatment comparisons was considered plausible.' – but not what the consequences of this were. For example, did they not consider grouping into similar timepoints to help address this? That seems to be what happened based on the sub-sections of the results.	We apologise for the lack of clarity regarding our approach to assessing transitivity. The sentence noted by the reviewer was intended to indicate that we had considered study characteristics to be similar across the body of evidence, such that we considered transitivity to be a reasonable assumption. As such, we have amended the text to state: "The transitivity assumption across treatment comparisons was considered plausible." [page 8, Hypertension primary analysis, paragraph 2].<br><br>We have also clarified the methods [page 6, Evidence synthesis and NMA model fitting and selection] to state:<br><br>"If there was evidence of intransitivity, pairwise or narrative synthesis would have been conducted instead of a NMA."

	"Random-effects models assuming a common between-study heterogeneity parameter were fitted for all analyses using multinma, where sufficient data were available, and transitivity was considered plausible."<br><br>We think our original phrasing was confusing - we did not carry out subgroup analysis by time point because of an explicit concern with transitivity. Rather, grouping of outcomes into the three timepoints was planned before the analysis stage. Indeed, this was specified a priori in the full protocol available from [Effectiveness of stress management and relaxation interventions for the management of hypertension and pre-hypertension: a systematic review and network meta-analysis - NIHR Funding and Awards].
I struggled to ascertain how many studies were contributing to each NMA at each time-point. Fig 3 gives the number of direct trials, but not the total number on the NMA.	We apologise that this was not clear. Although we did describe the number of studies contributing to each NMA in the main text of the review when reporting the results (54 studies for the short-term follow up [page 8], 21 studies for medium-term follow-up [page 9]) we have now also included this information in the caption for figures 2, 3 and 4. We hope that will make it easier for readers to have this information reported alongside the forest plots for each analysis. Tables S5-S9 also present the number of studies included in each primary, sensitivity, and subgroup network meta-analysis.
It would help to have some information on how many trials provided results toward all three time-groupings. For further research, would it rather be beneficial to model the correlation amongst time-points (e.g. in a multivariate NMA accounting for correlation of time-points in the same analysis)? Something for the discussion perhaps.	Thanks for the comment. We completely agree that it would be interesting to model the correlation across different time points and hoped we may be able to do such an analysis. However, multiple follow-up times were reported relatively infrequently and, unfortunately, no studies reported data at all three time points. Only ten of the analysed studies reported data at both short- and medium-term follow-up, and a single study reported data at both medium- and long-term follow up. Consequently we did not have sufficient data to investigate the correlation amongst time-points. As suggested by the reviewer, we have added a comment on this to the discussion [page 12].

Lastly, I suggest the supplementary results tables are labelled, e.g., Table S1 rather than Table 1 etc to avoid confusion	Thank you - we have now indicated supplementary tables and figures with the prefix S (e.g. table S1)
